# Supplementary material for: Dinuclear gold-catalyzed divergent dechlorinative radical borylation of gem-dichloroalkanes
Source: Nat Commun. 2024 May 2;15:3721. doi: 10.1038/s41467-024-48085-8 (PMC11066019; doi:10.1038/s41467-024-48085-8)
Supplement: Supplementary file 1 — Supplementary Information [file 41467_2024_48085_MOESM1_ESM.pdf]

Supplementary Information

Dinuclear Gold-Catalyzed Divergent Radical Dechloroborylation of *gem*-Dichloroalkanes

Cheng-Long Ji[1], Hongliang Chen[1], Qi Gao[1], Jie Han[1], Weipeng Li[1], Jin Xie[1]*

[1]State Key Laboratory of Coordination Chemistry, Jiangsu Key Laboratory of Advanced Organic Materials, Chemistry and Biomedicine Innovation Center (ChemBIC), School of Chemistry and Chemical Engineering, Nanjing University, Nanjing 210023, China

Email: xie@nju.edu.cn

Table of contents

Supplementary Methods 2

General Information 2

Optimization of the Reaction Conditions 3

General Procedure for Divergent Dechlorinative Borylation 9

Synthetic Applications 11

Analytical Data of Compounds 16

Examining Functional-Group Compatibility 45

Supplementary Discussion 54

Mechanistic Studies 54

Computational Investigations 75

Copies of NMR Spectra 80

Supplementary References 185

Supplementary Methods

General Information

All reactions were conducted in oven- or flame-dried glassware under an atmosphere of argon unless otherwise noted. Unless otherwise noted, all reagents were used as received and handled under air atmosphere. DEF and alcohol solvents are chemically pure reagents purchased from commercial suppliers and used as received and other solvents are anhydrous. Chloroform-d_1 was purchased from Adamas-beta®.

NMR spectra were recorded on a Bruker Ultra-shield 400, 500 and 600 MHz spectrometer. ^{1}H NMR, ^{13}C NMR and ^{19}F NMR are recorded on an NMR spectrometer with $CDCl_3$ as solvent. Chemical shifts of ^{1}H, ^{13}C, and ^{19}F NMR spectra are reported in parts per million (ppm). The ^{19}F NMR spectra is {1H} decoupled and the ^{13}C NMR spectra is {1H} decoupled. The residual solvent signals were used as standard, and the chemical shifts were converted to the corresponding scale ($CDCl_3$: δ H = 7.26 ppm, δ C = 77.00 ppm). All coupling constants (*J* values) were reported in hertz (Hz). Multiplicities are reported as follows: singlet (s), doublet (d), doublet of doublets (dd), triplet (t), quintet (quint), and multiplet (m). Gas chromatographic (GC) analyses were performed on a GC equipped with a flame-ionization detector and an Rtx@-65 (30 m × 0.32 mm ID × 0.25 μm df) column. GC-MS analyses were performed on a GC-MS with an EI mode. HRMS (ESI) was determined on the Micro-mass Q-TOF instrument. The IR spectrum was recorded on a Brucker Alpha FT/IR instrument. UV-Vis spectra were recorded with a double beam spectrophotometer Shimadzu UV2600 equipped with a deuterium lamp (190-350 nm), a halogen lamp (330-900 nm) and a photomultiplier (Hamamatsu R928). The blue LEDs (45 W, λ = 380-550 nm, λ_{max} = 466 nm) was purchased from Kessil. Schlenk tubes (10 mL and 100 mL) were purchased from synthware. Toppette was purchased from DLAB Scientific Co., Ltd. The compound names were generated by ChemDraw Professional 20.0 software (PerkinElmer) according to the guidelines specified by the International Union of Pure and Applied Chemistry (IUPAC).

All reagents were purchased from commercial suppliers, Aladdin, Adamas-beta®, TCI (Shanghai) Development Co., Ltd, Energy Chemical, J & K scientific Ltd., Bide Pharmatech Ltd, Alfa-Aesar, Shanghai Haohong Scientific Co., Ltd and Sigma-Aldrich unless otherwise noted.

Optimization of the Reaction Conditions

Screening of reaction conditions for gold-catalyzed *gem*-diborylation

Supplementary Table 1. Screening of photocatalyst[a]

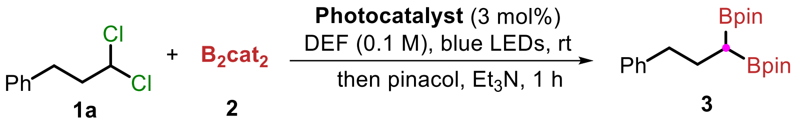

Entry	Photocatalyst	Yield (%) [b]
1	**PC1**	78 (73)[c]
2	**PC2**	trace
3	**PC3**	45
4	**PC4**	n.d.
5	**PC5**	n.d.
6	**PC6**	n.r.
7	$[Au(dcpm)Br]_2$	40
8	$[Au(dcpm)PF_6]_2$	57
9	$[Au(dcpm)BF_4]_2$	53
10	$[Au(dcpm)SCN]_2$	25

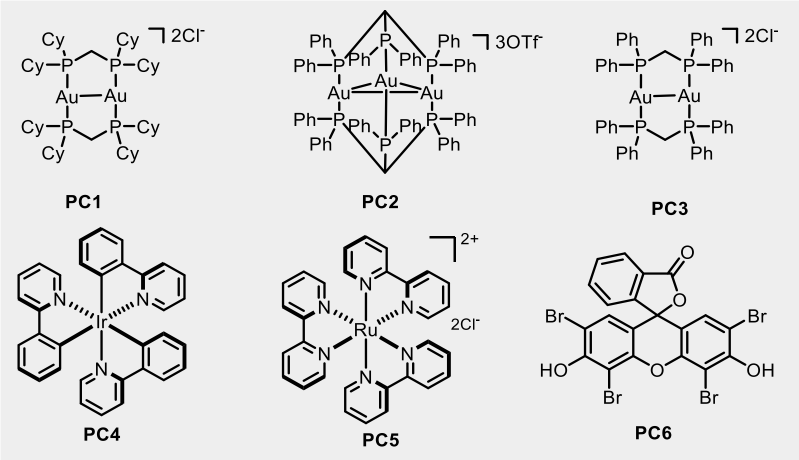

[a]Standard reaction conditions: **PC** (3 mol%), **1a** (0.2 mmol), B_2cat_2 **2** (0.8 mmol), DEF (2.0 mL), blue LEDs (λ_{max}=466 nm), ambient temperature, 24 h; then pinacol (4.0 equiv), Et_3N (0.5 mL), 1 h. [b]GC yield using biphenyl as an internal standard. [c]Isolated yield. n.d. = not detected. n.r. = no reaction.

Supplementary Table 2. Screening of boron source[a]

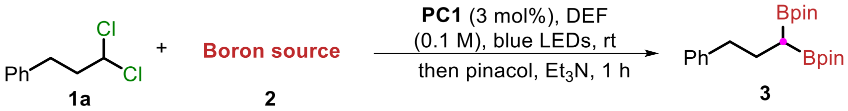

Entry	Boron source	Yield (%) [b]
1	B_2cat_2	78 (73)[c]
2	B_2pin_2	0
3	B_2neop_2	0
4	B_2hex_2	0
5	B_2pai_2	0
6	Bpin-Bdan	0

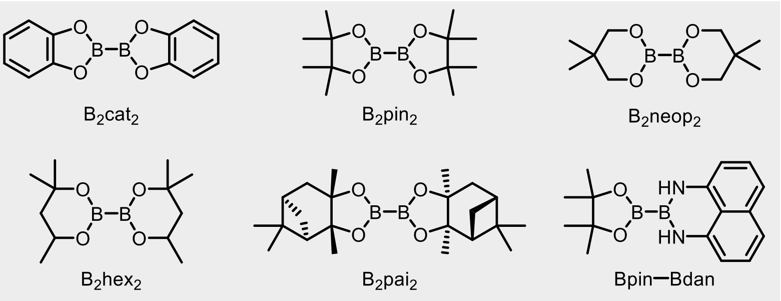

[a]Standard reaction conditions: **PC1** (3 mol%), **1a** (0.2 mmol), Boron source **2** (0.8 mmol), DEF (2.0 mL), blue LEDs (λ_{max}=466 nm), ambient temperature, 24 h. [b]GC yield using biphenyl as an internal standard. [c]Isolated yield.

Supplementary Table 3. Screening of different solvents[a]

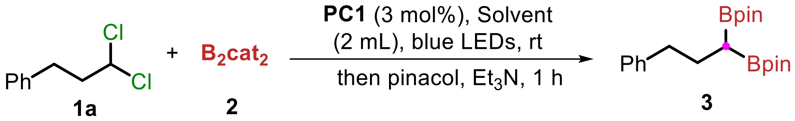

Entry	Solvents	Yield (%) [b]
1	DEF	78 (73)[c]
2	DMF	47
3	DMAc	33
4	HMPA	trace
5	DMPU	n.r.
6	NMP	27
7	EA	n.r.
8	MeCN	trace
10	DEF/EA (1:1)	52
11	DEF/MeOH (1:1)	n.r.
12	DEF/MeCN (1:1)	trace

[a]Standard reaction conditions: **PC1** (3 mol%), **1a** (0.2 mmol), B_2cat_2 **2** (0.8 mmol), Solvent (2.0 mL), blue LEDs (λ_{max}=466 nm), ambient temperature, 24 h; then pinacol (4.0 equiv), Et_3N (0.5 mL), 1 h. [b]GC yield using biphenyl as an internal standard.DEF, Diethylformamide. EA, Ethyl acetate. DMF = *N*,*N*-dimethylformamide; NMP = *N*-methyl-2-pyrrolidone. DMAc = *N*,*N*-Dimethylacetamide. DMPU = 1,3-Dimethyl-3,4,5,6-tetrahydro-2(1H)-pyrimidinone. HMPA = Hexamethylphosphoramide. n.d. = not detected. n.r. = no reaction.

Supplementary Table 4. Screening of B_2cat_2 loading[a]

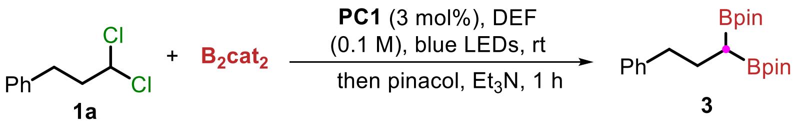

Entry	B_2cat_2 loading	Yield (%) [b]
1	1.5 equiv.	28
2	2.0 equiv.	41
3	2.5 equiv.	55
4	3.0 equiv.	62
5	3.5 equiv.	68
6	4.0 equiv.	78 (73)[c]
7	4.5 equiv.	65

[a]Standard reaction conditions: **PC** (3 mol%), **1a** (0.2 mmol), B_2cat_2 **2** (x equiv.), DEF (2.0 mL), blue LEDs (λ_{max}=466 nm), ambient temperature, 24 h; then pinacol (4.0 equiv), Et_3N (0.5 mL), 1 h. [b]GC yield using biphenyl as an internal standard. [c]Isolated yield.

Supplementary Table 5. Control experiments

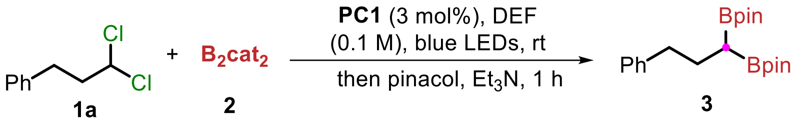

Entry	Variation of standard conditions	Yield (%)[b]
1	none	78 (73)[c]
2	Purple LEDs	52
3	Without **PC1**	n.r.
4	Without light irradiation	n.r.

[a]Standard reaction conditions: **PC1** (3 mol%), **1a** (0.2 mmol), B_2cat_2 **2** (0.8 mmol), DEF (2.0 mL), blue LEDs (λ_{max}=466 nm), ambient temperature, 24 h; then pinacol (4.0 equiv), Et_3N (0.5 mL), 1 h. [b]GC yield using biphenyl as an internal standard. [c]Isolated yield. n.r. = no reaction.

Screening of reaction conditions for gold-catalyzed hydroborylation

Supplementary Table 6. Screening of base [a]

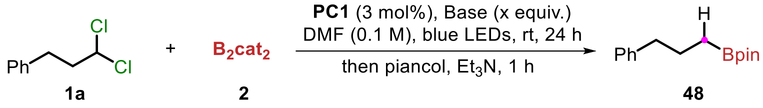

Entry	Base (3.0 equiv)	Yield (%) [b]
1	Et_3N	30
2	NaOAc	15
3	Cs_2CO_3	trace
4	MeOLi	10
5	DBU	trace
6	TMEDA	trace
7	TMG	28
8	BTMG	32
9	K_3PO_4	n.d.
10	KF	45
11	KOAc	trace
12	K_2CO_3	n.d.
13	KHF_2	trace
14	LiF	20
15	NaF	32
16	CsF	40
17	4-Phenylpyridine	n.r.
18	0.5 equiv of KF	trace
19	1.0 equiv of KF	19
20	2.0 equiv of KF	34
21	2.5 equiv of KF	40
22	3.5 equiv of KF	52
23	4.0 equiv of KF	47
24	5.0 equiv of KF	38

[a]Reaction conditions: **1a** (0.2 mmol), **2** (0.5 mmol), **PC1** (3 mol%), Base (0.6 mmol), DMF (2 mL), blue LEDs (λ_{max}=466 nm), ambient temperature, 24 h; then pinacol (4.0 equiv), Et_3N (0.5 mL),1 h.

[b]GC yield using biphenyl as an internal standard. n.d. = not detected. n.r. = no reaction.

Supplementary Table 7. Screening of different solvents [a]

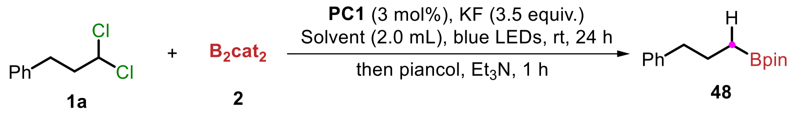

Entry	Solvent	Yield (%)[b]
1	MeCN	n.r.
2	DMF/MeCN (1:1)	n.d.
3	DMF/EA (1:1)	n.d.
4	DMF/THF (1:1)	trace
5	DMF/MeOH (1:1)	56
6	DMF/1,4-Dioxane (1:1)	trace
7	DMF/DCE (1:1)	trace
8	DMF/$PhCF_3$ (1:1)	10
9	DMF/EtOH (1:1)	25
10	DMF/DMSO (1:1)	n.r.
11	DMF/PhCl (1:1)	trace
12	DMF/MA (1:1)	trace
13	DMF/EtOH (1:1)	40
14	DMF/^{i}PrOH (1:1)	54
15	DMF/ ^{n}Pentanol (1:1)	62

[a]Reaction conditions: **1a** (0.2 mmol), **2** (0.5 mmol), **PC1** (3 mol%), KF (0.7 mmol), Solvent (2 mL), blue LEDs (λ_{max}=466 nm), ambient temperature, 24 h; then pinacol (4.0 equiv.), Et_3N (0.5 mL),1 h.

[b]GC yield using biphenyl as an internal standard. n.d. = not detected. n.r. = no reaction.

Supplementary Table 8. Screening of photocatalyst for hydroborylation[a]

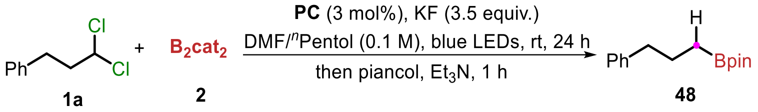

Entry	Photocatalyst	Yield (%)[b]
1	[Au(dcpm)Cl]$_2$	62
2	[Au$_3$(tppm)$_2$]Cl$_3$	trace
3	*fac*-Ir(ppy)$_3$	n.d.
4	4CzIPN	n.d.
5	Ru(bpy)$_3$Cl$_2$	n.d.
6	[Au(dcpm)Cl]$_2$ (1 mol%)	48
7	[Au(dcpm)Cl]$_2$ (2 mol%)	56
8	[Au(dcpm)Cl]$_2$ (5 mol%)	48
9	[Au(dcpm)Cl]$_2$ (10 mol%)	30

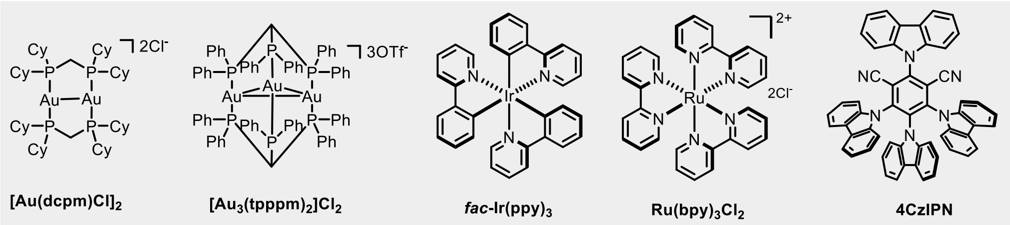

[a]Reaction conditions: **1a** (0.2 mmol), **2** (0.5 mmol), **PC** (3 mol%), KF (0.7 mmol), DMF/^{n}Pentol (1:1, 2 mL), blue LEDs (λ_{max}=466 nm), ambient temperature, 24 h; then pinacol (4.0 equiv), Et$_3$N (0.5 mL),1 h. [b]GC yield using biphenyl as an internal standard. n.d. = not detected. n.r. = no reaction.

Supplementary Table 9. Screening of B_2cat_2 loading[a]

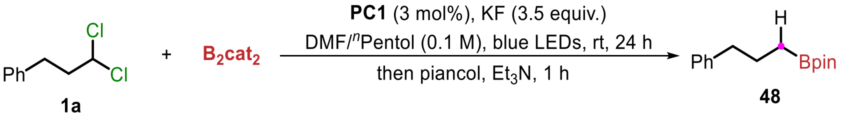

Entry	B_2cat_2 loading	Yield (%)[b]
1	1.0 equiv.	38
2	1.5 equiv.	42
3	2.0 equiv.	54
4	2.5 equiv.	62
5	3.0 equiv.	68 (62)[c]
6	4.0 equiv.	60
7	5.0 equiv.	57

[a]Reaction conditions: **1a** (0.2 mmol), **2** (x equiv.), **PC1** (3 mol%), KF (0.7 mmol), DMF/^{n}Pentanol (1:1, 2 mL), blue LEDs (λ_{max}=466 nm), ambient temperature, 24 h; then pinacol (4.0 equiv), Et$_3$N (0.5 mL),1 h. [b]GC yield using biphenyl as an internal standard. [c]Isolated yield.

General Procedure for Divergent Dechlorinative Borylation

General Procedure A: gold-catalyzed dechlorinative geminal diborylation

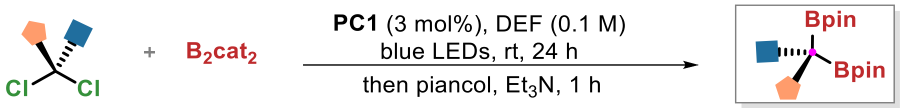

1 2 *Gem-diborylation*

To an oven-dried 10 mL sealed tube, **PC1** (3 mol%, 0.006 mmol, 7.7 mg), *gem*-dichloroalkanes **1** (0.2 mmol), B_2cat_2 **2** (0.8 mmol, 190 mg, 4 equiv.) and DEF (2 mL) are successively added and the tube is backfilled with argon three times under Schlenk line. The resulting reaction mixture is vigorously stirred under the irradiation of blue LEDs (distance app. 4.0 cm from the bulb) at ambient temperature (the fan was used to keep the reaction temperature around ambient temperature) for 24 h. Subsequently, a solution of pinacol (0.8 mmol, 4.0 equiv., 94.4 mg) in triethylamine (0.5 mL) is added to the resulting crude and the reaction mixture is kept stirring at room temperature for another 1 h. Then, saturated brine water (10 mL) is added to the reaction mixture and the aqueous layer is extracted with ethyl acetate (3 × 5 mL). The organic layers are combined, dried over Na_2SO_4, filtered, and concentrated. The crude residue is directly purified quickly by silica column chromatography (eluted with ethyl acetate/petroleum ether) to yield the desired *gem*-diborylation products. (Note: generally, the flash column chromatography should be done fast and is better to finish within 10 min; otherwise the product could be decomposed to give a lower yield.)

General Procedure B: gold-catalyzed dechlorinative monoborylation of *gem*-dichloroalkanes

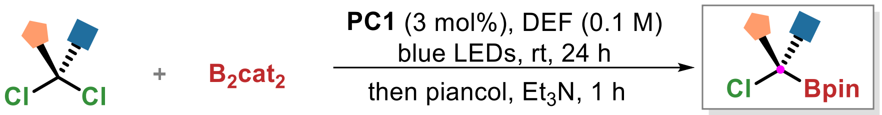

1 2 *Monoborylation*

To an oven-dried 10 mL sealed tube, **PC1** (3 mol%, 0.006 mmol, 7.7 mg), *gem*-dichloroalkanes **1** (0.2 mmol), B_2cat_2 **2** (0.8 mmol, 190 mg, 4 equiv.) and DEF (2 mL) are successively added and the tube is backfilled with argon three times under Schlenk line. The resulting reaction mixture is vigorously stirred under the irradiation of blue LEDs (distance app. 4.0 cm from the bulb) at ambient temperature (the fan is used to keep the reaction temperature around ambient temperature) for 24 h. Subsequently, a solution of pinacol (0.8 mmol, 4.0 equiv., 94.4 mg) in triethylamine (0.5 mL) is added to the resulting crude and the reaction mixture is kept stirring at room temperature for another 1 h. Then, saturated brine water (10 mL) is added to the reaction mixture and the aqueous layer is extracted with ethyl acetate (3 × 5 mL) three times. The organic layers are combined, dried over Na_2SO_4, filtered, and concentrated. The crude residue is directly purified quickly by silica column chromatography (eluted with ethyl acetate/petroleum ether) to yield the desired monoborylation products. (Note: generally, the flash column chromatography should be done fast and is better to finish within 10 min; otherwise the product could be decomposed to give a lower yield.)

General Procedure C: gold-catalyzed dechlorinative hydroborylation of *gem*-dichloroalkanes

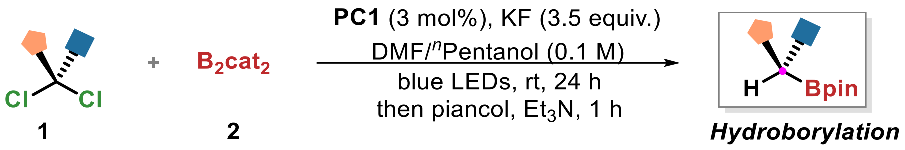

To an oven-dried 10 mL sealed tube, **PC1** (3 mol%, 0.006 mmol, 7.7 mg), *gem*-dichloroalkanes **1** (0.2 mmol), B_2cat_2 **2** (0.6 mmol, 142 mg, 3 equiv.), KF (0.7 mmol, 41 mg, 3.5 equiv.) and DMF/^{n}Pentanol (1:1, 2 mL) are successively added and the tube is backfilled with argon three times under Schlenk line. The resulting reaction mixture is vigorously stirred under the irradiation of blue LEDs (distance app. 4.0 cm from the bulb) at ambient temperature (the fan is used to keep the reaction temperature around ambient temperature) for 24 h. Subsequently, a solution of pinacol (0.8 mmol, 4.0 equiv., 94.4 mg) in triethylamine (0.5 mL) is added to the resulting crude and the reaction mixture is kept stirring at room temperature for another 1 h. Then, saturated brine water (10 mL) is added to the reaction mixture, and the aqueous layer is extracted with ethyl acetate (3 × 5 mL) three times. The organic layers are combined, dried over Na_2SO_4, filtered, and concentrated. The crude residue is directly purified quickly by silica column chromatography (eluted with ethyl acetate/petroleum ether) to yield the desired hydroborylation products. (Note: the flash column chromatography should be done fast, and is better to finish within 10 min; otherwise the product could be decomposed to give a lower yield.)

Synthetic Applications

General Procedure D for 10 mmol scaled-up reactions

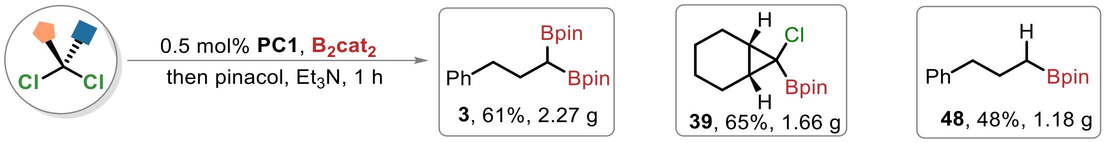

10 mmol scale for synthesis of 3:

To an oven-dried 100 mL sealed tube, **PC1** (0.5 mol%, 0.05 mmol, 64 mg), (3,3-dichloropropyl)benzene (10 mmol, 1.88 g), **2** (40 mmol, 9.48 g, 4 equiv.) and DEF (50 mL) are successively added and the tube is backfilled with argon three times under Schlenk line. The resulting reaction mixture is vigorously stirred under the irradiation of blue LEDs (distance app. 4.0 cm from the bulb) at ambient temperature (the fan is used to keep the reaction temperature around ambient temperature) for 36 h. Subsequently, a solution of pinacol (40 mmol, 4.0 equiv., 4.72 g) in triethylamine (25 mL) is added to the resulting crude and the reaction mixture is kept stirring at room temperature for another 1 h. Then, saturated brine water (100 mL) is added to the reaction mixture and the aqueous layer is extracted with ethyl acetate (3 × 50 mL). The organic layers are combined, dried over Na_2SO_4, filtered, and concentrated. The crude residue is directly purified quickly by silica column chromatography (eluted with ethyl acetate/petroleum ether) to yield the desired *gem*-diborylation product **3** (2.27 g, 61% yield).

10 mmol scale for synthesis of 39:

To an oven-dried 100 mL sealed tube, **PC1** (0.5 mol%, 0.05 mmol, 64 mg), 7,7-dichlorobicyclo[4.1.0]heptane (10 mmol, 1.65 g), **2** (40 mmol, 9.48 g, 4 equiv.) and DEF (50 mL) are successively added and the tube is backfilled with argon three times under Schlenk line. The resulting reaction mixture is vigorously stirred under the irradiation of blue LEDs (distance app. 4.0 cm from the bulb) at ambient temperature (the fan is used to keep the reaction temperature around ambient temperature) for 36 h. Subsequently, a solution of pinacol (40 mmol, 4.0 equiv., 4.72 g) in triethylamine (25 mL) is added to the resulting crude, and the reaction mixture is kept stirring at room temperature for another 1 h. Then, saturated brine water (100 mL) is added to the reaction mixture, and the aqueous layer is extracted with ethyl acetate (3 × 50 mL). The organic layers are combined, dried over Na_2SO_4, filtered, and concentrated. The crude residue is directly purified quickly by silica column chromatography (eluted with ethyl acetate/petroleum ether) to yield the desired monoborylation product **39** (1.66 g, 65% yield).

10 mmol scale for synthesis of 48:

To an oven-dried 100 mL sealed tube, **PC1** (0.5 mol%, 0.05 mmol, 64 mg), (3,3-dichloropropyl)benzene (10 mmol, 1.88 g), **2** (30 mmol, 7.11 g, 3 equiv.) KF (35 mmol, 2.03 g, 3.5 equiv.) and DMF/^{n}Pentanol (1:1, 50 mL) are successively added and the tube is backfilled with argon three times under Schlenk line. The resulting reaction mixture is vigorously stirred under the irradiation of blue LEDs (distance app. 4.0 cm from the bulb) at ambient temperature (the fan is used to keep the reaction temperature around ambient temperature) for 36 h. Subsequently, a

solution of pinacol (40 mmol, 4.0 equiv., 4.72 g) in triethylamine (25 mL) is added to the resulting crude and the reaction mixture is kept stirring at room temperature for another 1 h. Then, saturated brine water (100 mL) is added to the reaction mixture and the aqueous layer is extracted with ethyl acetate (3 ×50 mL). The organic layers are combined, dried over Na_2SO_4, filtered, and concentrated. The crude residue is directly purified quickly by silica column chromatography (eluted with ethyl acetate/petroleum ether) to yield the desired hydroborylation product **48** (1.18 g, 48% yield).

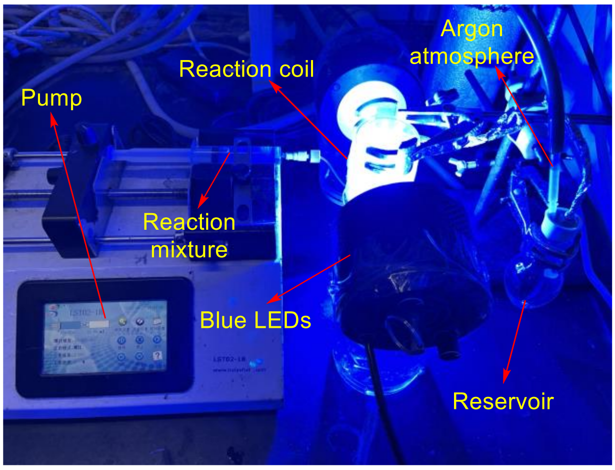

Supplementary Figure 1. Continuous-flow setup

General Procedure E: gold-catalyzed *gem*-diborylation, monoborylation and hydroborylation of *gem*-dichloroalkanes in continuous-flow

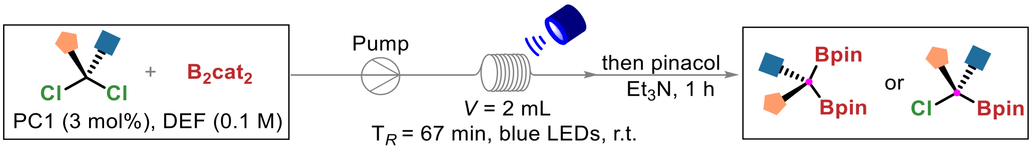

In the glovebox, the corresponding *gem*-dichloroalkanes **1** (1.0 mmol), **2** (4.0 equiv., 0.95 g), **PC1** (3 mol%, 38.5 mg), DEF (10 mL) are mixed in a 25 mL flask and the flask is swirled to achieve homogeneity. The liquid is then taken up with a syringe (50 mL) and mounted on a syringe pump. As shown in Supplementary Figure **1**, the flow apparatus is purged with degassed argon to remove the air first. The syringe is connected to the flow apparatus with a back-pressure regulator. The tubing (HPFA, O.D. 1/16”, I.D. 0.03”, 5.2 m, volume = 2.0 mL) is rounded on a glass cylinder (I.D. = 5.0 cm). The reaction is placed into the center of 45 W blue LED at ambient temperature (the temperature was controlled by fan and the distance between the tubing and light is around 4 cm). The flow apparatus itself is set up with T_R = 67 min, flow rate =0.03 mL/min. After approximately 15 min of equilibration, a solution of pinacol (4 mmol, 4.0 equiv., 0.472 g) in triethylamine (2.5 mL) is added to the resulting crude and the reaction mixture is kept stirring at room temperature for another 1 h. Finally, the solutions are diluted with H_2O and ethyl acetate. The layers are separated and the aqueous layer is extracted with ethyl acetate three times. The combined organic layers are washed with brine, dried with Na_2SO_4, filtered, and evaporated. The crude is purified via column chromatography on silica gel to afford the corresponding *gem*-diborylation or monoborylation product.

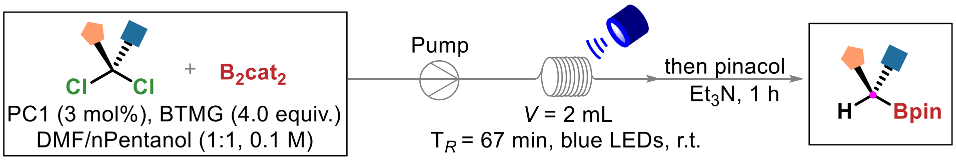

In the glovebox, the corresponding *gem*-dichloroalkanes **1** (1.0 mmol), **2** (3.0 equiv., 0.71 g), **PC1** (3 mol%, 38.5 mg) and BTMG (4.0 equiv., 0.55 mL), DMF/^{n}Pentanol (1:1, 10 mL) are mixed in a 25 mL flask and the flask is swirled to achieve homogeneity. The liquid is taken up with a syringe (50 mL) and mounted on a syringe pump. As shown in Supplementary Figure **1**, the flow apparatus is purged with degassed argon to remove the air first. The syringe is connected to the flow apparatus with a back-pressure regulator. The tubing (HPFA, O.D. 1/16", I.D. 0.03", 5.2 m, volume = 2.0 mL) is rounded on a glass cylinder (I.D. = 5.0 cm). The reaction is placed into the center of 45 W blue LED at ambient temperature (the temperature was controlled by fan and the distance between the tubing and light is around 4 cm). The flow apparatus itself is set up with T_R = 67 min, flow rate =0.03 mL/min. After approximately 15 min of equilibration, a solution of pinacol (4 mmol, 4.0 equiv., 0.472 g) in triethylamine (2.5 mL) is added to the resulting crude and the reaction mixture is kept stirring at room temperature for another 1 h. Finally, the solutions are diluted with H_2O and ethyl acetate. The layers are separated and the aqueous layer is extracted with ethyl acetate three times. The combined organic layers are washed with brine, dried with Na_2SO_4, filtered, and evaporated. The crude is purified via column chromatography on silica gel to afford the corresponding hydroborylation product.

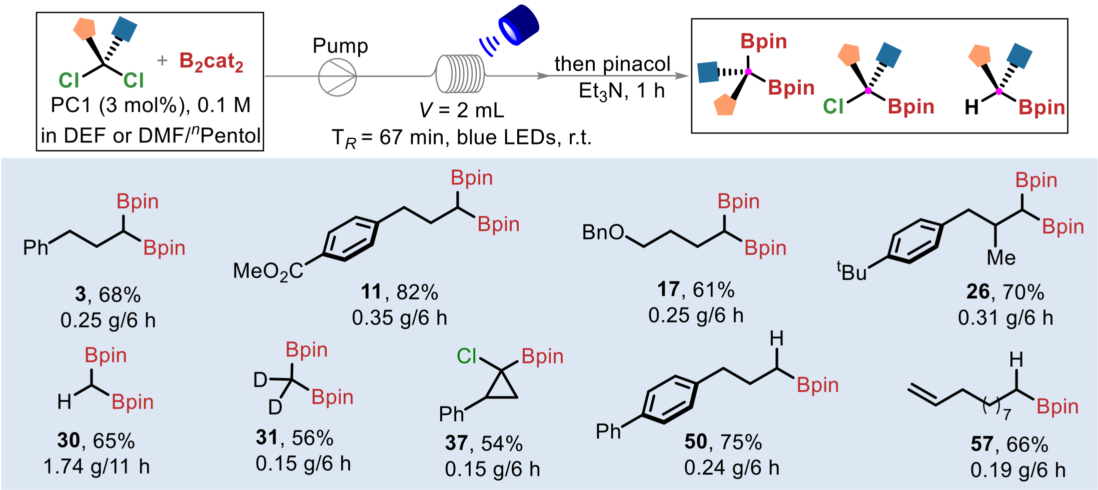

Supplementary Figure 2. Gram scale synthesis of diverse borylation products

General Procedure F: downstream transformations of the products

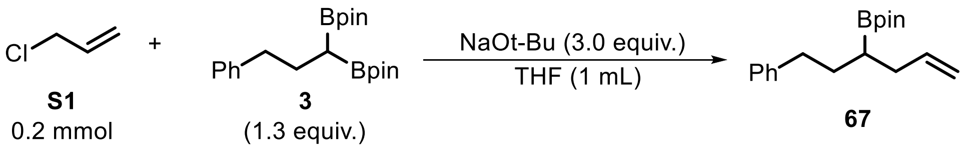

According to the literature[1], in the glove box, a 4-mL screw-capped vial with magnetic stir bar is charged with **3** (0.26 mmol), **S1** (0.20 mmol) and THF (1.0 mL), followed by the NaO^t-Bu (0.60

mmol). The vial is sealed with a polypropylene cap, removed from the glove box, and allowed to stir at room temperature for 3 hours. Upon completion, the reaction mixture is diluted with diethyl ether (2 mL), filtered through a silica gel plug, rinsed with diethyl ether, and concentrated in vacuo. The crude reaction mixture is purified on silica gel to afford the desired product **67**.

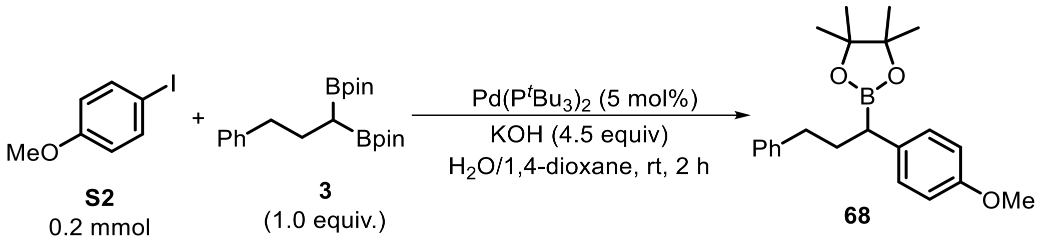

According to the literature[2], in the glove box, a 4-mL screw-capped vial is charged with Pd(P^{t}Bu$_3$)$_2$ (2.5 mg), **3** (0.2 mmol), **S2** (0.20 mmol), 1,4-dioxane (1.5 mL) and a magnetic stirring bar. The vial is sealed with a cap containing a PTFE septum with silicone seal and removed from the glove box. KOH (40% aq, 4.5 equiv.) is added to the vial using a syringe and the mixture is then stirred at room temperature for 2 h. The reaction is extracted with EtOAc (3 x 10 mL) and the combined extracts are washed with brine and dried with Na_2SO_4. The crude product is purified using silica gel flash column chromatography to afford the desired product **68**.

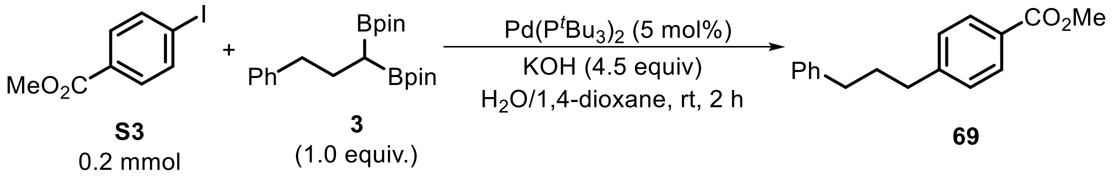

According to the literature[2,3], in the glove box, a 4-mL screw-capped vial is charged with Pd(P^{t}Bu$_3$)$_2$ (2.5 mg), **3** (0.2 mmol), **S3** (0.20 mmol), 1,4-dioxane (1.5 mL) and a magnetic stirring bar. The vial is sealed with a cap containing a PTFE septum with silicone seal and removed from the glove box. KOH (40% aq, 4.5 equiv.) is added to the vial using a syringe and the mixture is then stirred at room temperature for 2 h. The reaction is extracted with EtOAc (3 x 10 mL) and the combined extracts are washed with brine and dried with Na_2SO_4. The crude product is purified using silica gel flash column chromatography to afford the desired product **69**.

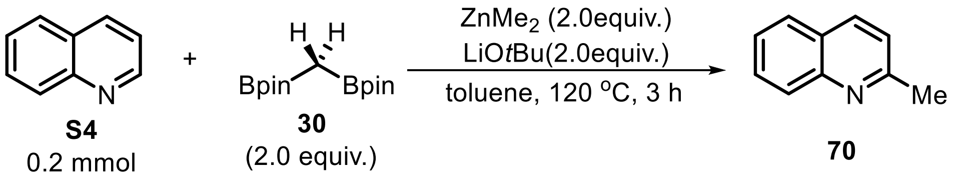

According to the literature[4], in the glove-box, **S4** (0.20 mmol), LiO*t*Bu (0.40 mmol), **30** (0.40 mmol), $ZnMe_2$ (2.0 M in toluene, 0.40 mmol) and anhydrous toluene (2.0 mL) are added to a 4.0 mL dram vial with a magnetic stir bar. The vial is sealed with a PTFE/silicone-lined septum cap, removed from the glove box, and stirred at 120 °C for 3 h. The reaction mixture is filtered through celite and washed with EtOAc. The combined organic layers are dried over Na_2SO_4, filtered and concentrated under reduced pressure. The crude mixture is purified by column chromatography to afford the

desired product **70**.

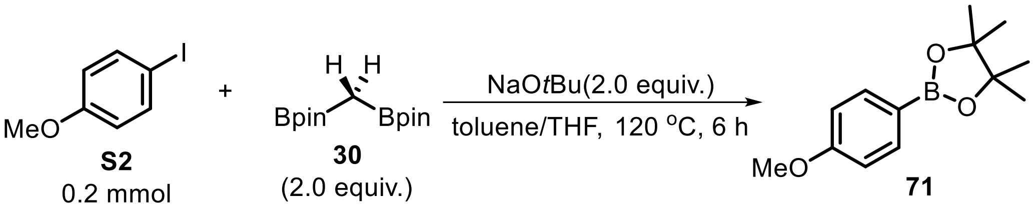

According to the literature[5], in the glove box, to an oven-dried 4-dram vial equipped with a Teflon coated magnetic stir bar, **S2** (0.20 mmol), **30** (0.40 mmol) and NaO*t*Bu (0.40 mmol) and toluene/THF (2.0 mL, 1:1) are added. The vial is sealed with a PTFE/silicone-lined septum cap and stirred at 120 °C for 6 h. The organic phase is washed with brine (20 mL) and the aqueous layer is extracted with CH_2Cl_2 (25 mL x 3). The combined organic layers are dried over Na_2SO_4, filtered and concentrated under reduced pressure. The crude mixture is purified by column chromatography to afford the desired product **71**.

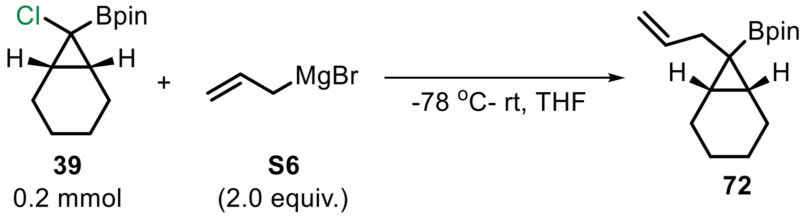

An oven-dried 10-mL Schlenk tube containing a Teflon stir bar is charged with **39** (0.2 mmol), Anhydrous THF (0.6 mL), the resulting solution is stirred for 5 min at -78 °C, and **S6** (2.0 equiv.) is added dropwise, and the reaction mixture is allowed to warm to room temperature for 12 h. The reaction mixture is diluted with H_2O followed by extraction with EtOAc, dried with anhydrous Na_2SO_4 and concentrated in vacuo. The residue is purified by flash column chromatography on silica gel to give the product **72**.

Analytical Data of Compounds

2,2'-(3-Phenylpropane-1,1-diyl)bis(4,4,5,5-tetramethyl-1,3,2-dioxaborolane) **(3)**

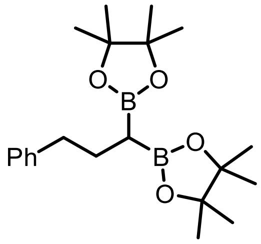

The title compound **3** was synthesized according to **General Procedure A**, and it was purified by column chromatography on silica gel (petroleum ether/ethyl acetate = 10:1), 54.3 mg, 73% yield, white solid. All recorded spectroscopic data matched those previously reported in the literature[6]. The reaction was performed on a 1 mmol scale according to **General Procedure E**, 253 mg, 68% yield.

^{1}H NMR (400 MHz, $CDCl_3$) δ 7.23 (d, *J* = 7.5 Hz, 2H), 7.20 – 7.10 (m, 3H), 2.61 – 2.56 (m, 2H), 1.88 – 1.82 (m, 2H), 1.24 (s, 12H), 1.23 (s, 12H), 0.81 (t, *J* = 7.9 Hz, 1H).

^{13}C NMR (101 MHz, $CDCl_3$) δ 142.9, 128.6, 128.1, 125.5, 83.0, 38.7, 28.0, 24.9, 24.5. The signal of the α-B-carbon was not observed.

^{11}B NMR (160 MHz, $CDCl_3$) δ 34.0.

2,2'-(3-(p-Tolyl)propane-1,1-diyl)bis(4,4,5,5-tetramethyl-1,3,2-dioxaborolane) **(4)**

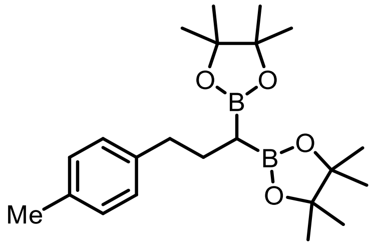

The title compound **4** was synthesized according to **General Procedure A**, and it was purified by column chromatography on silica gel (petroleum ether/ethyl acetate = 10:1), 43.2 mg, 56% yield, colorless liquid. All recorded spectroscopic data matched those previously reported in the literature[7].

^{1}H NMR (500 MHz, $CDCl_3$) δ 7.09 – 7.02 (m, 4H), 2.58 – 2.51 (m, 2H), 2.29 (s, 3H), 1.87 – 1.78 (m, 2H), 1.23 (s, 12H), 1.22 (s, 12H), 0.80 (t, *J* = 7.9 Hz, 1H).

^{13}C NMR (126 MHz, $CDCl_3$) δ 139.9, 134.8, 128.8, 128.4, 82.9, 38.2, 28.1, 24.9, 24.5, 21.0. The signal of the α-B-carbon was not observed.

^{11}B NMR (128 MHz, $CDCl_3$) δ 34.5.

2,2'-(3-(4-(Tert-butyl)phenyl)propane-1,1-diyl)bis(4,4,5,5-tetramethyl-1,3,2-dioxaborolane) **(5)**

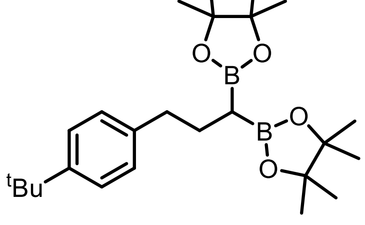

The title compound **5** was synthesized according to **General Procedure A**, and it was purified by

column chromatography on silica gel (petroleum ether/ethyl acetate = 10:1), 66.4 mg, 78% yield, colorless liquid.

^{1}H NMR (400 MHz, CDCl$_3$) δ 7.29 – 7.25 (m, 2H), 7.14 – 7.09 (m, 2H), 2.60 – 2.53 (m, 2H), 1.89 – 1.82 (m, 2H), 1.30 (s, 9H), 1.23 (s, 12H), 1.22 (s, 12H), 0.81 (t, *J* = 7.9 Hz, 1H).

^{13}C NMR (101 MHz, CDCl$_3$) δ 148.1, 139.8, 128.2, 124.9, 82.9, 38.1, 34.2, 31.4, 27.8, 24.9, 24.5, 10.4 (br, low intensity).

^{11}B NMR (128 MHz, CDCl$_3$) δ 33.8.

IR (ATR): ν = 3353, 2974, 2347, 1087, 1047, 906, 879, 727, 646 cm^{-1}.

HRMS m/z (ESI) calcd for $C_{25}H_{43}B_2O_4$ (M + H)$^+$: 429.3342; found: 429.3334.

2,2'-(3-(4-Methoxyphenyl)propane-1,1-diyl)bis(4,4,5,5-tetramethyl-1,3,2-dioxaborolane) **(6)**

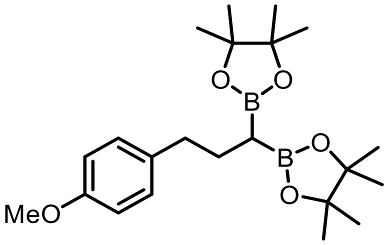

The title compound **6** was synthesized according to **General Procedure A**, and it was purified by column chromatography on silica gel (petroleum ether/ethyl acetate = 10:1), 60 mg, 75% yield, colorless liquid. All recorded spectroscopic data matched those previously reported in the literature[7].

^{1}H NMR (400 MHz, CDCl$_3$) δ 7.12 – 7.06 (m, 2H), 6.83 – 6.76 (m, 2H), 3.76 (s, 3H), 2.56 – 2.49 (m, 2H), 1.86 – 1.77 (m, 2H), 1.23 (s, 12H), 1.22 (s, 12H), 0.79 (t, *J* = 8.0 Hz, 1H).

^{13}C NMR (101 MHz, CDCl$_3$) δ 157.5, 135.1, 129.4, 113.5, 82.9, 55.2, 37.7, 28.1, 24.8, 24.5, 10.4 (br, low intensity).

^{11}B NMR (128 MHz, CDCl$_3$) δ 34.2.

2,2'-(3-([1,1'-Biphenyl]-4-yl)propane-1,1-diyl)bis(4,4,5,5-tetramethyl-1,3,2-dioxaborolane) **(7)**

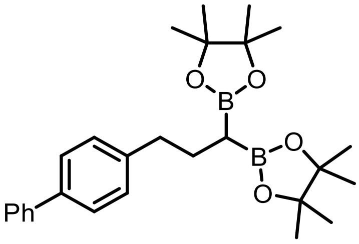

The title compound **7** was synthesized according to **General Procedure A**, and it was purified by column chromatography on silica gel (petroleum ether/ethyl acetate = 10:1), 59.5 mg, 66% yield, colorless liquid.

^{1}H NMR (400 MHz, CDCl$_3$) δ 7.61 – 7.56 (m, 2H), 7.50 (d, *J* = 8.1 Hz, 2H), 7.43 (t, *J* = 7.6 Hz, 2H), 7.32 (t, *J* = 7.3 Hz, 1H), 7.27 (d, *J* = 8.1 Hz, 2H), 2.69 – 2.63 (m, 2H), 1.95 – 1.88 (m, 2H), 1.26 (s, 12H), 1.25 (s, 12H), 0.86 (t, *J* = 7.9 Hz, 1H).

^{13}C NMR (101 MHz, CDCl$_3$) δ 142.1, 141.3, 138.5, 129.0, 128.6, 127.0, 126.9, 126.8, 83.0, 38.3, 27.9, 24.9, 24.5, 10.5 (br, low intensity).

^{11}B NMR (128 MHz, CDCl$_3$) δ 33.4.

IR (ATR): ν = 3328, 2975, 2358, 1316, 1138, 1087, 1046, 908, 879, 730 cm^{-1}.

HRMS m/z (ESI) calcd for $C_{27}H_{39}B_2O_4$ (M + H)$^+$: 449.3029; found: 449.3024.

***2,2'-(3-(4-Fluorophenyl)propane-1,1-diyl)bis(4,4,5,5-tetramethyl-1,3,2-dioxaborolane)* (8)**

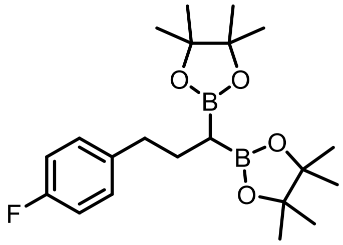

The title compound **8** was synthesized according to **General Procedure A**, and it was purified by column chromatography on silica gel (petroleum ether/ethyl acetate = 10:1), 71.8 mg, 92% yield, colorless liquid. All recorded spectroscopic data matched those previously reported in the literature[7].

^{1}H NMR (400 MHz, CDCl$_3$) δ 7.15 – 7.07 (m, 2H), 6.96 – 6.86 (m, 2H), 2.60 – 2.50 (m, 2H), 1.87 – 1.75 (m, 2H), 1.23 (s, 12H), 1.22 (s, 12H), 0.77 (t, *J* = 7.8 Hz, 1H).

^{13}C NMR (101 MHz, CDCl$_3$) δ 161.1 (d, *J* = 242.5 Hz), 138.5 (d, *J* = 3.2 Hz), 129.8 (d, *J* = 7.7 Hz), 114.8 (d, *J* = 21.0 Hz), 83.0, 37.8, 28.1, 24.9, 24.5, 10.4 (br, low intensity).

^{19}F NMR (376 MHz, CDCl$_3$) δ -118.4.

^{11}B NMR (128 MHz, CDCl$_3$) δ 33.5.

2,2'-(3-(4-Chlorophenyl)propane-1,1-diyl)bis(4,4,5,5-tetramethyl-1,3,2-dioxaborolane) (9)

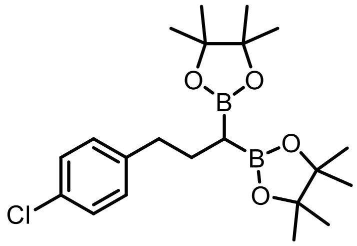

The title compound **9** was synthesized according to **General Procedure A**, and it was purified by column chromatography on silica gel (petroleum ether/ethyl acetate = 10:1), 44.3 mg, 55% yield, colorless liquid. All recorded spectroscopic data matched those previously reported in the literature[7].

^{1}H NMR (400 MHz, CDCl$_3$) δ 7.22 – 7.17 (m, 2H), 7.11 – 7.07 (m, 2H), 2.58 – 2.51 (m, 2H), 1.84 – 1.77 (m, 2H), 1.23 (s, 12H), 1.22 (s, 12H), 0.77 (t, *J* = 8.0 Hz, 1H).

^{13}C NMR (101 MHz, CDCl$_3$) δ 141.3, 131.1, 129.9, 128.2, 83.0, 37.9, 27.8, 24.9, 24.5. The signal of the α-B-carbon was not observed.

^{11}B NMR (128 MHz, CDCl$_3$) δ 34.2.

***4-(3,3-Bis(4,4,5,5-tetramethyl-1,3,2-dioxaborolan-2-yl)propyl)benzonitrile* (10)**

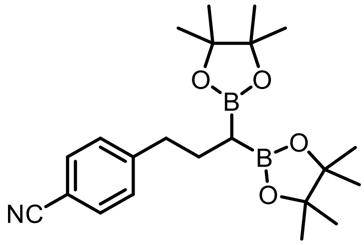

The title compound **10** was synthesized according to **General Procedure A**, and it was purified by column chromatography on silica gel (petroleum ether/ethyl acetate = 10:1), 47.8 mg, 60% yield, colorless liquid.

^{1}H NMR (400 MHz, CDCl$_3$) δ 7.55 – 7.52 (m, 2H), 7.29 – 7.25 (m, 2H), 2.67 – 2.62 (m, 2H), 1.88 – 1.81 (m, 2H), 1.24 (s, 12H), 1.23 (s, 12H), 0.78 (t, *J* = 7.8 Hz, 1H).
^{13}C NMR (101 MHz, CDCl$_3$) δ 148.6, 131.9, 129.3, 119.2, 109.3, 83.1, 38.6, 27.4, 24.8, 24.5, 10.4 (br, low intensity).
^{11}B NMR (128 MHz, CDCl$_3$) δ 34.0.
IR (ATR): ν = 2977, 2227, 1717, 1607, 1310, 1263, 1136, 1005, 967, 848 cm^{-1}.
HRMS m/z (ESI) calcd for $C_{22}H_{33}B_2NO_4$ (M + H)$^+$: 398.2668; found: 398.2658.

***Methyl 4-(3,3-bis(4,4,5,5-tetramethyl-1,3,2-dioxaborolan-2-yl)propyl)benzoate* (11)**

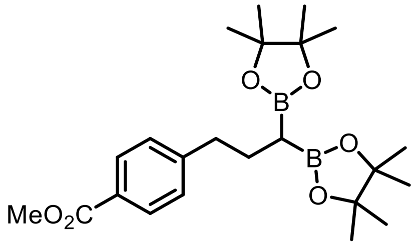

The title compound **11** was synthesized according to **General Procedure A**, and it was purified by column chromatography on silica gel (petroleum ether/ethyl acetate = 10:1), 66.9 mg, 78% yield, colorless liquid. The reaction was performed on a 1 mmol scale according to **General Procedure E**, 353 mg, 82% yield.
^{1}H NMR (400 MHz, CDCl$_3$) δ 7.94 – 7.86 (m, 2H), 7.23 – 7.18 (m, 2H), 3.86 (s, 3H), 2.65 – 2.59 (m, 2H), 1.88 – 1.79 (m, 2H), 1.21 (s, 12H), 1.20 (s, 12H), 0.77 (t, *J* = 8.0 Hz, 1H).
^{13}C NMR (101 MHz, CDCl$_3$) δ 167.1, 148.4, 129.4, 128.5, 127.4, 82.9, 51.8, 38.6, 27.5, 24.8, 24.4, 10.4 (br, low intensity).
^{11}B NMR (128 MHz, CDCl$_3$) δ 34.0.
IR (ATR): ν = 2974, 2362, 1715, 1373, 1138, 1087, 1046, 907, 728, 646 cm^{-1}.
HRMS m/z (ESI) calcd for $C_{23}H_{37}B_2O_6$ (M + H)$^+$: 431.2771; found: 431.2768.

***2,2'-(3-(2-Ethylphenyl)propane-1,1-diyl)bis(4,4,5,5-tetramethyl-1,3,2-dioxaborolane)* (12)**

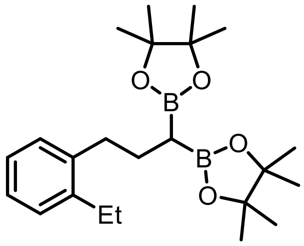

The title compound **12** was synthesized according to **General Procedure A**, and it was purified by column chromatography on silica gel (petroleum ether/ethyl acetate = 10:1), 45 mg, 56% yield, colorless liquid.
^{1}H NMR (400 MHz, CDCl$_3$) δ 7.17 – 7.08 (m, 4H), 2.70 (q, *J* = 7.6 Hz, 2H), 2.63 – 2.57 (m, 2H), 1.84 – 1.77 (m, 2H), 1.25 (s, 12H), 1.24 (s, 12H), 1.20 (d, *J* = 7.6 Hz, 3H), 0.86 (t, *J* = 7.8 Hz, 1H).
^{13}C NMR (101 MHz, CDCl$_3$) δ 141.8, 140.5, 129.4, 128.2, 125.8, 125.6, 82.9, 35.6, 27.9, 25.4, 24.9, 24.5, 15.5, 11.0 (br, low intensity).
^{11}B NMR (128 MHz, CDCl$_3$) δ 33.8.
IR (ATR): ν = 3326, 2973, 2354, 1379, 1087, 1045, 910, 879, 731, 646 cm^{-1}.
HRMS m/z (ESI) calcd for $C_{23}H_{39}B_2O_4$ (M + H)$^+$: 401.3029; found: 401.3028.

***2,2'-(3-(3-Chlorophenyl)propane-1,1-diyl)bis(4,4,5,5-tetramethyl-1,3,2-dioxaborolane)* (13)**

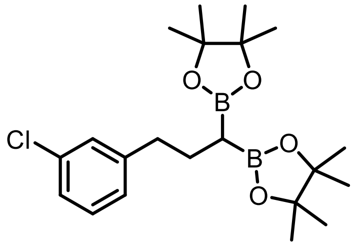

The title compound **13** was synthesized according to **General Procedure A**, and it was purified by column chromatography on silica gel (petroleum ether/ethyl acetate = 10:1), 68.8 mg, 85% yield, colorless liquid.

^{1}H NMR (400 MHz, CDCl$_3$) δ 7.18 – 7.07 (m, 3H), 7.06 – 6.97 (m, 1H), 2.55 (t, J = 8.0 Hz, 2H), 1.82 (q, J = 8.0 Hz, 2H), 1.22 (s, 12H), 1.21 (s, 12H), 0.77 (t, J = 8.0 Hz, 1H).

^{13}C NMR (101 MHz, CDCl$_3$) δ144.9, 133.8, 129.3, 128.6, 126.7, 125.6, 83.0, 38.2, 27.6, 24.8, 24.4, 10.3(br, low intensity).

^{11}B NMR (128 MHz, CDCl$_3$) δ 33.5.

IR (ATR): ν = 2973, 2262, 1455,1138, 1087, 909, 879, 730 cm^{-1}.

HRMS m/z (ESI) calcd for $C_{21}H_{34}B_2ClO_4$ (M + H)$^+$: 407.2326; found: 407.2325.

***2,2'-(3-(Thiophen-2-yl)propane-1,1-diyl)bis(4,4,5,5-tetramethyl-1,3,2-dioxaborolane)* (14)**

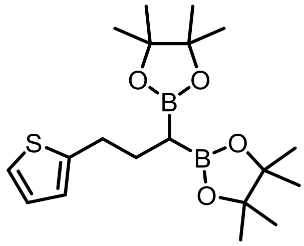

The title compound **14** was synthesized according to **General Procedure A**, and it was purified by column chromatography on silica gel (petroleum ether/ethyl acetate = 10:1), 47.2 mg, 62% yield, colorless liquid.

^{1}H NMR (400 MHz, CDCl$_3$) δ 7.06 (dd, J = 5.2, 1.2 Hz, 1H), 6.88 (dd, J = 5.2, 3.2 Hz, 1H), 6.77 (dd, J = 3.4, 1.2 Hz, 1H), 2.83 – 2.78 (m, 2H), 1.95 – 1.88 (m, 2H), 1.23 (s, 12H), 1.22 (s, 12H), 0.83 (t, J = 8.0 Hz, 1H).

^{13}C NMR (101 MHz, CDCl$_3$) δ 145.6, 126.5, 124.0, 122.6, 83.0, 32.2, 28.0, 24.8, 24.5, 10.2 (br, low intensity).

^{11}B NMR (128 MHz, CDCl$_3$) δ 33.9.

IR (ATR): ν = 2976, 1358, 1308, 1136, 967, 847, 690, 579 cm^{-1}.

HRMS m/z (ESI) calcd for $C_{19}H_{33}B_2O_4S$ (M + H)$^+$: 379.2280; found: 379.2273.

2,2'-(2-Phenylethane-1,1-diyl)bis(4,4,5,5-tetramethyl-1,3,2-dioxaborolane) (15)

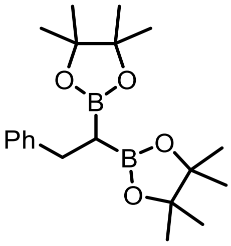

The title compound **15** was synthesized according to **General Procedure A**, and it was purified by column chromatography on silica gel (petroleum ether/ethyl acetate = 10:1), 52.9 mg, 74% yield, colorless liquid. All recorded spectroscopic data matched those previously reported in the literature[6].
^{1}H NMR (400 MHz, CDCl$_3$) δ 7.24 (d, *J* = 1.4 Hz, 3H), 7.21 (d, *J* = 8.1 Hz, 1H), 7.15 – 7.09 (m, 1H), 2.89 (d, *J* = 8.4 Hz, 2H), 1.20 – 1.18 (m, 25H).
^{13}C NMR (101 MHz, CDCl$_3$) δ 144.4, 128.3, 127.9, 125.3, 83.0, 31.2, 24.7, 24.5, 12.4 (br, low intensity).
^{11}B NMR (128 MHz, CDCl$_3$) δ 33.4.

2,2'-(4-Phenylbutane-1,1-diyl)bis(4,4,5,5-tetramethyl-1,3,2-dioxaborolane) **(16)**

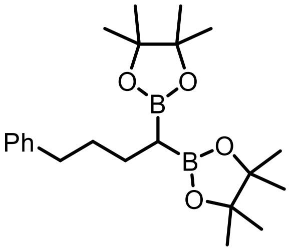

The title compound **16** was synthesized according to **General Procedure A**, and it was purified by column chromatography on silica gel (petroleum ether/ethyl acetate = 10:1), 39.9 mg, 52% yield, colorless liquid. All recorded spectroscopic data matched those previously reported in the literature[8].
^{1}H NMR (400 MHz, CDCl$_3$) δ 7.28 – 7.23 (m, 2H), 7.18 – 7.12 (m, 3H), 2.62 – 2.57 (m, 2H), 1.65 – 1.60 (m, 4H), 1.23 (s, 12H), 1.22 (s, 12H), 0.80 – 0.74 (m, 1H).
^{13}C NMR (101 MHz, CDCl$_3$) δ 143.0, 128.3, 128.1, 125.4, 82.9, 36.0, 34.3, 25.5, 24.8, 24.5, 10.5 (br, low intensity).
^{11}B NMR (128 MHz, CDCl$_3$) δ 33.9.

2,2'-(4-(Benzyloxy)butane-1,1-diyl)bis(4,4,5,5-tetramethyl-1,3,2-dioxaborolane) **(17)**

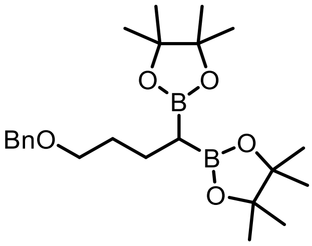

The title compound **17** was synthesized according to **General Procedure A**, and it was purified by column chromatography on silica gel (petroleum ether/ethyl acetate = 10:1), 44.9 mg, 54% yield, colorless liquid. The reaction was performed on a 1 mmol scale according to **General Procedure E**, 254 mg, 61% yield.
^{1}H NMR (400 MHz, CDCl$_3$) δ 7.34 – 7.21 (m, 5H), 4.47 (s, 2H), 3.43 (t, *J* = 6.0 Hz, 2H), 1.63 –

1.58 (m, 4H), 1.21 (s, 12H), 1.20 (s, 12H), 0.75 – 0.70 (m, 1H).
^{13}C NMR (101 MHz, CDCl$_3$) δ 138.7, 128.2, 127.6, 127.3, 82.9, 72.6, 70.5, 32.2, 24.8, 24.5, 22.1, 10.4 (br, low intensity).
^{11}B NMR (128 MHz, CDCl$_3$) δ 33.6.
IR (ATR): ν = 3340, 2974, 2356, 1380, 1087, 1046, 907, 879, 729, 646 cm^{-1}.
HRMS m/z (ESI) calcd for $C_{23}H_{39}B_2O_5$ (M + H)$^+$: 417.2978; found: 417.2965.

(5,5-Bis(4,4,5,5-tetramethyl-1,3,2-dioxaborolan-2-yl)pent-1-yn-1-yl)trimethylsilane **(18)**

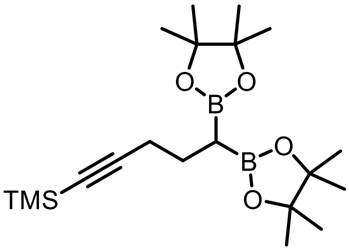

The title compound **18** was synthesized according to **General Procedure A**, and it was purified by column chromatography on silica gel (petroleum ether/ethyl acetate = 10:1), 50.1 mg, 64% yield, colorless liquid.
^{1}H NMR (400 MHz, CDCl$_3$) δ 2.25 – 2.19 (m, 2H), 1.77 (q, J = 7.8 Hz, 2H), 1.22 (s, 12H), 1.21 (s, 12H), 0.88 (t, J = 8.0 Hz, 1H), 0.12 (s, 9H).
^{13}C NMR (101 MHz, CDCl$_3$) δ 107.8, 84.1, 83.0, 24.8, 24.8, 24.5, 21.9, 0.2. The signal of the α-B-carbon was not observed.
^{11}B NMR (128 MHz, CDCl$_3$) δ 33.1.
IR (ATR): ν = 3333, 2973, 1379, 1138, 1087, 1045, 879, 731, 645 cm^{-1}.
HRMS m/z (ESI) calcd for $C_{20}H_{39}B_2O_4Si$ (M + H)$^+$: 393.2798; found: 393.2790.

2,2'-(5-Phenylpent-4-yne-1,1-diyl)bis(4,4,5,5-tetramethyl-1,3,2-dioxaborolane) **(19)**

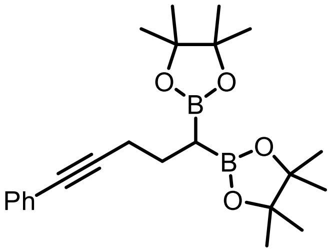

The title compound **19** was synthesized according to **General Procedure A**, and it was purified by column chromatography on silica gel (petroleum ether/ethyl acetate = 10:1), 56.2 mg, 71% yield, colorless liquid.
^{1}H NMR (400 MHz, CDCl$_3$) δ 7.39 – 7.33 (m, 2H), 7.27 – 7.20 (m, 3H), 2.41 (t, J = 7.6 Hz, 2H), 1.89 – 1.83 (m, 2H), 1.22 (s, 12H), 1.21 (s, 12H), 0.98 (t, J = 8.0 Hz, 1H).
^{13}C NMR (101 MHz, CDCl$_3$) δ 131.5, 128.0, 127.3, 124.2, 90.5, 83.0, 80.7, 24.9, 24.8, 24.4, 21.4, 10.0 (br, low intensity).
^{11}B NMR (128 MHz, CDCl$_3$) δ 34.0.
IR (ATR): ν = 2976, 1598, 1360, 1308, 1265, 1136, 966, 847, 755, 691 cm^{-1}.
HRMS m/z (ESI) calcd for $C_{23}H_{35}B_2O_4$ (M + H)$^+$: 397.2716; found: 397.2712.

2,2'-(Undec-10-ene-1,1-diyl)bis(4,4,5,5-tetramethyl-1,3,2-dioxaborolane) **(20)**

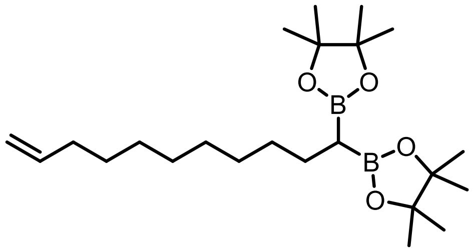

The title compound **20** was synthesized according to **General Procedure A**, and it was purified by column chromatography on silica gel (petroleum ether/ethyl acetate = 20:1), 52.3 mg, 64% yield, colorless liquid. All recorded spectroscopic data matched those previously reported in the literature[8].

^{1}H NMR (400 MHz, CDCl$_3$) δ 5.85 – 5.74 (m, 1H), 5.01 – 4.93 (m, 1H), 4.92 – 4.87 (m, 1H), 2.04 – 1.99 (m, 2H), 1.51 (t, *J* = 7.5 Hz, 2H), 1.37 – 1.33 (m, 2H), 1.24 (s, 10H), 1.22 (s, 12H), 1.21 (s, 12H), 0.70 (t, *J* = 7.9 Hz, 1H).

^{13}C NMR (101 MHz, CDCl$_3$) δ 139.3, 114.0, 82.8, 33.8, 32.5, 29.5, 29.4, 29.4, 29.1, 28.9, 25.6, 24.8, 24.5, 10.7 (br, low intensity).

^{11}B NMR (128 MHz, CDCl$_3$) δ 33.4.

2,2'-(Cyclohex-3-en-1-ylmethylene)bis(4,4,5,5-tetramethyl-1,3,2-dioxaborolane) **(21)**

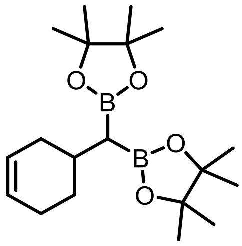

The title compound **21** was synthesized according to **General Procedure A**, and it was purified by column chromatography on silica gel (petroleum ether/ethyl acetate = 10:1), 43.8 mg, 63% yield, colorless liquid.

^{1}H NMR (500 MHz, CDCl$_3$) δ 5.63 – 5.55 (m, 2H), 2.25 – 2.18 (m, 1H), 2.06 – 1.93 (m, 3H), 1.79 – 1.71 (m, 1H), 1.69 – 1.60 (m, 1H), 1.30 – 1.25 (m, 1H), 1.24 – 1.20 (m, 24H), 0.71 (d, *J* = 10.0 Hz, 1H).

^{13}C NMR (126 MHz, CDCl$_3$) δ 126.9, 126.5, 82.8, 34.1, 31.7, 31.4, 25.7, 24.9, 24.5, 18.9 (br, low intensity).

^{11}B NMR (160 MHz, CDCl$_3$) δ 33.7.

IR (ATR): ν = 2977, 1468, 1309, 1264, 1211, 969, 848, 730, 646 cm^{-1}.

HRMS m/z (ESI) calcd for $C_{19}H_{35}B_2O_4$ $(M + H)^+$: 349.2716; found: 349.2713.

2,2'-(2-Phenylpropane-1,1-diyl)bis(4,4,5,5-tetramethyl-1,3,2-dioxaborolane) **(22)**

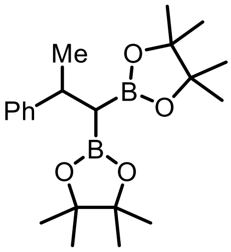

The title compound **22** was synthesized according to **General Procedure A**, and it was purified by column chromatography on silica gel (petroleum ether/ethyl acetate = 20:1), 35.7 mg, 48% yield, colorless liquid. All recorded spectroscopic data matched those previously reported in the literature[6].

^{1}H NMR (400 MHz, CDCl$_3$) δ 7.25 – 7.17 (m, 4H), 7.13 – 7.05 (m, 1H), 3.22 – 3.13 (m, 1H), 1.28 – 1.24 (m, 16H), 0.95 (s, 6H), 0.90 (s, 6H).

^{13}C NMR (101 MHz, CDCl$_3$) δ 149.5, 127.9, 127.0, 125.5, 83.1, 82.8, 37.8, 26.1, 24.9, 24.4, 24.4, 24.2. The signal of the α-B-carbon was not observed.

^{11}B NMR (128 MHz, CDCl$_3$) δ 34.0.

***2,2'-(2-(p-tolyl)Propane-1,1-diyl)bis(4,4,5,5-tetramethyl-1,3,2-dioxaborolane)* (23)**

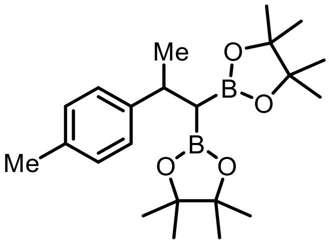

The title compound **23** was synthesized according to **General Procedure A**, and it was purified by column chromatography on silica gel (petroleum ether/ethyl acetate = 10:1), 45 mg, 58% yield, colorless liquid.

^{1}H NMR (500 MHz, CDCl$_3$) δ 7.12 (d, J = 8.0 Hz, 2H), 7.02 (d, J = 8.0 Hz, 2H), 3.18 – 3.09 (m, 1H), 2.27 (s, 3H), 1.26 (s, 6H), 1.26 – 1.23 (m, 9H), 1.21 (d, J = 12.0 Hz, 1H), 0.96 (s, 6H), 0.92 (s, 6H).

^{13}C NMR (126 MHz, CDCl$_3$) δ 146.5, 134.8, 128.6, 126.9, 83.1, 82.8, 37.4, 26.1, 24.9, 24.4, 24.4, 24.2, 20.9. The signal of the α-B-carbon was not observed.

^{11}B NMR (128 MHz, CDCl$_3$) δ 33.8.

IR (ATR): ν = 3333, 2973, 2363, 1379, 1087, 1045, 908, 879, 729, 646 cm^{-1}.

HRMS m/z (ESI) calcd for $C_{22}H_{36}B_2O_4$ (M + H)$^+$: 387.2872; found: 387.2866.

***2,2'-(3-(Benzo[d][1,3]dioxol-5-yl)-2-methylpropane-1,1-diyl)bis(4,4,5,5-tetramethyl-1,3,2-dioxaborolane)* (24)**

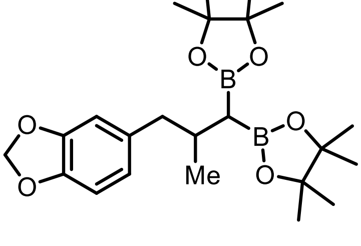

The title compound **24** was synthesized according to **General Procedure A**, and it was purified by column chromatography on silica gel (petroleum ether/ethyl acetate = 10:1), 60.3 mg, 75% yield, colorless liquid. All recorded spectroscopic data matched those previously reported in the literature[6].

^{1}H NMR (400 MHz, CDCl$_3$) δ 6.71 – 6.68 (m, 2H), 6.62 – 6.59 (m, 1H), 5.89 (s, 2H), 2.77 (d, J = 9.1 Hz, 1H), 2.20 – 2.09 (m, 2H), 1.25 (d, J = 4.0 Hz, 12H), 1.23 (d, J = 4.4Hz, 12H), 0.85 (d, J = 6.2 Hz, 3H), 0.76 (d, J = 9.3 Hz, 1H).

^{13}C NMR (101 MHz, CDCl$_3$) δ 147.2, 145.3, 135.8, 122.1, 109.7, 107.7, 100.6, 83.0, 82.9, 45.6, 33.4, 25.0, 24.9, 24.5, 24.5, 21.0. The signal of the α-B-carbon was not observed.

^{11}B NMR (128 MHz, CDCl$_3$) δ 33.2.

***2,2'-(3-(4-Isopropylphenyl)-2-methylpropane-1,1-diyl)bis(4,4,5,5-tetramethyl-1,3,2-dioxaborolane)* (25)**

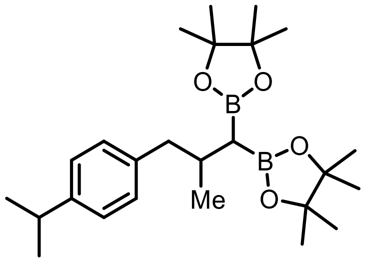

The title compound **25** was synthesized according to **General Procedure A**, and it was purified by column chromatography on silica gel (petroleum ether/ethyl acetate = 10:1), 46.7 mg, 54% yield, colorless liquid. All recorded spectroscopic data matched those previously reported in the literature[8].

^{1}H NMR (400 MHz, CDCl$_3$) δ 7.11 (s, 4H), 2.89 – 2.80 (m, 2H), 2.27 – 2.17 (m, 2H), 1.26 – 1.22 (m, 30H), 0.86 (d, J = 6.1 Hz, 3H), 0.79 (d, J = 9.2 Hz, 1H).

^{13}C NMR (101 MHz, CDCl$_3$) δ 145.9, 139.0, 129.3, 125.9, 82.9, 82.9, 45.4, 33.6, 33.2, 25.0, 24.9, 24.5, 24.5, 24.1, 24.0, 21.2, 19.5 (br, low intensity).

^{11}B NMR (128 MHz, CDCl$_3$) δ 33.1.

***2,2'-(3-(4-(Tert-butyl)phenyl)-2-methylpropane-1,1-diyl)bis(4,4,5,5-tetramethyl-1,3,2-dioxaborolane)* (26)**

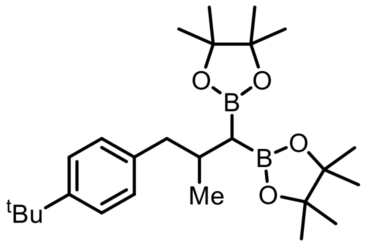

The title compound **26** was synthesized according to **General Procedure A**, and it was purified by column chromatography on silica gel (petroleum ether/ethyl acetate = 10:1), 59.1 mg, 67% yield, colorless liquid. All recorded spectroscopic data matched those previously reported in the literature[6]. The reaction was performed on a 1 mmol scale according to **General Procedure E**, 310 mg, 70% yield.

^{1}H NMR (400 MHz, CDCl$_3$) δ 7.27 (d, J = 8.2 Hz, 2H), 7.14 – 7.08 (m, 2H), 2.87 – 2.77 (m, 1H), 2.29 – 2.17 (m, 2H), 1.30 (s, 9H), 1.24 (t, J = 5.2 Hz, 24H), 0.87 (d, J = 6.2 Hz, 3H), 0.79 (d, J = 9.2 Hz, 1H).

^{13}C NMR (101 MHz, CDCl$_3$) δ 148.1, 138.6, 129.0, 124.8, 82.9, 82.9, 45.3, 34.3, 33.1, 31.4, 25.0,

24.9, 24.5, 24.5, 21.3. The signal of the α-B-carbon was not observed.
^{11}B NMR (128 MHz, $CDCl_3$) δ 32.9.

2,2'-((2,3-Dihydro-1H-inden-2-yl)methylene)bis(4,4,5,5-tetramethyl-1,3,2-dioxaborolane) **(27)**

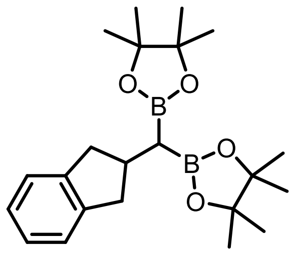

The title compound **27** was synthesized according to **General Procedure A**, and it was purified by column chromatography on silica gel (petroleum ether/ethyl acetate = 10:1), 50.2 mg, 65% yield, colorless liquid.
^{1}H NMR (400 MHz, $CDCl_3$) δ 7.18 – 7.11 (m, 2H), 7.11 – 7.03 (m, 2H), 3.15 (dd, J = 15.2, 7.6 Hz, 2H), 2.86 – 2.73 (m, 1H), 2.54 (dd, J = 15.2, 9.2 Hz, 2H), 1.26 (s, 12H), 1.25 (s, 12H), 0.98 (d, J = 10.4 Hz, 1H).
^{13}C NMR (101 MHz, $CDCl_3$) δ 144.0, 125.7, 124.1, 83.0, 41.5, 38.0, 24.9, 24.5. The signal of the α-B-carbon was not observed.
^{11}B NMR (128 MHz, $CDCl_3$) δ 33.9.
IR (ATR): ν = 2980, 2252, 1322, 1138, 968, 903, 849, 723, 649 cm^{-1}.
HRMS m/z (ESI) calcd for $C_{22}H_{35}B_2O_4$ $(M + H)^+$: 385.2716; found: 385.2712.

2,2'-(((1r,4r)-4-(4-Chlorophenyl)cyclohexyl)methylene)bis(4,4,5,5-tetramethyl-1,3,2-dioxaborolane) **(28)**

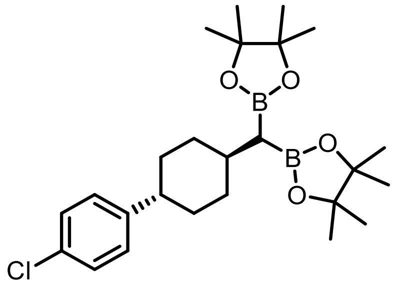

The title compound **28** was synthesized according to **General Procedure A**, and it was purified by column chromatography on silica gel (petroleum ether/ethyl acetate = 10:1), 42.7 mg, 46% yield, colorless liquid.
^{1}H NMR (400 MHz, $CDCl_3$) δ 7.22 (d, J = 8.4 Hz, 2H), 7.11 (d, J = 8.4 Hz, 2H), 2.39 (tt, J = 12.2, 3.6 Hz, 1H), 1.97 – 1.88 (m, 2H), 1.84 – 1.73 (m, 3H), 1.53 – 1.39 (m, 2H), 1.24 (s, 12H), 1.23 (s, 12H), 1.12 (td, J = 12.4, 3.2 Hz, 3H), 0.66 (d, J = 10.0 Hz, 1H).
^{13}C NMR (101 MHz, $CDCl_3$) δ 146.4, 131.2, 128.2, 128.2, 82.9, 43.6, 35.8, 35.4, 34.6, 24.9, 24.6. The signal of the α-B-carbon was not observed.
^{11}B NMR (128 MHz, $CDCl_3$) δ 33.1.
IR (ATR): ν = 3354, 2974, 2247, 1380, 1137, 1087, 1046, 907, 879, 729 cm^{-1}.
HRMS m/z (ESI) calcd for $C_{25}H_{40}B_2ClO_4$ $(M + H)^+$: 461.2796; found: 461.2794.

***2,2'-(Tetrahydro-2H-pyran-4,4-diyl)bis(4,4,5,5-tetramethyl-1,3,2-dioxaborolane)* (29)**

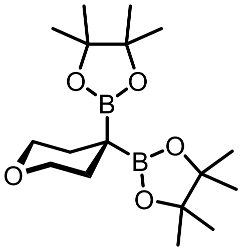

The title compound **29** was synthesized according to **General Procedure A**, and it was purified by column chromatography on silica gel (petroleum ether/ethyl acetate = 20:1), 47.3 mg, 70% yield, colorless liquid.

^{1}H NMR (500 MHz, CDCl$_3$) δ 3.63 – 3.59 (m, 4H), 1.78 – 1.75 (m, 4H), 1.22 (s, 24H).

^{13}C NMR (126 MHz, CDCl$_3$) δ 83.2, 68.3, 29.2, 24.6. The signal of the α-B-carbon was not observed.

^{11}B NMR (160 MHz, CDCl$_3$) δ 34.0.

IR (ATR): ν = 3361, 2974, 2357, 1380, 1046, 906, 726, 647 cm^{-1}.

HRMS m/z (ESI) calcd for $C_{17}H_{33}B_2O_5$ (M + H)$^+$: 339.2509; found: 339.2504.

***Bis(4,4,5,5-tetramethyl-1,3,2-dioxaborolan-2-yl)methane* (30)**

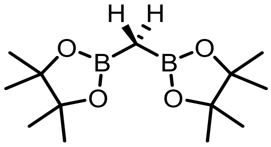

The title compound **30** was synthesized according to **General Procedure A**, and it was purified by column chromatography on silica gel (petroleum ether/ethyl acetate = 20:1), 37.8 mg, 71% yield, white solid. All recorded spectroscopic data matched those previously reported in the literature[8]. The reaction was performed on a 10 mmol scale according to **General Procedure E**, 1.74 g, 65% yield.

^{1}H NMR (400 MHz, CDCl$_3$) δ 1.21 (s, 24H), 0.33 (s, 2H).

^{13}C NMR (101 MHz, CDCl$_3$) δ 83.0, 24.7. The signal of the α-B-carbon was not observed.

^{11}B NMR (128 MHz, CDCl$_3$) δ 33.5.

***Bis(4,4,5,5-tetramethyl-1,3,2-dioxaborolan-2-yl)methane-d_2* (31)**

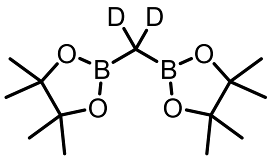

The title compound **31** was synthesized according to **General Procedure A**, and it was purified by column chromatography on silica gel (petroleum ether/ethyl acetate = 10:1), 32.4 mg, 60% yield, colorless liquid. The reaction was performed on a 1 mmol scale according to **General Procedure E**, 151 mg, 56% yield.

^{1}H NMR (400 MHz, CDCl$_3$) δ 1.22 (s, 24H).

^{13}C NMR (101 MHz, CDCl$_3$) δ 83.0, 24.7. The signal of the α-B-carbon was not observed.

^{11}B NMR (128 MHz, CDCl$_3$) δ 33.5.

IR (ATR): ν = 3362, 2975, 2250, 1139, 1087, 1047, 905, 725, 647 cm^{-1}.
HRMS m/z (ESI) calcd for $C_{13}H_{24}D_2B_2O_4$ $(M + H)^+$: 271.2216; found: 271.2209.

2,2'-(2-(2-Chlorophenyl)-2-(4-chlorophenyl)ethane-1,1-diyl)bis(4,4,5,5-tetramethyl-1,3,2-dioxaborolane) **(32)**

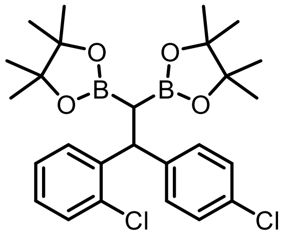

The title compound **32** was synthesized according to **General Procedure A**, and it was purified by column chromatography on silica gel (petroleum ether/ethyl acetate = 10:1), 52.2 mg, 52% yield, colorless liquid.
^{1}H NMR (400 MHz, $CDCl_3$) δ 7.41 (dd, *J* = 8.0, 1.6 Hz, 1H), 7.27 – 7.21 (m, 2H), 7.20 – 7.17 (m, 1H), 7.16 – 7.12 (m, 1H), 7.11 – 7.06 (m, 2H), 7.01 – 6.95 (m, 1H), 4.84 (d, *J* = 13.2 Hz, 1H), 1.82 (d, *J* = 13.2 Hz, 1H), 0.99 (d, *J* = 7.2 Hz, 12H), 0.96 (s, 6H), 0.84 (s, 6H).
^{13}C NMR (101 MHz, $CDCl_3$) δ 144.0, 143.6, 134.1, 131.5, 129.5, 129.5, 128.0, 128.0, 127.1, 126.8, 83.3, 83.2, 43.4, 24.6, 24.5, 24.2, 23.9. The signal of the α-B-carbon was not observed.
^{11}B NMR (128 MHz, $CDCl_3$) δ 33.9.
IR (ATR): ν = 2976, 2362, 1372, 1088, 1046, 879, 730, 647 cm^{-1}.
HRMS m/z (ESI) calcd for $C_{26}H_{35}B_2Cl_2O_4$ $(M + H)^+$: 503.2093; found: 503.2079.

2-(3,3-Bis(4,4,5,5-tetramethyl-1,3,2-dioxaborolan-2-yl)propyl)-4,5-diphenyloxazole **(33)**

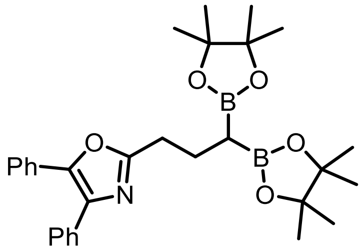

The title compound **33** was synthesized according to **General Procedure A**, and it was purified by column chromatography on silica gel (petroleum ether/ethyl acetate = 3:1), 63.3 mg, 61% yield, colorless liquid.
^{1}H NMR (400 MHz, $CDCl_3$) δ 7.66 – 7.61 (m, 2H), 7.61 – 7.56 (m, 2H), 7.38 – 7.29 (m, 6H), 2.88 (d, *J* = 8.2 Hz, 2H), 2.09 (q, *J* = 8.0 Hz, 2H), 1.24 (s, 12H), 1.24 (s, 12H), 0.88 (t, *J* = 8.0 Hz, 1H).
^{13}C NMR (101 MHz, $CDCl_3$) δ 164.0, 145.0, 134.8, 132.6, 129.2, 128.51, 128.46, 128.2, 128.0, 127.9, 126.5, 83.2, 30.1, 24.9, 24.5, 23.5. The signal of the α-B-carbon was not observed.
^{11}B NMR (128 MHz, $CDCl_3$) δ 33.6.
IR (ATR): ν = 3353, 2974, 1380, 1087, 1046, 906, 879, 727, 647 cm^{-1}.
HRMS m/z (ESI) calcd for $C_{29}H_{38}B_2NO_5$ $(M + H)^+$: 502.2931; found: 502.2946.

2,2'-((9Z,12E)-octadeca-9,12-diene-1,1-diyl)bis(4,4,5,5-tetramethyl-1,3,2-dioxaborolane) **(34)**

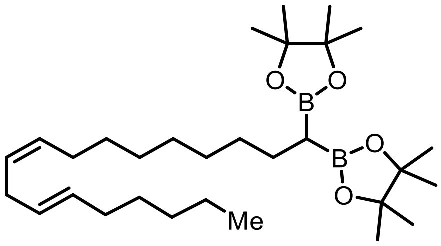

The title compound **34** was synthesized according to **General Procedure A**, and it was purified by column chromatography on silica gel (petroleum ether/ethyl acetate = 10:1), 46.2 mg, 46% yield, colorless liquid.

^{1}H NMR (400 MHz, CDCl$_3$) δ 5.42 – 5.26 (m, 4H), 2.76 (t, *J* = 6.4 Hz, 2H), 2.04 (q, *J* = 6.8 Hz, 4H), 1.52 (d, *J* = 7.2 Hz, 2H), 1.37 – 1.25 (m, 16H), 1.22 (s, 12H), 1.21 (s, 12H), 0.88 (t, *J* = 6.8 Hz, 3H), 0.70 (t, *J* = 8.0 Hz, 1H).

^{13}C NMR (101 MHz, CDCl$_3$) δ 130.23, 130.15, 128.0, 127.9, 82.8, 32.6, 31.5, 29.7, 29.6, 29.4, 29.3, 29.3, 27.3, 27.2, 25.7, 25.6, 24.8, 24.5, 22.6, 14.1. The signal of the α-B-carbon was not observed.

^{11}B NMR (128 MHz, CDCl$_3$) δ 33.4.

IR (ATR): ν = 3359, 2974, 2248, 1087, 1046, 907, 879, 727, 647 cm^{-1}.

HRMS m/z (ESI) calcd for $C_{30}H_{57}B_2O_4$ (M + H)$^+$: 503.4437; found: 503.4438.

***4-(4,4-Bis(4,4,5,5-tetramethyl-1,3,2-dioxaborolan-2-yl)butyl)-N,N-bis(2-chloroethyl)aniline* (35)**

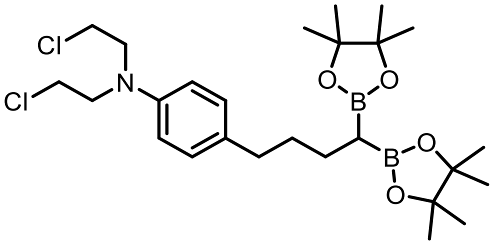

The title compound **35** was synthesized according to **General Procedure A**, and it was purified by column chromatography on silica gel (petroleum ether/ethyl acetate = 5:1), 50.7 mg, 48% yield, colorless liquid.

^{1}H NMR (400 MHz, CDCl$_3$) δ 7.08 (d, *J* = 8.4 Hz, 2H), 6.68 (d, *J* = 8.0 Hz, 2H), 3.73 – 3.67 (m, 4H), 3.64 – 3.60 (m, 4H), 2.51 (t, *J* = 7.2 Hz, 2H), 1.63 – 1.56 (m, 4H), 1.23 (s, 12H), 1.22 (s, 12H), 0.75 (t, *J* = 7.0 Hz, 1H).

^{13}C NMR (101 MHz, CDCl$_3$) δ 144.8, 129.7, 129.6, 112.8, 82.9, 54.0, 40.2, 34.9, 34.5, 25.5, 24.9, 24.5. The signal of the α-B-carbon was not observed.

^{11}B NMR (128 MHz, CDCl$_3$) δ 34.6.

IR (ATR): ν = 2979, 2252, 1518, 1138, 1047, 903, 723, 649 cm^{-1}.

HRMS m/z (ESI) calcd for $C_{26}H_{44}B_2Cl_2NO_4$ (M + H)$^+$: 526.2828; found: 526.2819.

***(R)-2,5,7,8-tetramethyl-2-((4R,8R)-4,8,12-trimethyltridecyl)chroman-6-yl 4,4-bis(4,4,5,5-tetramethyl-1,3,2-dioxaborolan-2-yl)butanoate* (36)**

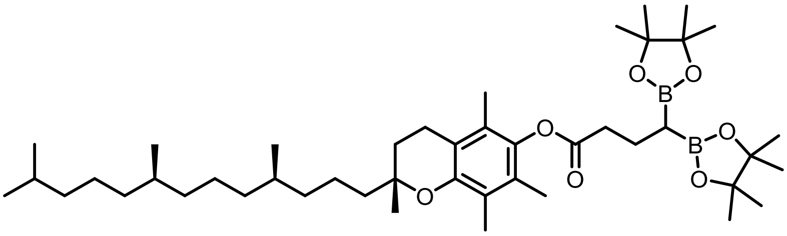

The title compound **36** was synthesized according to **General Procedure A**, and it was purified by column chromatography on silica gel (petroleum ether/ethyl acetate = 10:1), 75.7 mg, 50% yield, colorless liquid.

^{1}H NMR (400 MHz, CDCl$_3$) δ 2.66 – 2.60 (m, 2H), 2.58 (t, *J* = 7.0 Hz, 2H), 2.08 (s, 3H), 2.06 – 2.01 (m, 2H), 2.00 (s, 3H), 1.96 (s, 3H), 1.83 – 1.69 (m, 2H), 1.58 – 1.47 (m, 3H), 1.44 – 1.34 (m, 4H), 1.28 – 1.19 (m, 35H), 1.17 – 1.12 (m, 3H), 1.11 – 1.02 (m, 4H), 0.88 – 0.83 (m, 12H).

^{13}C NMR (101 MHz, CDCl$_3$) δ 172.1, 149.2, 140.5, 126.7, 124.9, 122.8, 117.2, 83.1, 74.9, 39.3, 37.4, 37.2, 36.0, 32.7, 32.6, 31.1, 27.9, 24.8, 24.7, 24.4, 24.4, 22.7, 22.6, 21.0, 21.0, 20.5, 19.7, 19.6, 13.0, 12.1, 11.7, 10.1 (br, low intensity).

^{11}B NMR (128 MHz, CDCl$_3$) δ 32.8.

IR (ATR): ν = 3379, 2974, 2249, 1734, 1373, 1086, 1047, 905, 727, 647 cm^{-1}.

HRMS m/z (ESI) calcd for $C_{45}H_{79}B_2O_7$ (M + H)$^+$: 753.6006;found: 753.6005.

***2-(1-Chloro-2-phenylcyclopropyl)-4,4,5,5-tetramethyl-1,3,2-dioxaborolane* (37)**

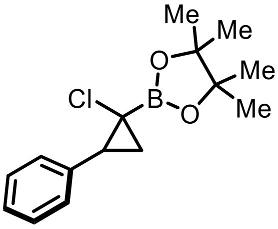

The title compound **37** was synthesized according to **General Procedure B**, and it was purified by column chromatography on silica gel (petroleum ether/ethyl acetate = 20:1), 28.2 mg, 51% yield, colorless liquid. The reaction was performed on a 1 mmol scale according to **General Procedure E**, 150 mg, 54% yield.

^{1}H NMR (400 MHz, CDCl$_3$) δ 7.35 – 7.29 (m, 2H), 7.28 – 7.26 (m, 2H), 7.26 – 7.24 (m, 1H), 2.58 (dd, *J* = 9.6, 8.0 Hz, 1H), 1.66 (dd, *J* = 9.6, 6.0 Hz, 1H), 1.50 (dd, *J* = 7.6, 6.0 Hz, 1H), 1.31 (s, 6H), 1.30 (s, 6H).

^{13}C NMR (101 MHz, CDCl$_3$) δ 136.0, 129.6, 127.8, 126.8, 84.8, 29.2, 24.7, 20.3. The signal of the α-B-carbon was not observed.

^{11}B NMR (128 MHz, CDCl$_3$) δ 32.4.

IR (ATR): ν = 3358, 2974, 2247, 1380, 1087, 1046, 907, 879, 728, 647 cm^{-1}.

HRMS m/z (ESI) calcd for $C_{15}H_{21}BClO_2$ (M + H)$^+$: 279.1318; found: 279.1316.

***2-(1-Chloro-2,2-diphenylcyclopropyl)-4,4,5,5-tetramethyl-1,3,2-dioxaborolane* (38)**

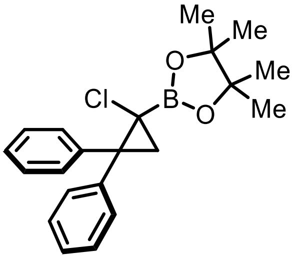

The title compound **38** was synthesized according to **General Procedure B**, and it was purified by column chromatography on silica gel (petroleum ether/ethyl acetate = 20:1), 37.1 mg, 52% yield, colorless liquid.

^{1}H NMR (400 MHz, $CDCl_3$) δ 7.57 – 7.51 (m, 2H), 7.49 – 7.43 (m, 2H), 7.32 – 7.27 (m, 2H), 7.25 – 7.18 (m, 3H), 7.16 – 7.10 (m, 1H), 2.23 (d, J = 5.6 Hz, 1H), 1.78 (d, J = 5.8 Hz, 1H), 1.15 (s, 6H), 0.93 (s, 6H).

^{13}C NMR (101 MHz, $CDCl_3$) δ 142.7, 141.5, 130.3, 129.3, 128.4, 128.1, 126.9, 126.8, 84.4, 42.7, 26.2, 24.7, 24.4. The signal of the α-B-carbon was not observed.

^{11}B NMR (128 MHz, $CDCl_3$) δ 30.9.

IR (ATR): ν = 2974, 2263, 1382, 1087, 1046, 906, 726, 647 cm^{-1}.

HRMS m/z (ESI) calcd for $C_{21}H_{25}BClO_2$ $(M + H)^+$: 355.1631; found: 355.1628.

2-(7-Chlorobicyclo[4.1.0]heptan-7-yl)-4,4,5,5-tetramethyl-1,3,2-dioxaborolane **(39)**

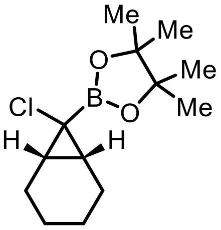

The title compound **39** was synthesized according to **General Procedure B**, and it was purified by column chromatography on silica gel (petroleum ether/ethyl acetate = 20:1), 37.2 mg, 73% yield, colorless liquid.

^{1}H NMR (500 MHz, $CDCl_3$) δ 1.96 – 1.88 (m, 2H), 1.63 – 1.57 (m, 2H), 1.38 – 1.31 (m, 4H), 1.25 – 1.21 (m, 14H).

^{13}C NMR (126 MHz, $CDCl_3$) δ 84.3, 24.6, 21.5, 19.1, 18.8. The signal of the α-B-carbon was not observed.

^{11}B NMR (160 MHz, $CDCl_3$) δ 37.6.

IR (ATR): ν = 2983, 2252, 1365, 1142, 903, 851, 723, 649 cm^{-1}.

HRMS m/z (ESI) calcd for $C_{13}H_{23}BClO_2$ $(M + H)^+$: 257.1474; found: 257.1467.

7-Chlorobicyclo[4.1.0]heptan-7-yl)boronic acid **(39a)**

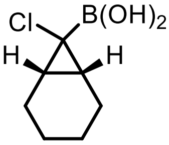

The title compound **39a** was synthesized was synthesized from **39** using the literature procedure[36], 22.6 mg, 65% yield, white solid. **^{1}H NMR (400 MHz, $CDCl_3$)** δ 4.47 (s, 1H), 2.03 – 1.87 (m, 2H),

1.78 – 1.56 (m, 2H), 1.56 – 1.41 (m, 2H), 1.42 – 1.31 (m, 2H), 1.31 – 1.20 (m, 2H). **^{13}C NMR (126 MHz, CDCl$_3$)** δ 21.4, 21.0, 18.8. The signal of the α-B-carbon was not observed. **^{11}B NMR (160 MHz, CDCl$_3$)** δ 31.1**. HRMS m/z (ESI)** calcd for $C_7H_{11}BClO_2$ (M – H)$^+$: 173.0546; found: 173.0538.

***2-(8-Chlorobicyclo[5.1.0]octan-8-yl)-4,4,5,5-tetramethyl-1,3,2-dioxaborolane* (40)**

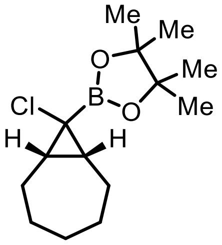

The title compound **40** was synthesized according to **General Procedure B**, and it was purified by column chromatography on silica gel (petroleum ether/ethyl acetate = 20:1), 22.1 mg, 41% yield, colorless liquid.

^{1}H NMR (400 MHz, CDCl$_3$) δ 1.98 – 1.85 (m, 4H), 1.84 – 1.78 (m, 2H), 1.44 – 1.38 (m, 4H), 1.36 – 1.31 (m, 2H), 1.26 (s, 12H).

^{13}C NMR (101 MHz, CDCl$_3$) δ 84.3, 32.4, 28.2, 27.3, 24.8, 24.7. The signal of the α-B-carbon was not observed.

^{11}B NMR (128 MHz, CDCl$_3$) δ 32.5.

IR (ATR): ν = 2979, 1419, 1361, 1324, 1128, 977, 909, 852, 723, 678 cm^{-1}.

HRMS m/z (ESI) calcd for $C_{14}H_{25}BClO_2$ (M + H)$^+$: 271.1631; found: 271.1633.

***2-(9-Chlorobicyclo[6.1.0]nonan-9-yl)-4,4,5,5-tetramethyl-1,3,2-dioxaborolane* (41)**

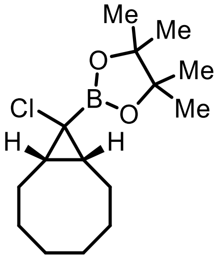

The title compound **41** was synthesized according to **General Procedure B**, and it was purified by column chromatography on silica gel (petroleum ether/ethyl acetate = 20:1), 33.4 mg, 59% yield, colorless liquid.

^{1}H NMR (400 MHz, CDCl$_3$) δ 1.85 – 1.79 (m, 2H), 1.69 – 1.62 (m, 4H), 1.47 – 1.35 (m, 6H), 1.26 (s, 12H), 1.15 – 1.08 (m, 2H).

^{13}C NMR (101 MHz, CDCl$_3$) δ 84.3, 28.7, 26.6, 25.9, 24.7, 22.4. The signal of the α-B-carbon was not observed.

^{11}B NMR (128 MHz, CDCl$_3$) δ 33.1.

IR (ATR): ν = 2973, 2262, 1456, 1087, 1046, 908, 879, 729, 646 cm^{-1}.

HRMS m/z (ESI) calcd for $C_{15}H_{27}BClO_2$ (M + H)$^+$: 285.1787; found: 285.1797.

***2-(1-Chloro-2,2-dimethyl-3-(m-tolyl)propyl)-4,4,5,5-tetramethyl-1,3,2-dioxaborolane* (42)**

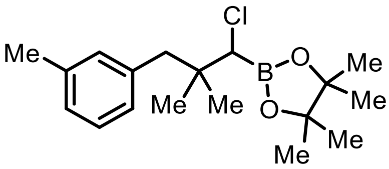

The title compound **42** was synthesized according to **General Procedure B**, and it was purified by column chromatography on silica gel (petroleum ether/ethyl acetate = 10:1), 34.2 mg, 53% yield, colorless liquid.

^{1}H NMR (400 MHz, CDCl$_3$) δ 7.18 – 7.11 (m, 1H), 7.04 – 6.99 (m, 3H), 3.27 (s, 1H), 2.85 (d, J = 13.2 Hz, 1H), 2.54 – 2.46 (m, 2H), 2.33 (s, 3H), 1.28 (s, 12H), 1.11 (s, 3H), 0.95 (s, 3H).

^{13}C NMR (101 MHz, CDCl$_3$) δ 138.1, 137.3, 131.4, 127.7, 127.7, 126.8, 84.1, 50.7, 45.4, 29.2, 24.6, 24.5, 21.4. The signal of the α-B-carbon was not observed.

^{11}B NMR (128 MHz, CDCl$_3$) δ 31.5.

IR (ATR): ν = 3329, 2973, 2360, 1379, 1087, 1045, 908, 879, 729, 646 cm^{-1}.

HRMS m/z (ESI) calcd for $C_{18}H_{29}BClO_2$ $(M + H)^+$: 323.1944; found: 323.1941.

***2-(1-Chloro-5-(2,5-dimethylphenoxy)-2,2-dimethylpentyl)-4,4,5,5-tetramethyl-1,3,2-dioxaborolane* (43)**

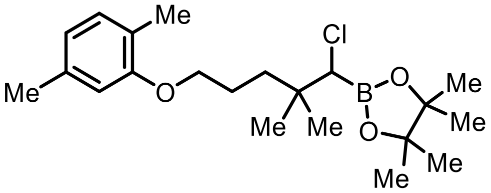

The title compound **43** was synthesized according to **General Procedure B**, and it was purified by column chromatography on silica gel (petroleum ether/ethyl acetate = 10:1), 36.5 mg, 48% yield, colorless liquid.

^{1}H NMR (400 MHz, CDCl$_3$) δ 7.00 (d, J = 7.2 Hz, 1H), 6.65 (d, J = 7.0 Hz, 1H), 6.62 (s, 1H), 3.95 – 3.89 (m, 2H), 3.34 (s, 1H), 2.31 (s, 3H), 2.18 (s, 3H), 1.82 – 1.72 (m, 2H), 1.68 – 1.57 (m, 2H), 1.30 – 1.24 (m, 12H), 1.08 (d, J = 12.2 Hz, 6H).

^{13}C NMR (101 MHz, CDCl$_3$) δ 157.0, 136.4, 130.2, 123.6, 120.6, 112.0, 84.1, 68.3, 36.3, 24.8, 24.6, 24.5, 24.2, 21.4, 15.8. The signal of the α-B-carbon was not observed.

^{11}B NMR (128 MHz, CDCl$_3$) δ 31.5.

IR (ATR): ν = 3329, 2973, 2360, 1379, 1087, 1045, 908, 879, 729, 646 cm^{-1}.

HRMS m/z (ESI) calcd for $C_{21}H_{34}BClNaO_3$ $(M + Na)^+$: 403.2182; found:403.2174.

***2-(7-Chlorobicyclo[4.1.0]heptan-7-yl)-5,5-dimethyl-1,3,2-dioxaborinane* (44)**

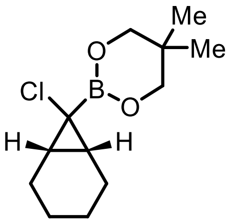

The title compound **44** was synthesized according to **General Procedure B**, and it was purified by column chromatography on silica gel (petroleum ether/ethyl acetate = 10:1), 29.2 mg, 60% yield,

colorless liquid.

^{1}H NMR (400 MHz, CDCl$_3$) δ 3.61 (s, 4H), 1.95 – 1.88 (m, 2H), 1.62 – 1.58 (m, 2H), 1.37 – 1.31 (m, 4H), 1.27 – 1.24 (m, 2H), 0.95 (s, 6H).

^{13}C NMR (101 MHz, CDCl$_3$) δ 72.5, 21.7, 21.6, 18.9, 18.7. The signal of the *α*-B-carbon was not observed.

^{11}B NMR (128 MHz, CDCl$_3$) δ 28.6.

IR (ATR): ν = 2926, 2253, 1478, 1254, 1163, 904, 729, 649 cm^{-1}.

HRMS m/z (ESI) calcd for $C_{14}H_{25}BCl$ (M + H)$^+$: 239.1732; found:239.1739.

***2-(7-Chlorobicyclo[4.1.0]heptan-7-yl)-4,4,6-trimethyl-1,3,2-dioxaborinane* (45)**

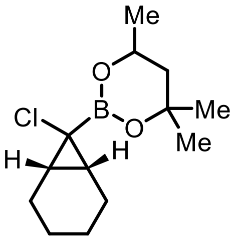

The title compound **45** was synthesized according to **General Procedure B**, and it was purified by column chromatography on silica gel (petroleum ether/ethyl acetate = 10:1), 41.2 mg, 80% yield, colorless liquid.

^{1}H NMR (400 MHz, CDCl$_3$) δ 4.23 – 4.13 (m, 1H), 1.93 – 1.83 (m, 2H), 1.73 (dd, *J* = 14.0, 2.8 Hz, 1H), 1.62 – 1.52 (m, 2H), 1.44 (dd, *J* = 14.0, 11.6 Hz, 1H), 1.37 – 1.31 (m, 2H), 1.30 – 1.26 (m, 2H), 1.25 (s, 3H), 1.23 (s, 6H), 1.23 – 1.18 (m, 2H).

^{13}C NMR (101 MHz, CDCl$_3$) δ 71.3, 65.3, 45.6, 31.0, 27.9, 23.0, 21.7, 18.9, 18.7, 18.6. The signal of the *α*-B-carbon was not observed.

^{11}B NMR (128 MHz, CDCl$_3$) δ 28.1.

IR (ATR): ν = 2978, 2252, 1399, 1299, 1166, 902 722, 649 cm^{-1}.

HRMS m/z (ESI) calcd for $C_{13}H_{23}BClO_2$ (M + H)$^+$: 257.1474; found:257.1468

***2-(7-Chlorobicyclo[4.1.0]heptan-7-yl)-4,4,6,6-tetramethyl-1,3,2-dioxaborinane* (46)**

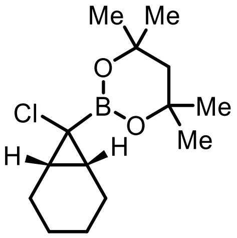

The title compound **46** was synthesized according to **General Procedure B**, and it was purified by column chromatography on silica gel (petroleum ether/ethyl acetate = 10:1), 32.9 mg, 61% yield, colorless liquid.

^{1}H NMR (400 MHz, CDCl$_3$) δ 1.94 – 1.84 (m, 2H), 1.77 (s, 2H), 1.63 – 1.53 (m, 2H), 1.39 – 1.32 (m, 2H), 1.30 (s, 12H), 1.28 – 1.18 (m, 4H).

^{13}C NMR (101 MHz, CDCl$_3$) δ 71.2, 48.7, 31.6, 21.7, 19.0, 18.5. The signal of the *α*-B-carbon was not observed.

^{11}B NMR (128 MHz, CDCl$_3$) δ 28.0.

IR (ATR): ν = 2976, 2252, 1508, 1382, 1045, 903, 722, 649 cm^{-1}.

HRMS m/z (ESI) calcd for $C_{14}H_{24}BClNaO_2$ (M + Na)$^+$: 293.1450; found:293.1442

***(3aS,4S,6S,7aR)-2-(7-Chlorobicyclo[4.1.0]heptan-7-yl)-3a,5,5-trimethylhexahydro-4,6-methanobenzo[d][1,3,2]dioxaborole* (47)**

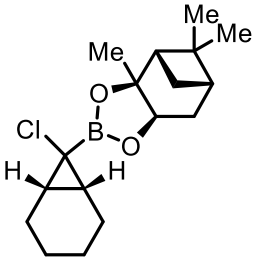

The title compound **47** was synthesized according to **General Procedure B**, and it was purified by column chromatography on silica gel (petroleum ether/ethyl acetate = 10:1), 40 mg, 65% yield, colorless liquid.

^{1}H NMR (400 MHz, CDCl$_3$) δ 4.32 (dd, *J* = 8.8, 2.0 Hz, 1H), 2.37 – 2.28 (m, 1H), 2.26 – 2.17 (m, 1H), 2.03 (dd, *J* = 6.0, 4.8 Hz, 1H), 1.98 – 1.84 (m, 4H), 1.67 – 1.56 (m, 3H), 1.40 – 1.38 (m, 1H), 1.38 (s, 3H), 1.37 – 1.36 (m, 1H), 1.35 – 1.29 (m, 2H), 1.27 (s, 3H), 1.25 (s, 1H), 1.13 (d, *J* = 11.2 Hz, 1H), 0.82 (s, 3H).

^{13}C NMR (101 MHz, CDCl$_3$) δ 86.8, 78.7, 51.3, 39.4, 35.4, 28.5, 27.0, 26.4, 23.9, 21.5, 19.3, 19.06, 18.8. The signal of the α-B-carbon was not observed.

^{11}B NMR (128 MHz, CDCl$_3$) δ 32.5.

IR (ATR): ν = 2932, 2862, 2253, 1399, 1282, 903, 723, 649 cm^{-1}.

HRMS m/z (ESI) calcd for $C_{17}H_{27}BClO_2$ (M + H)$^+$: 309.1787; found: 309.1780.

***4,4,5,5-Tetramethyl-2-(3-phenylpropyl)-1,3,2-dioxaborolane* (48)**

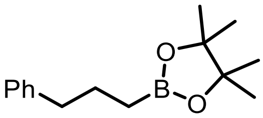

The title compound **48** was synthesized according to **General Procedure C**, and it was purified by column chromatography on silica gel (petroleum ether/ethyl acetate = 20:1), 30.5 mg, 62% yield, colorless liquid. All recorded spectroscopic data matched those previously reported in the literature[9].

^{1}H NMR (500 MHz, CDCl$_3$) δ 7.27 (t, *J* = 7.5 Hz, 2H), 7.22 – 7.13 (m, 3H), 2.64 – 2.59 (m, 2H), 1.74 (quint, *J* = 8.0 Hz, 2H), 1.25 (s, 12H), 0.84 (t, *J* = 8.0 Hz, 2H).

^{13}C NMR (126 MHz, CDCl$_3$) δ 142.7, 128.5, 128.4, 128.1, 125.5, 82.9, 38.6, 26.1, 24.8, 11.0 (br, low intensity).

^{11}B NMR (160 MHz, CDCl$_3$) δ 34.2.

***2-(3-(4-(Tert-butyl)phenyl)propyl)-4,4,5,5-tetramethyl-1,3,2-dioxaborolane* (49)**

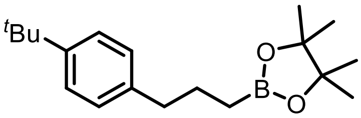

The title compound **49** was synthesized according to **General Procedure C**, and it was purified by column chromatography on silica gel (petroleum ether/ethyl acetate = 20:1), 31.6 mg, 53% yield, colorless liquid.

^{1}H NMR (400 MHz, CDCl$_3$) δ 7.31 – 7.27 (m, 2H), 7.14 – 7.10 (m, 2H), 2.61 – 2.56 (m, 2H), 1.78 – 1.69 (m, 2H), 1.31 (s, 9H), 1.25 (s, 12H), 0.84 (t, *J* = 8.0 Hz, 2H).
^{13}C NMR (101 MHz, CDCl$_3$) δ 148.3, 139.6, 128.2, 125.0, 82.9, 38.0, 31.4, 26.0, 24.8, 11.0 (br, low intensity).
^{11}B NMR (128 MHz, CDCl$_3$) δ 34.3.
IR (ATR): ν = 3358, 2974, 2247, 1320, 1087, 1046, 907, 879, 728, 646 cm^{-1}.
HRMS m/z (ESI) calcd for $C_{19}H_{32}BO_2$ (M + H)$^+$: 303.2490; found: 303.2480.

2-(3-([1,1'-Biphenyl]-4-yl)propyl)-4,4,5,5-tetramethyl-1,3,2-dioxaborolane **(50)**

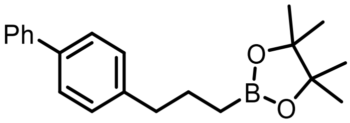

The title compound **50** was synthesized according to **General Procedure C**, and it was purified by column chromatography on silica gel (petroleum ether/ethyl acetate = 20:1), 46.4 mg, 72% yield, colorless liquid. The reaction was performed on a 1 mmol scale according to **General Procedure E**, 242 mg, 75% yield.
^{1}H NMR (400 MHz, CDCl$_3$) δ 7.41 – 7.37 (m, 2H), 7.33 – 7.30 (m, 2H), 7.26 – 7.21 (m, 2H), 7.16 – 7.11 (m, 1H), 7.09 – 7.05 (m, 2H), 2.47 (dd, *J* = 9.0, 6.5 Hz, 2H), 1.64 – 1.55 (m, 2H), 1.07 (s, 12H), 0.68 (t, *J* = 8.0 Hz, 2H).
^{13}C NMR (101 MHz, CDCl$_3$) δ 141.9, 141.3, 138.6, 129.0, 128.7, 127.0, 127.0, 126.9, 83.0, 38.2, 26.1, 24.9, 10.7(br, low intensity).
^{11}B NMR (128 MHz, CDCl$_3$) δ 34.1.
IR (ATR): ν = 3027, 2976, 1519, 1370, 1142, 967, 845, 761, 697 cm^{-1}.
HRMS m/z (ESI) calcd for $C_{21}H_{28}BO_2$ (M + H)$^+$: 323.2177; found: 323.2172.

2-(3-(4-Fluorophenyl)propyl)-4,4,5,5-tetramethyl-1,3,2-dioxaborolane **(51)**

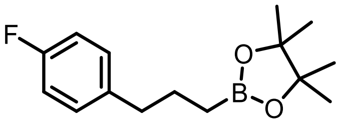

The title compound **51** was synthesized according to **General Procedure C**, and it was purified by column chromatography on silica gel (petroleum ether/ethyl acetate = 20:1), 24.7 mg, 47% yield, colorless liquid.
^{1}H NMR (400 MHz, CDCl$_3$) δ 7.15 – 7.09 (m, 2H), 6.97 – 6.90 (m, 2H), 2.60 – 2.54 (m, 2H), 1.75 – 1.65 (m, 2H), 1.24 (s, 12H), 0.80 (t, *J* = 8.0 Hz, 2H).
^{13}C NMR (101 MHz, CDCl$_3$) δ 161.2 (d, *J* = 242.6 Hz), 138.2 (d, *J* = 3.2 Hz), 129.8 (d, *J* = 7.7 Hz), 114.8 (d, *J* = 20.9 Hz), 83.0, 37.7, 26.2, 24.8. The signal of the α-B-carbon was not observed.
^{19}F NMR (376 MHz, CDCl$_3$) δ -118.3.
^{11}B NMR (128MHz, CDCl$_3$) δ 34.0.
IR (ATR): ν = 2976, 2361, 1509, 1374, 1221, 1088, 908, 730 cm^{-1}.
HRMS m/z (ESI) calcd for $C_{15}H_{23}BFO_2$ (M + H)$^+$: 265.1770; found: 265.1771.

2-(3-(4-Chlorophenyl)propyl)-4,4,5,5-tetramethyl-1,3,2-dioxaborolane **(52)**

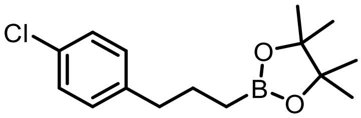

The title compound **52** was synthesized according to **General Procedure C**, and it was purified by column chromatography on silica gel (petroleum ether/ethyl acetate = 20:1), 27.5 mg, 49% yield, colorless liquid. All recorded spectroscopic data matched those previously reported in the literature[9].

^{1}H NMR (500 MHz, CDCl$_3$) δ 7.23 – 7.20 (m, 2H), 7.11 – 7.08 (m, 2H), 2.59 – 2.55 (m, 2H), 1.73 – 1.67 (m, 2H), 1.24 (s, 12H), 0.80 (t, J = 8.0 Hz, 2H).

^{13}C NMR (126 MHz, CDCl$_3$) δ 141.1, 131.2, 129.9, 128.2, 83.0, 37.8, 25.9, 24.8. The signal of the α-B-carbon was not observed.

^{11}B NMR (160 MHz, CDCl$_3$) δ 39.4.

***4,4,5,5-Tetramethyl-2-(3-(thiophen-2-yl)propyl)-1,3,2-dioxaborolane* (53)**

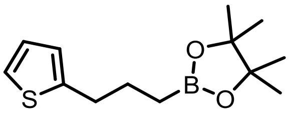

The title compound **53** was synthesized according to **General Procedure C**, and it was purified by column chromatography on silica gel (petroleum ether/ethyl acetate = 20:1), 33.9 mg, 67% yield, colorless liquid.

^{1}H NMR (400 MHz, CDCl$_3$) δ 7.09 (dd, J = 5.2, 1.2 Hz, 1H), 6.92 – 6.88 (m, 1H), 6.79 – 6.76 (m, 1H), 2.85 – 2.81 (m, 2H), 1.79 (quint, J = 7.6 Hz, 2H), 1.25 (s, 12H), 0.86 (t, J = 8.0 Hz, 2H).

^{13}C NMR (101 MHz, CDCl$_3$) δ 145.5, 126.6, 124.1, 122.7, 83.0, 32.3, 26.4, 24.8. The signal of the α-B-carbon was not observed.

^{11}B NMR (128 MHz, CDCl$_3$) δ 33.8.

IR (ATR): ν = 2980, 2251, 1373, 1143, 903, 723, 649 cm^{-1}.

HRMS m/z (ESI) calcd for $C_{13}H_{22}BO_2S$ (M + H)$^+$: 253.1428; found: 253.1423.

***4,4,5,5-Tetramethyl-2-phenethyl-1,3,2-dioxaborolane* (54)**

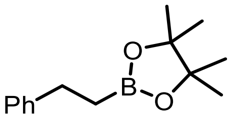

The title compound **54** was synthesized according to **General Procedure C**, and it was purified by column chromatography on silica gel (petroleum ether/ethyl acetate = 20:1), 26.2 mg, 56% yield, colorless liquid.

^{1}H NMR (400 MHz, CDCl$_3$) δ 7.29 – 7.18 (m, 4H), 7.18 – 7.06 (m, 1H), 2.76 – 2.71 (m, 2H), 1.21 (s, 12H), 1.13 (t, J = 8.2 Hz, 2H).

^{13}C NMR (126 MHz, CDCl$_3$) δ 144.4, 128.2, 128.0, 125.0, 83.1, 29.9, 24.8. The signal of the α-B-carbon was not observed.

^{11}B NMR (160 MHz, CDCl$_3$) δ 39.4.

IR (ATR): ν = 3328, 2973, 2361, 1379, 1087, 1045, 909, 879, 731, 646 cm^{-1}.

HRMS m/z (ESI) calcd for $C_{14}H_{22}BO_2$ (M + H)$^+$: 233.1707; found: 233.1701.

***4,4,5,5-Tetramethyl-2-(4-phenylbutyl)-1,3,2-dioxaborolane* (55)**

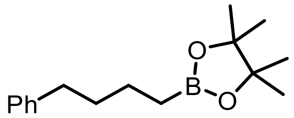

The title compound **55** was synthesized according to **General Procedure C**, and it was purified by column chromatography on silica gel (petroleum ether/ethyl acetate = 10:1), 27.6 mg, 53% yield, colorless liquid.

^{1}H NMR (500 MHz, $CDCl_3$) δ 7.26 – 7.23 (m, 2H), 7.17 – 7.13 (m, 3H), 2.59 (t, *J* = 8.0 Hz, 2H), 1.63 – 1.59 (m, 2H), 1.46 (quint, *J* = 8.0 Hz, 2H), 1.22 (s, 12H), 0.80 (t, *J* = 8.0 Hz, 2H).

^{13}C NMR (126 MHz, $CDCl_3$) δ 142.9, 128.4, 128.2, 125.5, 82.9, 35.8, 34.2, 29.7, 24.8, 23.7. The signal of the *α*-B-carbon was not observed.

^{11}B NMR (160 MHz, $CDCl_3$) δ 39.3.

IR (ATR): ν = 2975, 2359, 1378, 1086, 1046, 905, 726, 648 cm^{-1}.

HRMS m/z (ESI) calcd for $C_{16}H_{26}BO_2$ $(M + H)^+$: 261.2020; found: 261.2012.

***2-(4-(Benzyloxy)butyl)-4,4,5,5-tetramethyl-1,3,2-dioxaborolane* (56)**

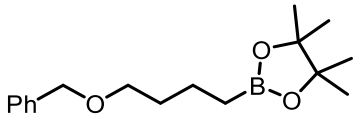

The title compound **56** was synthesized according to **General Procedure C**, and it was purified by column chromatography on silica gel (petroleum ether/ethyl acetate = 20:1), 34.2 mg, 59% yield, colorless liquid.

^{1}H NMR (400 MHz, $CDCl_3$) δ 7.33 (d, *J* = 4.4 Hz, 3H), 7.31 – 7.26 (m, 2H), 4.49 (s, 2H), 3.47 (t, *J* = 6.4 Hz, 2H), 1.65 – 1.60 (m, 2H), 1.53 – 1.47 (m, 2H), 1.24 (s, 12H), 0.80 (t, *J* = 7.6 Hz, 2H).

^{13}C NMR (101 MHz, $CDCl_3$) δ 138.7, 128.3, 127.6, 127.4, 82.9, 72.8, 70.3, 32.2, 24.8, 20.6. The signal of the *α*-B-carbon was not observed.

^{11}B NMR (128 MHz, $CDCl_3$) δ 34.0.

IR (ATR): ν = 3337, 2973, 2246, 1380, 1087, 1046, 908, 879, 728, 646 cm^{-1}.

HRMS m/z (ESI) calcd for $C_{17}H_{28}BO_3$ $(M + H)^+$: 291.2126; found: 291.2117.

***4,4,5,5-Tetramethyl-2-(undec-10-en-1-yl)-1,3,2-dioxaborolane* (57)**

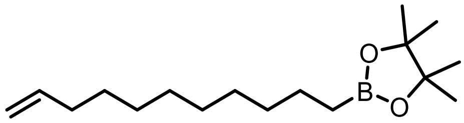

The title compound **57** was synthesized according to **General Procedure C**, and it was purified by column chromatography on silica gel (petroleum ether/ethyl acetate = 20:1), 32.7 mg, 58% yield, colorless liquid. The reaction was performed on a 1 mmol scale according to **General Procedure E**, 185 mg, 66% yield.

^{1}H NMR (400 MHz, $CDCl_3$) δ 5.86 – 5.75 (m, 1H), 5.02 – 4.95 (m, 1H), 4.94 – 4.89 (m, 1H), 2.06 – 2.00 (m, 2H), 1.42 – 1.34 (m, 4H), 1.26 (d, *J* = 2.1 Hz, 10H), 1.24 (s, 12H), 0.76 (t, *J* = 7.6 Hz, 2H).

^{13}C NMR (101 MHz, $CDCl_3$) δ 139.3, 114.0, 82.8, 33.8, 32.4, 29.5, 29.5, 29.4, 29.1, 29.0, 24.8, 24.0. The signal of the *α*-B-carbon was not observed.

^{11}B NMR (128 MHz, $CDCl_3$) δ 34.1.

IR (ATR): ν = 3335, 2973, 2362, 1379, 1087, 1045, 908, 730 cm^{-1}.

HRMS m/z (ESI) calcd for $C_{17}H_{34}BO_2$ $(M + H)^+$: 281.2646; found: 281.2647.

***Trimethyl(5-(4,4,5,5-tetramethyl-1,3,2-dioxaborolan-2-yl)pent-1-yn-1-yl)silane* (58)**

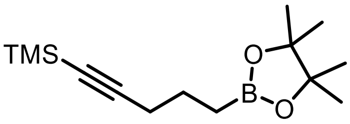

The title compound **58** was synthesized according to **General Procedure C**, and it was purified by column chromatography on silica gel (petroleum ether/ethyl acetate = 20:1), 23.5 mg, 44% yield, colorless liquid.

^{1}H NMR (400 MHz, CDCl$_3$) δ 2.23 (t, *J* = 7.3 Hz, 2H), 1.65 – 1.61 (m, 2H), 1.24 (s, 12H), 0.87 (s, 2H), 0.13 (s, 9H).

^{13}C NMR (101 MHz, CDCl$_3$) δ 107.6, 84.4, 83.0, 29.7, 24.8, 23.3, 22.2, 0.2. The signal of the α-B-carbon was not observed.

^{11}B NMR (128 MHz, CDCl$_3$) δ 33.8.

IR (ATR): ν = 3341, 2973, 2245, 1379, 1087, 1046, 908, 879, 729, 646 cm^{-1}.

HRMS m/z (ESI) calcd for $C_{14}H_{28}BO_2Si$ $(M + H)^+$: 267.1946; found: 267.1945.

***4,4,5,5-Tetramethyl-2-(2-phenylpropyl)-1,3,2-dioxaborolane* (59)**

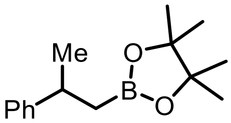

The title compound **59** was synthesized according to **General Procedure C**, and it was purified by column chromatography on silica gel (petroleum ether/ethyl acetate = 20:1), 26.9 mg, 55% yield, colorless liquid. All recorded spectroscopic data matched those previously reported in the literature[10].

^{1}H NMR (400 MHz, CDCl$_3$) δ 7.24 (s, 4H), 7.25 – 7.18 (m, 5H), 7.17 – 7.09 (m, 2H), 3.00 (dt, *J* = 14.4, 7.2 Hz, 1H), 1.29 – 1.22 (m, 4H), 1.14 (s, 13H).

^{13}C NMR (101 MHz, CDCl$_3$) δ 149.2, 128.1, 126.6, 125.6, 83.0, 35.8, 24.9, 24.7, 24.7. The signal of the *α*-B-carbon was not observed.

^{11}B NMR (128 MHz, CDCl$_3$) δ 33.7.

***4,4,5,5-tetramethyl-2-(2-(p-tolyl)propyl)-1,3,2-dioxaborolane* (60)**

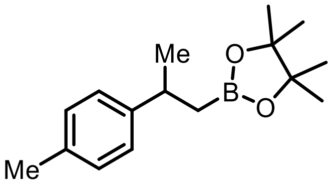

The title compound **60** was synthesized according to **General Procedure C**, and it was purified by column chromatography on silica gel (petroleum ether/ethyl acetate = 20:1), 26.7 mg, 52% yield, colorless liquid.

^{1}H NMR (400 MHz, CDCl$_3$) δ 7.13 (d, *J* = 8.0 Hz, 2H), 7.08 (d, *J* = 8.0 Hz, 2H), 3.01 (dd, *J* = 15.2, 6.8 Hz, 1H), 2.31 (s, 3H), 1.26 (d, *J* = 6.8 Hz, 3H), 1.18 (s, 12H), 1.14 (dd, *J* = 7.6, 4.4 Hz, 2H).

^{13}C NMR (101 MHz, CDCl$_3$) δ 146.3, 135.0, 128.8, 126.4, 82.9, 35.3, 24.9, 24.8, 24.7, 20.9. The signal of the *α*-B-carbon was not observed.

^{11}B NMR (128 MHz, CDCl$_3$) δ 33.4.

IR (ATR): ν = 2973, 2263, 1455, 1087, 1045, 911, 879, 732 cm^{-1}.
HRMS m/z (ESI) calcd for $C_{16}H_{26}BO_2$ (M + H)$^+$: 261.2020; found: 261.2018.

***2-(3-(Benzo[d][1,3]dioxol-5-yl)-2-methylpropyl)-4,4,5,5-tetramethyl-1,3,2-dioxaborolane* (61)**

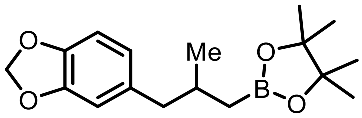

The title compound **61** was synthesized according to **General Procedure C**, and it was purified by column chromatography on silica gel (petroleum ether/ethyl acetate = 20:1), 30.4 mg, 50% yield, colorless liquid.
^{1}H NMR (400 MHz, CDCl$_3$) δ 6.70 (d, *J* = 8.0 Hz, 1H), 6.66 (d, *J* = 1.6 Hz, 1H), 6.59 (dd, *J* = 7.8, 1.6 Hz, 1H), 5.90 (s, 2H), 2.51 (dd, *J* = 13.2, 6.4 Hz, 1H), 2.35 (dd, *J* = 13.2, 7.6 Hz, 1H), 2.00 – 1.87 (m, 1H), 1.24 (s, 12H), 0.89 (d, *J* = 6.6 Hz, 3H), 0.87 – 0.80 (m, 1H), 0.66 (dd, *J* = 15.6, 8.4 Hz, 1H).
^{13}C NMR (101 MHz, CDCl$_3$) δ 147.3, 145.4, 135.5, 122.0, 109.6, 107.8, 100.6, 82.9, 45.7, 31.9, 24.9, 24.8, 21.9. The signal of the α-B-carbon was not observed.
^{11}B NMR (128 MHz, CDCl$_3$) δ 33.8.
IR (ATR): ν = 2974, 2364, 1377, 1087, 1045, 908, 873, 729 cm^{-1}.
HRMS m/z (ESI) calcd for $C_{17}H_{26}BO_4$ (M + H)$^+$: 305.1919; found: 305.1909.

***2-(((1r,4r)-4-(4-Chlorophenyl)cyclohexyl)methyl)-4,4,5,5-tetramethyl-1,3,2-dioxaborolane* (62)**

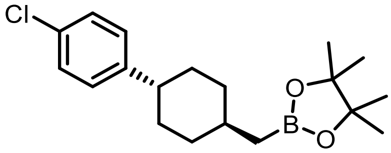

The title compound **62** was synthesized according to **General Procedure C**, and it was purified by column chromatography on silica gel (petroleum ether/ethyl acetate = 20:1), 33.0 mg, 49% yield, colorless liquid.
^{1}H NMR (400 MHz, CDCl$_3$) δ 7.25 – 7.21 (m, 2H), 7.15 – 7.10 (m, 2H), 2.41 (tt, *J* = 12.0, 3.2 Hz, 1H), 1.88 – 1.78 (m, 4H), 1.61 – 1.55 (m, 1H), 1.50 – 1.40 (m, 2H), 1.26 (s, 12H), 1.16 – 1.06 (m, 2H), 0.77 (d, *J* = 7.2 Hz, 2H).
^{13}C NMR (101 MHz, CDCl$_3$) δ 146.3, 131.2, 128.3, 128.2, 82.9, 43.6, 35.9, 34.4, 33.8, 24.8. The signal of the α-B-carbon was not observed.
^{11}B NMR (128 MHz, CDCl$_3$) δ 33.5.
IR (ATR): ν = 2975, 2362, 1374, 1089, 1047, 905, 726, 648 cm^{-1}.
HRMS m/z (ESI) calcd for $C_{19}H_{29}BClO_2$ (M + H)$^+$: 335.1944;found: 335.1958.

***2-((2,3-Dihydro-1H-inden-2-yl)methyl)-4,4,5,5-tetramethyl-1,3,2-dioxaborolane* (63)**

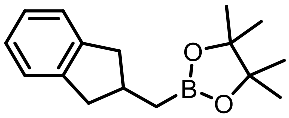

The title compound **63** was synthesized according to **General Procedure C**, and it was purified by column chromatography on silica gel (petroleum ether/ethyl acetate = 20:1), 23.2 mg, 45% yield, colorless liquid.

^{1}H NMR (400 MHz, CDCl$_3$) δ 7.19 – 7.14 (m, 2H), 7.13 – 7.07 (m, 2H), 3.08 (dd, *J* = 14.4, 6.8 Hz, 2H), 2.68 – 2.55 (m, 3H), 1.26 (s, 12H), 1.05 (d, *J* = 7.2 Hz, 2H).
^{13}C NMR (101 MHz, CDCl$_3$) δ 143.8, 125.9, 124.3, 83.0, 41.5, 36.2, 24.8. The signal of the α-B-carbon was not observed.
^{11}B NMR (128 MHz, CDCl$_3$) δ 33.8.
IR (ATR): ν = 2983, 2362, 1373, 1144, 902, 722, 650 cm^{-1}.
HRMS m/z (ESI) calcd for $C_{16}H_{24}BO_2$ (M + H)$^+$: 259.1864; found: 259.1856.

***4,4,5,5-Tetramethyl-2-(2-phenylcyclopropyl)-1,3,2-dioxaborolane* (64)**

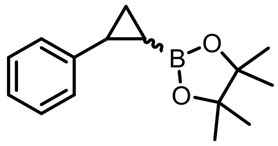

The title compound **64** was synthesized according to **General Procedure C**, and it was purified by column chromatography on silica gel (petroleum ether/ethyl acetate = 20:1), 26.7 mg, 55% yield, d.r. = 1:0.7, colorless liquid. All recorded spectroscopic data matched those previously reported in the literature[11].
^{1}H NMR (500 MHz, CDCl$_3$) δ 7.27 (d, *J* = 7.5 Hz, 2H), 7.24 – 7.18 (m, 2H), 7.16 – 7.09 (m, 1H), 2.35 (dt, *J* = 10.5, 8.0, 6.0 Hz, 1H), 1.30 – 1.28 (m, 1H), 1.12 – 1.08 (m, 1H), 1.01 (s, 6H), 0.88 (s, 6H), 0.49 – 0.39 (m, 1H).
^{13}C NMR (126 MHz, CDCl$_3$) δ 140.7, 128.8, 127.6, 125.7, 82.9, 24.7, 24.4, 21.7, 8.9. The signal of the α-B-carbon was not observed.
^{11}B NMR (160 MHz, CDCl$_3$) δ 38.3.
IR (ATR): ν = 2981, 2252, 1410, 1219, 1143, 903, 722, 649 cm^{-1}.
HRMS m/z (ESI) calcd for $C_{15}H_{22}BO_2$ (M + H)$^+$: 245.1707; found: 245.1699.

***2-(2-(2-Chlorophenyl)-2-(4-chlorophenyl)ethyl)-4,4,5,5-tetramethyl-1,3,2-dioxaborolane* (65)**

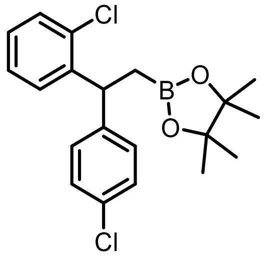

The title compound **65** was synthesized according to **General Procedure C**, and it was purified by column chromatography on silica gel (petroleum ether/ethyl acetate = 20:1), 38.8 mg, 52% yield, colorless liquid.
^{1}H NMR (500 MHz, CDCl$_3$) δ 7.36 – 7.29 (m, 2H), 7.24 – 7.19 (m, 5H), 7.14 – 7.09 (m, 1H), 4.75 (t, *J* = 8.4 Hz, 1H), 1.55 (dd, *J* = 8.4, 3.2 Hz, 2H), 1.08 (s, 6H), 1.06 (s, 6H).
^{13}C NMR (126 MHz, CDCl$_3$) δ 143.8, 143.2, 133.9, 131.7, 129.6, 129.3, 128.34, 128.25, 127.4, 126.9, 83.3, 41.9, 24.5. The signal of the α-B-carbon was not observed.
^{11}B NMR (128 MHz, CDCl$_3$) δ 33.7.
IR (ATR): ν = 2975, 2362, 1374, 1087, 1047, 904, 724, 648 cm^{-1}.
HRMS m/z (ESI) calcd for $C_{20}H_{24}BCl_2O_2$ (M + H)$^+$: 377.1241; found: 377.1237.

***4,5-Diphenyl-2-(3-(4,4,5,5-tetramethyl-1,3,2-dioxaborolan-2-yl)propyl)oxazole* (66)**

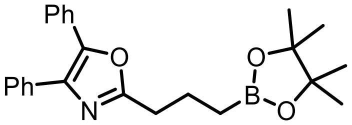

The title compound **66** was synthesized according to **General Procedure C**, and it was purified by column chromatography on silica gel (petroleum ether/ethyl acetate = 3:1), 60.2 mg, 77% yield, colorless liquid.

^{1}H NMR (400 MHz, CDCl$_3$) δ 7.66 – 7.61 (m, 2H), 7.61 – 7.55 (m, 2H), 7.38 – 7.34 (m, 3H), 7.34 – 7.27 (m, 3H), 2.88 (t, J = 7.6 Hz, 2H), 1.96 (quint, J = 7.8 Hz, 2H), 1.25 (s, 12H), 0.94 (t, J = 7.9 Hz, 2H).

^{13}C NMR (101 MHz, CDCl$_3$) δ 163.8, 145.0, 134.8, 132.5, 129.1, 128.6, 128.5, 128.3, 128.0, 126.4, 83.1, 30.5, 24.8, 21.9. The signal of the α-B-carbon was not observed.

^{11}B NMR (128 MHz, CDCl$_3$) δ 34.3.

IR (ATR): ν = 3335, 2974, 2362, 1319, 1143, 1087, 1046, 908, 879, 730 cm^{-1}.

HRMS m/z (ESI) calcd for $C_{24}H_{29}BNO_3$ (M + H)$^+$: 390.2232; found: 390.2232.

***4,4,5,5-Tetramethyl-2-(1-phenylhex-5-en-3-yl)-1,3,2-dioxaborolane* (67)**

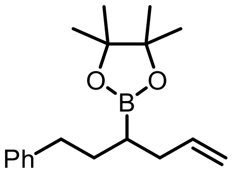

The title compound **67** was synthesized according to **General Procedure F**, and it was purified by column chromatography on silica gel (petroleum ether/ethyl acetate = 20:1), 36.1 mg, 63% yield, colorless liquid.

^{1}H NMR (400 MHz, CDCl$_3$) δ 7.27 – 7.25 (m, 2H), 7.18 – 7.15 (m, 3H), 5.86 – 5.76 (m, 1H), 5.05 – 5.00 (m, 1H), 4.96 – 4.93 (m, 1H), 2.63 – 2.59 (m, 2H), 2.24 – 2.16 (m, 2H), 1.77 – 1.73 (m, 1H), 1.71 – 1.66 (m, 1H), 1.26 (s, 12H), 1.19 – 1.09 (m, 1H).

^{13}C NMR (101 MHz, CDCl$_3$) δ 142.9, 138.4, 128.4, 128.2, 125.6, 115.0, 83.1, 35.44, 35.35, 32.9, 24.9, 24.8.

***2-(1-(4-Methoxyphenyl)-3-phenylpropyl)-4,4,5,5-tetramethyl-1,3,2-dioxaborolane* (68)**

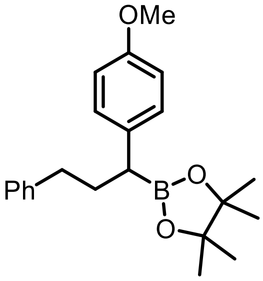

The title compound **68** was synthesized according to **General Procedure F**, and it was purified by column chromatography on silica gel (petroleum ether/ethyl acetate = 25:1), 60.5 mg, 86% yield, colorless liquid.

^{1}H NMR (400 MHz, CDCl$_3$) δ 7.30 – 7.22 (m, 2H), 7.20 – 7.07 (m, 5H), 6.90 – 6.79 (m, 2H), 3.79 (s, 3H), 2.60 – 2.54 (m, 2H), 2.31 (t, *J* = 8.0 Hz, 1H), 2.19 – 2.05 (m, 1H), 2.01 – 1.85 (m, 1H), 1.22 (s, 6H), 1.20 (s, 6H).
^{13}C NMR (101 MHz, CDCl$_3$) δ 157.3, 142.6, 134.8, 129.2, 128.5, 128.2, 125.6, 113.8, 83.2, 55.1, 35.3, 34.5, 24.7, 24.6.

***Methyl 4-(3-phenylpropyl)benzoate* (69)**

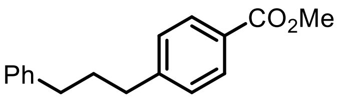

The title compound **69** was synthesized according to **General Procedure F**, and it was purified by column chromatography on silica gel (petroleum ether/ethyl acetate = 25:1), 31.3 mg, 62% yield, colorless liquid.
^{1}H NMR (400 MHz, CDCl$_3$) δ 7.98 (d, *J* = 8.2 Hz, 2H), 7.32 – 7.24 (m, 4H), 7.23 – 7.17 (m, 3H), 3.91 (s, 3H), 2.74 – 2.69 (m, 2H), 2.69 – 2.64 (m, 2H), 2.04 – 1.94 (m, 2H).
^{13}C NMR (101 MHz, CDCl$_3$) δ 167.1, 147.8, 141.9, 129.6, 128.4, 128.3, 127.8, 125.8, 51.9, 35.4, 35.3, 32.5.

***2-Methylquinoline* (70)**

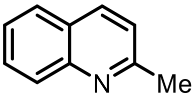

The title compound **70** was synthesized according to **General Procedure F**, and it was purified by column chromatography on silica gel (petroleum ether/ethyl acetate = 10:1), 15.7 mg, 55% yield, light yellow liquid.
^{1}H NMR (400 MHz, CDCl$_3$) δ 8.02 (dd, *J* = 8.4, 1.6 Hz, 2H), 7.75 (dd, *J* = 8.0, 1.6 Hz, 1H), 7.70 – 7.63 (m, 1H), 7.50 – 7.41 (m, 1H), 7.26 (d, *J* = 8.4 Hz, 1H), 2.73 (s, 3H).
^{13}C NMR (101 MHz, CDCl$_3$) δ 158.9, 147.8, 136.1, 129.4, 128.5, 127.4, 125.6, 121.9, 25.28.

***2-(4-Methoxyphenyl)-4,4,5,5-tetramethyl-1,3,2-dioxaborolane* (71)**

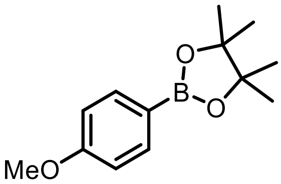

The title compound **71** was synthesized according to **General Procedure F**, and it was purified by column chromatography on silica gel (petroleum ether/ethyl acetate = 15:1), 35.2 mg, 75% yield, light yellow liquid.
^{1}H NMR (400 MHz, CDCl$_3$) δ 7.81 – 7.72 (m, 2H), 6.90 (d, *J* = 8.8 Hz, 2H), 3.82 (s, 3H), 1.34 (s, 12H).
^{13}C NMR (101 MHz, CDCl$_3$) δ 162.1, 136.5, 113.3, 83.5, 55.0, 24.8.

***2-(7-Allylbicyclo[4.1.0]heptan-7-yl)-4,4,5,5-tetramethyl-1,3,2-dioxaborolane* (72)**

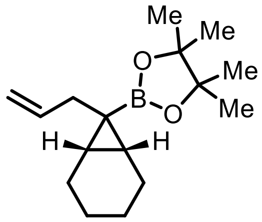

The title compound **72** was synthesized according to **General Procedure F**, and it was purified by column chromatography on silica gel (petroleum ether/ethyl acetate = 20:1), 36.7 mg, 70% yield, light yellow liquid.

^{1}H NMR (500 MHz, CDCl$_3$) δ 5.89 – 5.79 (m, 1H), 5.01 (dd, *J* = 17.0, 2.0 Hz, 1H), 4.95 – 4.87 (m, 1H), 1.97 – 1.91 (m, 2H), 1.89 – 1.83 (m, 2H), 1.76 – 1.70 (m, 2H), 1.24 (s, 12H), 1.21 – 1.18 (m, 2H), 0.90 – 0.84 (m, 2H), 0.76 (d, *J* = 6.0 Hz, 2H).

^{13}C NMR (126 MHz, CDCl$_3$) δ 138.0, 114.5, 82.7, 44.3, 29.7, 25.0, 22.1, 21.7, 21.0. The signal of the *α*-B-carbon was not observed.

^{11}B NMR (160 MHz, CDCl$_3$) δ 33.4.

IR (ATR): ν = 2982, 2253, 1389, 1144, 1094, 903, 724, 650 cm^{-1}.

HRMS m/z (ESI) calcd for $C_{16}H_{28}BO_2$ (M + H)$^+$: 263.2177; found: 263.2172.

Examining Functional-Group Compatibility

In order to further prove the possibility of this reaction for biological application. A broad of biomolecules were selected including amino acids, peptides, starch, DNA, RNA, and heteroatom-containing molecules. In addition, all bimolecules was commercially available. DNA sodium salt from salmon testes (stDNA, >90%) and Ribonucleic acid (RNA, yeast) were acquired from Shanghai yuanye Bio-Technology Co., Ltd. (Shanghai, China, www.shyuanye.com). The reaction was performed according to **General Procedure A, B** and **C** in the presence of an additive. After the reaction is finished, the small biomolecules can be tested by HNMR and HRMS. From MS-ESI (negative mode), the added amino acid and peptide molecules can be found and its formation was analyzed by ^{1}H NMR analysis. This implies that the added biomolecule remains intact in the reaction mixture. The reaction is not affected for some macromolecules such as Porcine trypsin, starch, Bovine serum albumin, DNA and RNA, but they are difficult to detect these biomolecules after finishing the reactions by appropriate means. This result is also in accordance with the previous literature for these macromolecules[12]. The recovery of the heteroatom-containing additive was isolated and given in parentheses. As shown in the following, all reactions worked and gave the desired product in these biomolecules' presence.

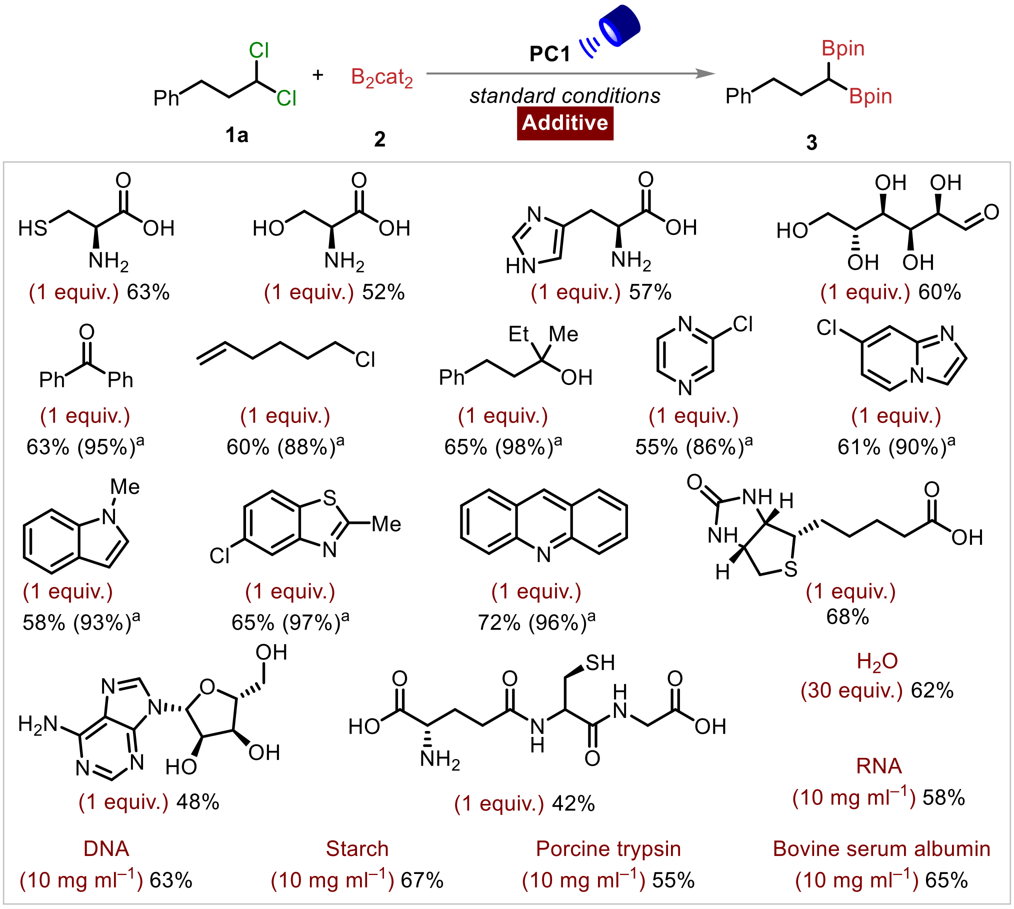

Supplementary Figure 3. Investigation of biocompatibility of *gem*-diborylation. Standard reaction conditions. After the reaction was complete, the reaction mixture was analyzed and the isolated yield was shown above. [a]Recovery yields of functionalized additives were given in parentheses.

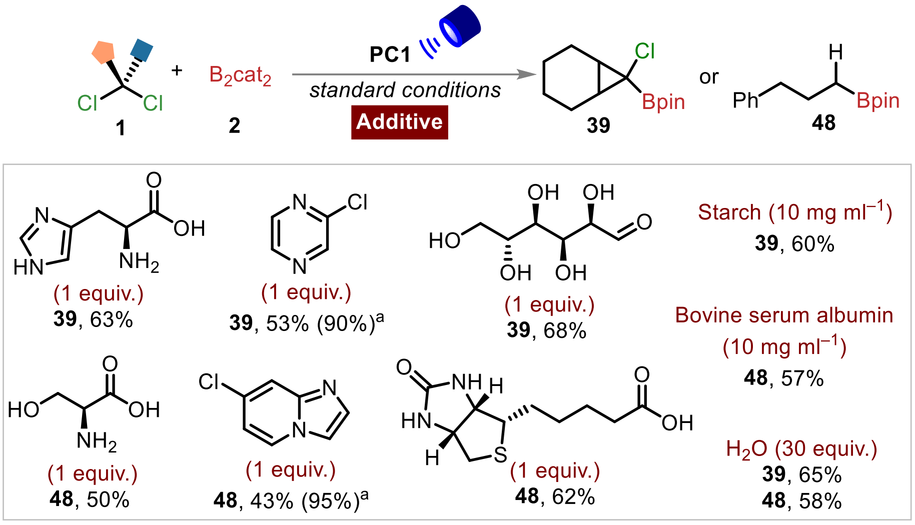

Supplementary Figure 4. Investigation of biocompatibility of monoborylation and hydroborylation. Standard reaction conditions. After the reaction was complete, the reaction mixture was analyzed and the isolated yield was shown above. [a]Recovery yields of functionalized additives were given in parentheses.

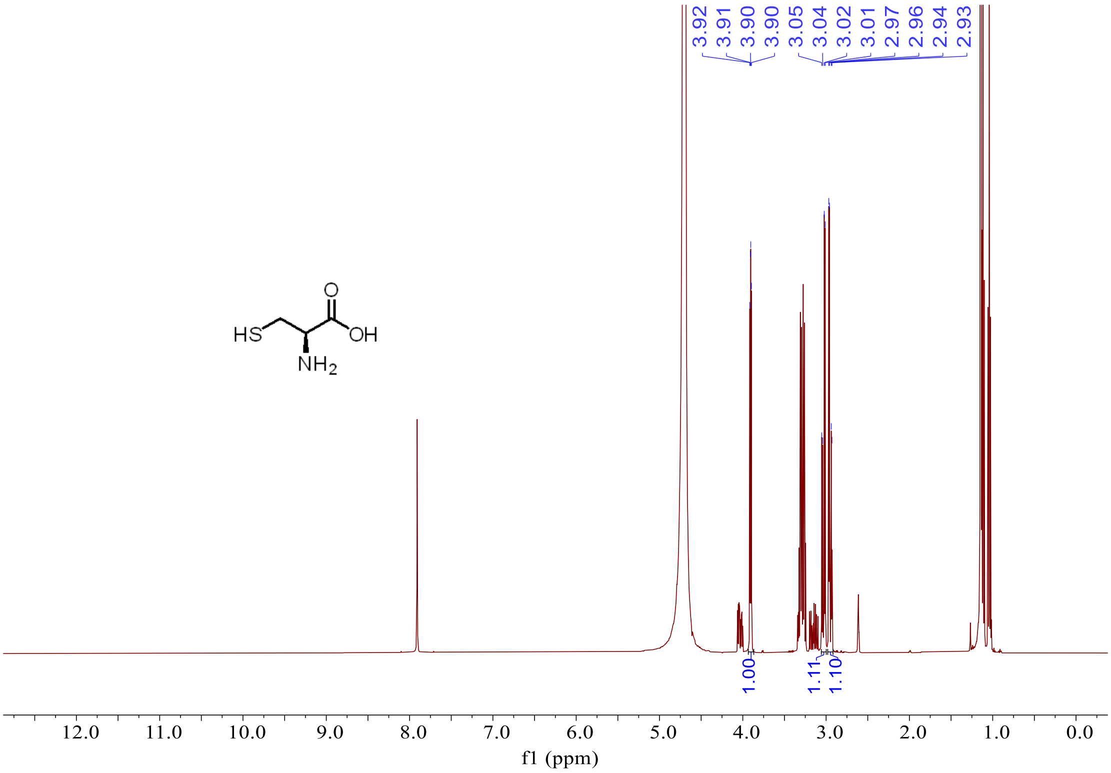

Supplementary Figure 5. ^{1}H NMR spectrum of reaction mixture with bio-additive of *L*-Cysteine

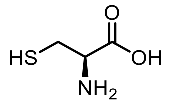

L-Cysteine
M = 121.0197
[M-H]$^-$ = 120.0108

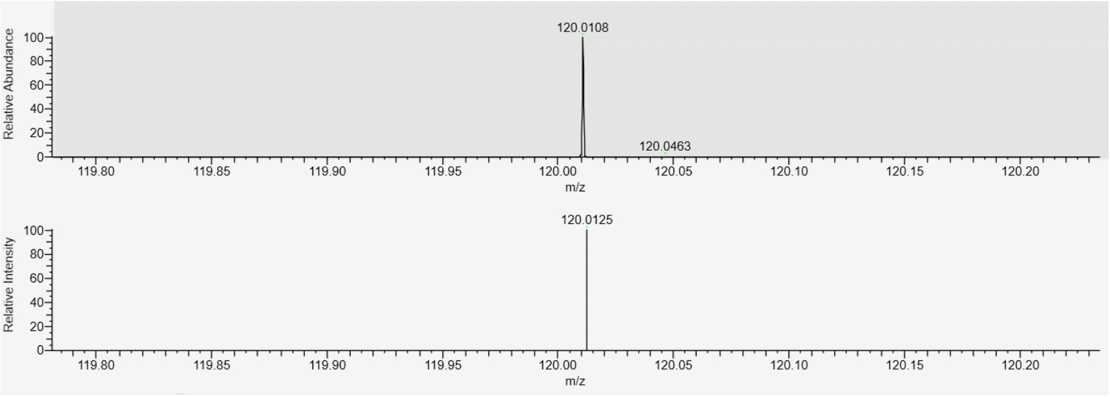

Supplementary Figure 6. HR-MS spectrum of reaction mixture with bio-additive of *L*-Cysteine

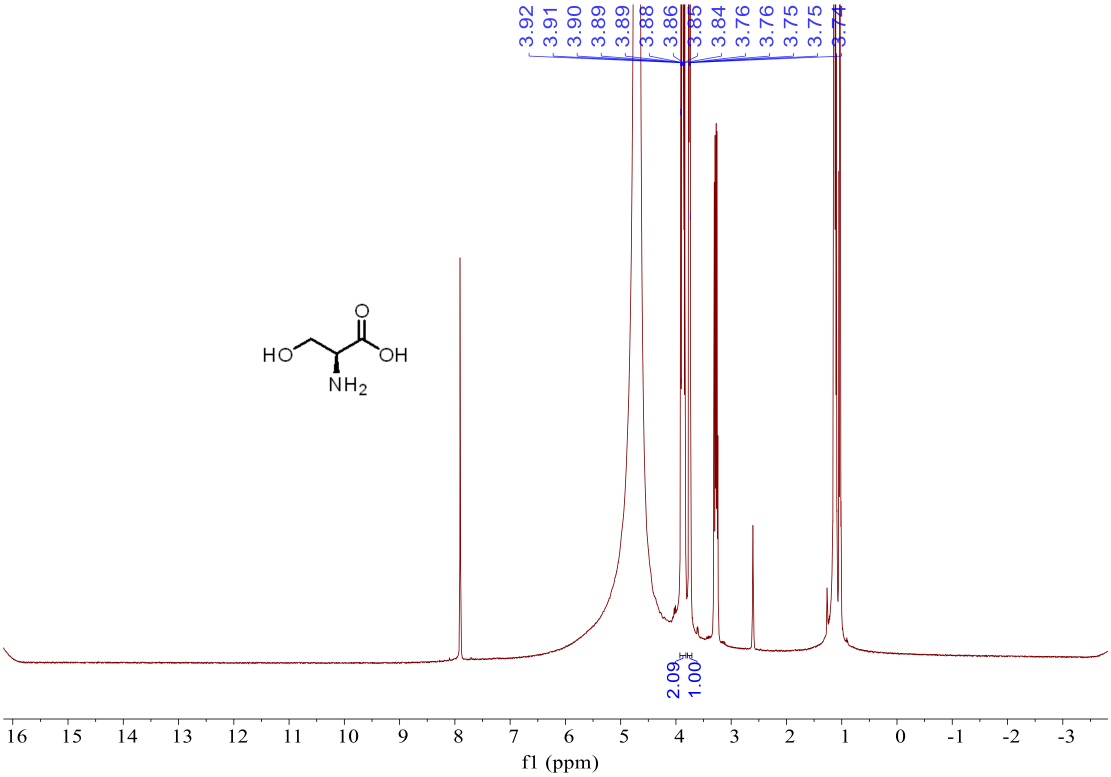

Supplementary Figure 7. ^{1}H NMR spectrum of reaction mixture with bio-additive of *L*-Serine

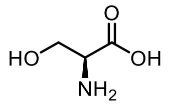

L-Serine
M = 105.0426
[M-H]$^-$ = 104.0338

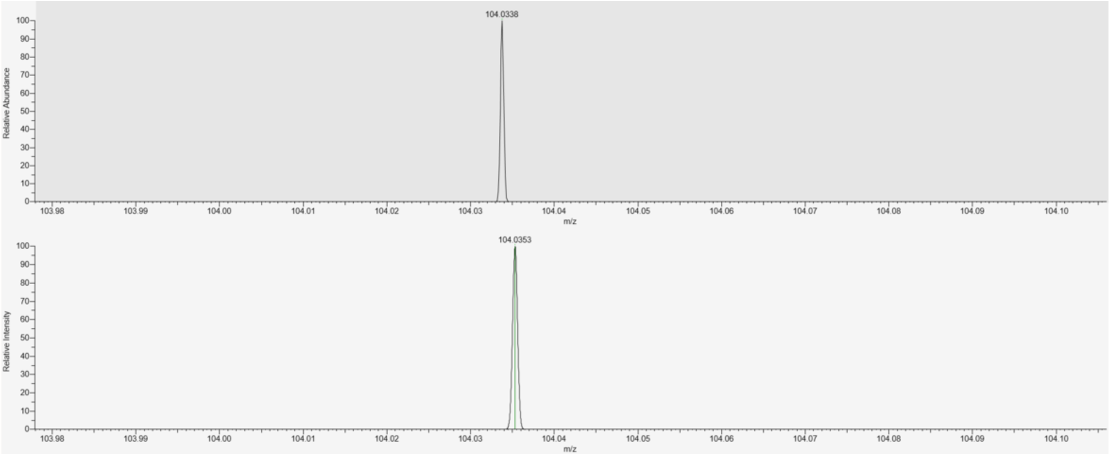

Supplementary Figure 8. HR-MS spectrum of reaction mixture with bio-additive of *L*-Serine

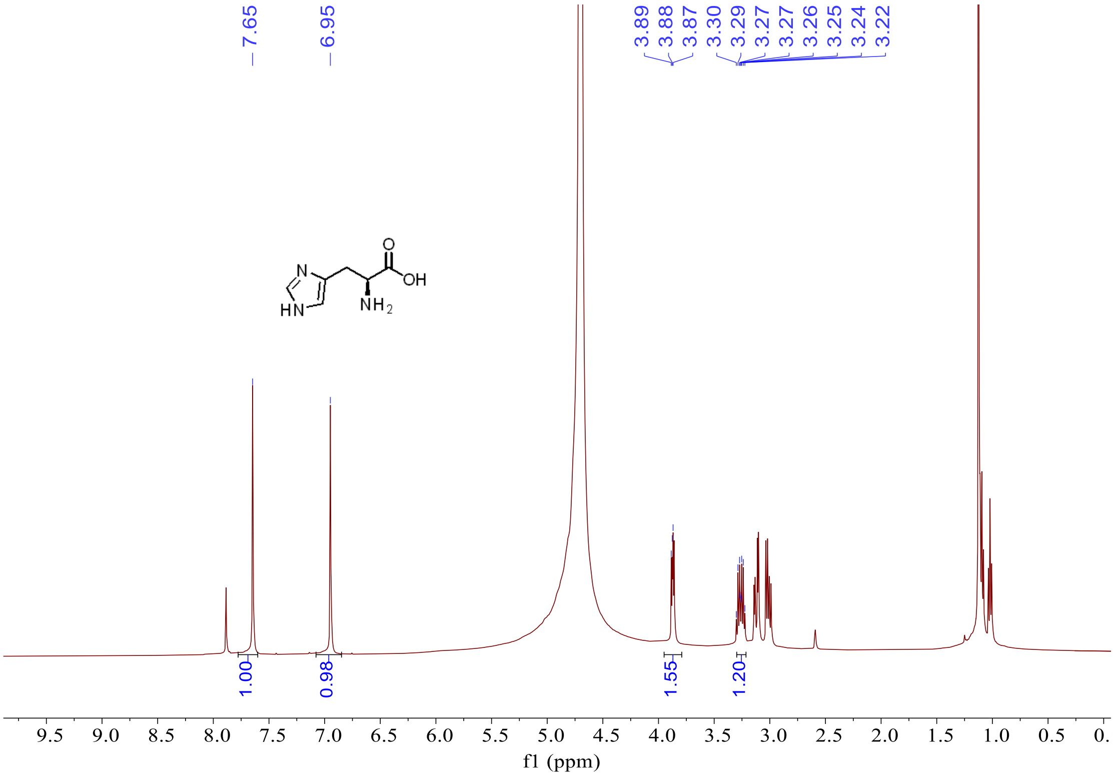

Supplementary Figure 9. ^{1}H NMR spectrum of reaction mixture with bio-additive of *L*-Histidine

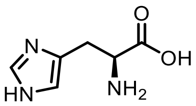

L-Histidine

M = 155.0695

[M-H]$^-$ = 154.0608

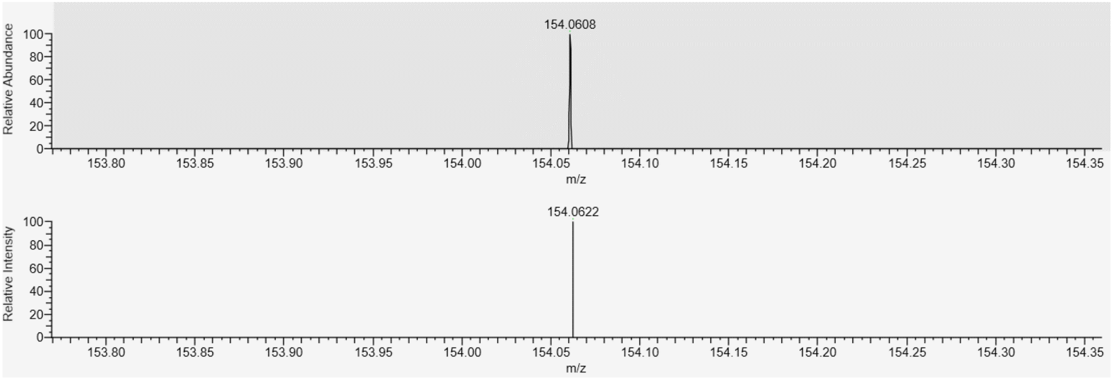

Supplementary Figure 10. HR-MS spectrum of reaction mixture with bio-additive of *L*-Histidine

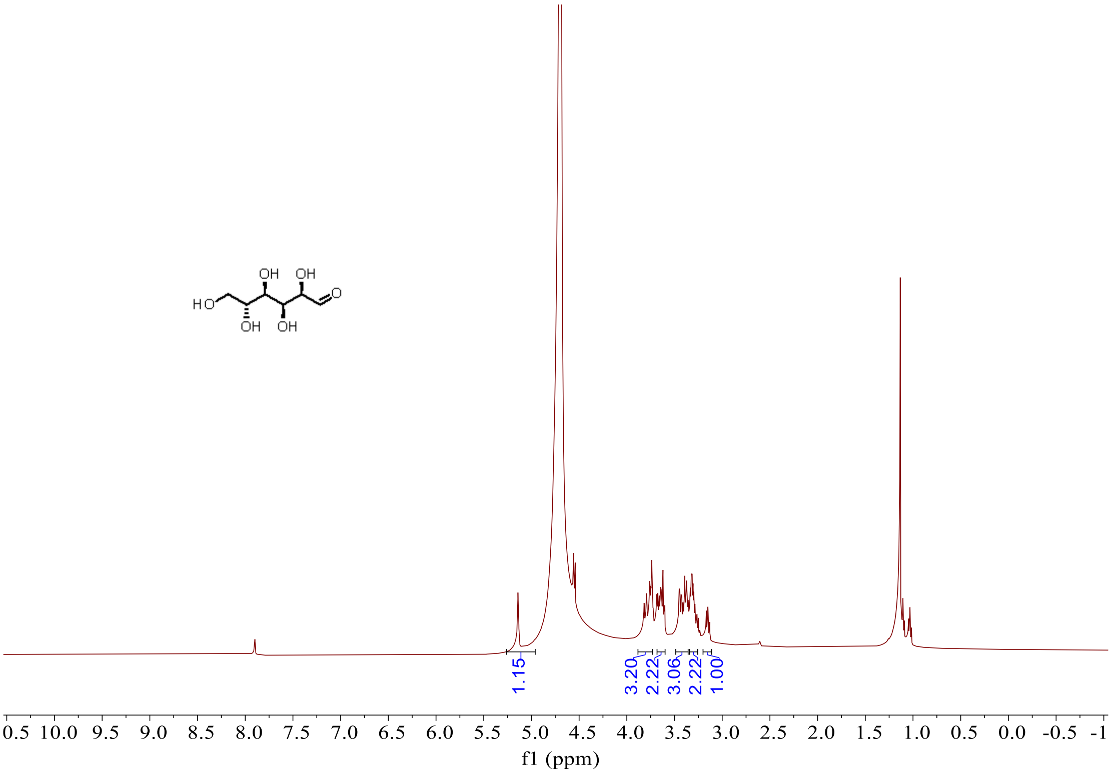

Supplementary Figure 11. ^{1}H NMR spectrum of reaction mixture with bio-additive of *D*(+)-Glucose

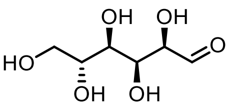

D(+)-Glucose

M = 180.0634

$[M-H]^{-}$ = 179.0548

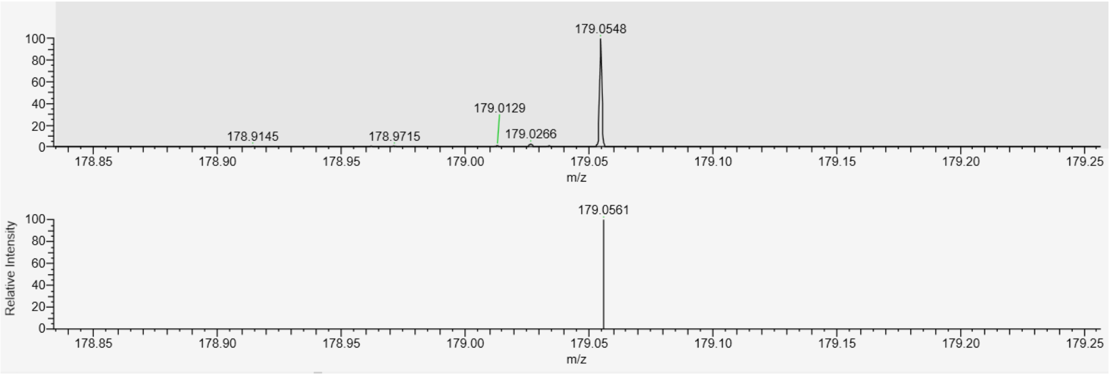

Supplementary Figure 12. HR-MS spectrum of reaction mixture with bio-additive of *D*(+)-Glucose

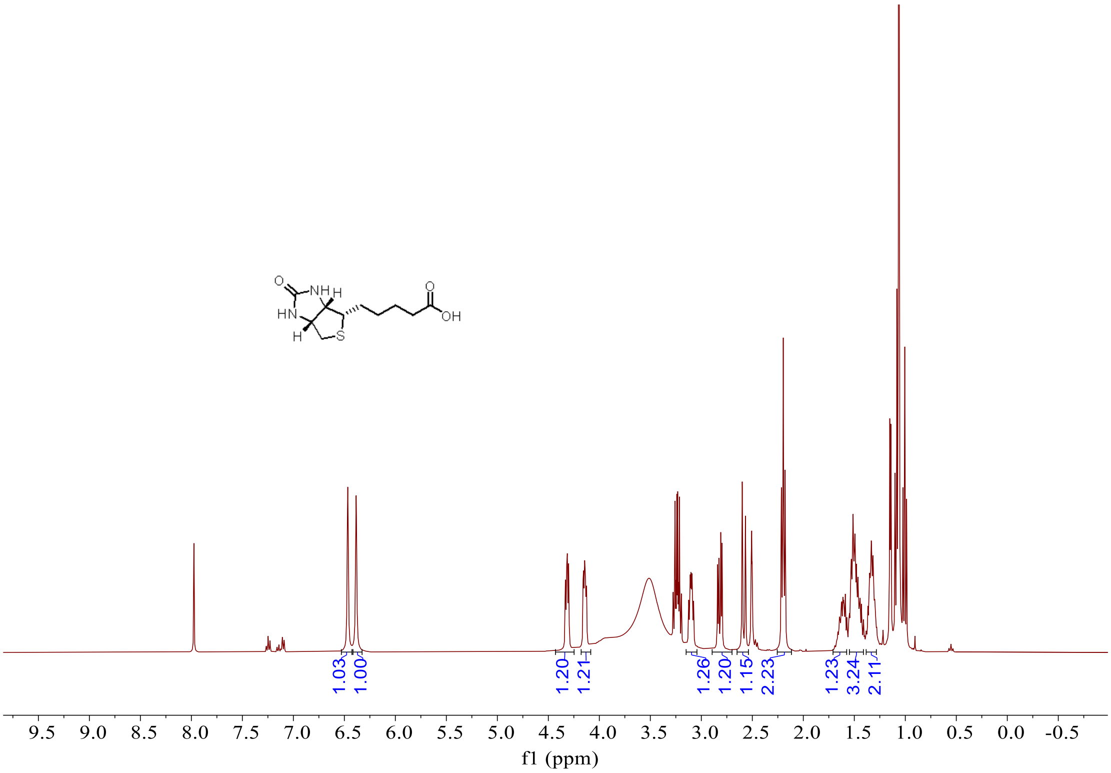

Supplementary Figure 13. ^{1}H NMR spectrum of reaction mixture with bio-additive of *D*-Biotin

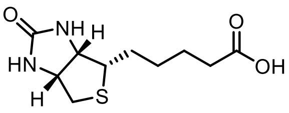

D-Biotin
M = 244.0882
[M-H]$^{-}$ = 243.0804

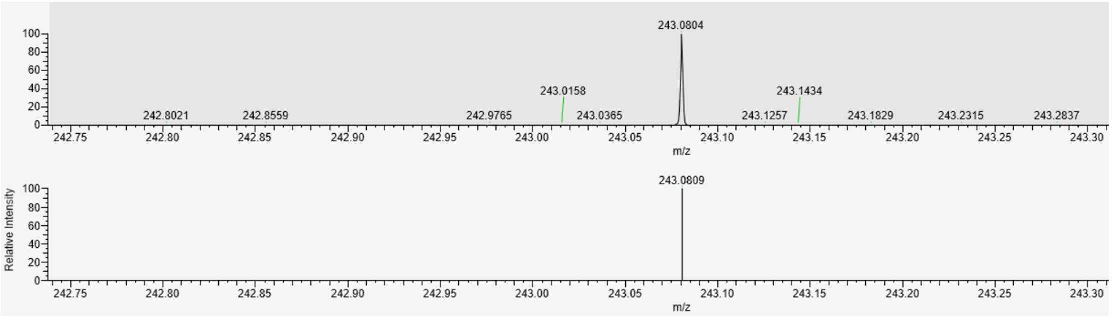

Supplementary Figure 14. HR-MS spectrum of reaction mixture with bio-additive of *D*-Biotin

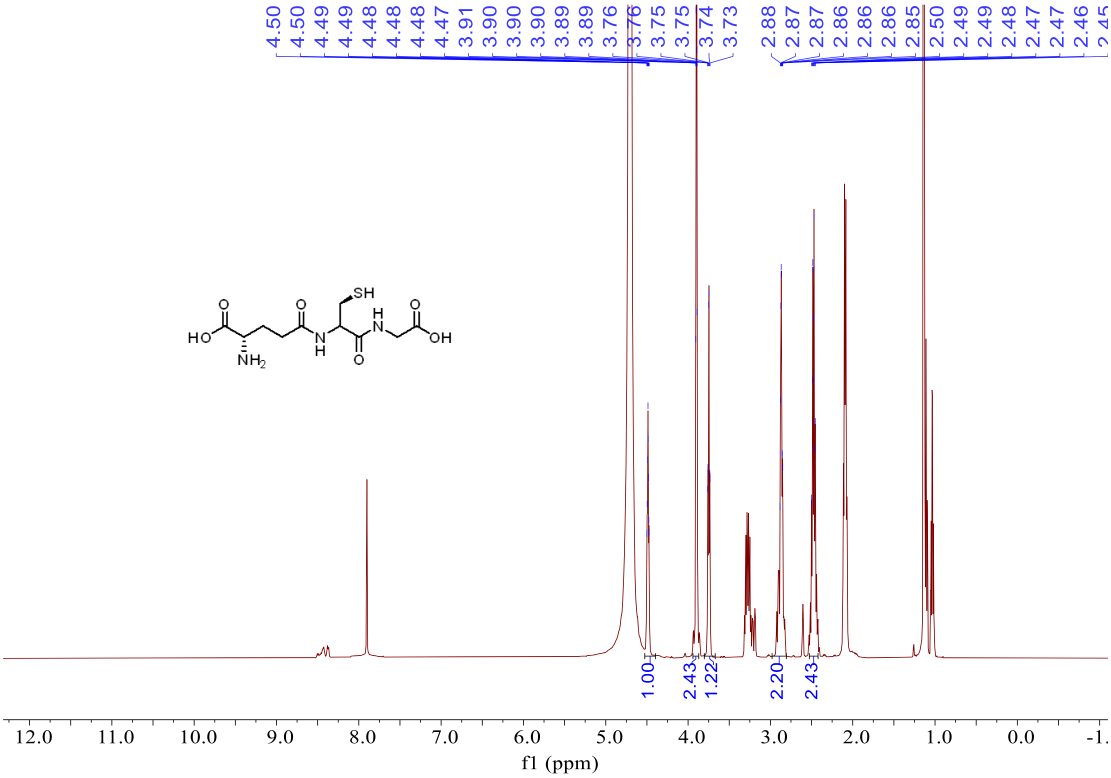

Supplementary Figure 15. ^{1}H NMR spectrum of reaction mixture with bio-additive of Glutathione

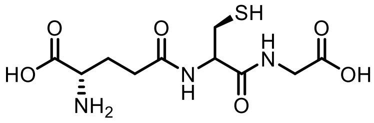

Glutathione

M = 307.0838

$[M\text{-}H]^-$ = 306.0768

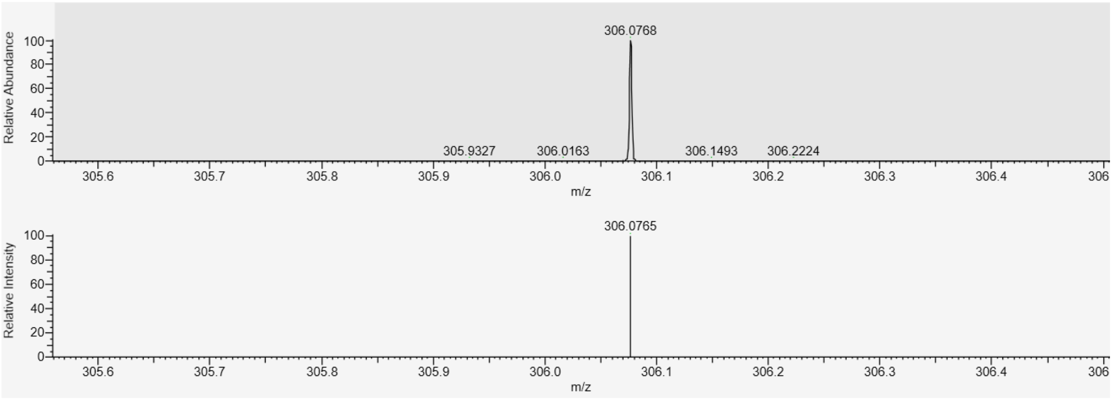

Supplementary Figure 16. HR-MS spectrum of reaction mixture with bio-additive of Glutathione

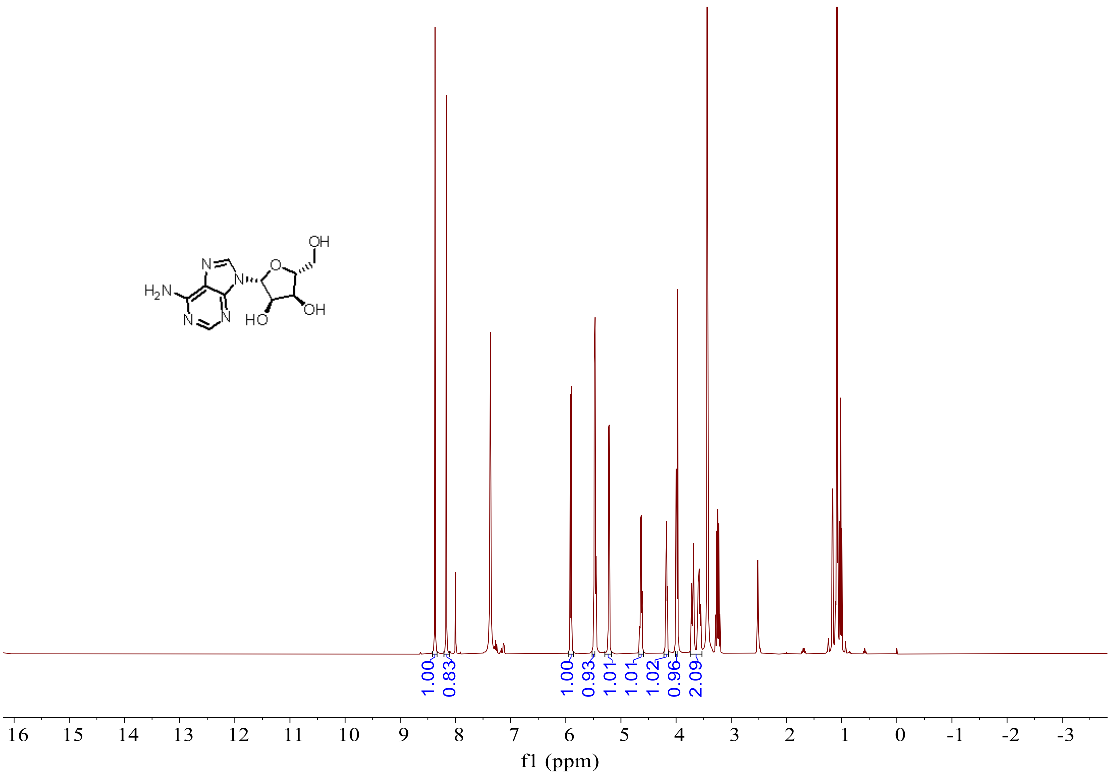

Supplementary Figure 17. ^{1}H NMR spectrum of reaction mixture with bio-additive of Adenosine

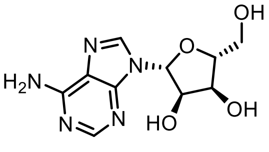

Adenosine

M = 267.0968

$[M\text{-}H]^-$ = 266.0893

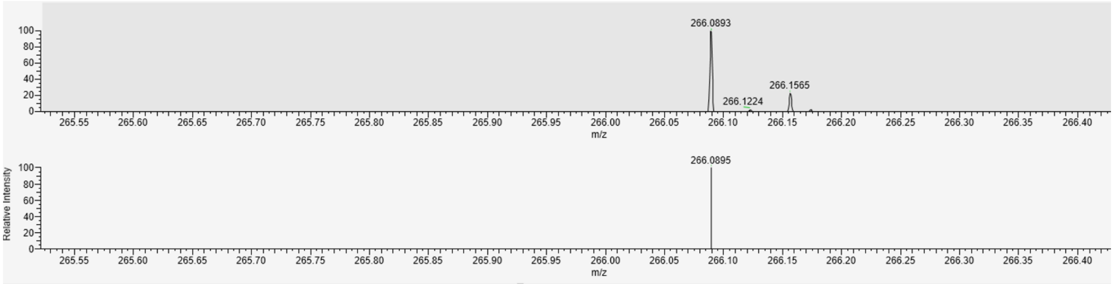

Supplementary Figure 18. HR-MS spectrum of reaction mixture with bio-additive of Adenosine

Supplementary Discussion

Mechanistic Studies

Radical trapping experiments

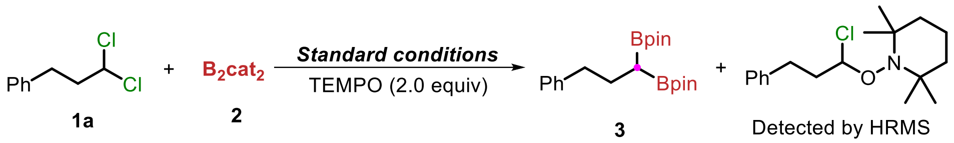

To an oven-dried 10 mL sealed tube, **PC1** (3 mol%, 7.7 mg), (3,3-dichloropropyl)benzene **1a** (0.2 mmol, 37.6 mg), **2** (0.8 mmol, 190 mg, 4 equiv.), 2,2,6,6-tetramethylpiperidinooxy (TEMPO) (2 equiv, 0.4 mmol, 62.4 mg) and DEF (2 mL) were added and the tube was backfilled with argon. The resulting reaction mixture was vigorously stirred under the irradiation of blue LEDs (distance app. 4.0 cm from the bulb) at ambient temperature for 24 h. After the reaction finished, the reaction mixture was analyzed by GC and HRMS.

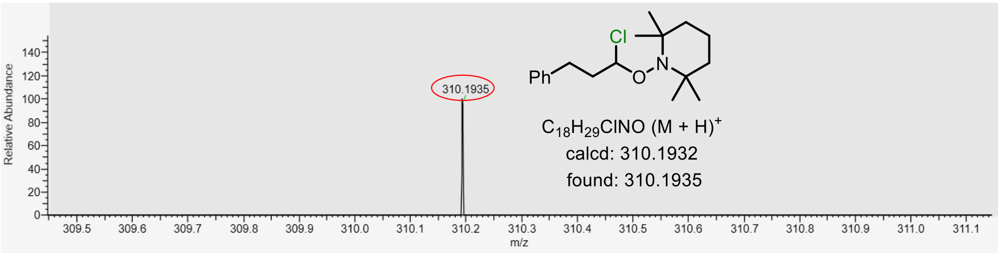

Supplementary Figure 19. The HR-MS result for radical trapping with TEMPO.

No desired *gem*-diborylation product **3** was detected. These results indicated that a radical mechanism might be operative.

Control experiments

1) Evidence for the formation of intermidate *α*-boryl radical

To investigate the possible intermediate of dechlorinative *gem*-diborylation, we performed the following experiments. Firstly, we synthesized this intermediate **74** according to known method, which employed in the model reaction. After the reaction was completed, which give the desired *gem*-diborylation product **3** in 68 % isolated yield.

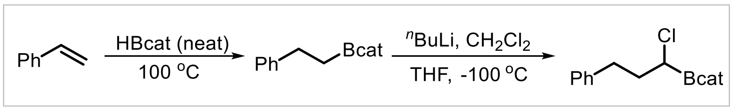

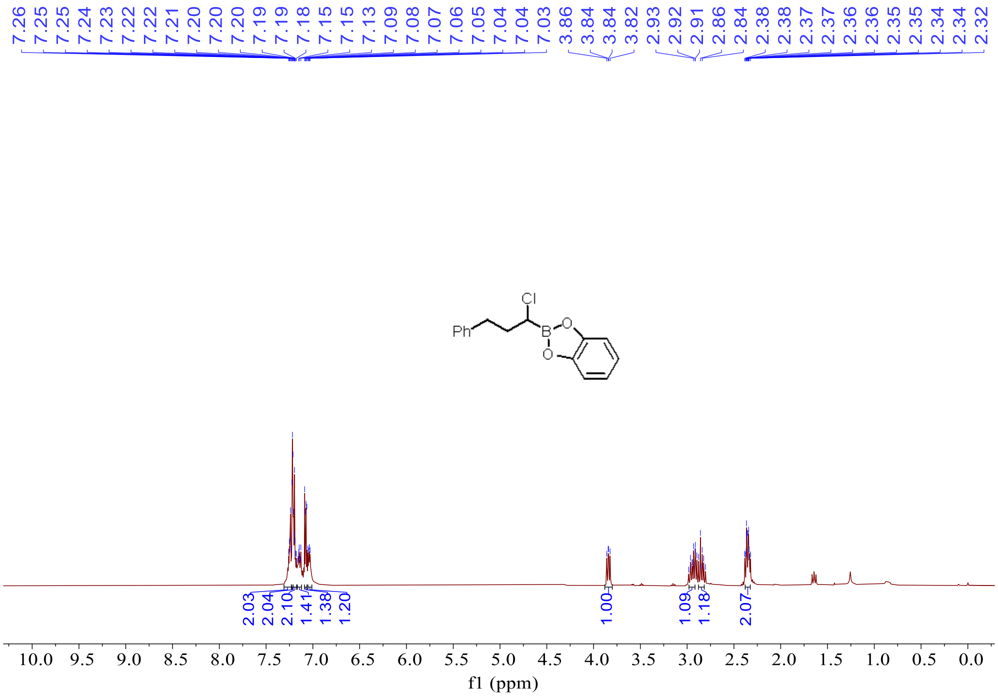

Supplementary Figure 20. **^{1}H NMR** (400 MHz, $CDCl_3$) spectra for compound **Int-74**

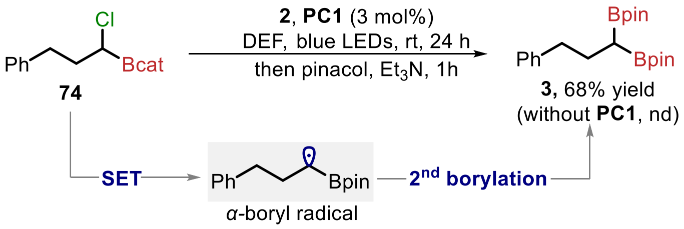

Supplementary Figure 21. The formation of intermidate *α*-boryl radical

According to the results, we speculated the *α*-boryl radical intermediate was formed. Control experiment indicated the **PC1** was essential for this crucial transformation.

2) Evidence for the formation of the hydroborylation reaction

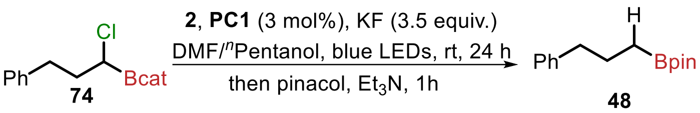

Entry	Deviation from standard conditions	Yield (%)
1	none	60
2	without **PC1**	nr
3	without KF	nd
4	without B_2cat_2	nr

Supplementary Figure 22. Control experiments for the hydroborylation reaction

Control experiments showed that **PC1**, **2** and **KF** were essential for this hydroborylation transformation.

3) Rule out the hydroborylation product 48 from *gem*-diborylation 3

Firstly, we synthesized this intermediate **3'** according to known method. Under standard conditions, we performed the following reaction and the results showed that the hydroborylation product **48** was not formed from **3'**.

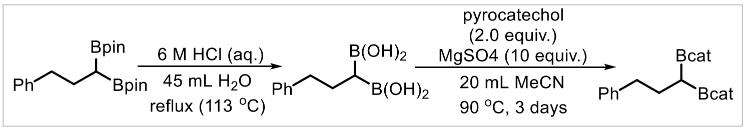

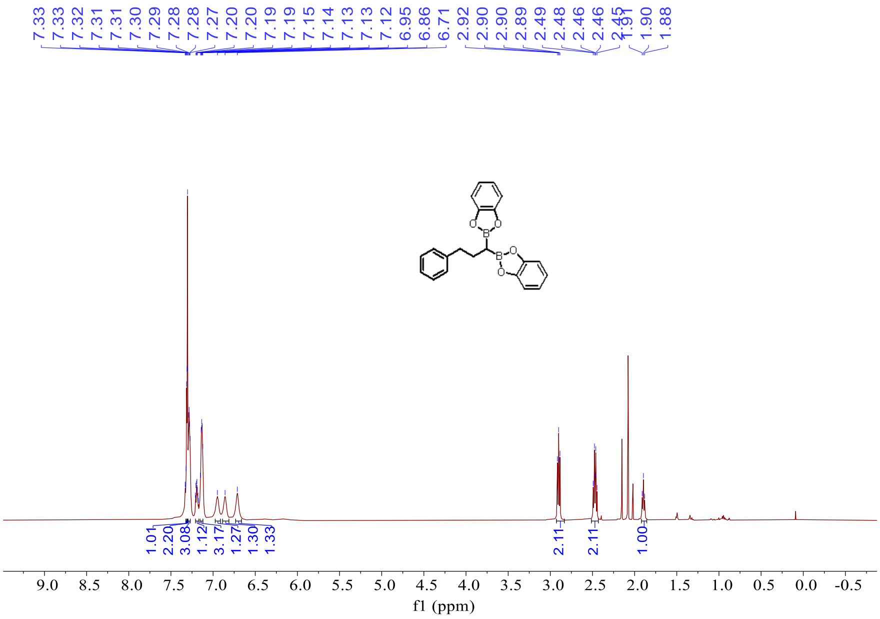

Supplementary Figure 23. **^{1}H NMR** (500 MHz, $CDCl_3$) spectra for compound **Int-3'**

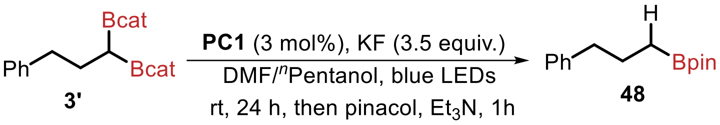

Entry	Deviation from standard conditions	Yield (%)
1	none	nr
2	in the dark	nr
3	without **PC1**	nr

Supplementary Figure 24. Various reaction conditions from **3'** to **48**

Further control experiments indicated either in the dark or under blue light in the presence of **KF** can't finish this transformation.

4) Deuterium labelling experiment

To figure out the hydrogen source of hydroborylation, we performed the deuterium labelling experiments as following produce.

In the glove box, a 4-mL screw-capped vial is charged with **PC1** (3 mol%, 0.006 mmol, 7.7 mg), *gem*-dichloroalkanes **1** (0.2 mmol), B_2cat_2 **2** (0.6 mmol, 142 mg, 3 equiv.), KF (0.7 mmol, 41 mg, 3.5 equiv.). Followed by DMF(*d*-7) (1 mL) was syringed into the reaction tube. The vial was sealed with a cap containing a PTFE septum with silicone seal and removed from the glove box. The resulting reaction mixture is vigorously stirred under the irradiation of blue LEDs (distance app. 4.0 cm from the bulb) at ambient temperature (the fan is used to keep the reaction temperature around ambient temperature) for 24 h. Subsequently, a solution of pinacol (0.8 mmol, 4.0 equiv., 94.4 mg) in triethylamine (0.5 mL) is added to the resulting crude and the reaction mixture is kept stirring at room temperature for another 1 h. Then, saturated brine water (10 mL) is added to the reaction mixture, and the aqueous layer is extracted with ethyl acetate (3 × 5 mL) three times. The organic layers are combined, dried over Na_2SO_4, filtered, and concentrated. The crude residue is directly purified quickly by silica column chromatography (eluted with ethyl acetate/petroleum ether) to yield the product **48-D**.

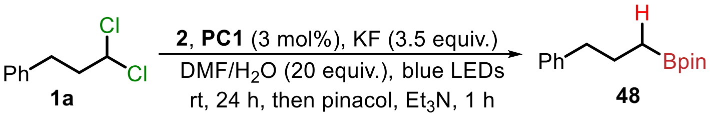

Entry	Deviation from standard conditions	Yield (%)	D (%)
1	none	63	nd
2	DMF/D_2O was used	55	<5
3	DMF(d-7)/H_2O was used	50	<5
4	DMF(d-7) was used	54	>95

Supplementary Figure 25. Identification of hydrogen source for hydroborylation

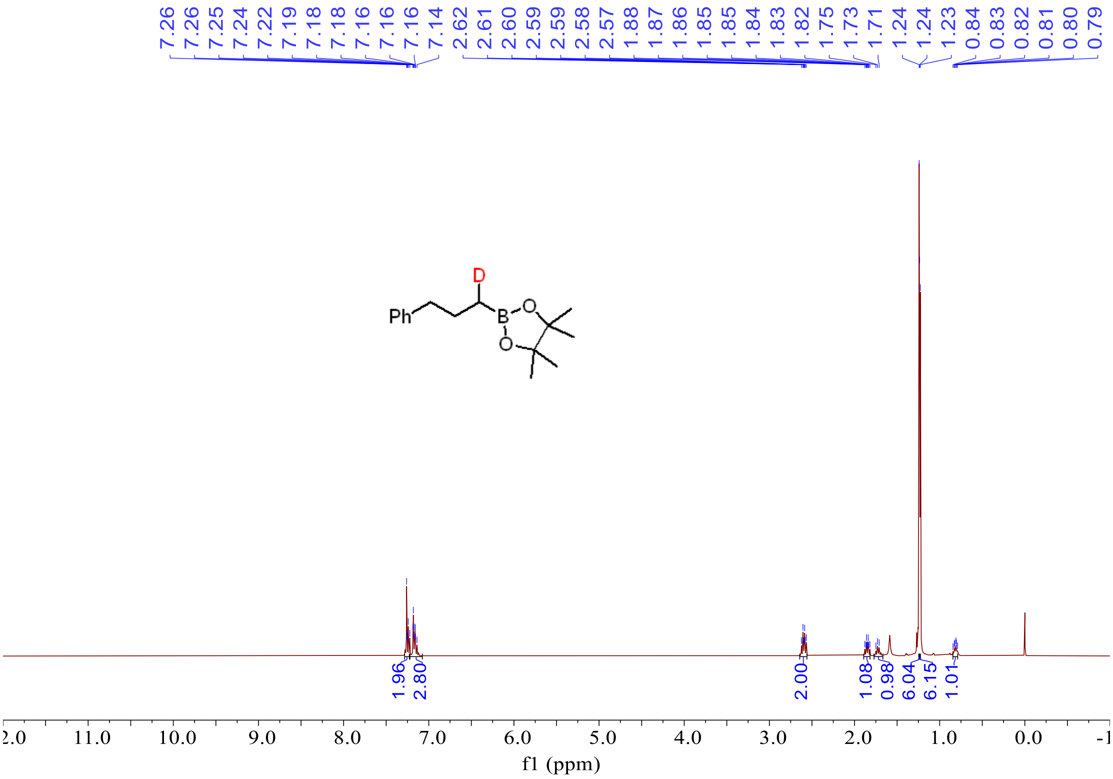

Supplementary Figure 26. ^{1}H NMR (400 MHz, $CDCl_3$) spectra for compound **48-D**

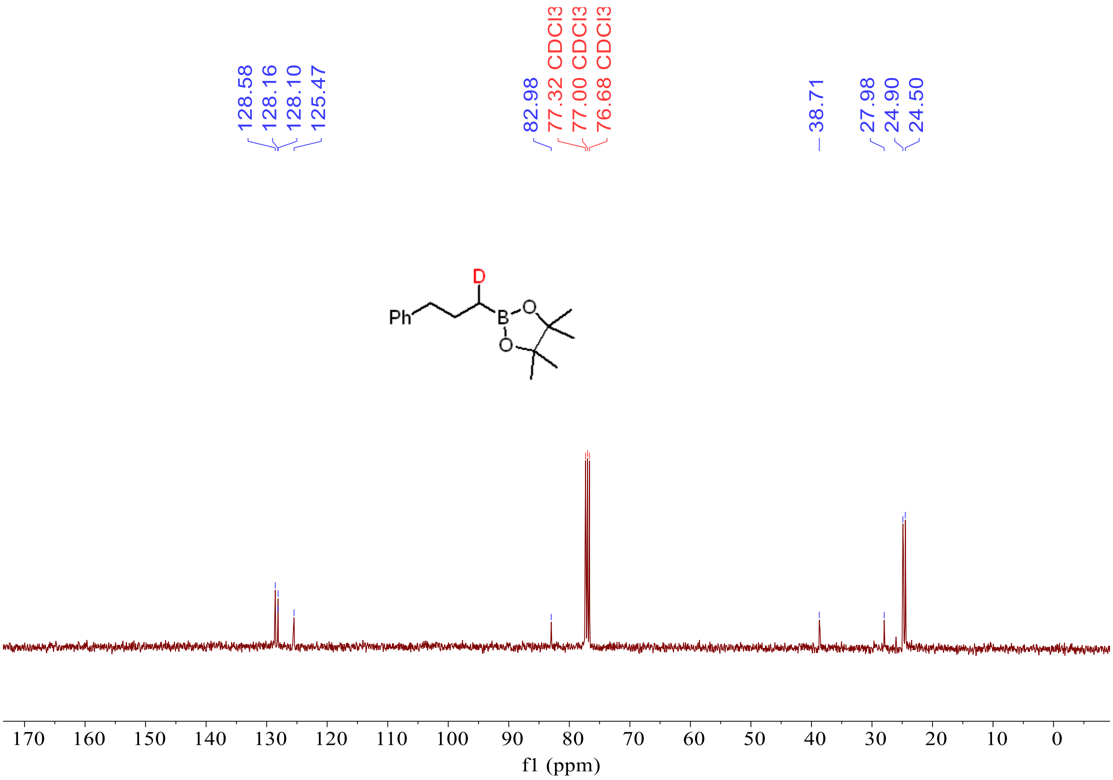

Supplementary Figure 27. ^{13}C NMR (400 MHz, $CDCl_3$) spectra for compound **48-D**

Control experiments indicated the proton source is mainly from DMF in the hydroborylation reaction.

UV-Vis spectroscopic analysis

We decided to investigate ground state interactions between the reactants in our mixture by using UV–vis absorption spectroscopy. The solvent DEF is degassed with a stream of argon for 1 h. First, **1a** (0.1 mmol, 18.8 mg), **2** (0.4 mmol, 95 mg), **PC1** (0.003 mmol, 3.9 mg) were added into a 25 mL volumetric flask for constant volume, respectively. Pure DEF solution absorption is subtracted as background. In particular, we started by recording an absorption spectrum of single components (**PC1**, **1a**, **2**,) and that of a mixture of **1a** and **PC1** with **2**, respectively.

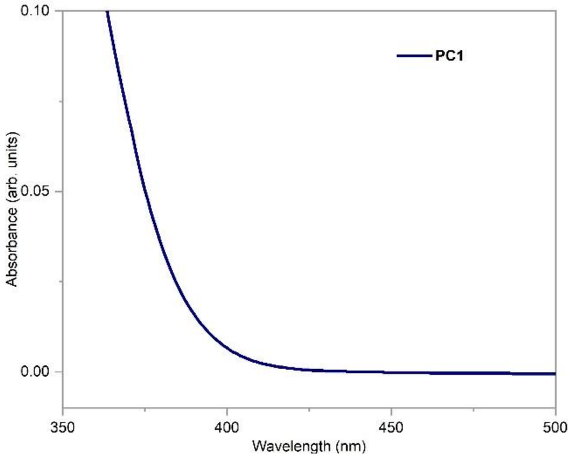

Supplementary Figure 28. Absorption spectra of **PC1** in DEF. All spectra were recorded in quartz cuvettes (optical path: 1 cm) with a bandwidth of 5 nm and a data pitch of 1 nm. Scan rate: medium.

From the above spectra, the **PC1** showcased a weak absorption in the visible light region (blue LEDs λ_{max} = 466 nm).

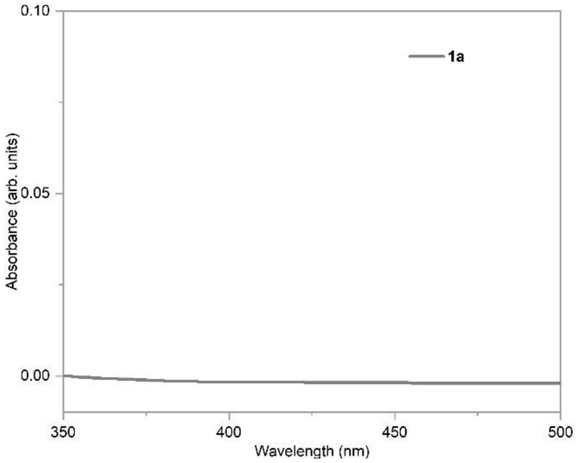

Supplementary Figure 29. Absorption spectra of **1a** in DEF. All spectra were recorded in quartz cuvettes (optical path: 1 cm) with a bandwidth of 5 nm and a data pitch of 1 nm. Scan rate: medium

From the above spectra, **1a** showed no absorption in the visible light region (blue LEDs λ_{max} = 466 nm).

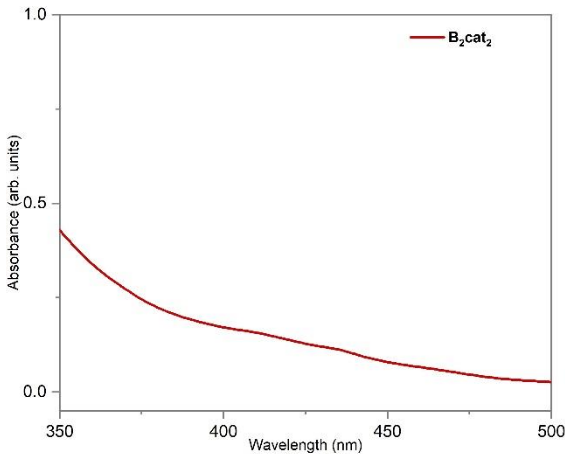

Supplementary Figure 30. Absorption spectra of **2** in DEF. All spectra were recorded in quartz cuvettes (optical path: 1 cm) with a bandwidth of 5 nm and a data pitch of 1 nm. Scan rate: medium.

From the above spectra, **2** showed strong absorption in the visible light region (blue LEDs λ_{max} = 466 nm).

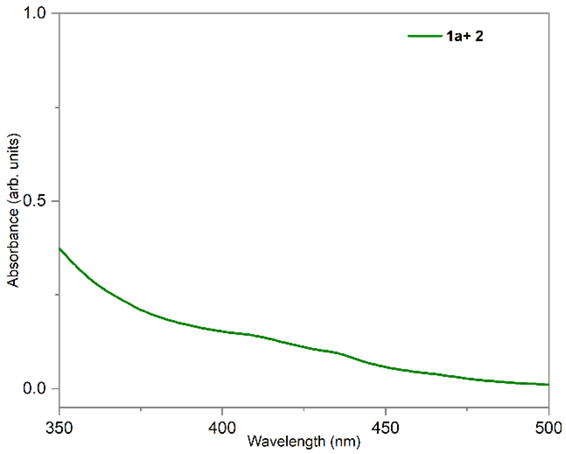

Supplementary Figure 31. Absorption spectra of **1a** and **2** in DEF. All spectra were recorded in quartz cuvettes (optical path: 1 cm) with a bandwidth of 5 nm and a data pitch of 1 nm. Scan rate: medium.

From the above spectra, **1a** and **2** showed strong absorption in the visible light region, which has no difference from **2**'s absorption alone (blue LEDs λ_{max} = 466 nm).

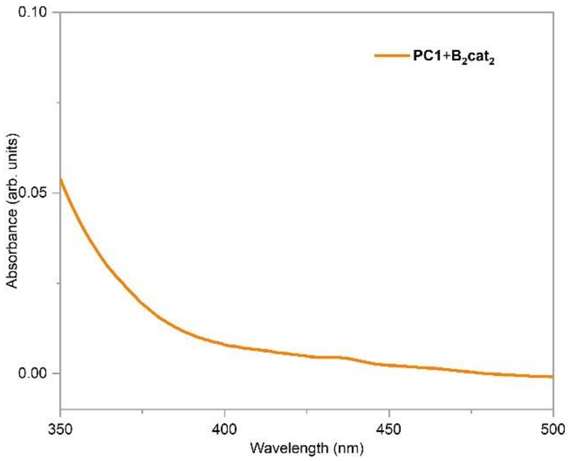

Supplementary Figure 32. Absorption spectra of **PC1** and **2** in DEF. All spectra were recorded in quartz cuvettes (optical path: 1 cm) with a bandwidth of 5 nm and a data pitch of 1 nm. Scan rate: medium.

From the above spectra, it is found a slightly shift appearance and we speculate that a weakly bound **PC1**/**2** complex might be formed to enhance its absorption under irradiation by blue LEDs.

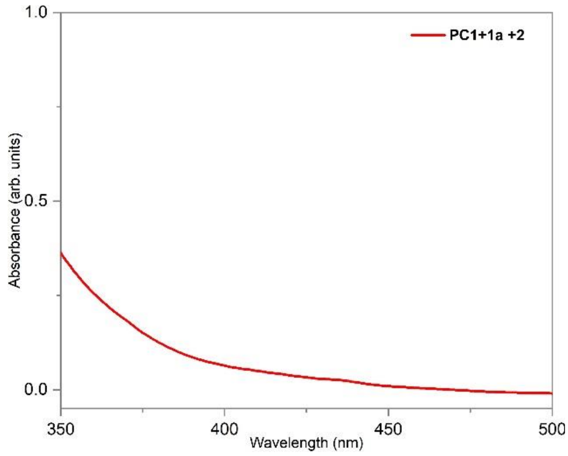

Supplementary Figure 33. Absorption spectra of **PC1**, **1a** and **2** in DEF. All spectra were recorded in quartz cuvettes (optical path: 1 cm) with a bandwidth of 5 nm and a data pitch of 1 nm. Scan rate: medium.

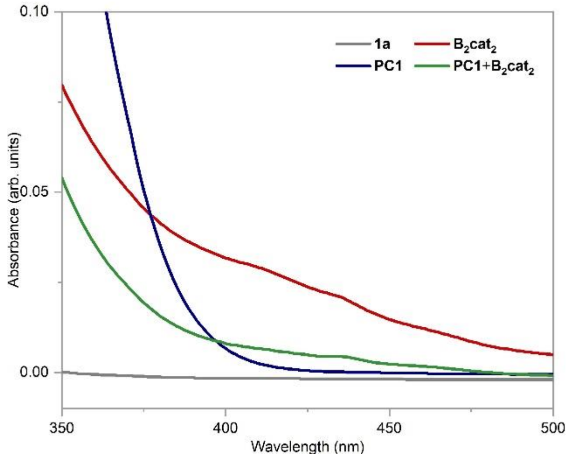

Supplementary Figure 34. UV-vis absorption spectra in DEF

From the above spectra, the mixture system of **PC1**, **1a** and **2** has strong absorption in the visible light region, which indicated the weak interaction between **PC1** and **DEF-ligated B_2cat_2**.

To further investigate ground state interactions between the **PC1** and **2** in reaction mixture, we underwent the control experiments by using of UV–vis absorption spectroscopy. The solvent DCM is degassed with a stream of argon for 1 h., **2** (0.4 mmol, 95 mg), **PC1** (0.003 mmol, 3.9 mg) and their mixture were added into a 25 mL volumetric flask for constant volume, respectively. Pure DCM solution absorption is subtracted as background. In particular, we started by recording an absorption spectrum of single components (**PC1**, **2**,) and that of a mixture of **2** and **PC1**, respectively.

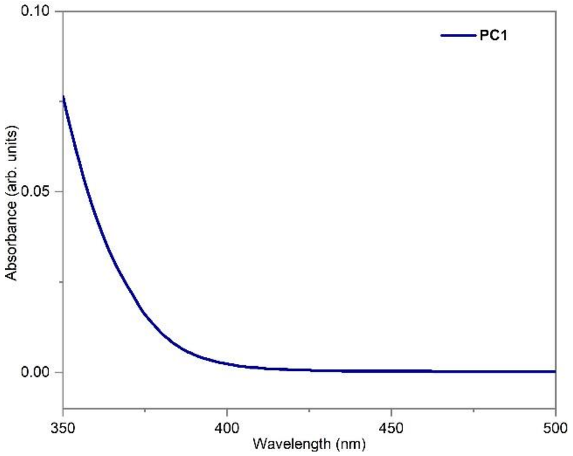

Supplementary Figure 35. Absorption spectra of **PC1** in DCM. All spectra were recorded in quartz cuvettes (optical path: 1 cm) with a bandwidth of 5 nm and a data pitch of 1 nm. Scan rate: medium.

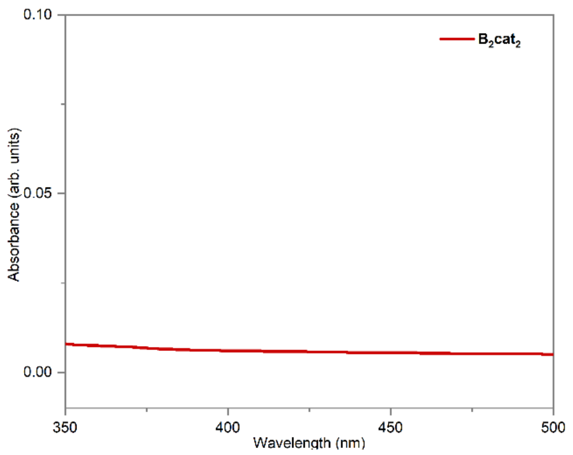

Supplementary Figure 36. Absorption spectra of **2** in DCM. All spectra were recorded in quartz cuvettes (optical path: 1 cm) with a bandwidth of 5 nm and a data pitch of 1 nm. Scan rate: medium.

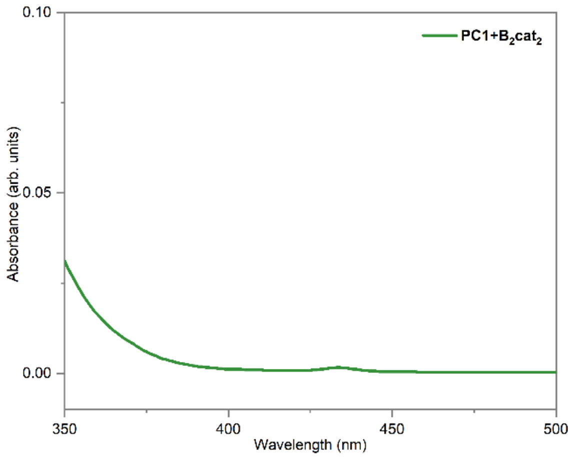

Supplementary Figure 37. Absorption spectra of **PC1** and **2** in DCM. All spectra were recorded in quartz cuvettes (optical path: 1 cm) with a bandwidth of 5 nm and a data pitch of 1 nm. Scan rate: medium.

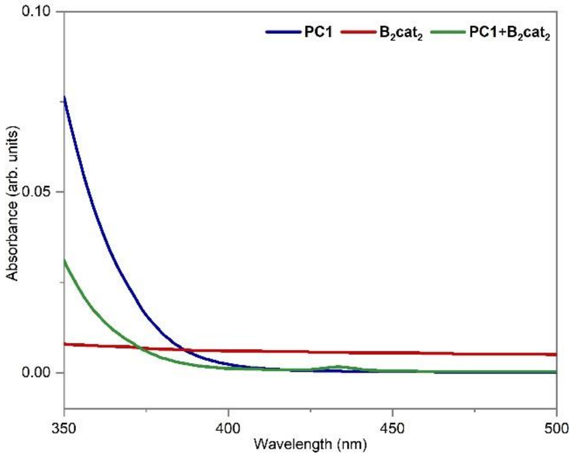

Supplementary Figure 38. UV-vis absorption spectra in DCM

From the above spectra, the mixture system of **PC1** and **2** in **DCM** has no obvious absorption and difference in a single system under visible light region, which could further showcase no interaction between **PC1** and **2**.

^{31}P NMR studies

To investigate the interactions between the reactants in our mixture, we performed the following experiments by using of NMR tracking.

1) Add **PC1** (0.003 mmol, 3.9 mg) and 0.5 mL CD_3CN to a 4 mL sample bottle and stir for 30 mins, then the sample was detected by ^{31}P NMR spectra, using Ph_3P as external standard.

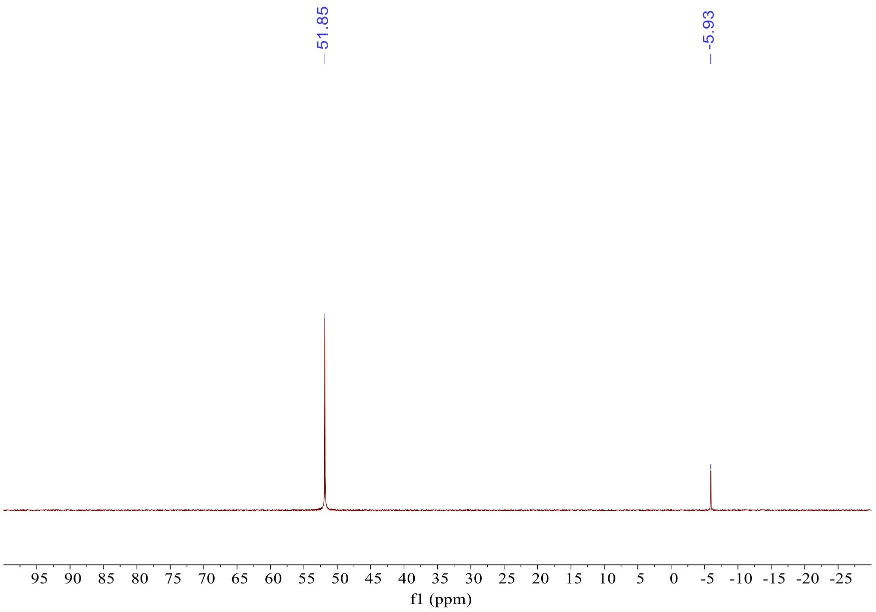

Supplementary Figure 39. ^{31}P NMR spectra of the mixture of **PC1** in CD_3CN

From the above spectra, peak at δ = 51.85 ppm can be attributed to **PC1**.

2) Add **PC1** (0.003 mmol, 3.9 mg), DEF (1 mL) and 0.5 mL CD_3CN to a 4 mL sample bottle and stir for 30 mins, then the sample was detected by ^{31}P NMR spectra, using Ph_3P as external standard.

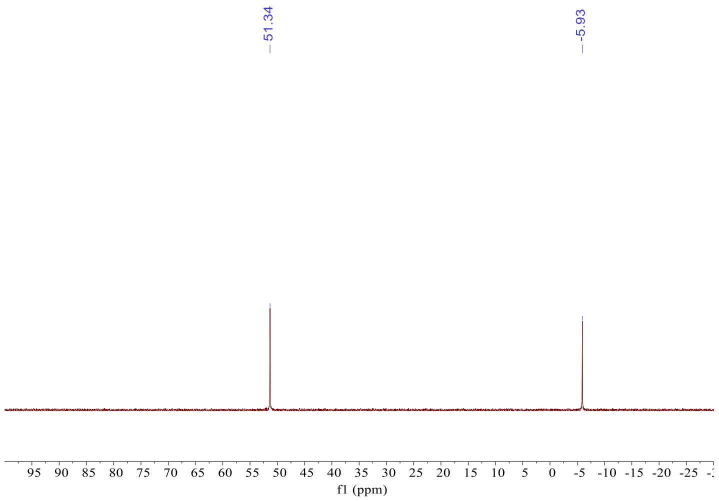

Supplementary Figure 40. ^{31}P NMR spectra of the mixture of **PC1** and DEF in CD_3CN

From the above spectra, peak at δ = 51.34 ppm can be attributed to **PC1**, which suggested there is no interaction of **PC1** and DEF.

3) Add **PC1** (0.003 mmol, 3.9 mg), **2** (0.2 mmol, 47 mg) and 1 mL CD_3CN to a 4 mL sample bottle and stir for 30 mins, then the sample was detected by ^{31}P NMR spectra, using Ph_3P as external standard.

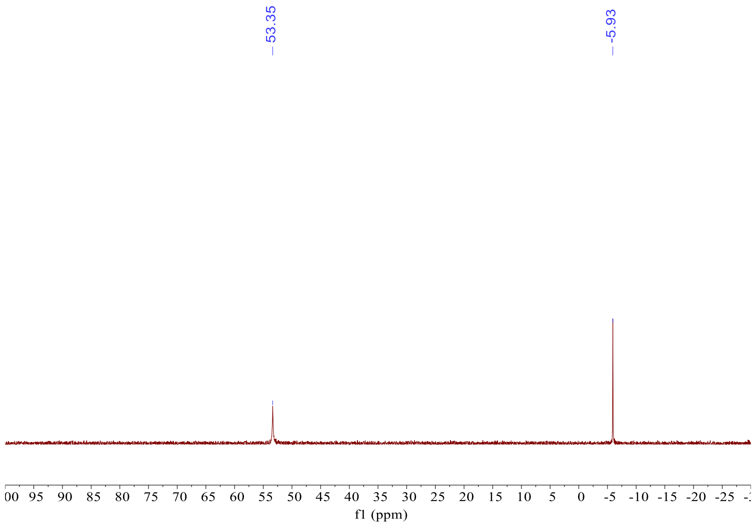

Supplementary Figure 41. ^{31}P NMR spectra of the mixture of **PC1** and **2** in CD_3CN

From the above spectra, the chemical shifts of the ^{31}P spectra are shifted to lower fields (δ = 53.35 ppm), indicating the weak interaction between **PC1** and **2**.

4) Add **PC1** (0.003 mmol, 3.9 mg), **2** (0.2 mmol, 47 mg), DEF (1 mL) and 0.5 mL CD_3CN to a 4 mL sample bottle and stir for 30 mins, then the sample was detected by ^{31}P NMR spectra, using Ph_3P as external standard.

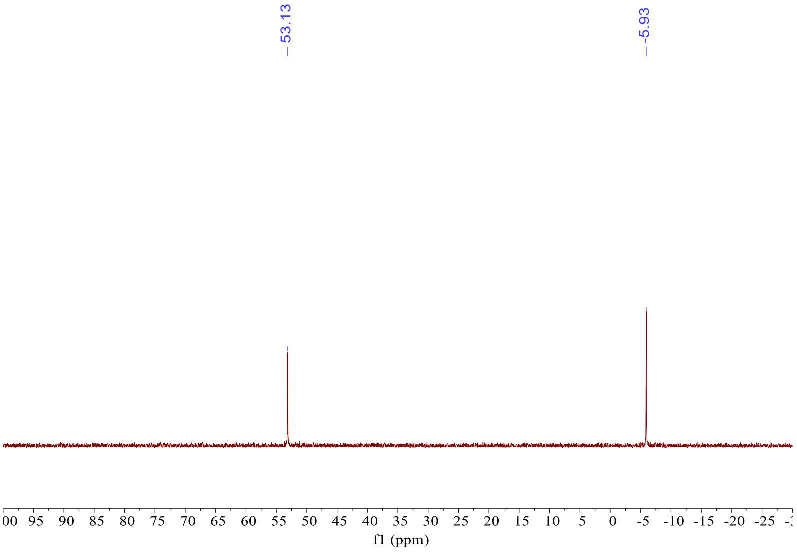

Supplementary Figure 42. ^{31}P NMR spectra of the mixture of **PC1**, **2** and DEF in CD_3CN

From the above spectra, the chemical shifts of the ^{31}P spectra are shifted to lower fields (δ = 53.13 ppm), indicating the weak interaction between **PC1** and **DEF-ligated B_2cat_2**.

^{11}B NMR studies

^{11}B NMR study on gold catalyzed dechlorinative *gem*-diborylation

1) Add **2** (0.2 mmol, 47 mg) and 1 mL $CDCl_3$ to a 4 mL sample bottle and stir for 30 mins, then the sample was detected by ^{11}B NMR spectra.

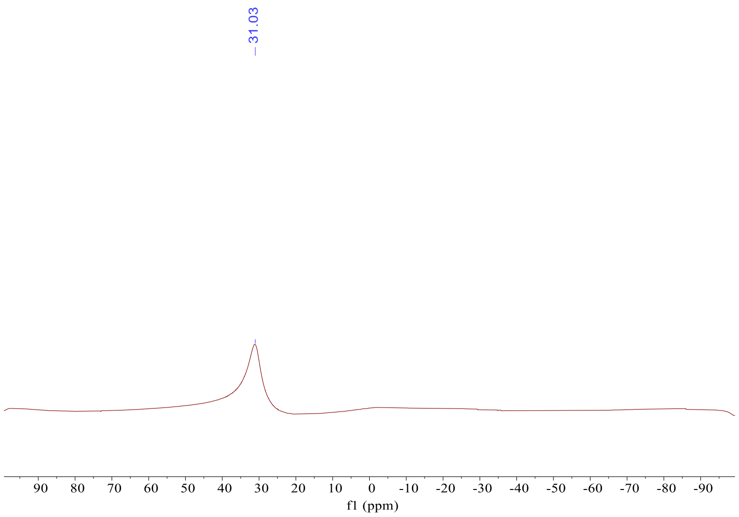

Supplementary Figure 43. ^{11}B NMR spectra of the mixture of **2** at room temperature in $CDCl_3$

From the above spectra, peak at δ = 31.03 ppm can be attributed to B_2cat_2 (**2**).

2) Add **2** (0.2 mmol, 47 mg), DEF (0.5 mL) and 1 mL $CDCl_3$ to a 4 mL sample bottle and stir for 30 mins, then the sample was detected by ^{11}B NMR spectra.

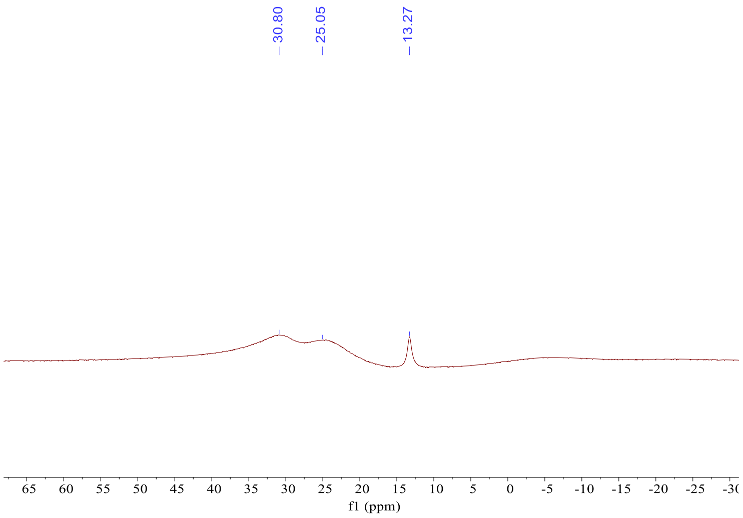

Supplementary Figure 44. ^{11}B NMR spectra of the mixture of **2** and DEF in $CDCl_3$

From the above spectra, peak at δ = 25.05 ppm can be attributed to the formation of DEF-ligated B_2cat_2 (**B1**) and Peak at δ = 13.27 ppm can be attributed to the formation bis(catecholato) boronate (**B2**), as described previously[13]. The above results suggest a weak interaction between **2** and DEF.

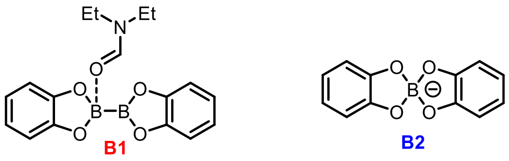

3) Add **2** (0.2 mmol, 47 mg), DEF (0.5 mL), **PC1** (3.9 mg) and 1 mL $CDCl_3$ to a 4 mL sample bottle and stir for 30 mins, then the sample was detected by ^{11}B NMR spectra.

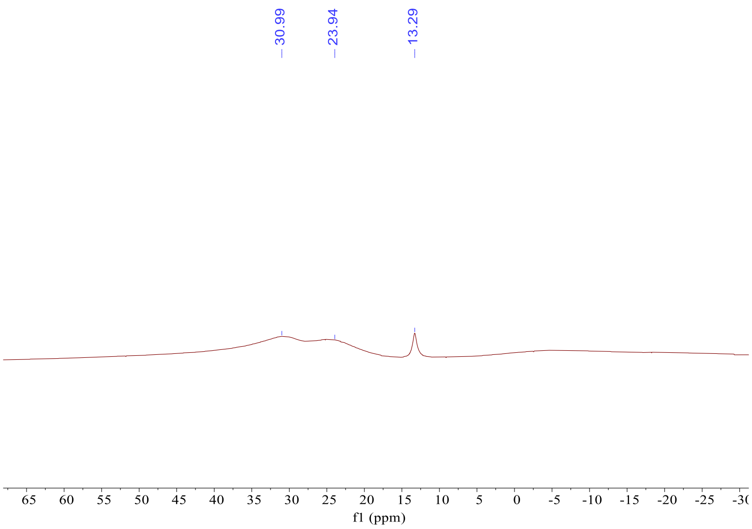

Supplementary Figure 45. ^{11}B NMR spectra of the mixture of **2**, **PC1** and DEF in $CDCl_3$

From the above spectra, peak at δ = 23.94 ppm can be attributed to the formation of DEF-ligated B_2cat_2 (**B1**) and the chemical shift changed, which influenced by **PC1**. Peak at δ = 13.29 ppm can be attributed to the formation bis(catecholato) boronate (**B2**). The above results suggest a weak interaction between **PC1** and **DEF-ligated B_2cat_2**.

4) Add **2** and DEF with different mixture ratio with 0.5 mL $CDCl_3$ to a 4 mL sample bottle and stir for 30 mins, then the sample was detected by ^{11}B NMR spectra.

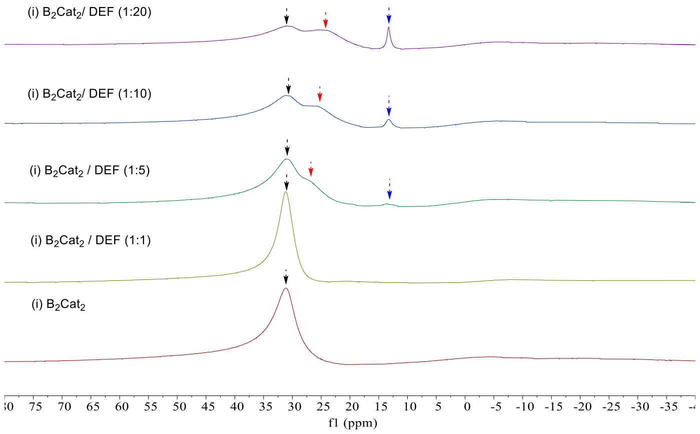

Supplementary Figure 46. ^{11}B NMR spectra of the different ratio mixture of **2** and DEF in $CDCl_3$

From the above spectra, the peak of the formation of DEF-ligated B_2cat_2 (**B1**) (red arrow) and the formation bis(catecholato) boronate (**B2**) (blue arrow). With the increase of the proportion of DEF, the formation of complex **B1** and **B2** correspondingly increases, which indicates that there is a certain complexation between **2** and DEF.

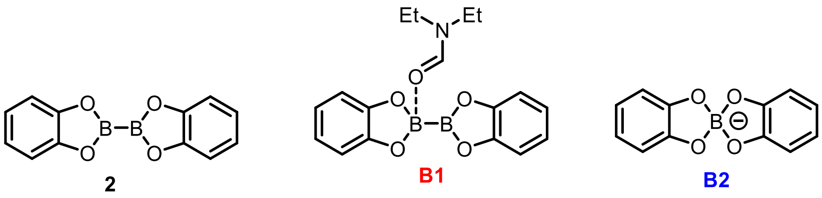

^{11}B NMR study on gold catalyzed dechlorinative hydroborylation

1) Add **2** (0.2 mmol, 47 mg) and 1 mL $CDCl_3$ to a 4 mL sample bottle and stir for 30 mins, then the sample was detected by ^{11}B NMR spectra, using MeOBpin as external standard.

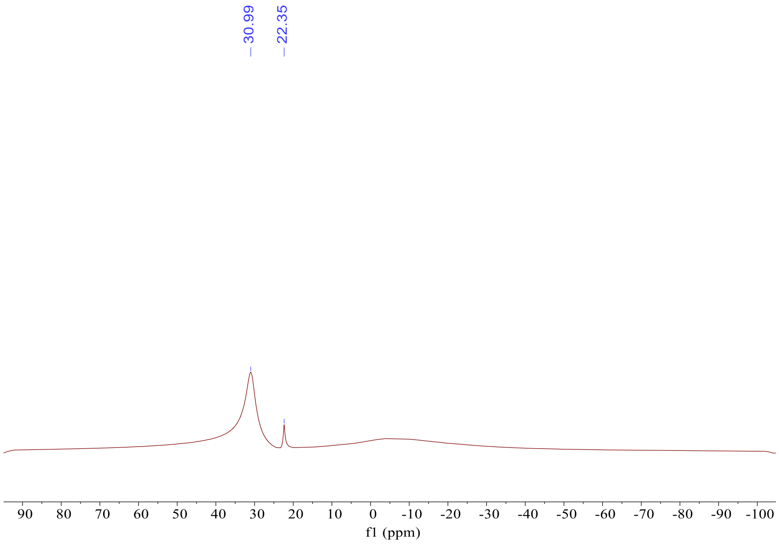

Supplementary Figure 47. ^{11}B NMR spectra of **2** in $CDCl_3$

From the above spectra, peak at δ = 30.93 ppm can be attributed to B_2cat_2 (**2**), which suggested the same as the previous results.

2) Add **2** (0.2 mmol, 47 mg), KF (0.2 mmol, 12 mg) and 1 mL $CDCl_3$ to a 4 mL sample bottle and stir for 30 mins, then the sample was detected by ^{11}B NMR spectra, using MeOBpin as external standard.

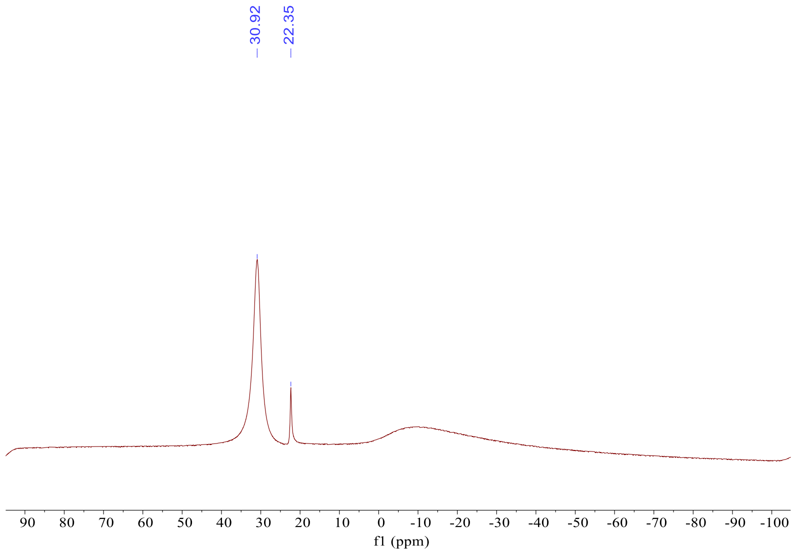

Supplementary Figure 48.^{11}B NMR spectra of the mixture of **2** and KF in $CDCl_3$

From the above spectra, peak at δ = 30.92 ppm can be attributed to B_2cat_2, which suggested that there is no interaction between B_2cat_2 and KF.

3) Add **2** (0.2 mmol, 47 mg), KF (0.2 mmol, 12 mg), DMF/^{n}Pentol (1:1, 0.5 mL) and 1 mL $CDCl_3$ to a 4 mL sample bottle and stir for 30 mins, then the sample was detected by ^{11}B NMR spectra, using MeOBpin as external standard.

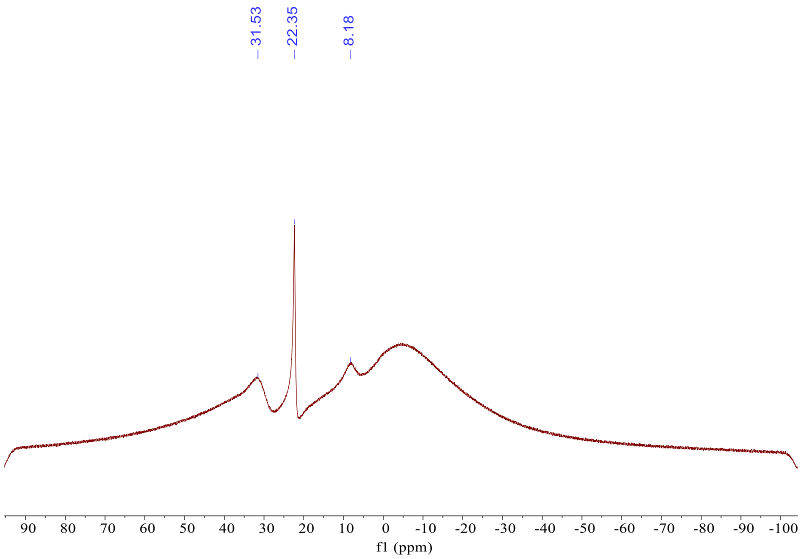

Supplementary Figure 49.^{11}B NMR spectra of the mixture of **2**, KF and DMF/^{n}Pentol at room temperature in $CDCl_3$

From the above spectra, peak at δ = 31.53 ppm can be attributed to DMF-ligated B_2cat_2 (**B3**) and the chemical shift changed, which influenced by KF. A small amount of a new boron "ate" complex (δ = 8.18 ppm) can be attributed to B_2cat_2•KF adduct (**B4**), which was proposed in literature[14].

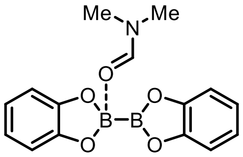

B3

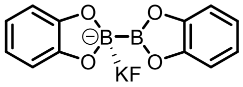

B4

4) Add **2** (0.2 mmol, 47 mg), KF (0.2 mmol, 12 mg), DMF/^{n}Pentol (1:1, 0.5 mL), **PC1** (3.9 mg) and 1 mL $CDCl_3$ to a 4 mL sample bottle and stir for 30 mins, then the sample was detected by ^{11}B NMR spectra, using MeOBpin as external standard.

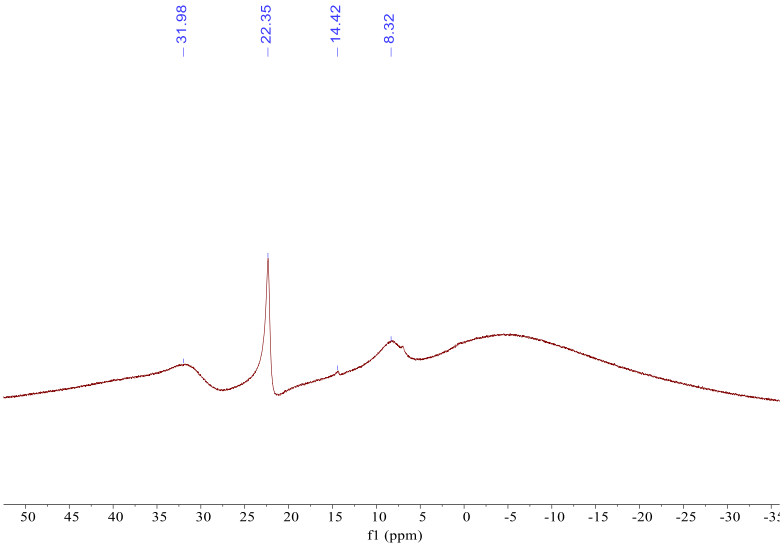

Supplementary Figure 50.^{11}B NMR spectra of the mixture of **2**, KF, **PC1** and DMF/^{n}Pentol in $CDCl_3$

From the above spectra, peak at δ = 31.98 ppm can be attributed to DMF-ligated B_2cat_2 (**B3**) and the chemical shift changed, which influenced by PC1. Peak at δ = 14.42 ppm can be attributed to the formation bis(catecholato) boronate (**B2**). Peak at δ = 8.32 ppm can be attributed to B_2cat_2•KF adduct (**B4**). The above results suggest a weak interaction between **2**, **PC1** and **DMF**.

Cyclic Voltammetry Experiments

Cyclic voltammetry was performed in a three-electrode cell connected to a schlenk line under argon at room temperature. A cyclic voltammograms in Solvent (10 mL) by using glassy carbon as the working electrode, Pt wire as the counter electrode and $Ag/AgNO_3$ as the reference electrode. The scan rate was 100 mV/s, ranging from 0 V to 2.0 V or 0 V to -2.0 V. $^{n}Bu_4NPF_6$ (387 mg, 1.0 mmol) was used as the electrolyte.

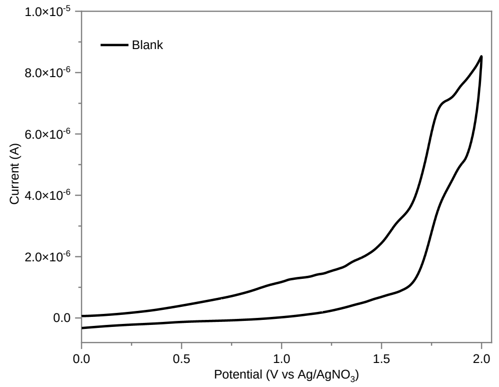

Supplementary Figure 51. The CV data of Blank. Test conditions: $^{n}Bu_4NPF_6$ (1.0 mmol, 387 mg) in MeCN (10 mL).

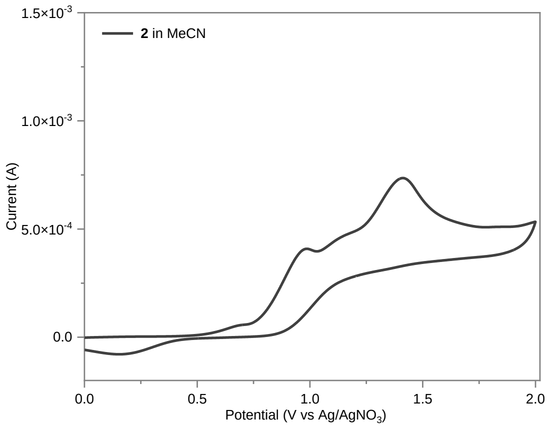

Supplementary Figure 52. The CV data of **2** in MeCN. Test conditions: $^{n}Bu_4NPF_6$ (1.0 mmol, 387 mg), 2 (0.1 mmol, 24 mg) in MeCN (10 mL).

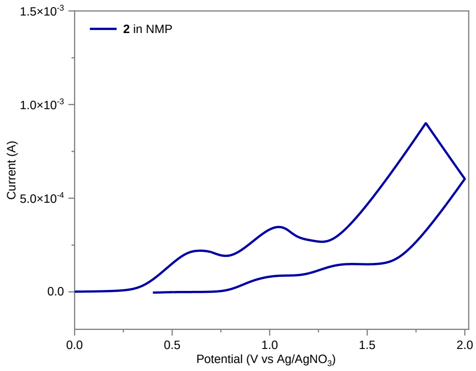

Supplementary Figure 53. The CV data of **2** in NMP. Test conditions: $^{n}Bu_4NPF_6$ (1.0 mmol, 387 mg), 2 (0.1 mmol, 24 mg) in NMP (10 mL).

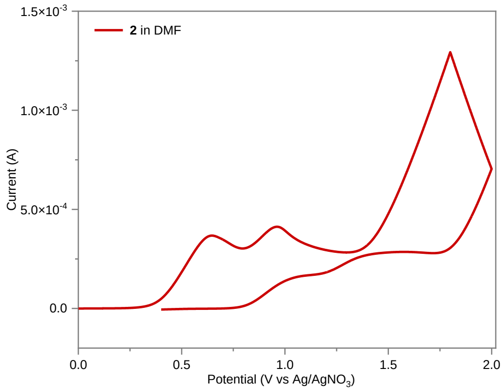

Supplementary Figure 54. The CV data of **2** in DMF. Test conditions: $^{n}Bu_4NPF_6$ (1.0 mmol, 387 mg), 2 (0.1 mmol, 24 mg) in DMF (10 mL).

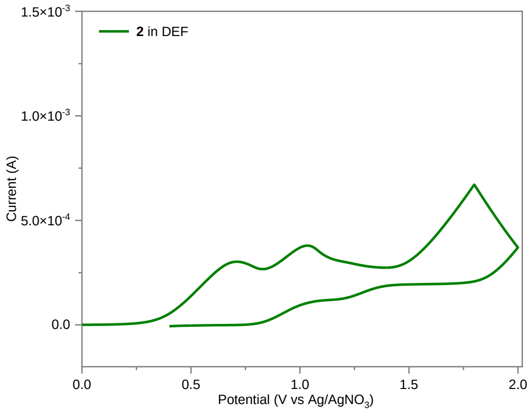

Supplementary Figure 55. The CV data of **2** in DEF. Test conditions: $^{n}Bu_4NPF_6$ (1.0 mmol, 387 mg), 2 (0.1 mmol, 24 mg) in DEF (10 mL).

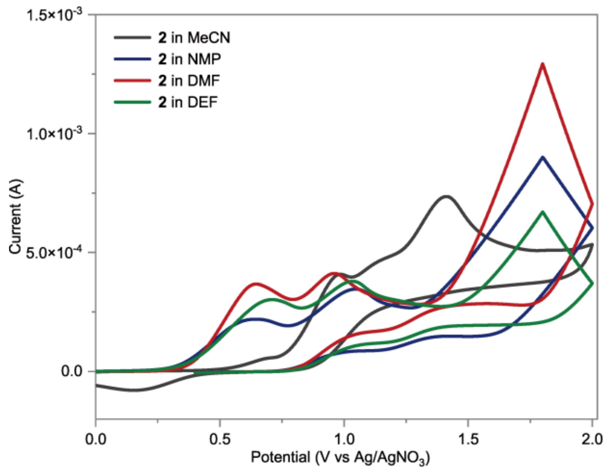

Supplementary Figure 56. The CV data of **2** in different solvents. Test conditions: $^{n}Bu_4NPF_6$ (1.0 mmol, 387 mg), **2** (0.1 mmol, 24 mg) in Solvent (10 mL). Black line: in MeCN. Blue line: in NMP. Red line: in DMF. Green line: in DEF.

From cyclic voltammetry (CV) experiments, the onset potential for DEF-ligated B_2cat_2 was lower than DMF-ligated B_2cat_2, indicating that the electronic properties of B_2cat_2 were reversed from a Lewis acid to an electron-donor complex after coordinating with the amide-based solvent and easier participate in the radical reaction.

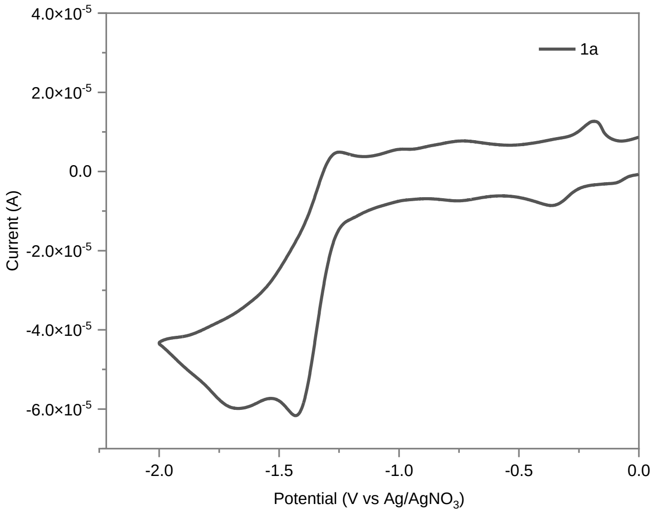

Supplementary Figure 57. The CV data of **1a**. Test conditions: $^{n}Bu_4NPF_6$ (1.0 mmol, 387 mg), **1a** (0.1 mmol, 18.8 mg) in DEF (10 mL).

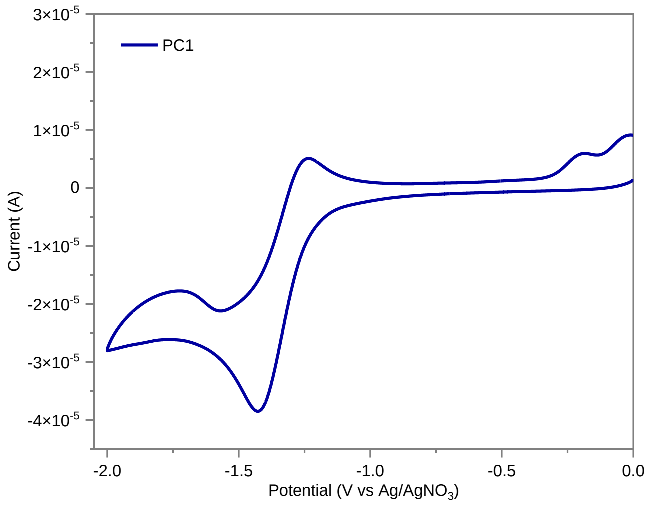

Supplementary Figure 58. The CV data of **PC1**. Test conditions: $^{n}Bu_4NPF_6$ (1.0 mmol, 387 mg), **PC1** (0.05 mmol, 64 mg) in DEF (10 mL).

Computational Investigations

All geometries were optimized in solution-phase using the PBE functional[15]and Grimme's dispersion correction with Becke-Johnson damping[16]. Gold atoms were treated with the def2-TZVP basis set and the def2-ECP pseudopotentials, and all other atoms were described by def2-SVP basis set[17]. In consideration of the solvation effects of *N*,*N*-Diethylformamide, the Integral Equation Formalism PCM (IEFPCM)[18-20] was employed. Vibrational frequency analyses were performed at the same theory as geometry optimizations to ensure all optimized stationary points as local minima (zero imaginary frequencies) or transition states (one imaginary frequency), and then thermodynamic corrections for free energies at 298.15 K were derived in Shermo Program accordingly[21]. Cogent evidences of the transition states with the corresponding intermediates were presented by intrinsic reaction coordinate (IRC) calculations[22,23]. Vertical excitation energies were computed using time-dependent density function theory (TDDFT)[24] in conjunction with PBE functional. Optimizations and vertical excitation were conducted with Gaussian 09B program[25]. The single-point energies were carried out in ORCA 5.0 program[26,27] with ωB97M-V functional[28]. And all atoms were described by def2-TZVP basis set. To evaluate the solvent effects, Conductor-like Polarizable Continuum Model (C-PCM)[29] was utilized. The resolution of identify (RI) approximation[30,31] with the Split-RI-J method was employed to accelerate the DFT calculations. Simulated UV-vis spectra were calculated with Multiwfn 3.8 dev[32] and the relevant iso-surface plot was given by VMD program[33]. All optimized geometric figures were plotted by CYLview [34].

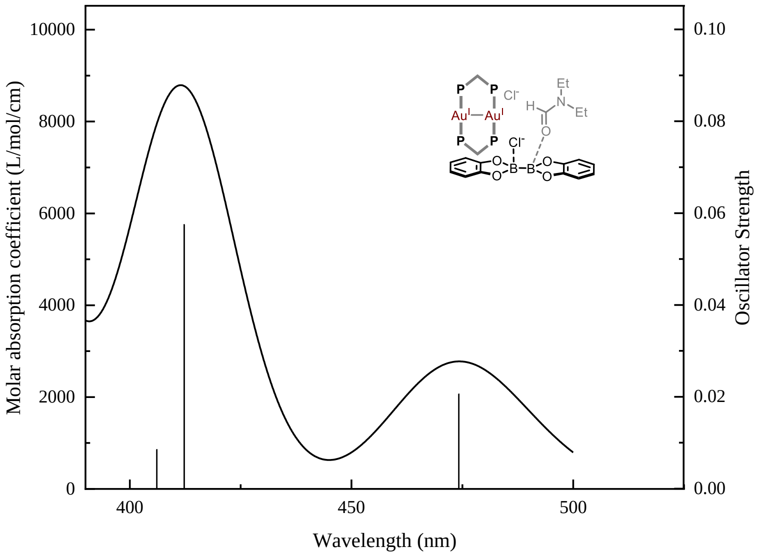

Supplementary Figure 59. Simulated UV-vis absorption spectra with state number and major character for proposed luminescent species using Gaussian broadening with a full-width at half-maximum of 0.2 eV. The gold complex at TDDFT/PBE/def2-SVP/def2-TZVP/IEFPCM(*N*,*N*-Diethylformamide) level of theory.

The intermolecular energy was approximated as:

$$E_{Inter} = E - E_1 - E_2 \qquad \text{(Supplementary Equation 1)}$$

Where E represents the single-point energy of the gold complex calculated at ωB97M-V/def2-TZVP/SMD(*N*,*N*-Diethylformamide) level of theory. E1 and E2 represent the single-point energy of the part **PC1** and **DEF-ligated B_2cat_2** in the optimized complex accordingly. According to the equations above, E_{Inter} = -37.7 kcal·mol^{-1}.

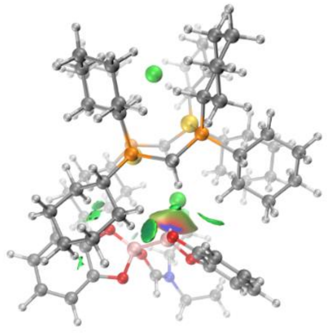

Supplementary Figure 60. Intermolecular interaction analysis between **PC1** and DEF-ligated **B_2cat_2**, and the corresponding iso-surfaces by independent gradient model based on Hirshfeld partition (IGMH)[30] (ISO=0.004 a.u.), where blue, green and red represent strong interaction, weak interaction, and steric effect.

In order to further explore the influence of fluoride anions on the HAT process, theoretical calculations were carried out.

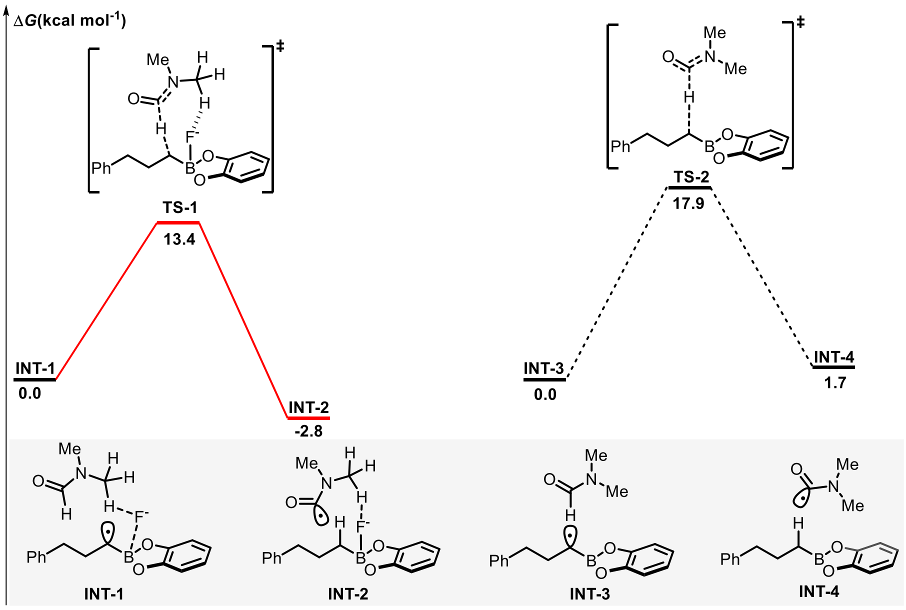

Supplementary Figure 61. At the current level of theory, the free energy of barrier of the transition state (**TS-1**) in the HAT process with fluorine anion is 13.4 kcal·mol^{-1}, 4.5 kcal·mol^{-1} lower than that of **TS-2** (17.9 kcal·mol^{-1}). In the presence of KF, the F^- anion was easily captured by α-boron radical, where the B-F bond i 1.43 Å. The hydrogen bond in **INT-1** could shorten the distance between α-

boron radical and DMF (**INT-1**: 2.41 Å vs. **INT-3**: 3.12 Å), enhancing the HAT process.

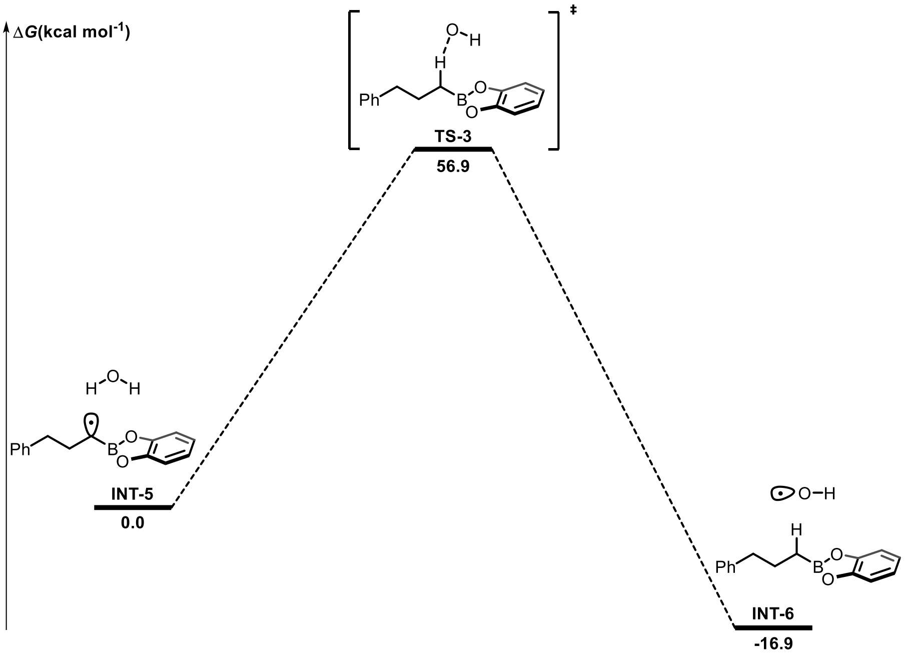

Supplementary Figure 62. Energy profile for the direct HAT process with H_2O at the PBE (D3BJ)/def2-SVP/def2-TZVP/PCM(H_2O)//RI-wB97M-V/def2-TZVP/SMD(H_2O) level theory. The free energy barrier of the transition state (**TS-3**) is 56.9 kcal·mol^{-1}, which is extremely high to proceed.

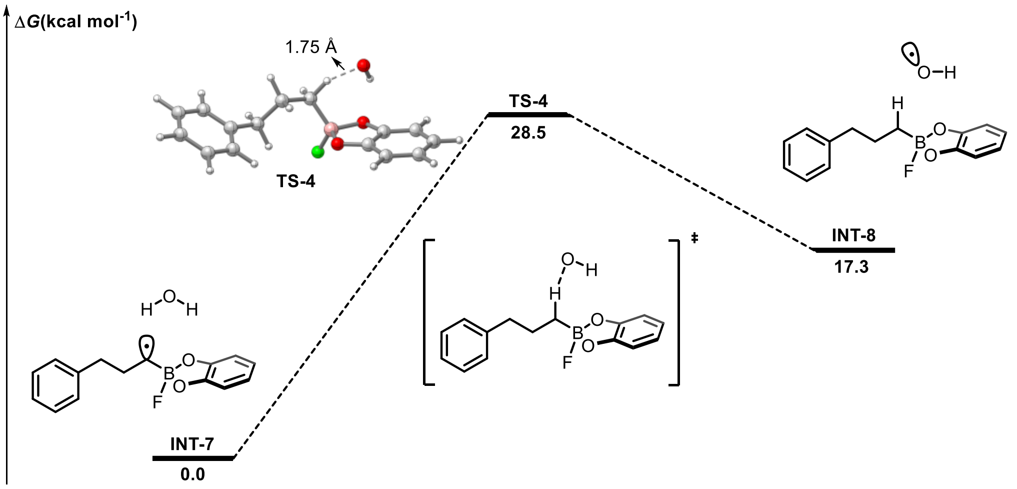

Supplementary Figure 63. Energy profile for the HAT process assistance with the fluoride in H_2O at the PBE (D3BJ)/def2-SVP/PCM(H_2O)//RI-wB97M-V/def2-TZVP/SMD(H_2O) level theory. The free energy barrier of the transition state (**TS-4**) is 28.5 kcal·mol^{-1}, much lower than that in direct HAT process (**TS-3**: 56.9 kcal·mol^{-1}), since the electron deficiency in **INT-7** promotes HAT process.

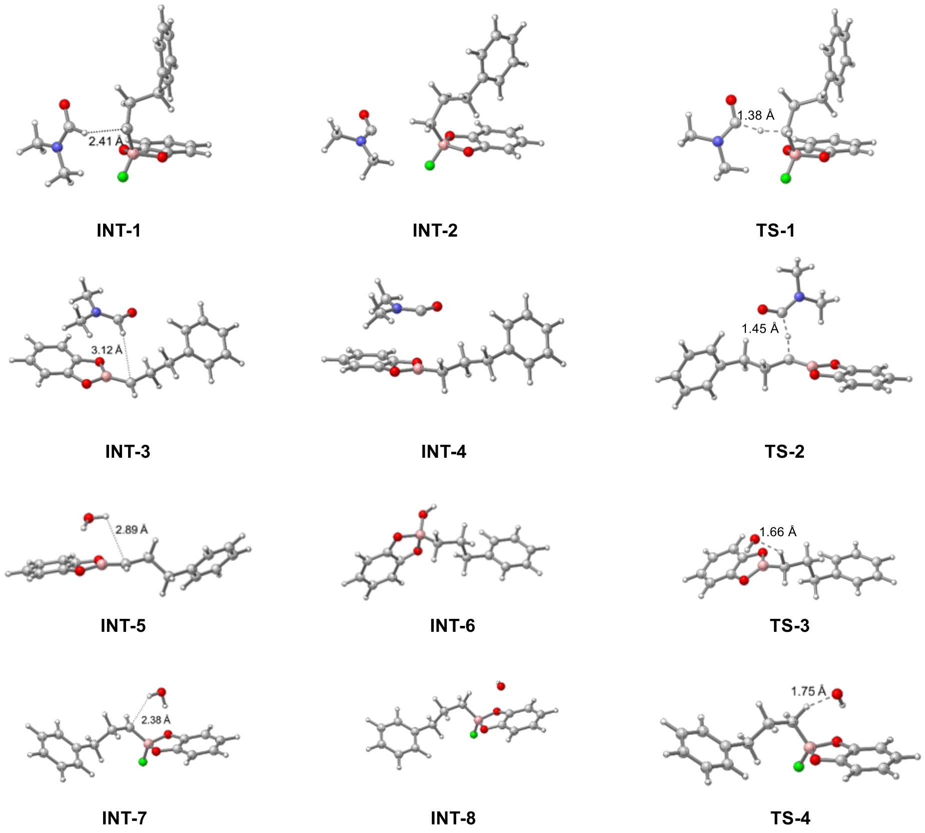

Supplementary Figure 64. Images of key optimized structures

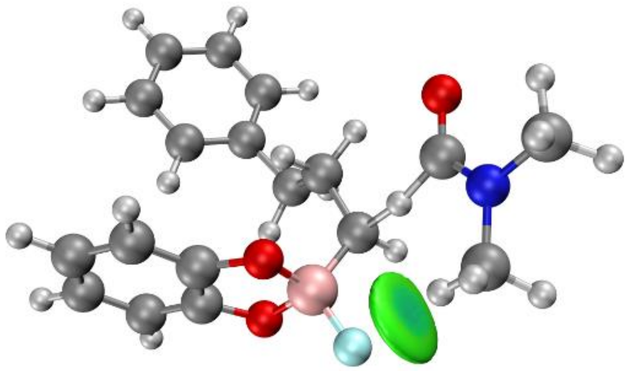

Supplementary Figure 65. Intermolecular interaction analysis between α-boron radical and DMF, and the corresponding iso-surfaces by independent gradient model based on Hirshfeld partition (IGMH) [16] (ISO=0.002 a.u.), where blue, green and red represent strong interaction, weak interaction, and steric effect.

All geometries in computationally redox potential calculations are optimized using B3LYP hybrid functional[21] and D3 version of Grimme's dispersion corrections with Becke-Johnson damping. Gold atoms are treated with the def2-TZVP basis set, and the def2-SVP basis set is for the other atoms. Vibrational frequency analyses are conducted at the same level of theory (B3LYP-D3/def2-SVP/def2-TZVP) to ensure all stationary points as local minima. The single point energy calculations are conducted using B3LYP hybrid functional and Grimme's dispersion correction with Becke–Johnson damping, and the def2-TZVP is used for all atoms. And the solvation effects of acetonitrile are incorporated by with the Solvent Model Density (SMD)[22] method. The redox potentials can be estimated by [23]:

$$E = -\frac{G_{298}(red) - G_{298}(ox)}{F} - E_{ref} \qquad \text{(Supplementary Equation 2)}$$

where G_{red} and G_{ox} are Gibbs free energies for oxidized species and reduced species respectively, and F is Faraday's constant. The E_{ref} is absolute potential of saturated calomel electrode (SCE) in acetonitrile (E_{ref} = 4.422 V)[35].

Supplementary Table 10. Gibbs free energies and redox potential for intermediate **81** and **82**

**Gibbs free energy (298K) in a.u.**				**Redox potential (V)**
Oxidized Species		Reduced Species		
**83**	-733.60	**82**	-733.70	-1.56
**81**	-4518.38	**PC1**	-4518.54	-0.10

Copies of NMR Spectra

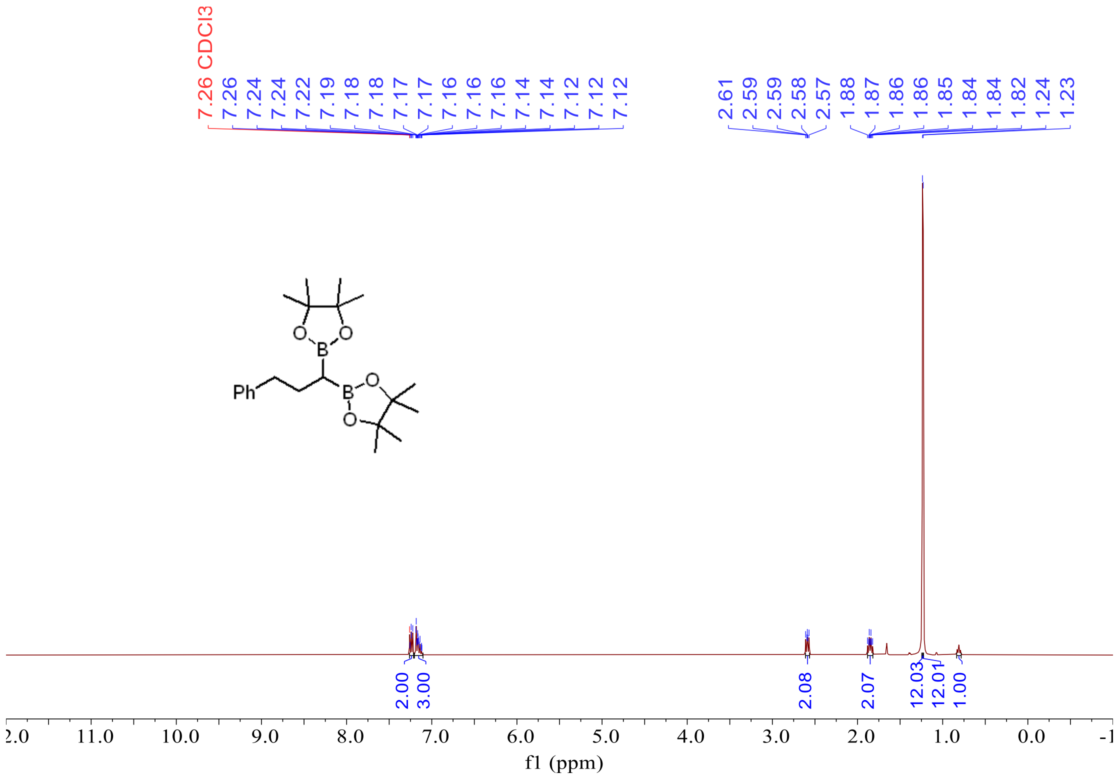

Supplementary Figure 66. ^{1}H NMR (400 MHz, $CDCl_3$) spectra for compound **3**

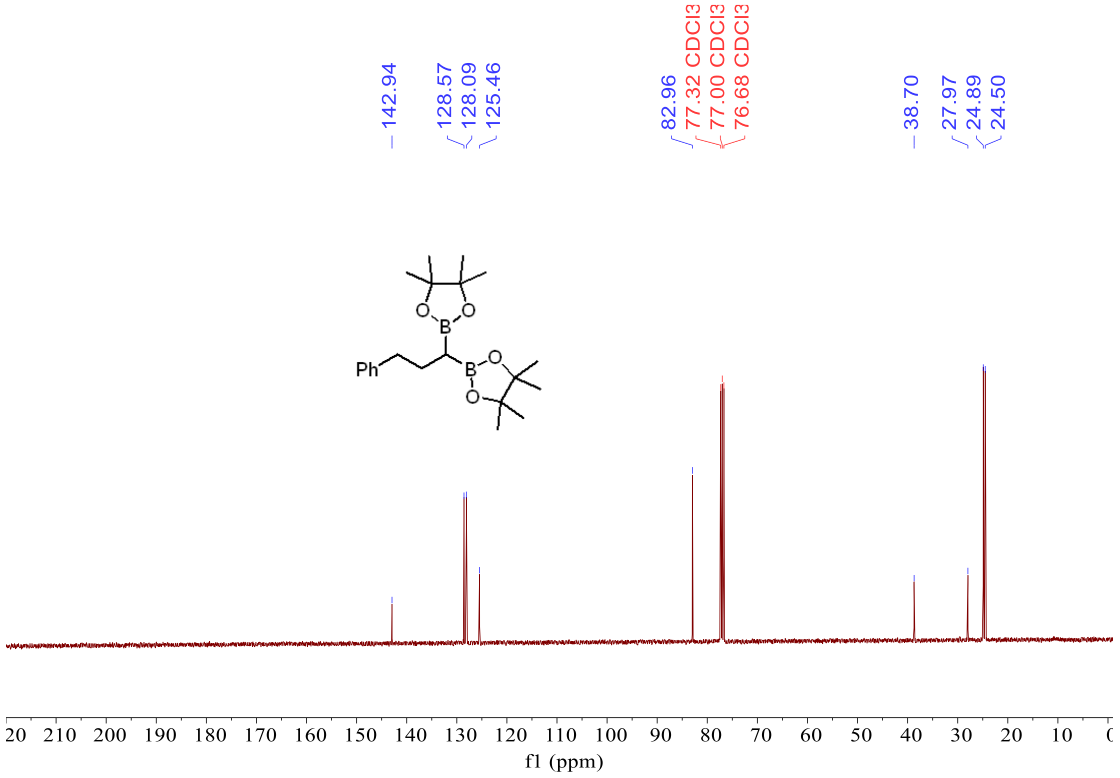

Supplementary Figure 67. ^{13}C NMR (101 MHz, $CDCl_3$) spectra for compound **3**

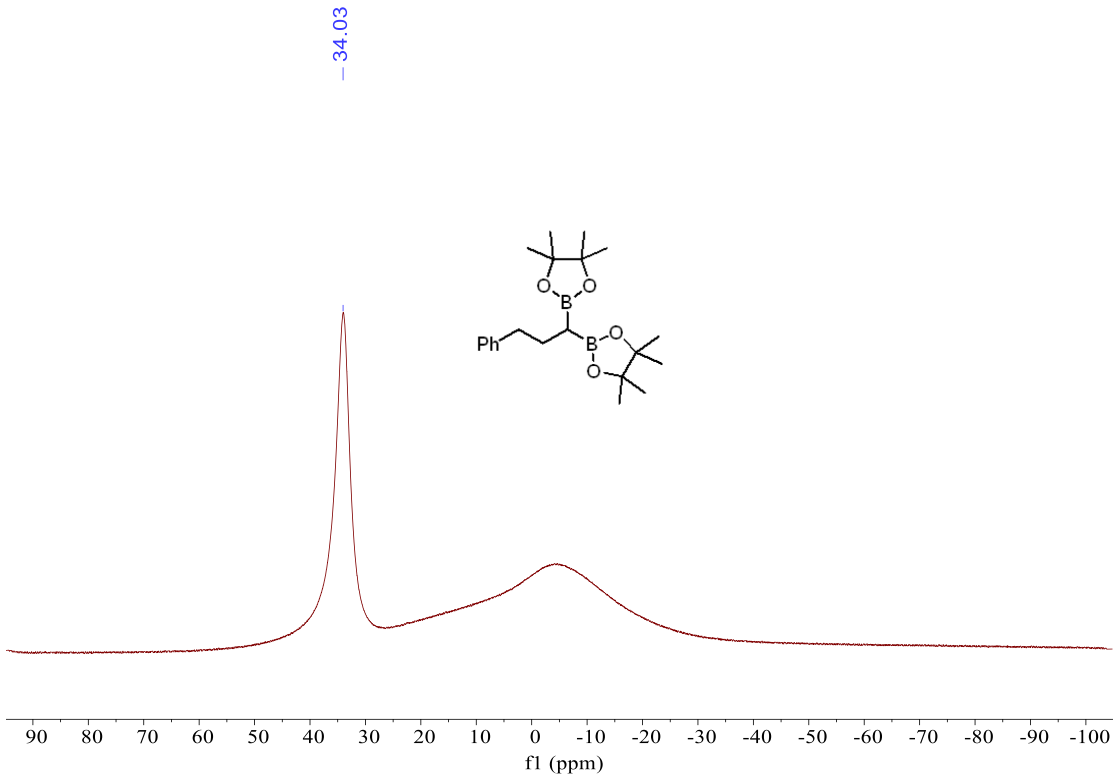

Supplementary Figure 68. ^{11}B NMR (160 MHz, $CDCl_3$) spectra for compound **3**

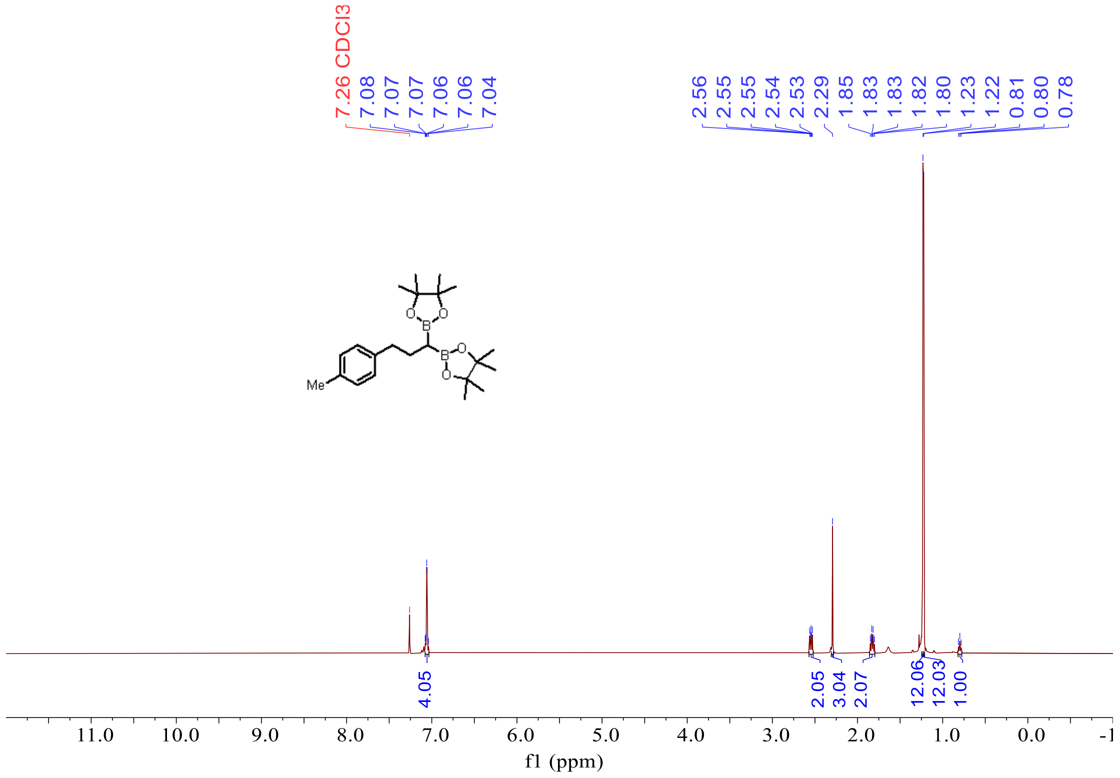

Supplementary Figure 69. ^{1}H NMR (500 MHz, $CDCl_3$) spectra for compound **4**

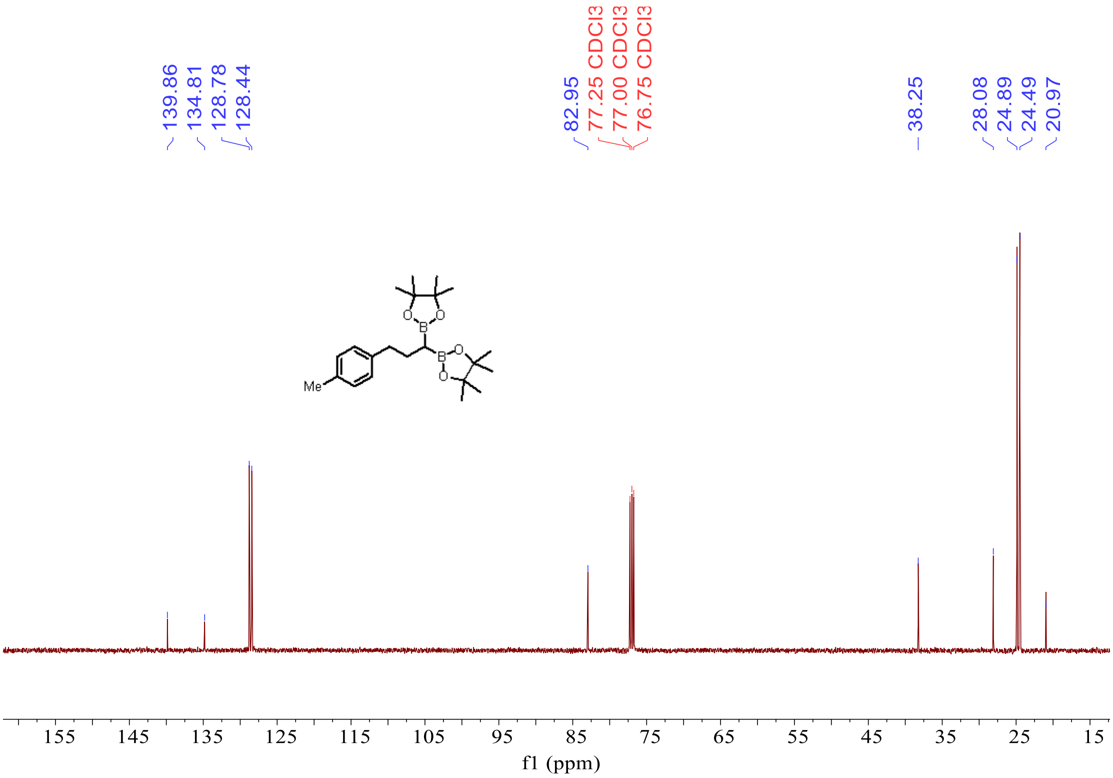

Supplementary Figure 70. ^{13}C NMR (126 MHz, $CDCl_3$) spectra for compound **4**

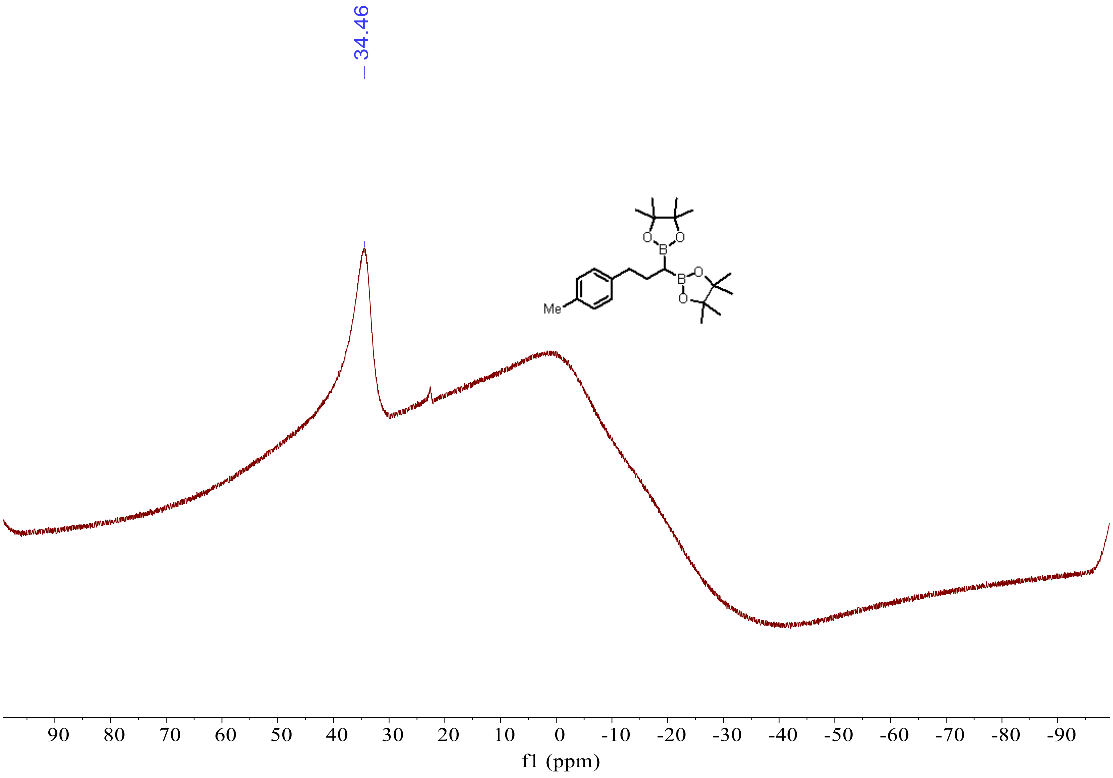

Supplementary Figure 71. ^{11}B NMR (128 MHz, $CDCl_3$) spectra for compound **4**

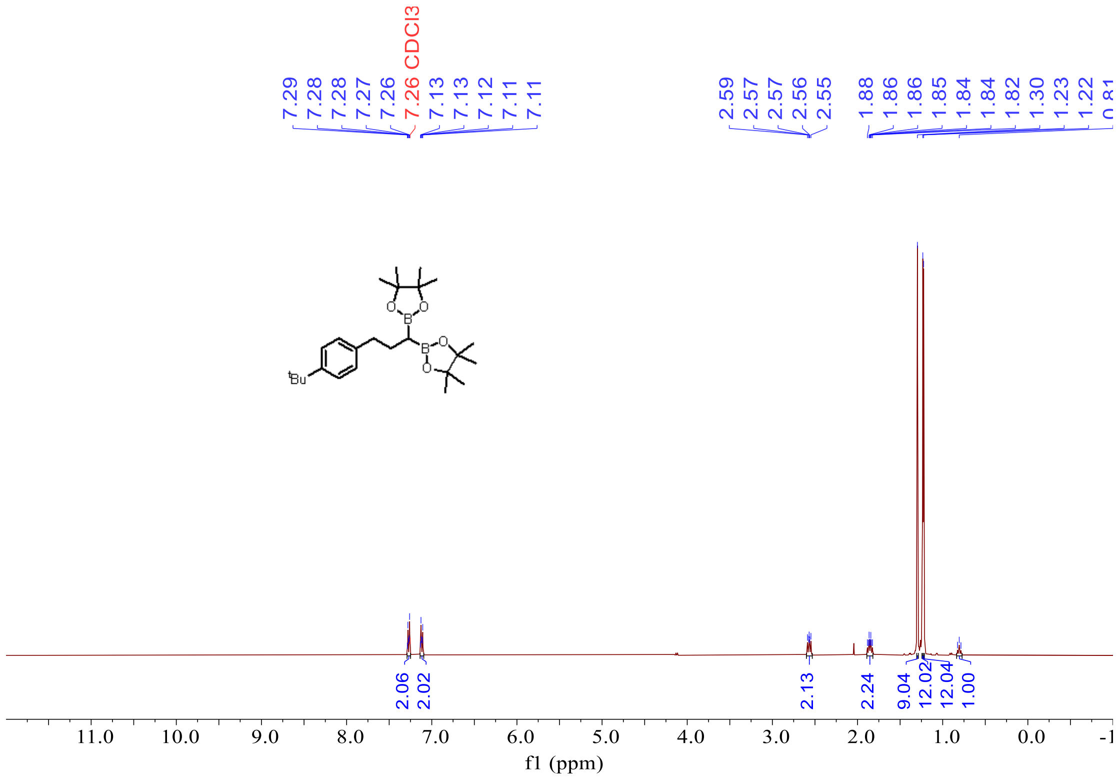

Supplementary Figure 72. ^{1}H NMR (400 MHz, $CDCl_3$) spectra for compound **5**

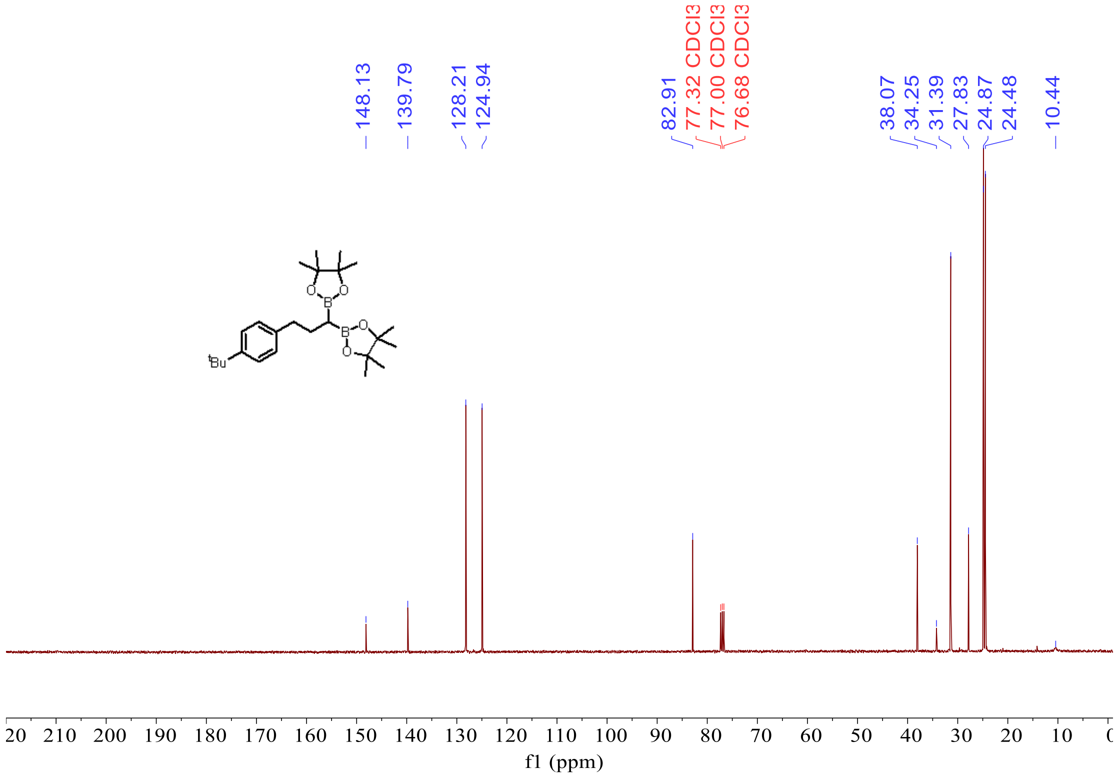

Supplementary Figure 73. ^{13}C NMR (101 MHz, $CDCl_3$) spectra for compound **5**

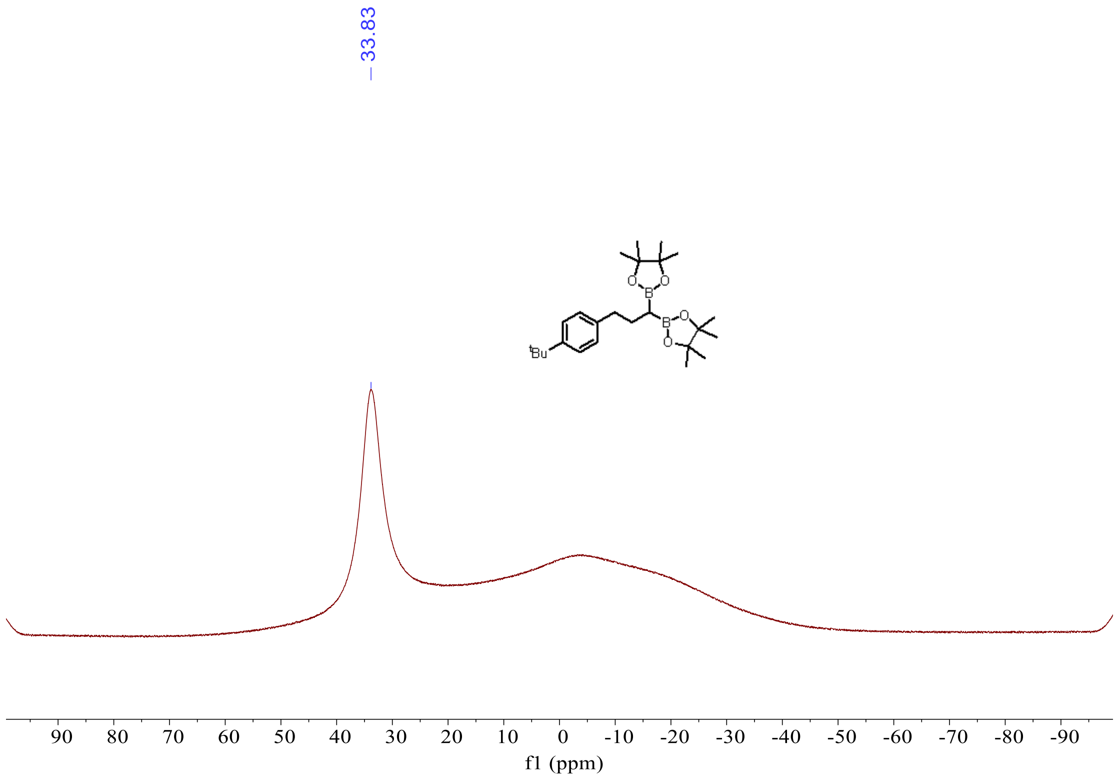

Supplementary Figure 74. ^{11}B NMR (128 MHz, $CDCl_3$) spectra for compound **5**

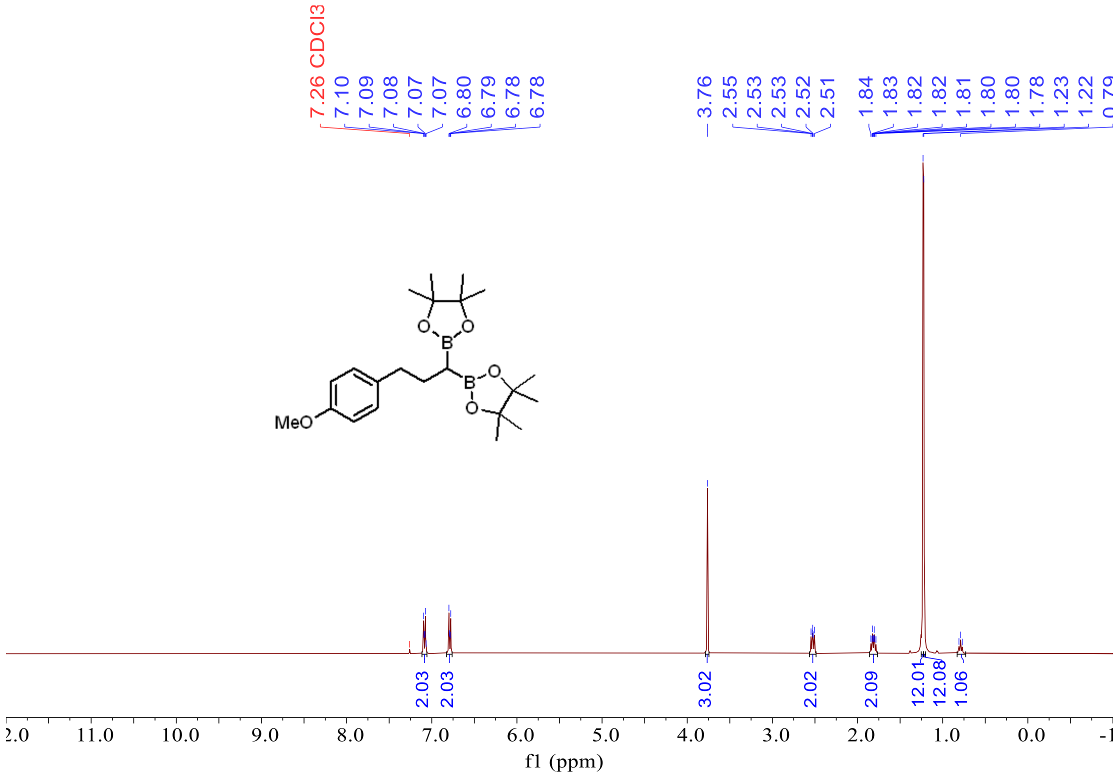

Supplementary Figure 75. ^{1}H NMR (400 MHz, $CDCl_3$) spectra for compound **6**

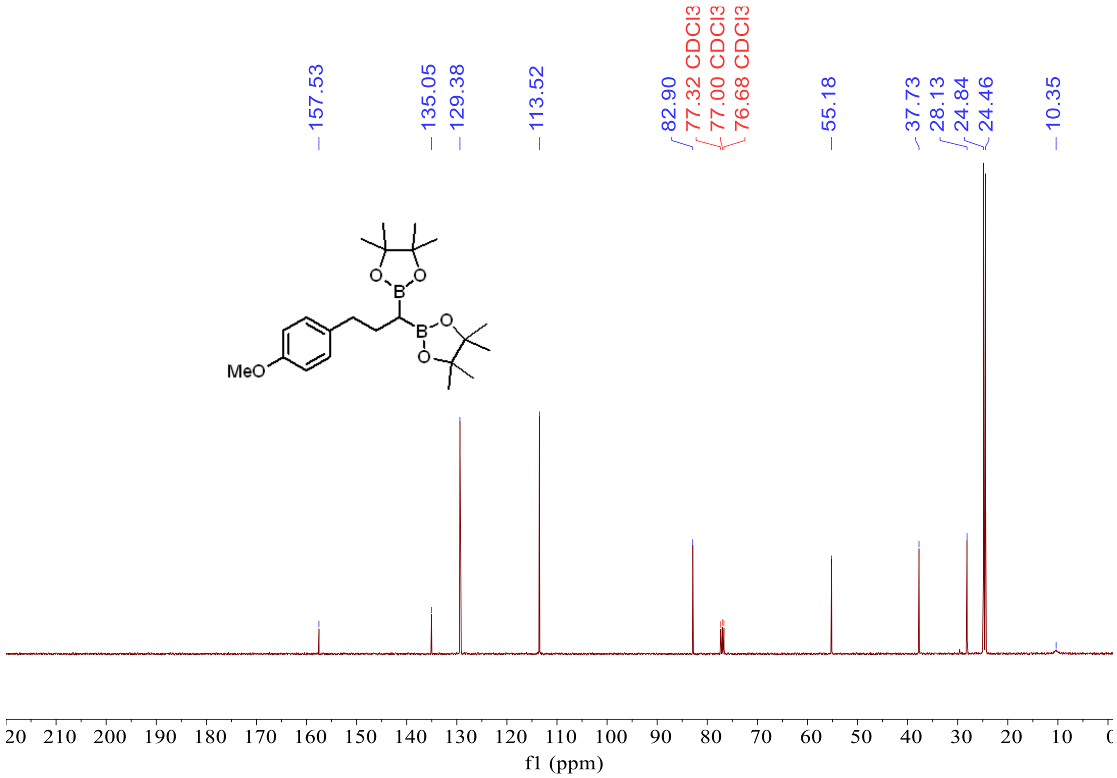

Supplementary Figure 76. ^{13}C NMR (101 MHz, $CDCl_3$) spectra for compound **6**

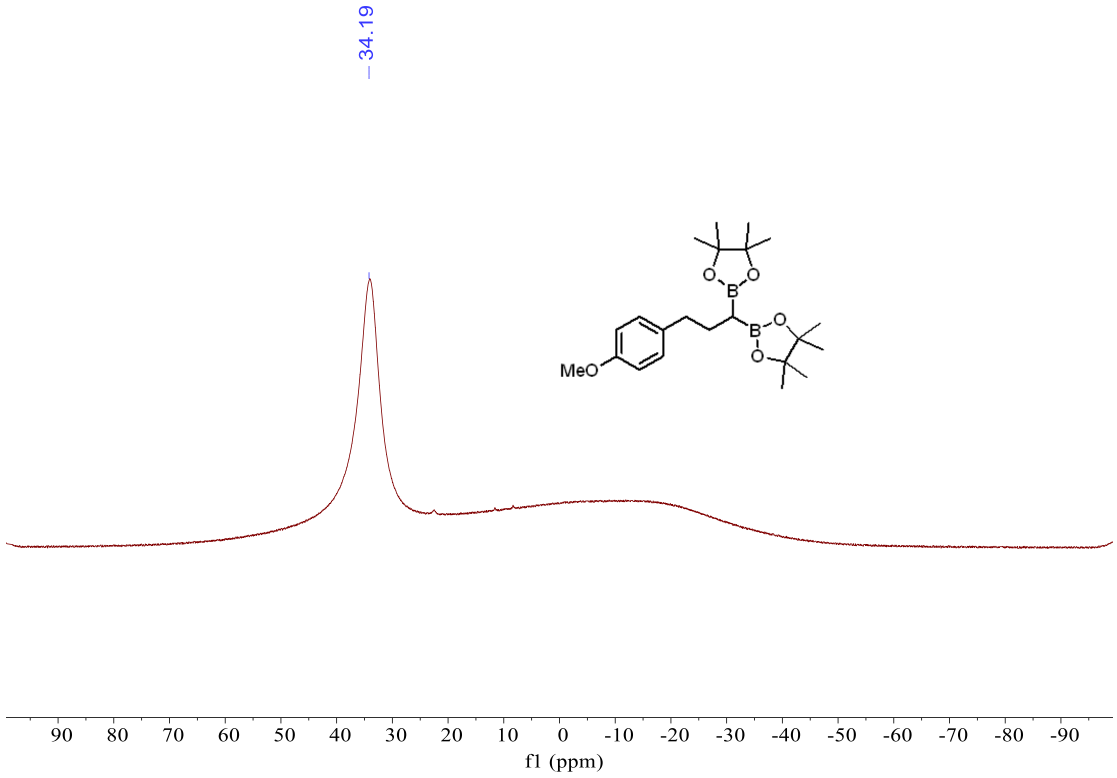

Supplementary Figure 77. ^{11}B NMR (128 MHz, $CDCl_3$) spectra for compound **6**

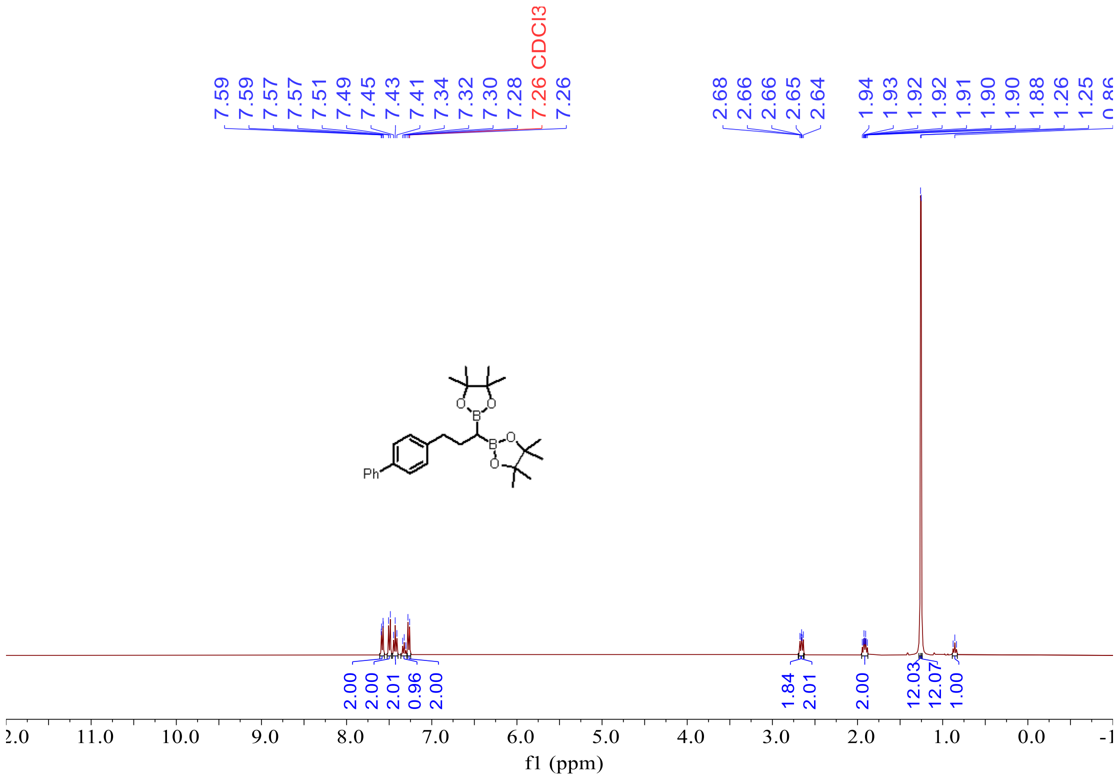

Supplementary Figure 78. ^{1}H NMR (400 MHz, $CDCl_3$) spectra for compound **7**

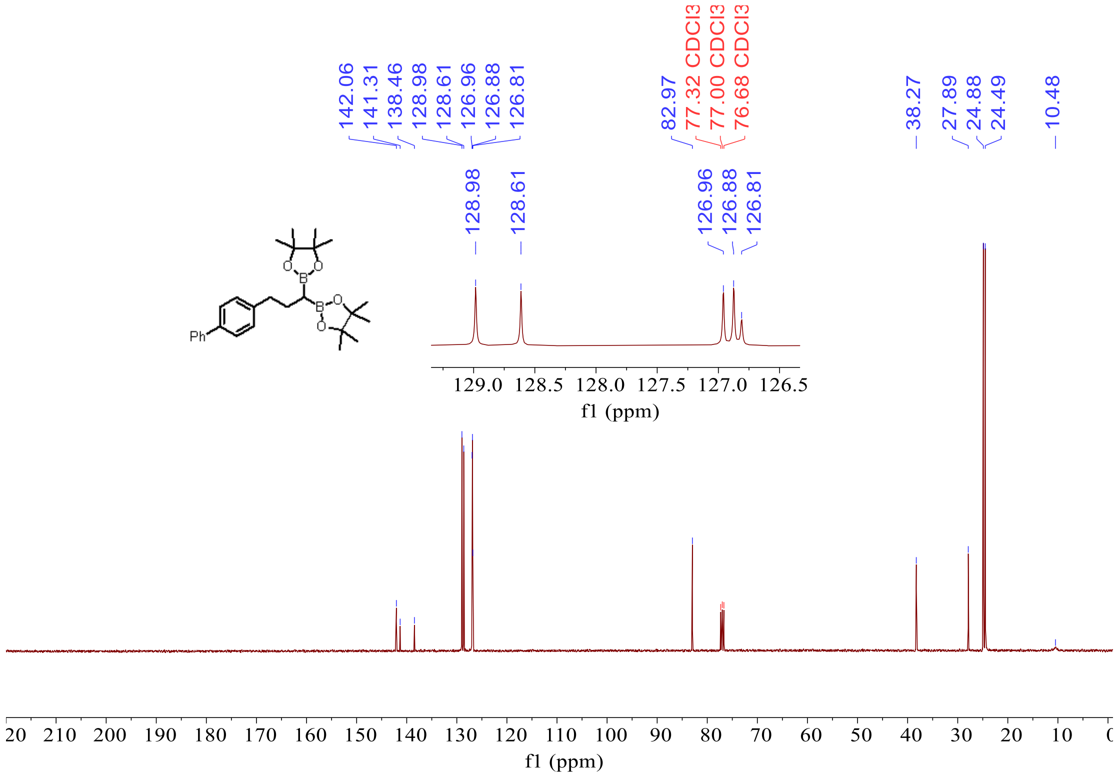

Supplementary Figure 79. ^{13}C NMR (101 MHz, $CDCl_3$) spectra for compound **7**

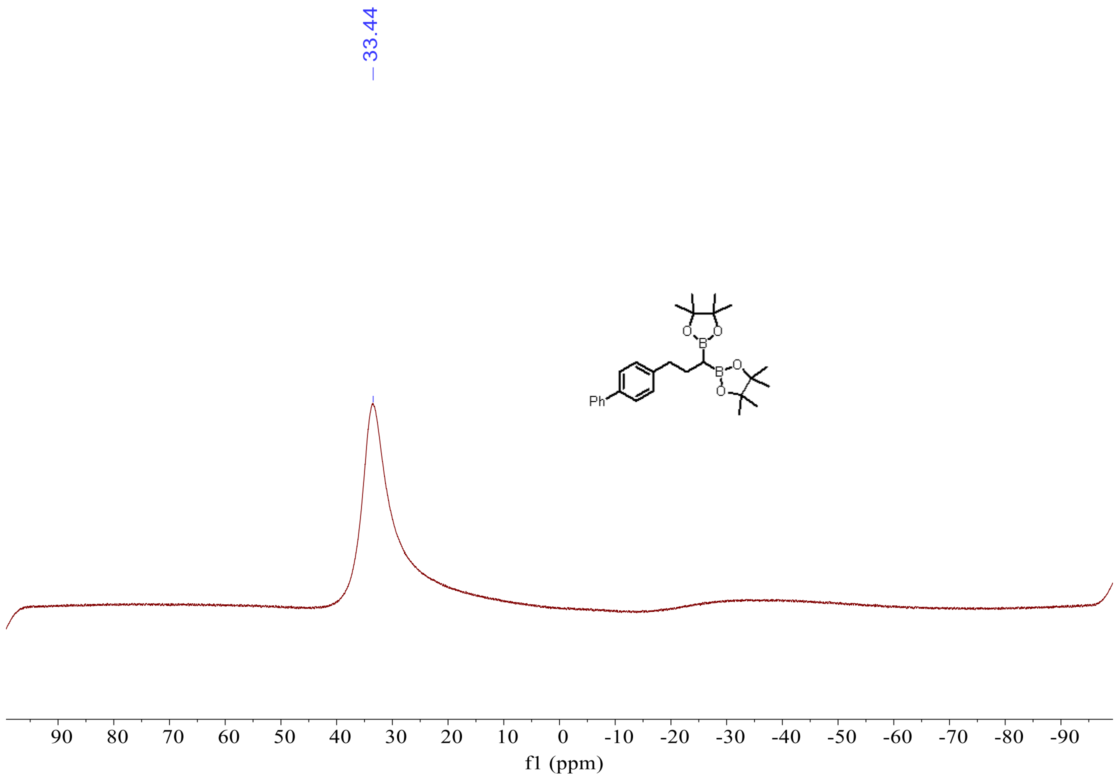

Supplementary Figure 80. ^{11}B NMR (128 MHz, $CDCl_3$) spectra for compound **7**

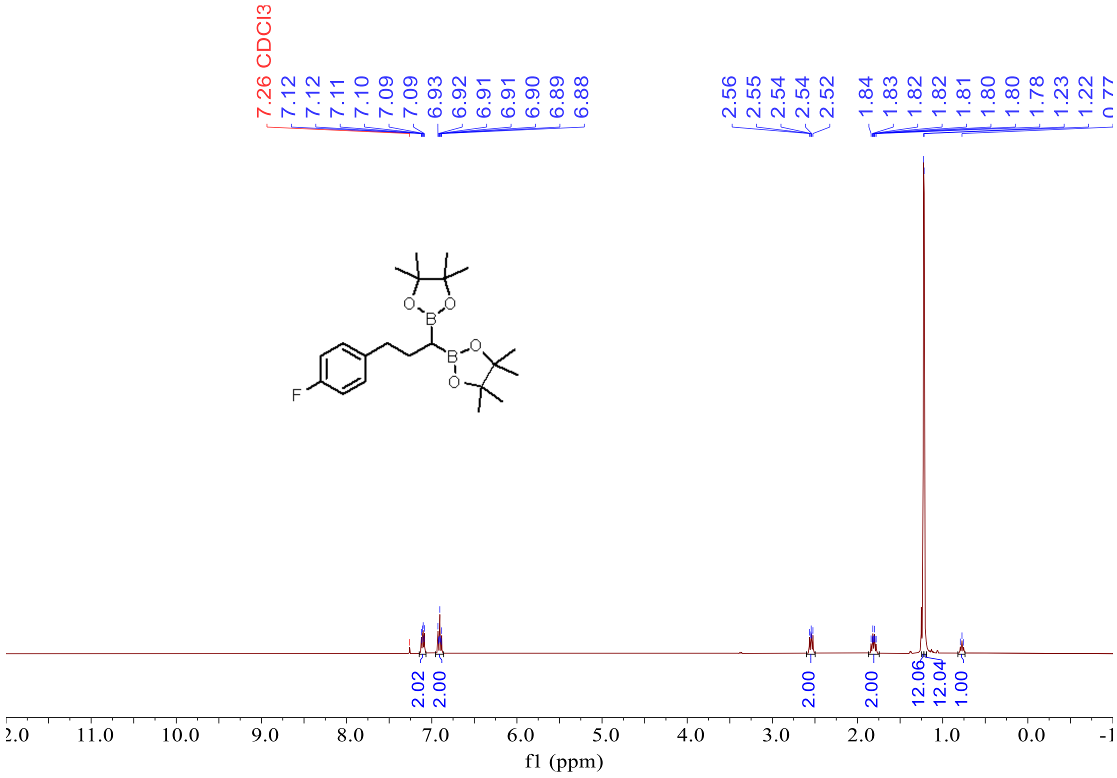

Supplementary Figure 81. ^{1}H NMR (400 MHz, $CDCl_3$) spectra for compound **8**

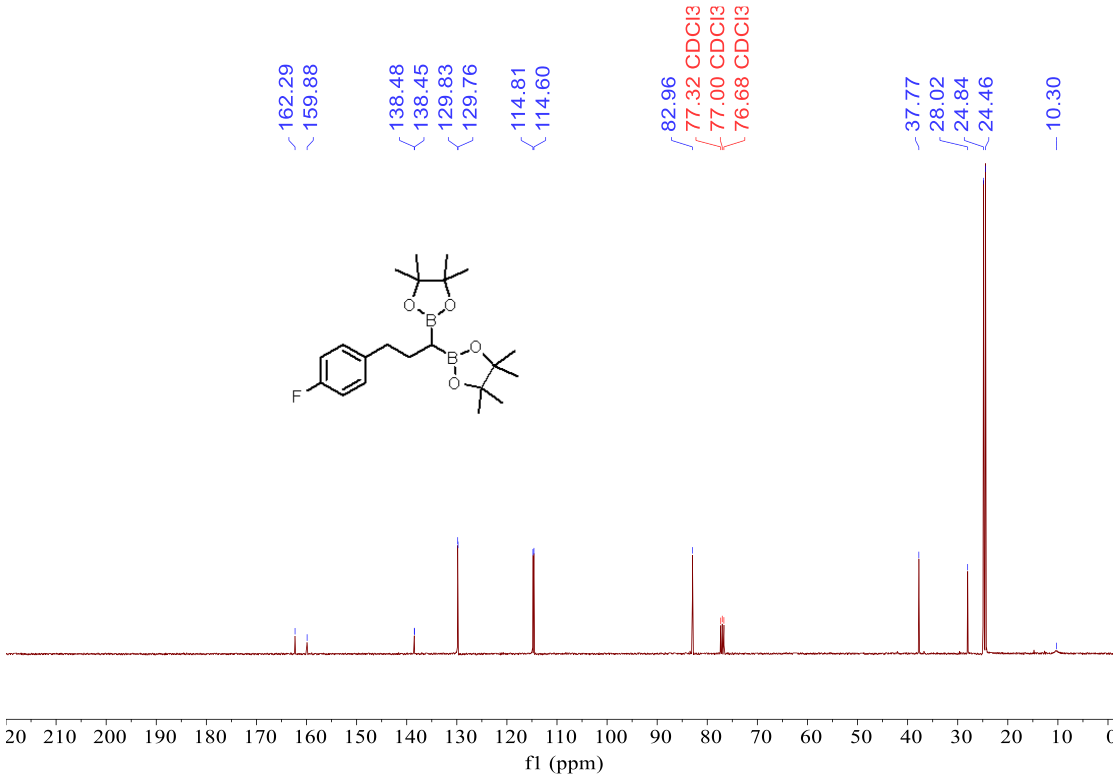

Supplementary Figure 82. ^{13}C NMR (101 MHz, $CDCl_3$) spectra for compound **8**

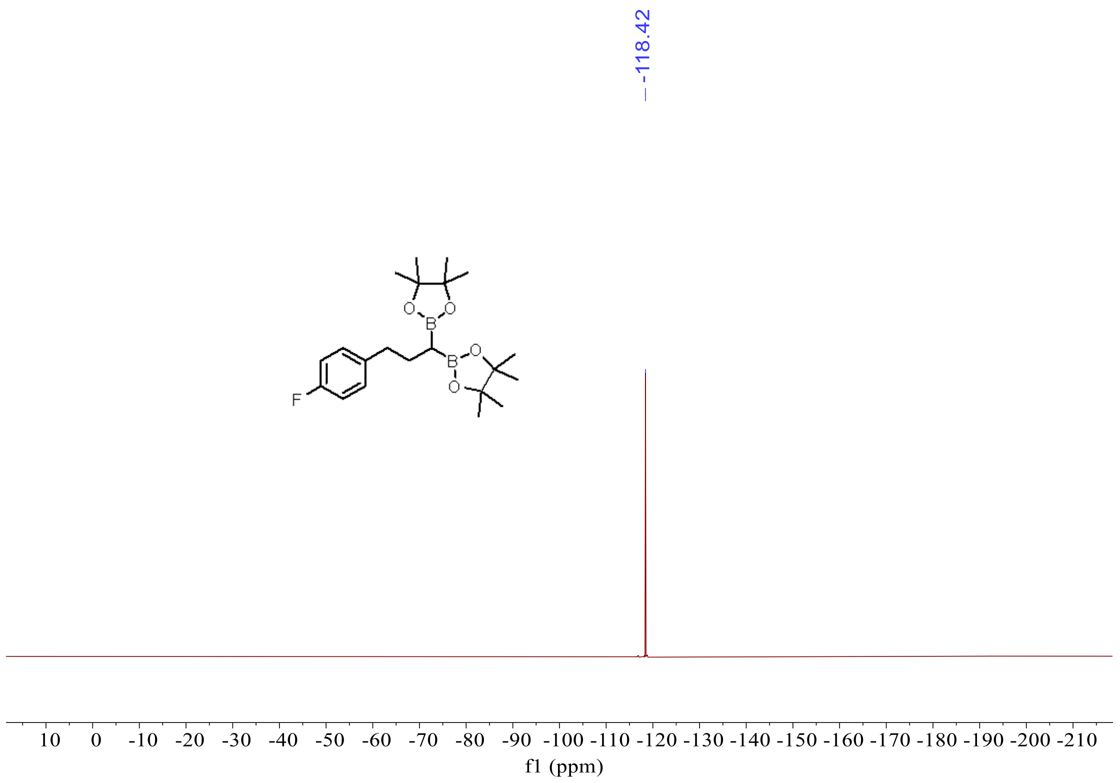

Supplementary Figure 83. ^{19}F NMR (376 MHz, $CDCl_3$) spectra for compound **8**

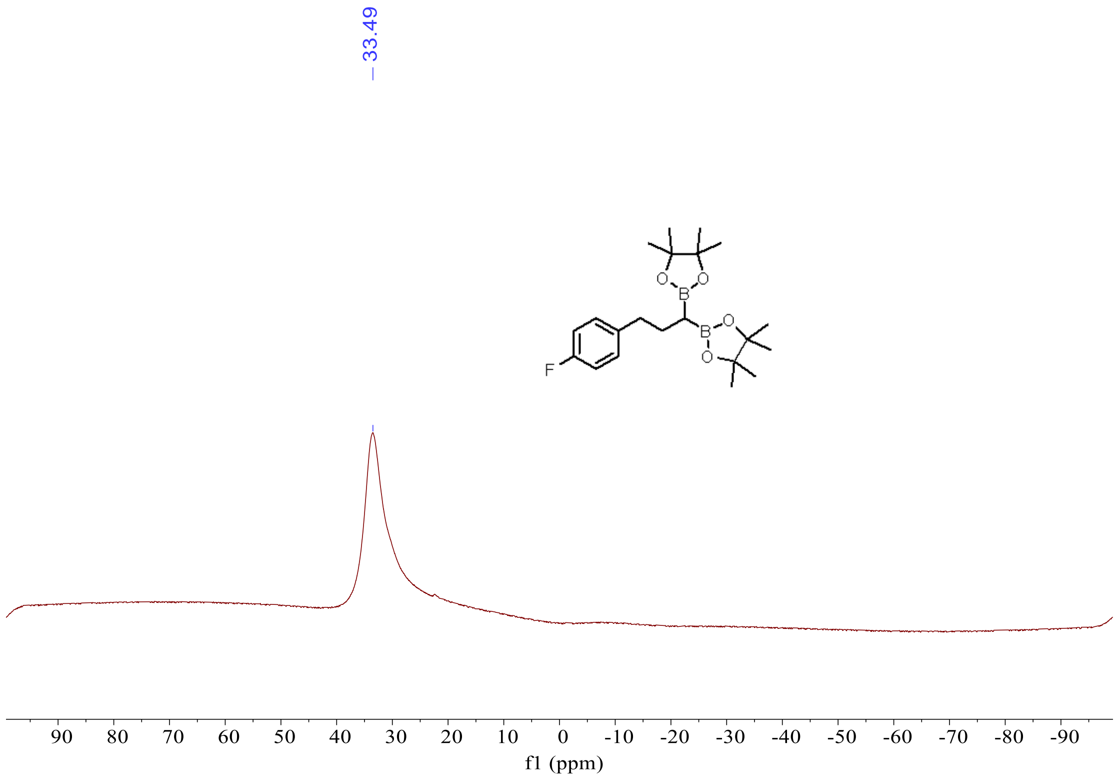

Supplementary Figure 84. ^{11}B NMR (128 MHz, $CDCl_3$) spectra for compound **8**

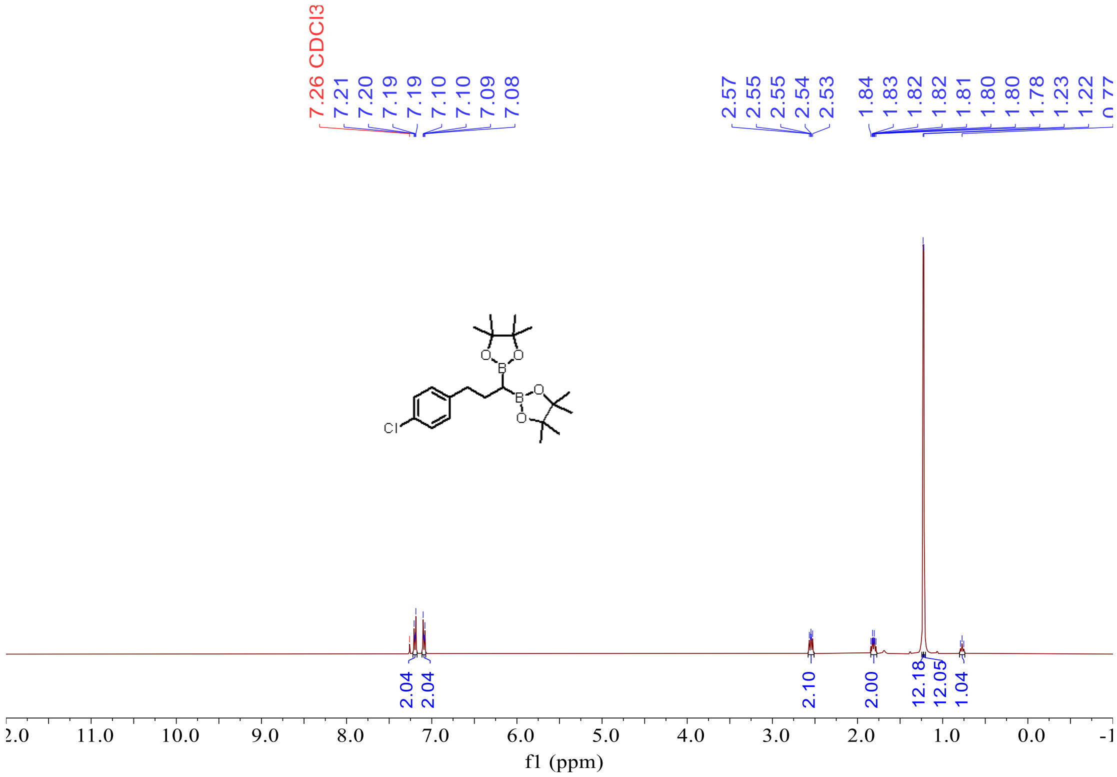

Supplementary Figure 85. ^{1}H NMR (400 MHz, $CDCl_3$) spectra for compound **9**

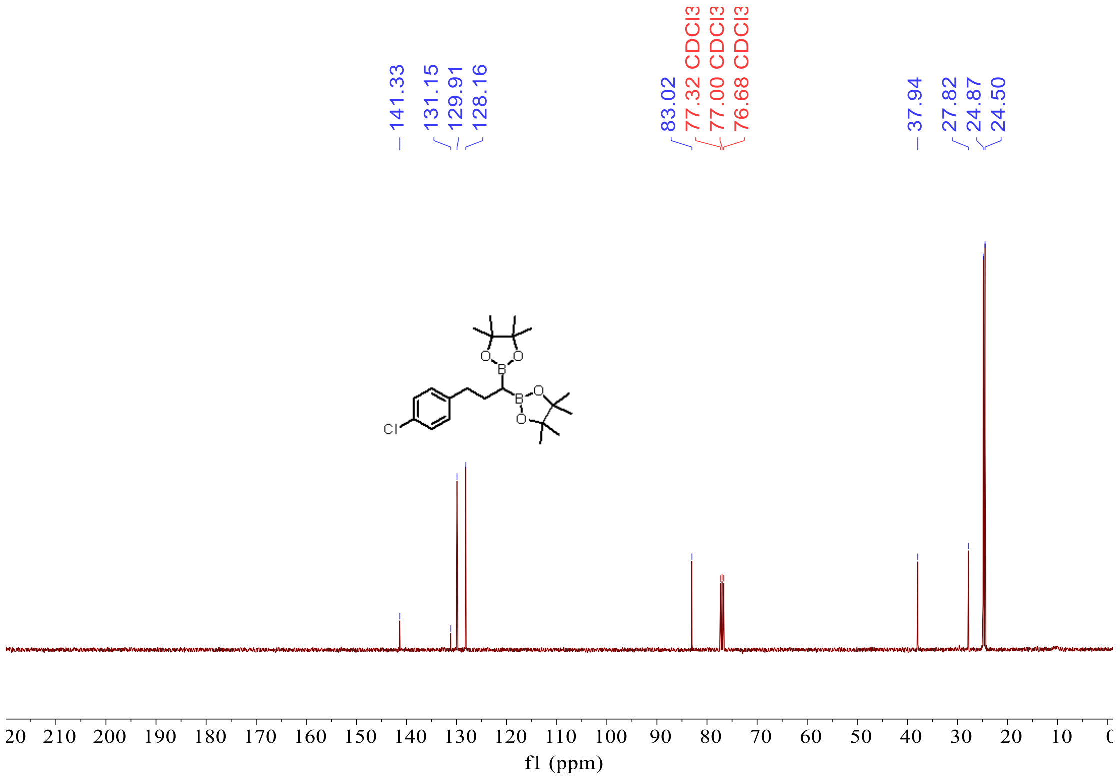

Supplementary Figure 86. ^{13}C NMR (101 MHz, $CDCl_3$) spectra for compound **9**

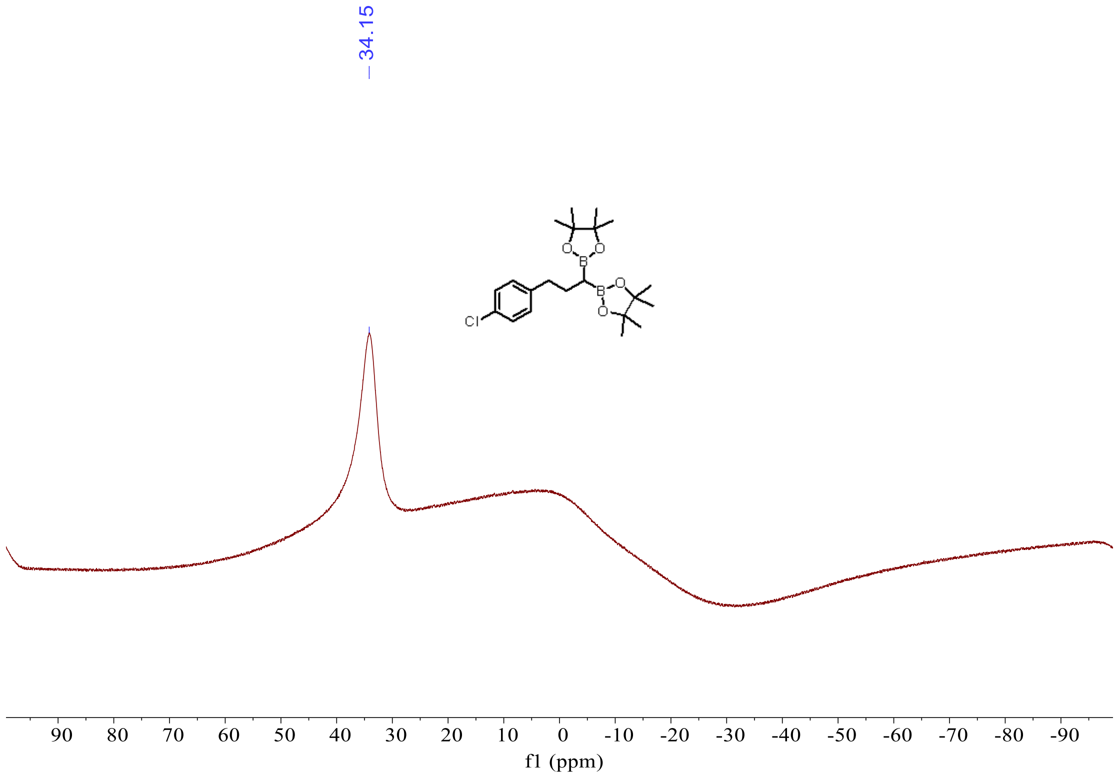

Supplementary Figure 87. ^{11}B NMR (128 MHz, $CDCl_3$) spectra for compound **9**

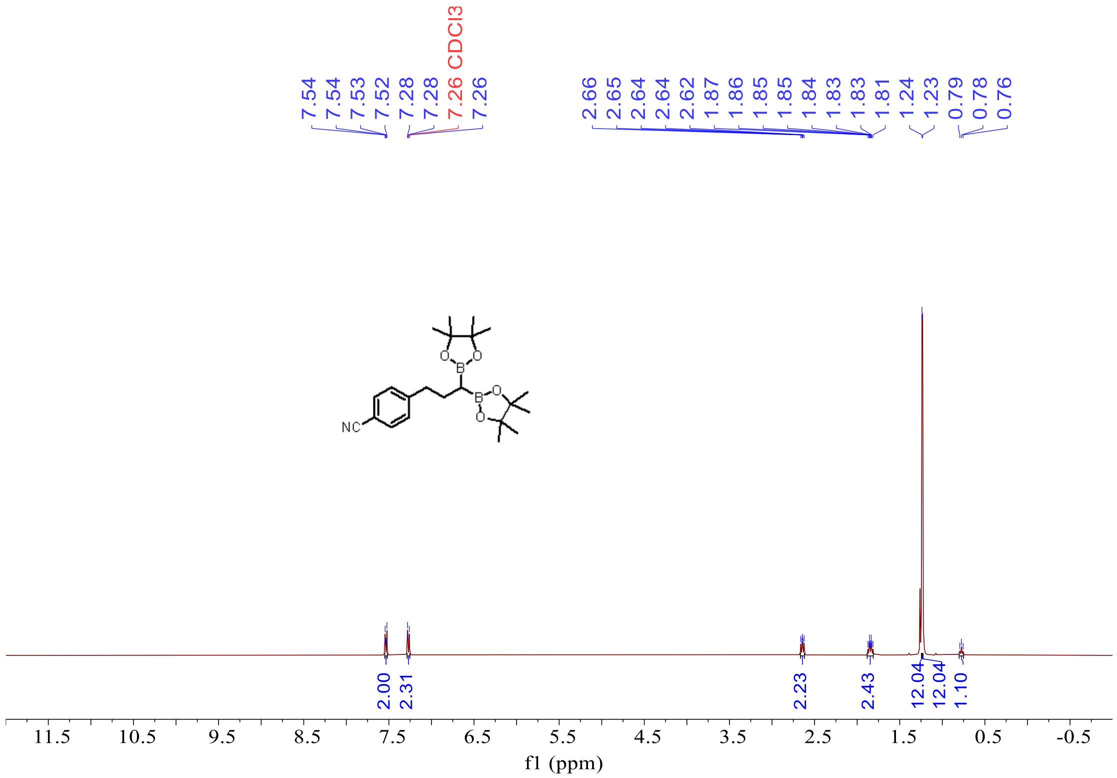

Supplementary Figure 88. ^{1}H NMR (400 MHz, $CDCl_3$) spectra for compound **10**

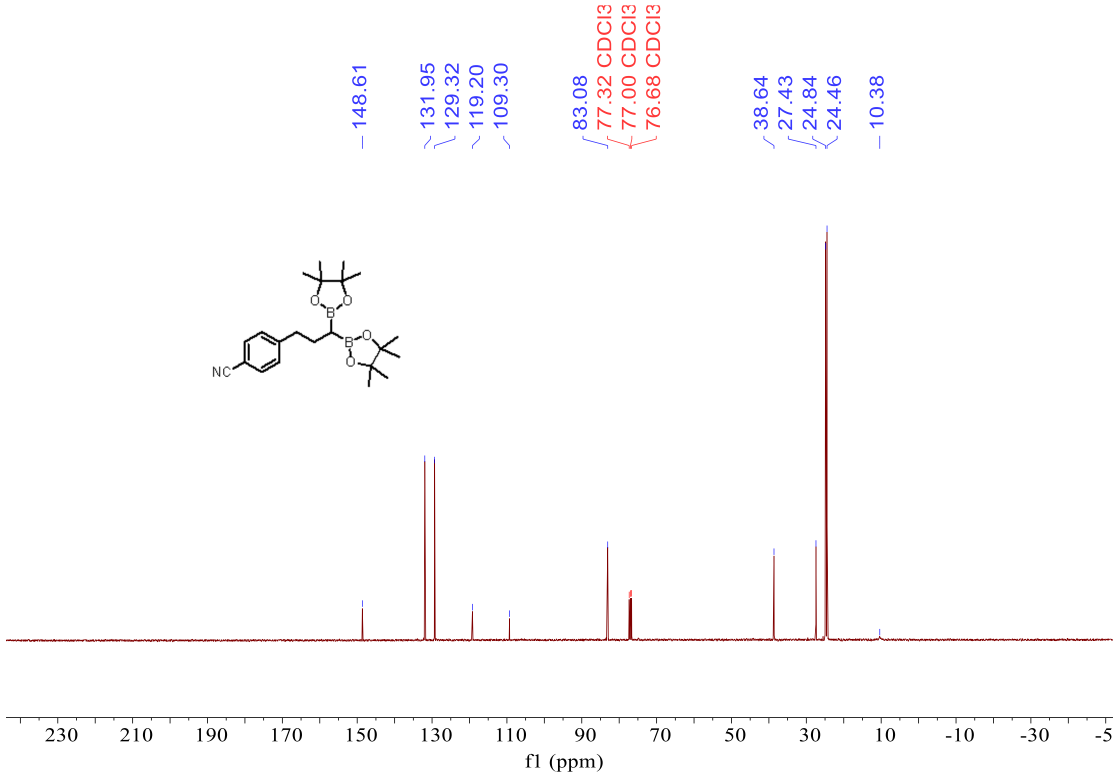

Supplementary Figure 89. ^{13}C NMR (101 MHz, $CDCl_3$) spectra for compound **10**

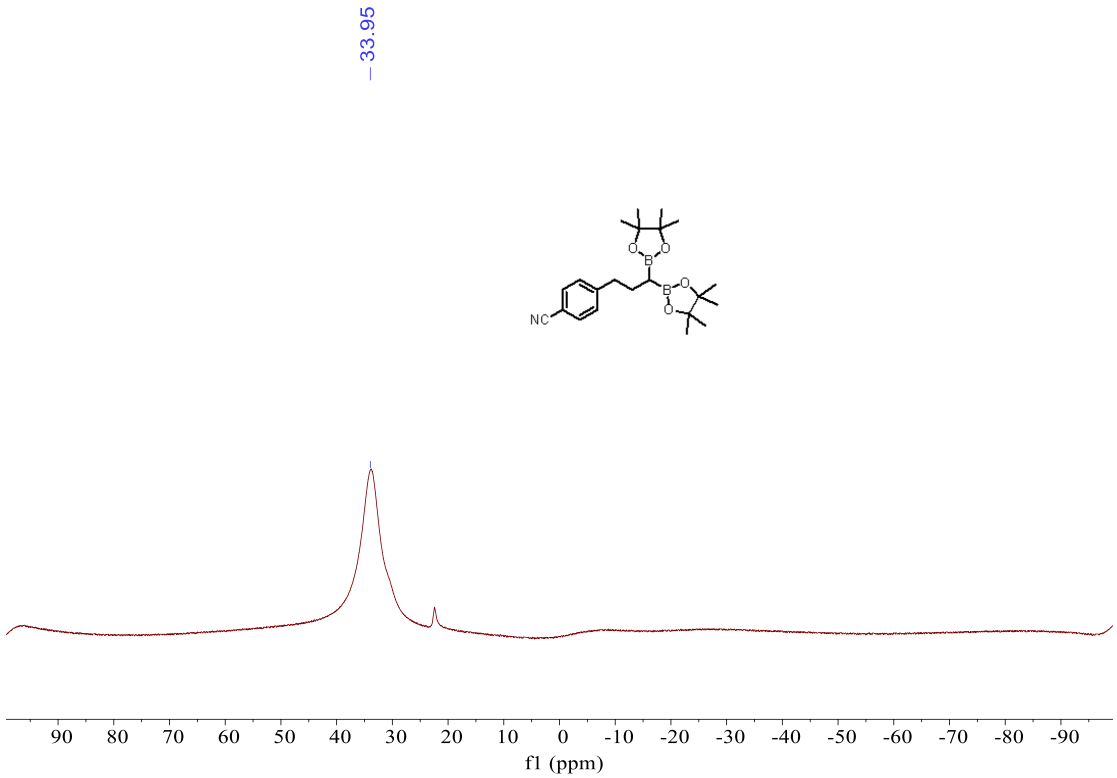

Supplementary Figure 90. ^{11}B NMR (128 MHz, $CDCl_3$) spectra for compound **10**

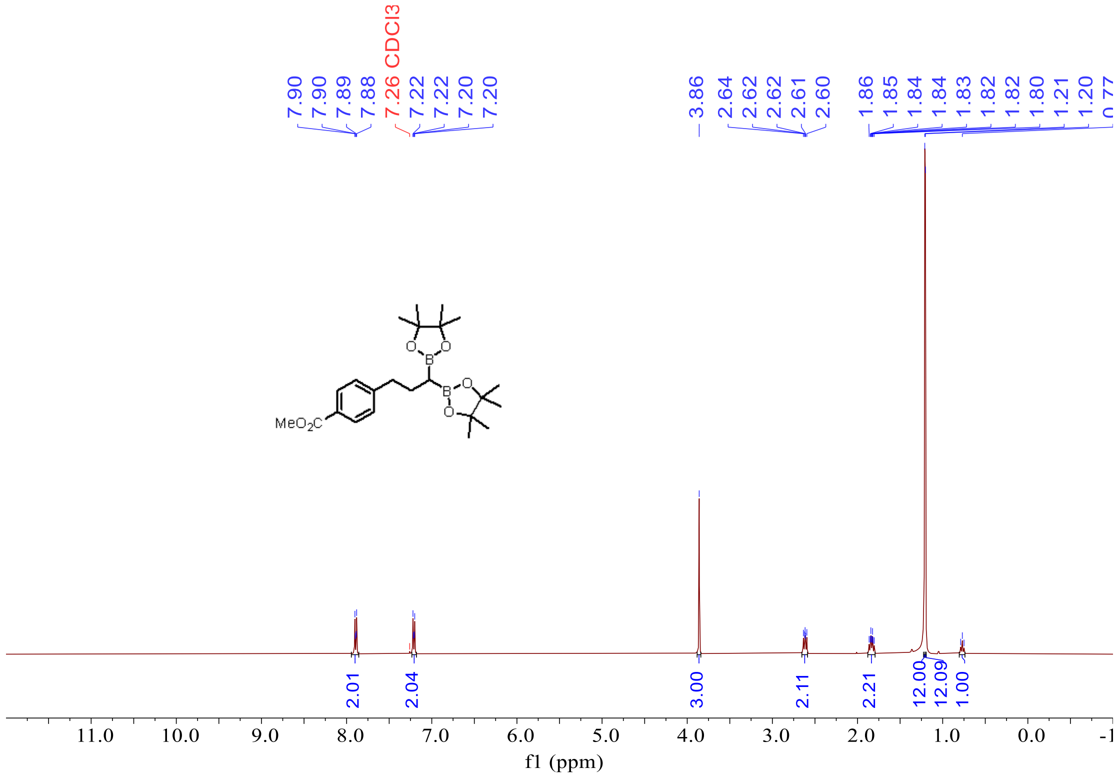

Supplementary Figure 91. ^{1}H NMR (400 MHz, $CDCl_3$) spectra for compound **11**

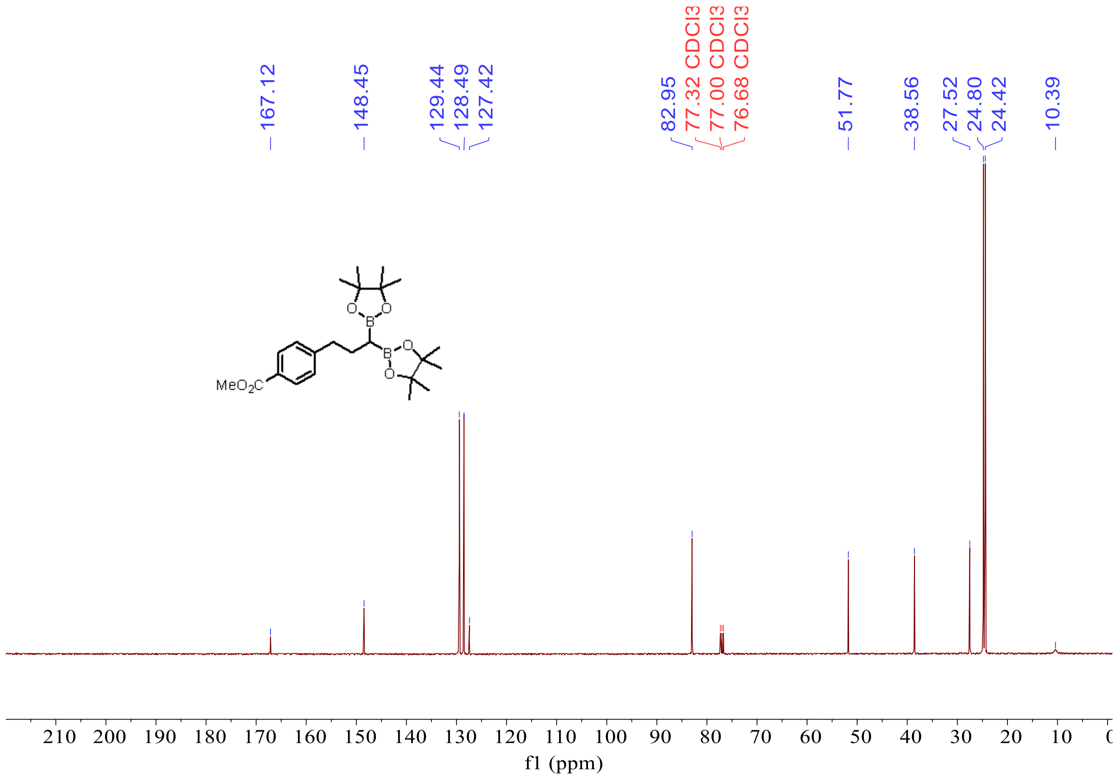

Supplementary Figure 92. ^{13}C NMR (101 MHz, $CDCl_3$) spectra for compound **11**

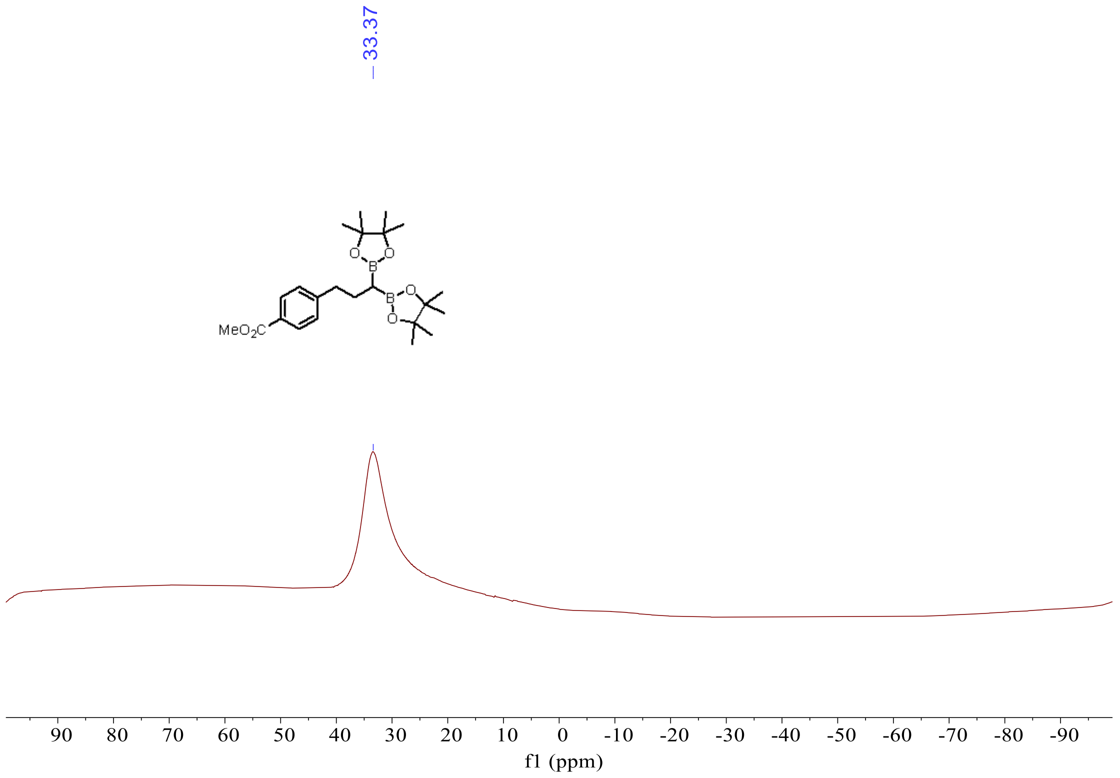

Supplementary Figure 93. ^{11}B NMR (128 MHz, $CDCl_3$) spectra for compound **11**

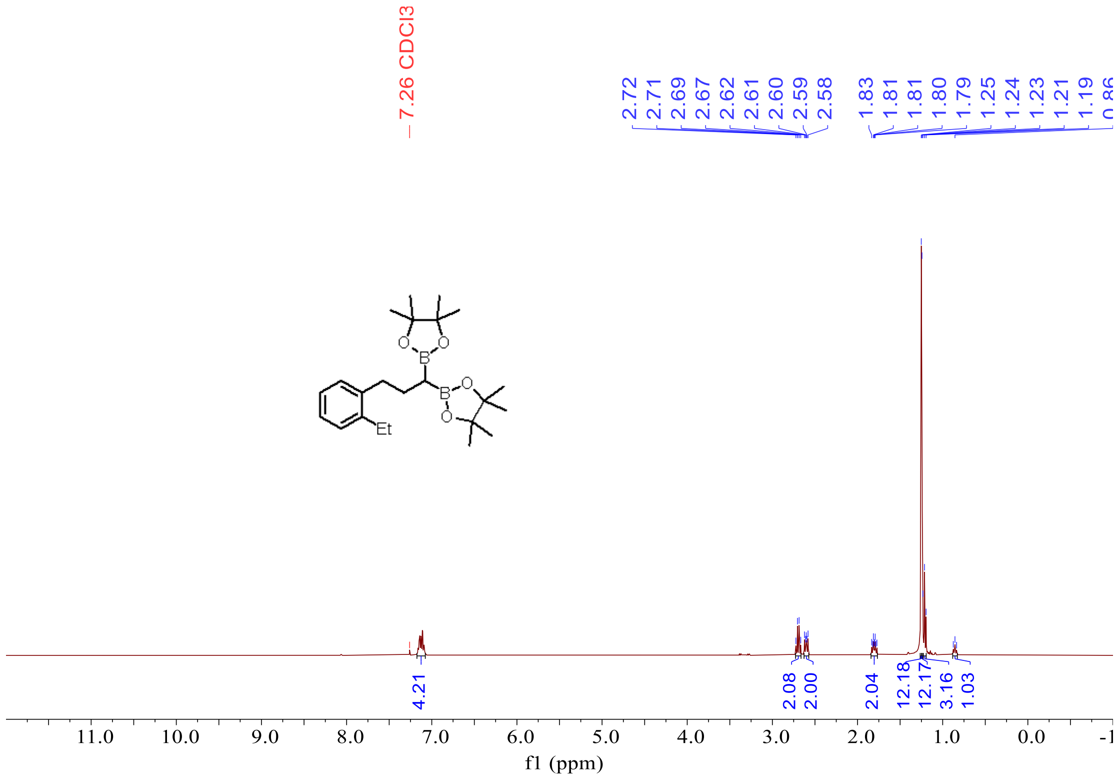

Supplementary Figure 94. ^{1}H NMR (400 MHz, $CDCl_3$) spectra for compound **12**

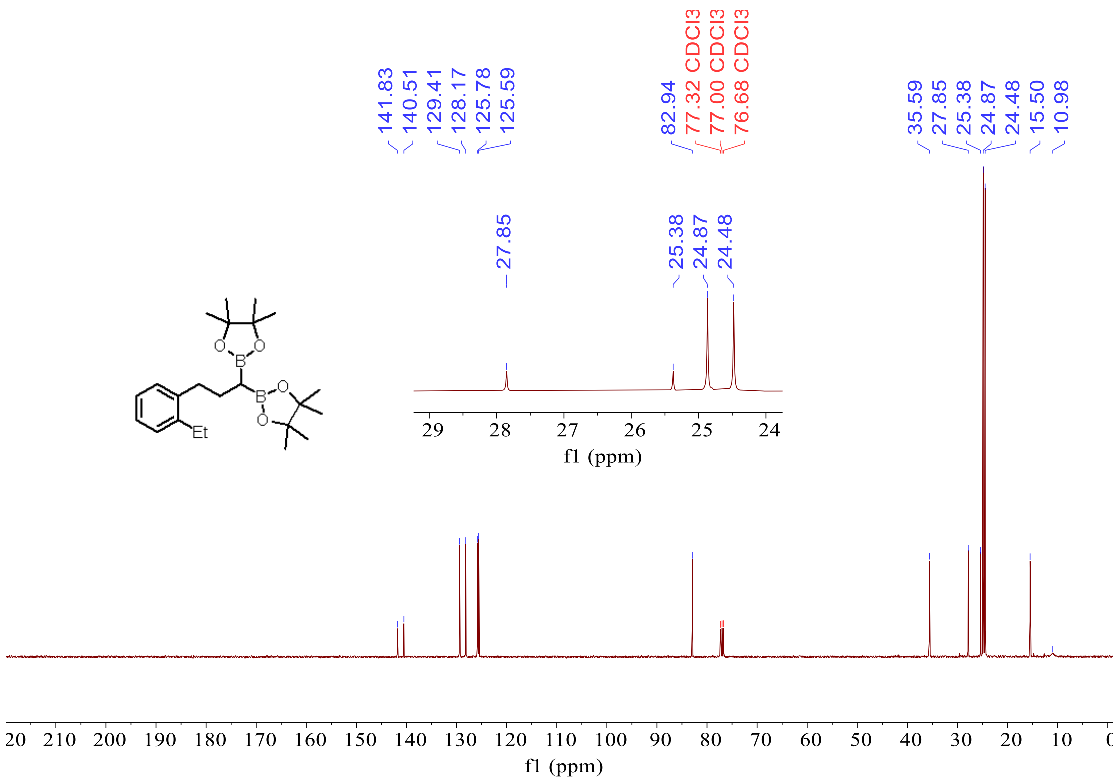

Supplementary Figure 95. ^{13}C NMR (101 MHz, $CDCl_3$) spectra for compound **12**

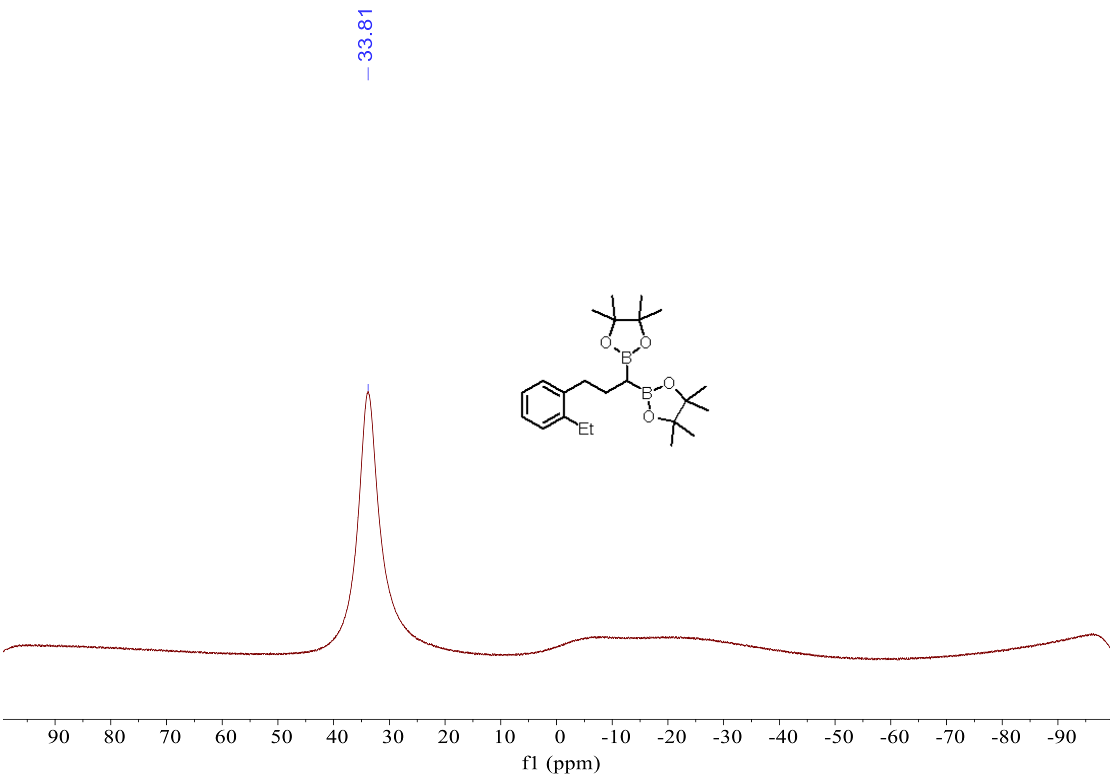

Supplementary Figure 96. ^{11}B NMR (128 MHz, $CDCl_3$) spectra for compound **12**

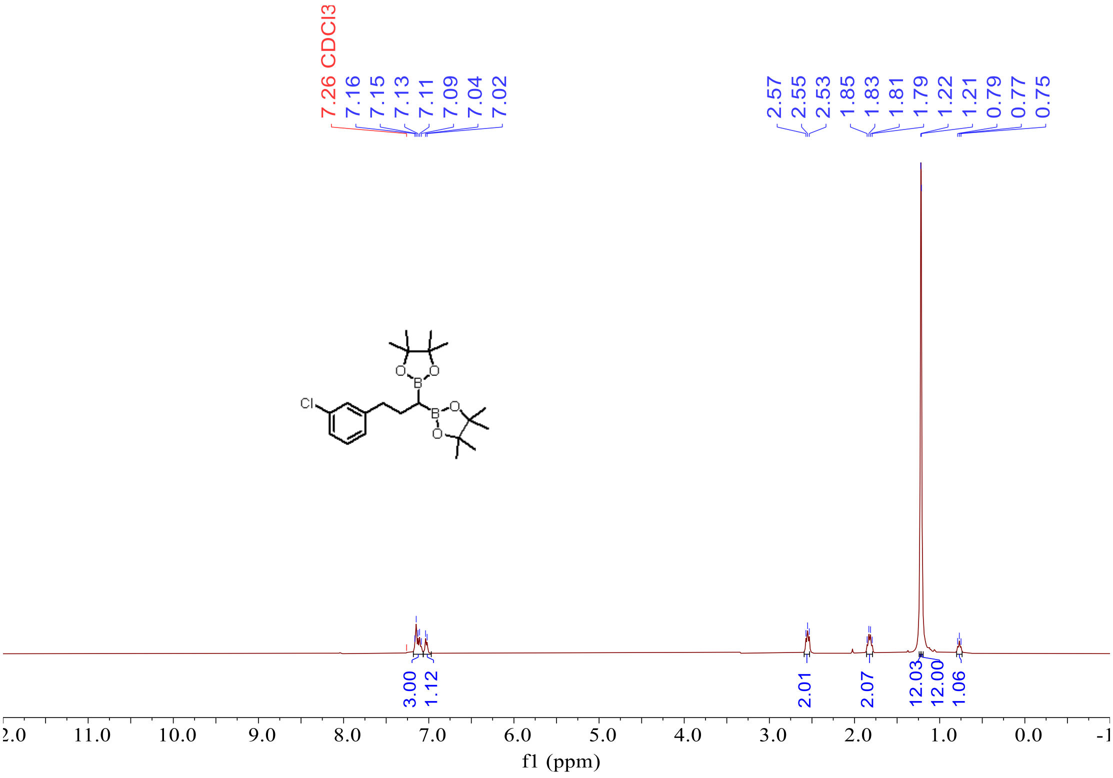

Supplementary Figure 97. ^{1}H NMR (400 MHz, $CDCl_3$) spectra for compound **13**

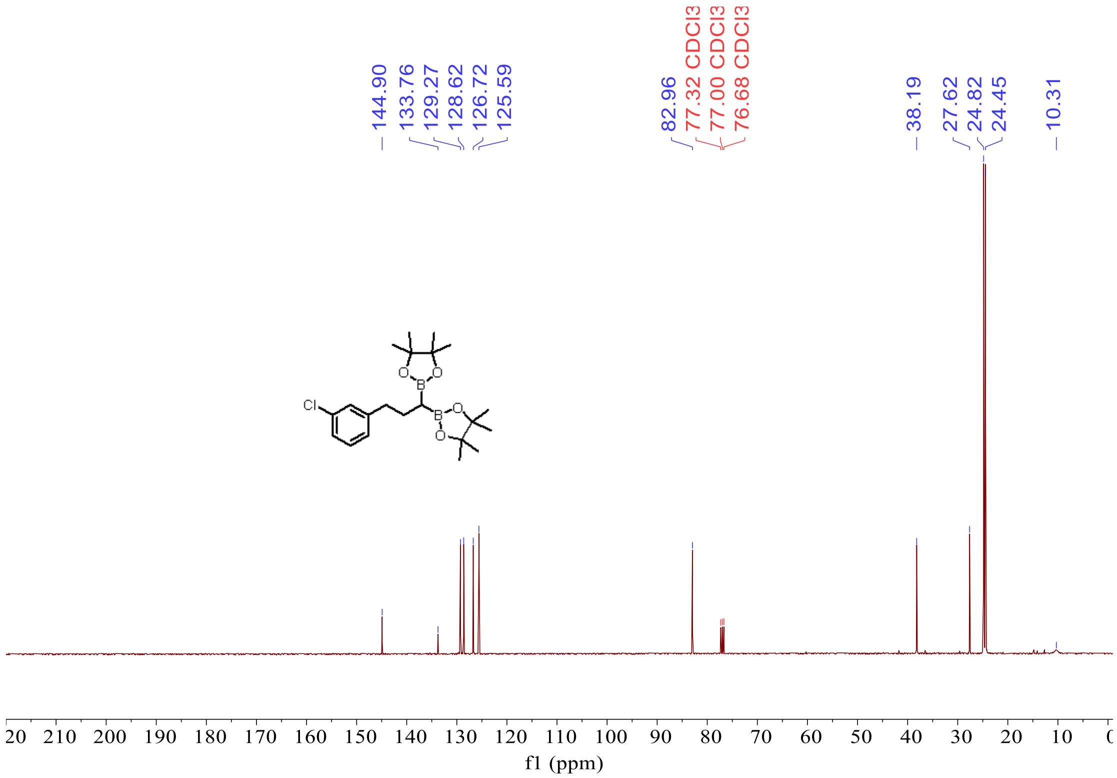

Supplementary Figure 98. ^{13}C NMR (101 MHz, $CDCl_3$) spectra for compound **13**

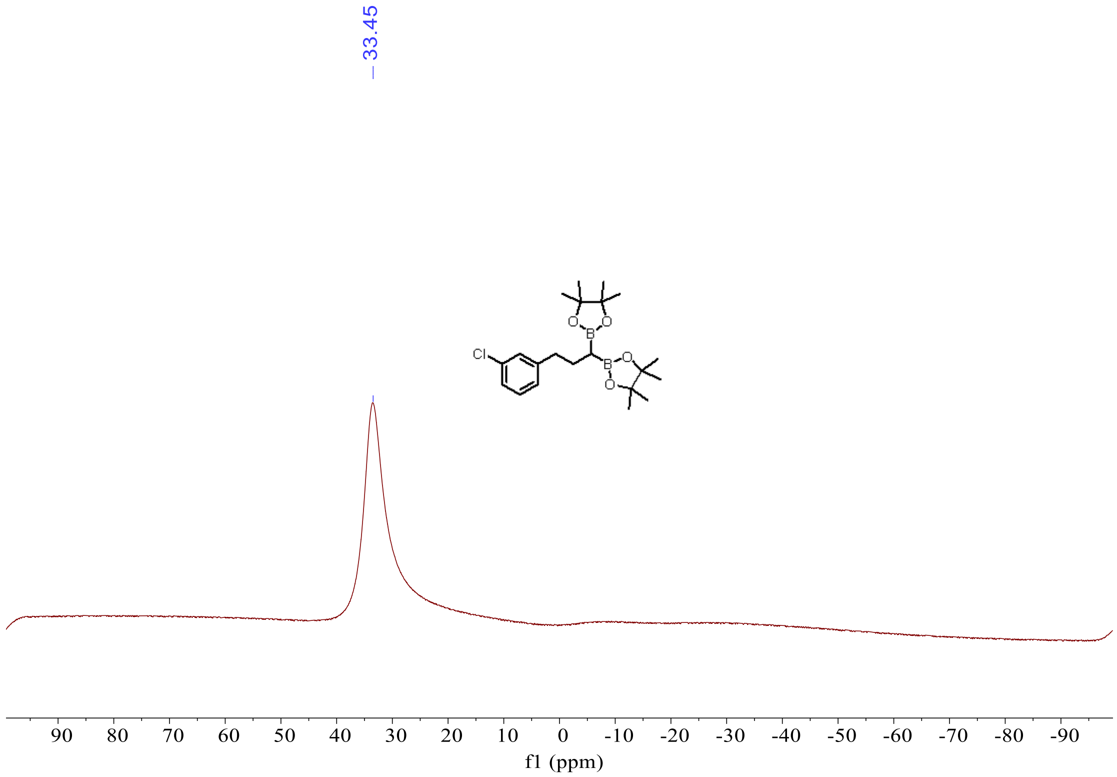

Supplementary Figure 99. ^{11}B NMR (128 MHz, $CDCl_3$) spectra for compound **13**

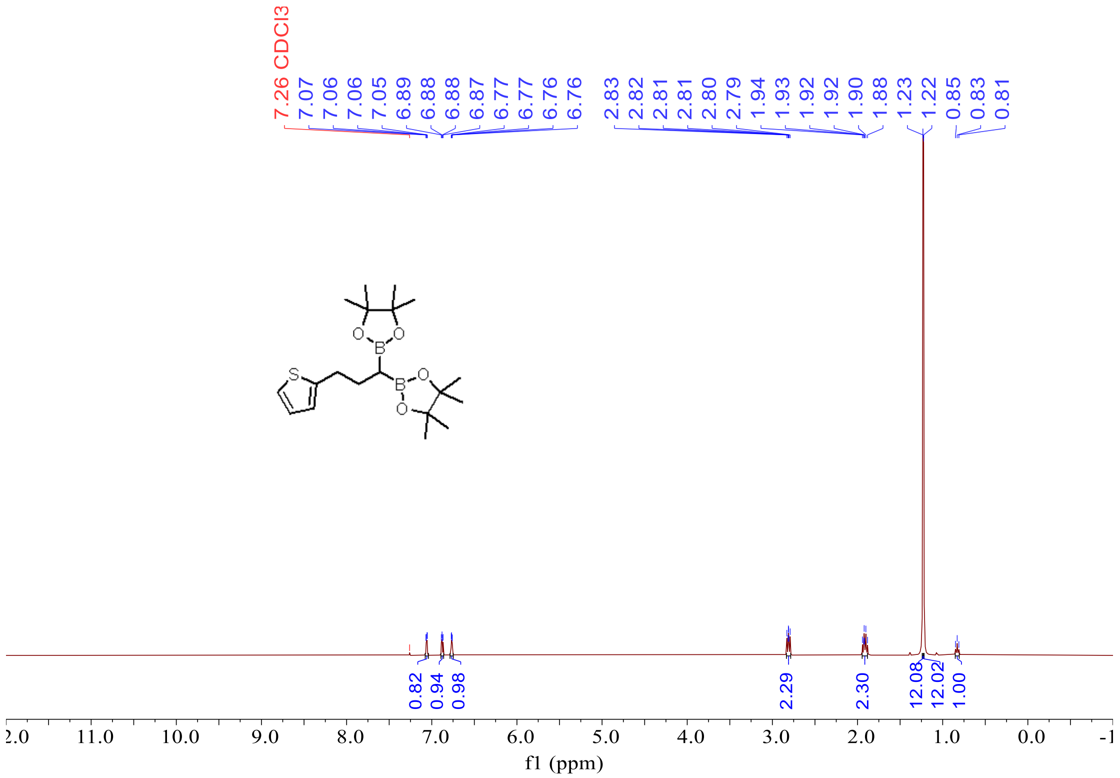

Supplementary Figure 100. ^{1}H NMR (400 MHz, $CDCl_3$) spectra for compound **14**

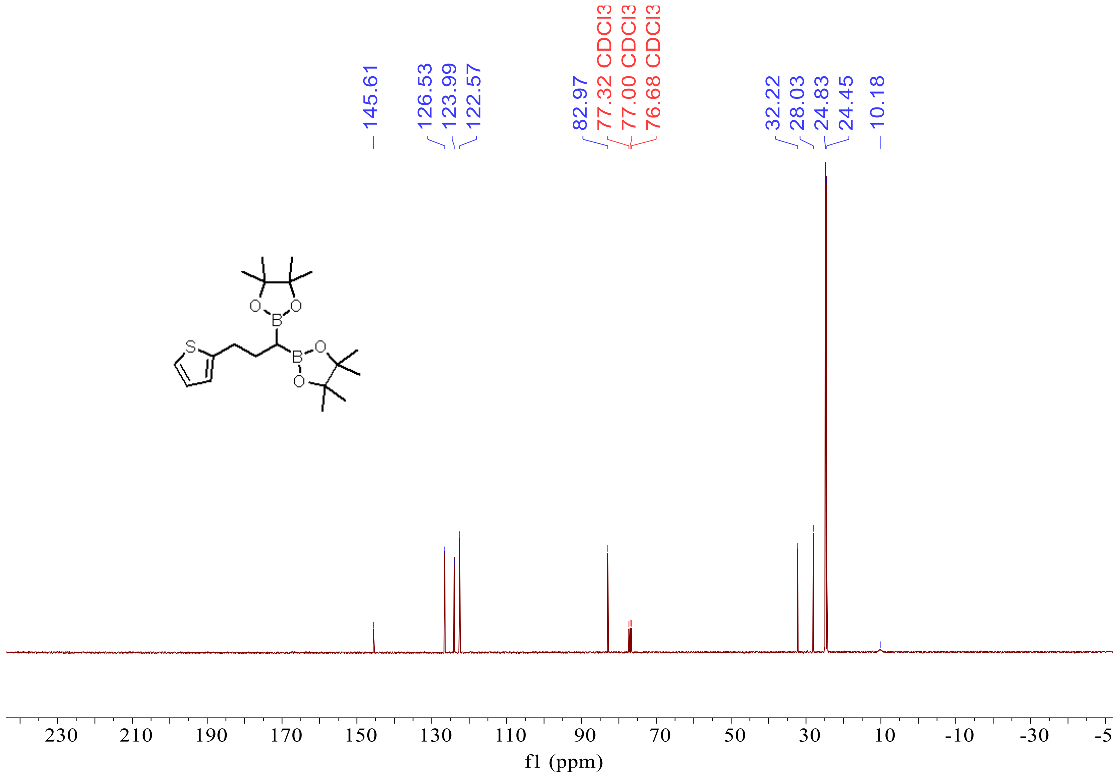

Supplementary Figure 101. ^{13}C NMR (101 MHz, $CDCl_3$) spectra for compound **14**

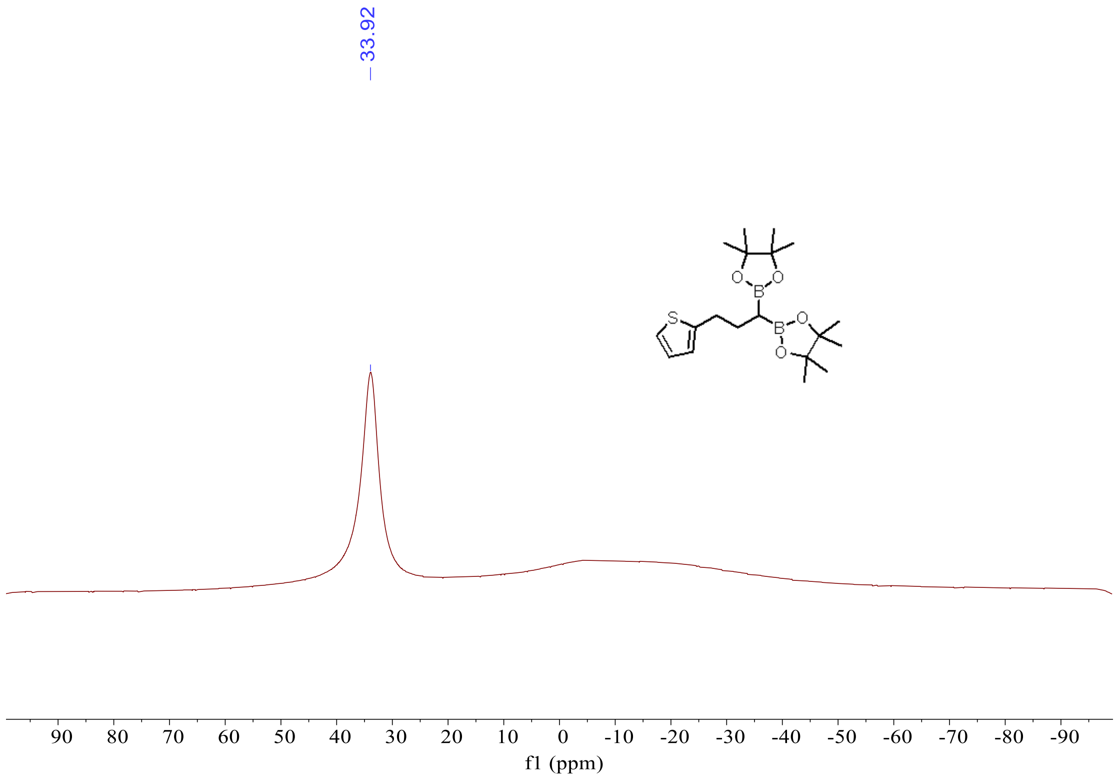

Supplementary Figure 102. ^{11}B NMR (128 MHz, $CDCl_3$) spectra for compound **14**

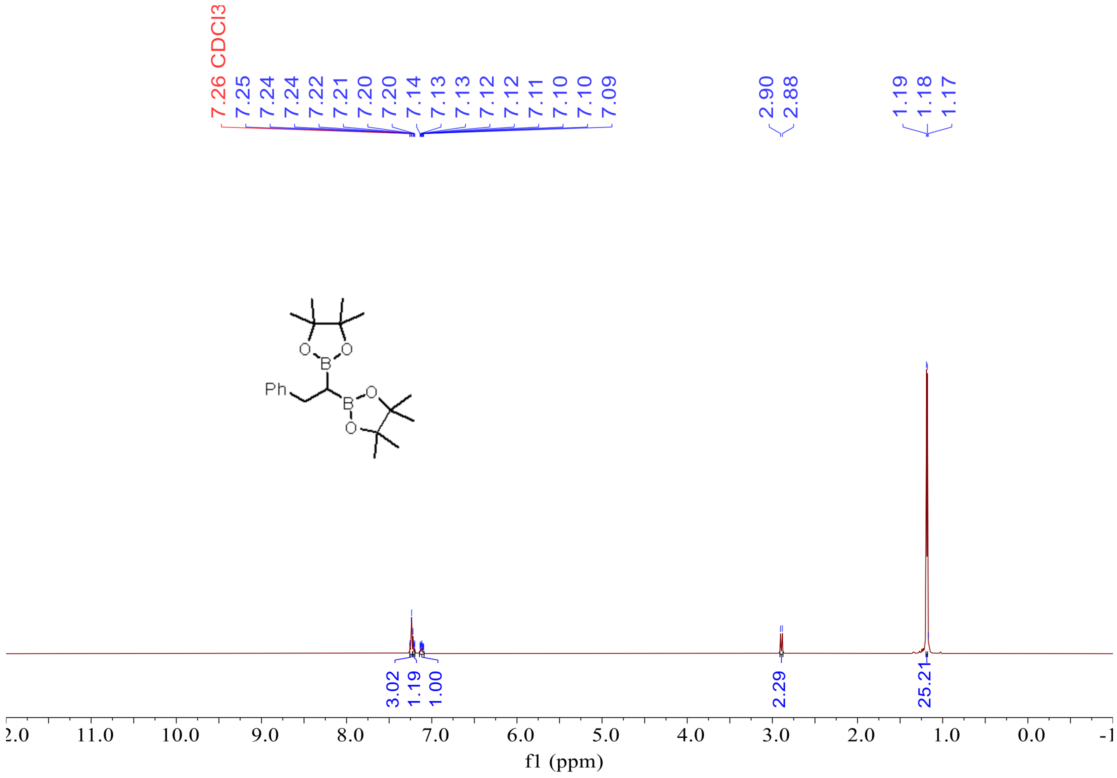

Supplementary Figure 103. ^{1}H NMR (400 MHz, $CDCl_3$) spectra for compound **15**

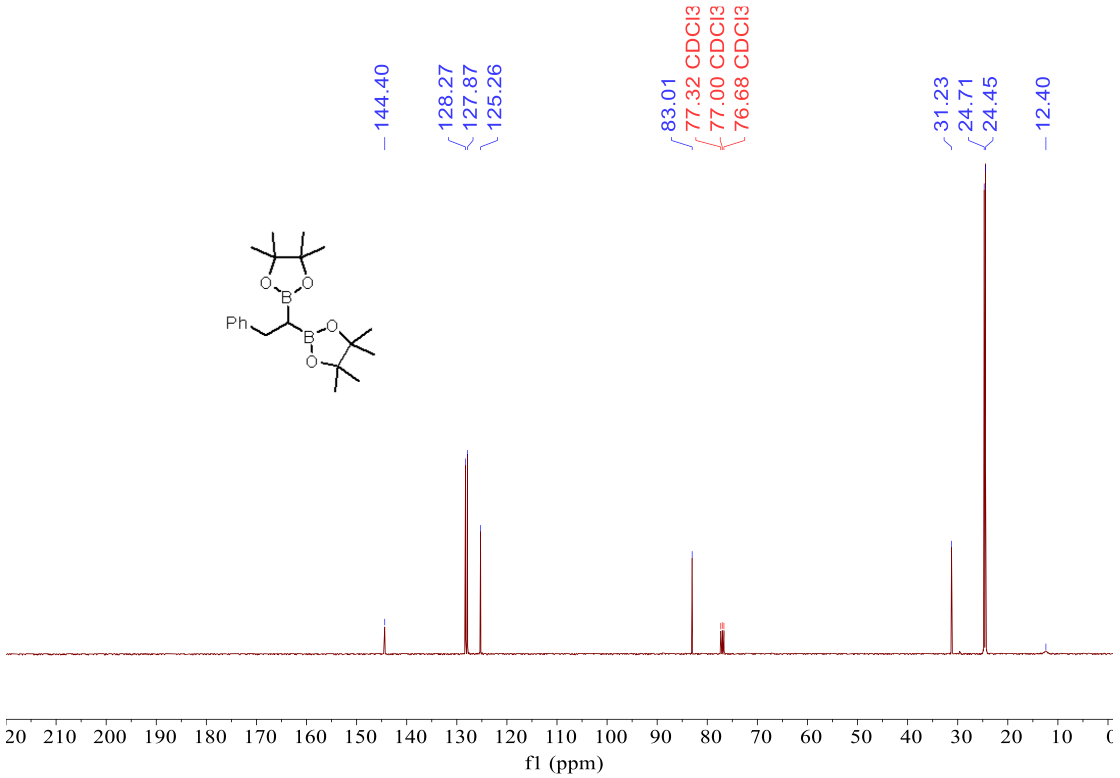

Supplementary Figure 104. ^{13}C NMR (101 MHz, $CDCl_3$) spectra for compound **15**

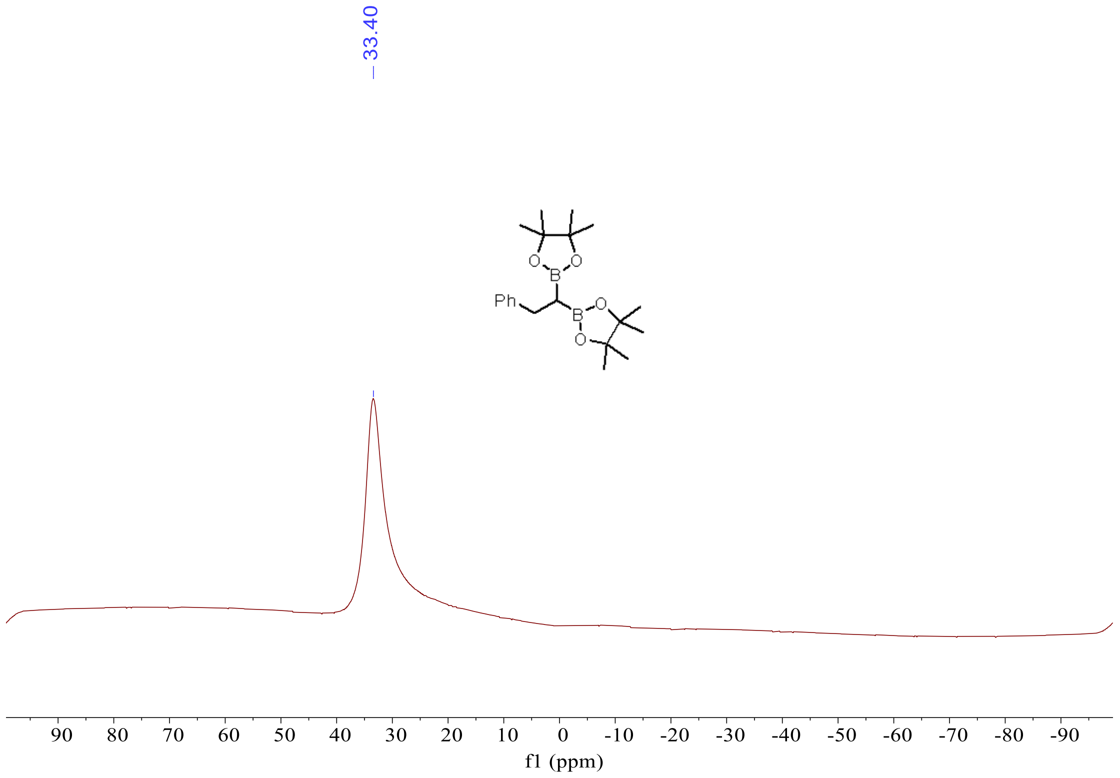

Supplementary Figure 105. ^{11}B NMR (128 MHz, $CDCl_3$) spectra for compound **15**

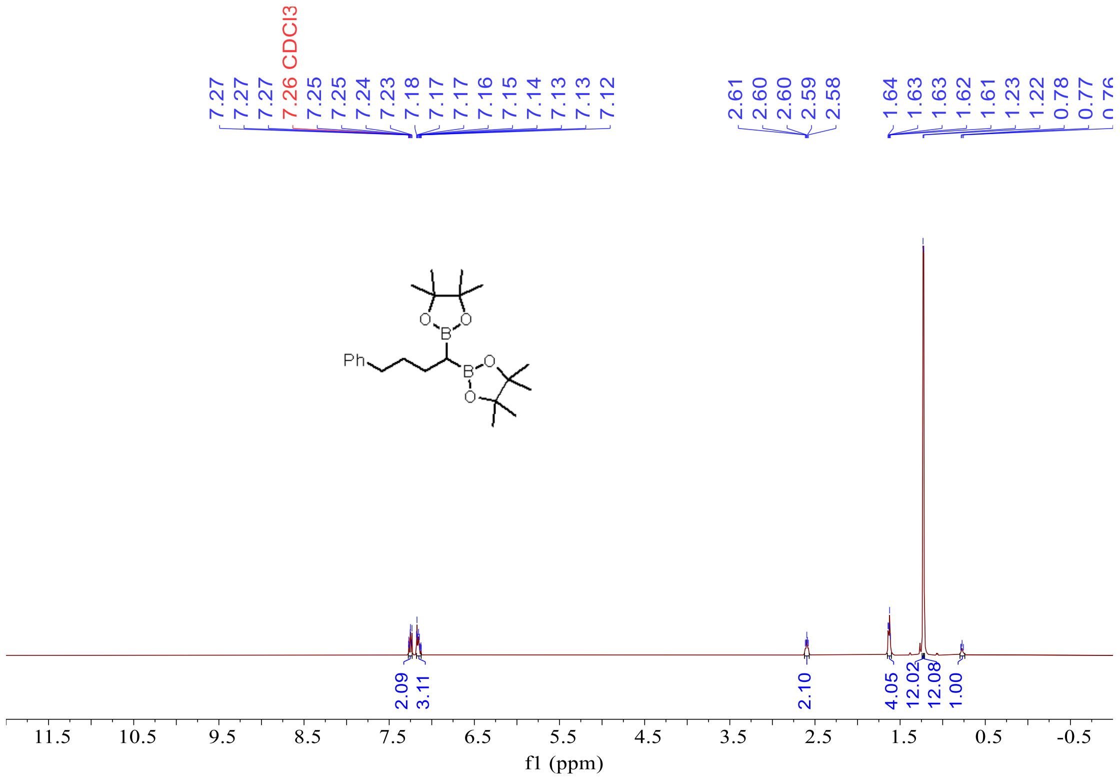

Supplementary Figure 106. ^{1}H NMR (400 MHz, $CDCl_3$) spectra for compound **16**

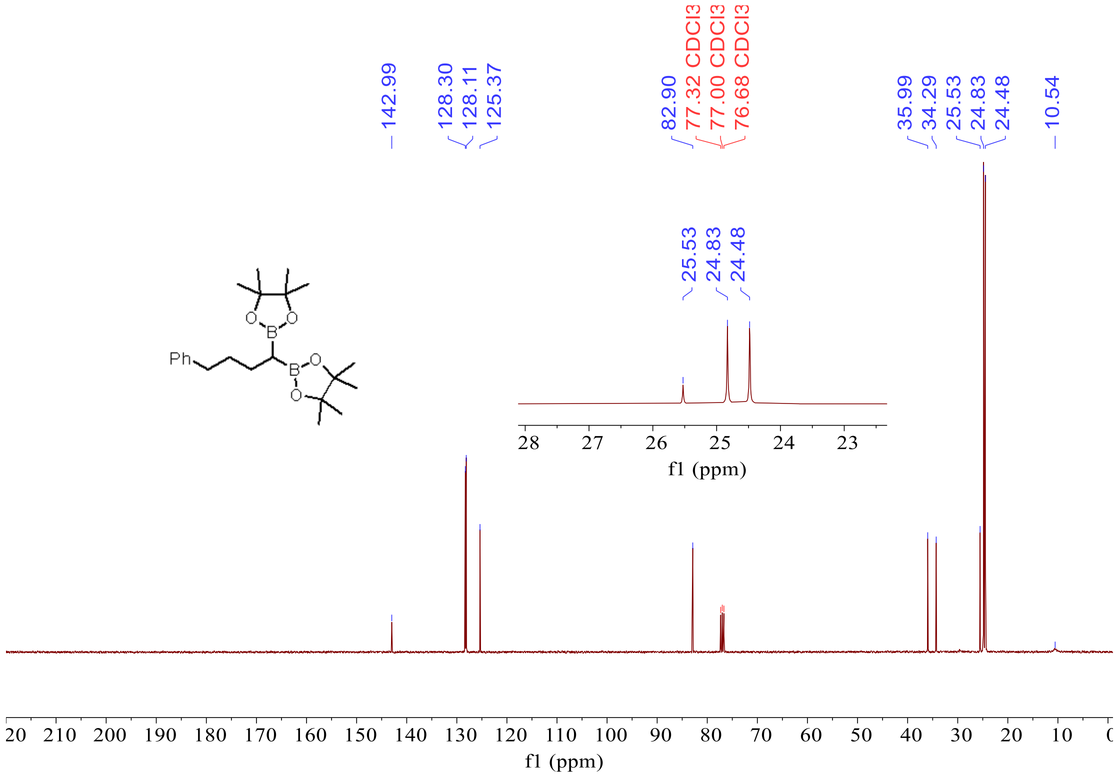

Supplementary Figure 107. ^{13}C NMR (101 MHz, $CDCl_3$) spectra for compound **16**

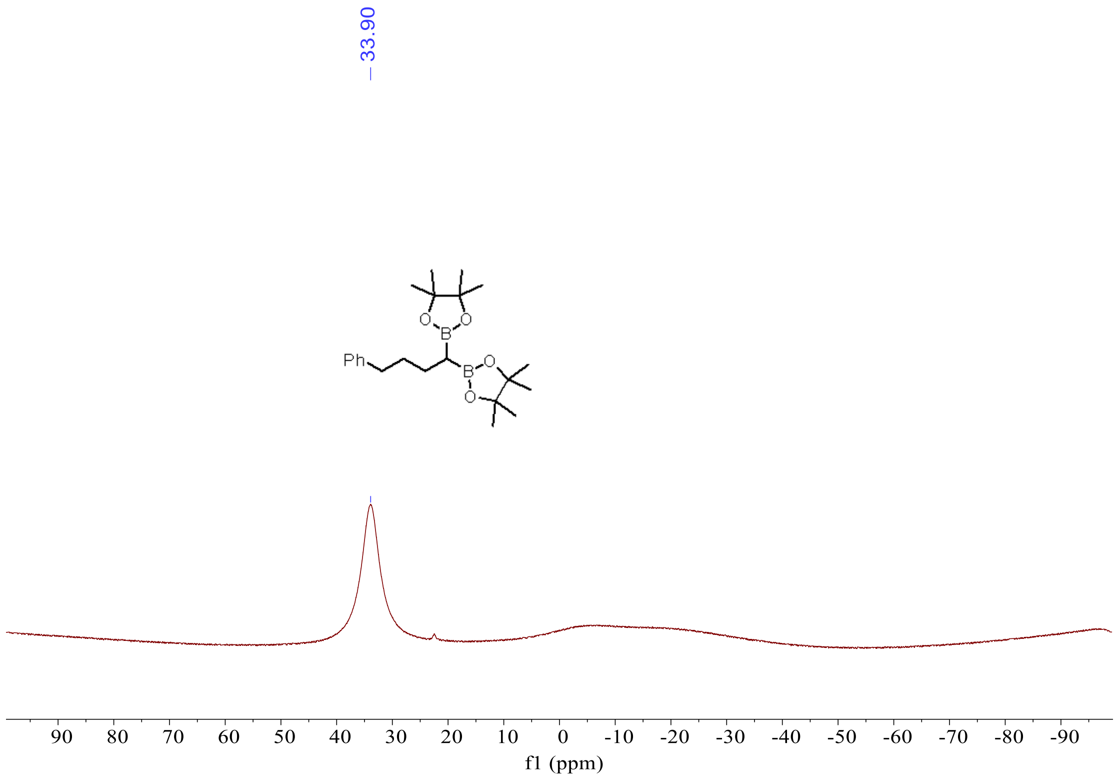

Supplementary Figure 108. ^{11}B NMR (128 MHz, $CDCl_3$) spectra for compound **16**

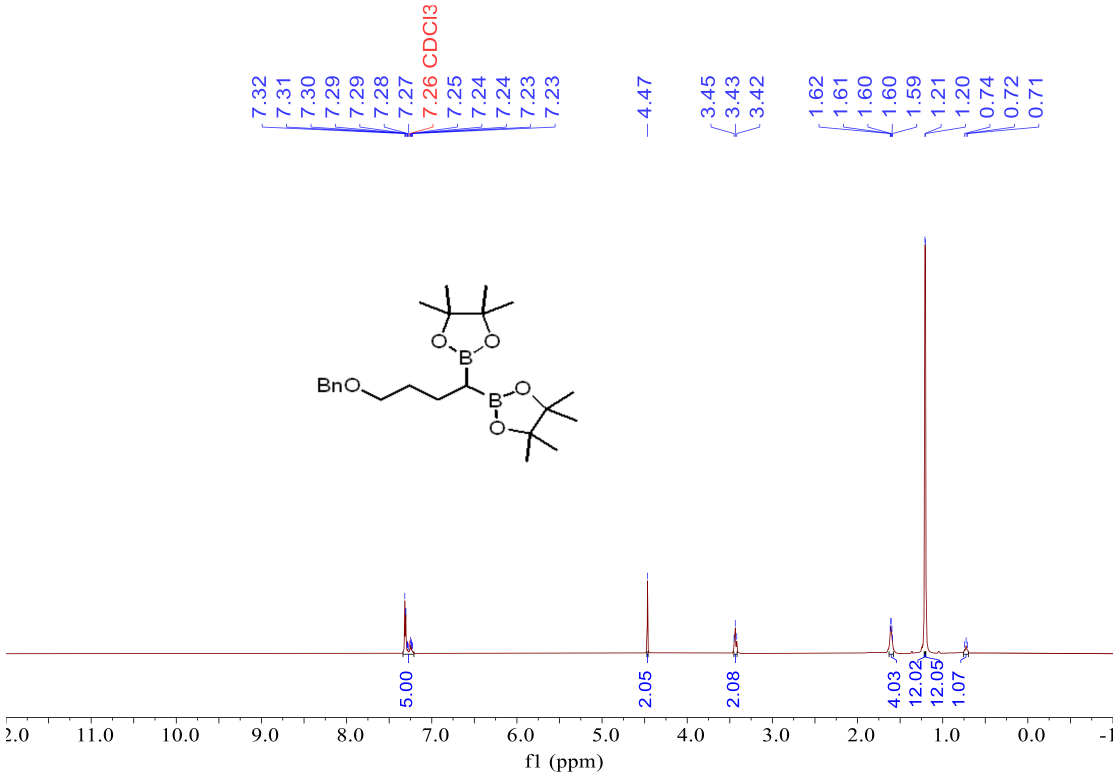

Supplementary Figure 109. ^{1}H NMR (400 MHz, $CDCl_3$) spectra for compound **17**

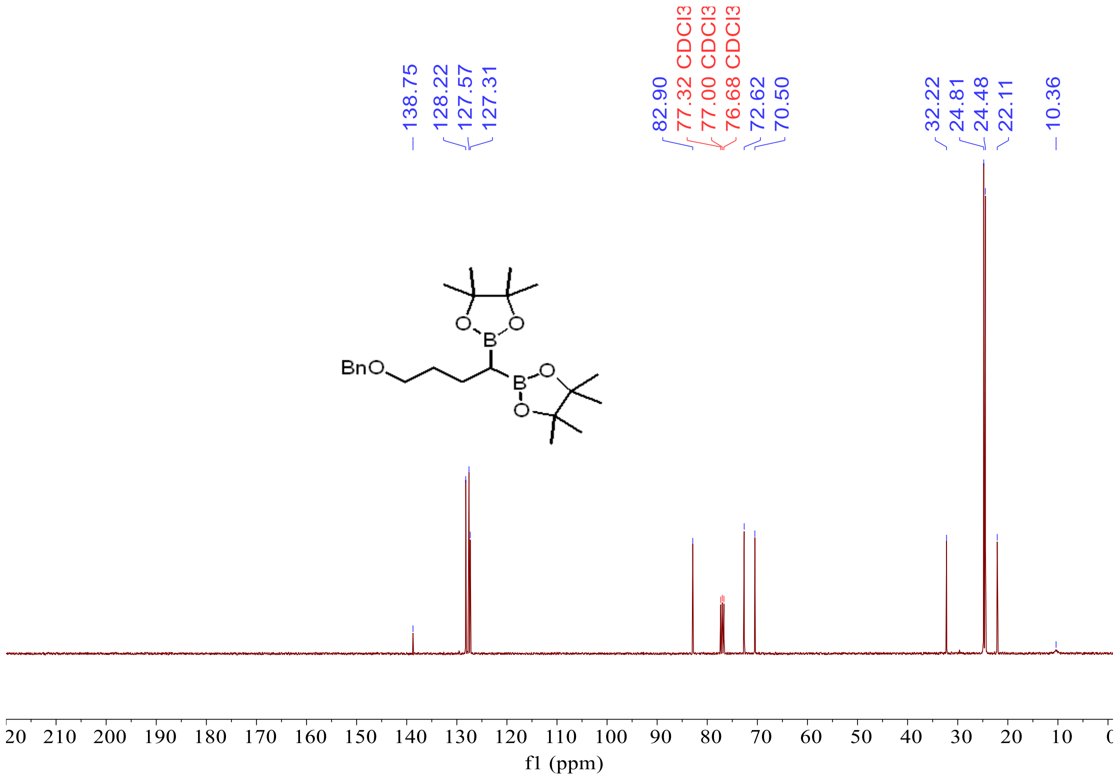

Supplementary Figure 110. ^{13}C NMR (101 MHz, $CDCl_3$) spectra for compound **17**

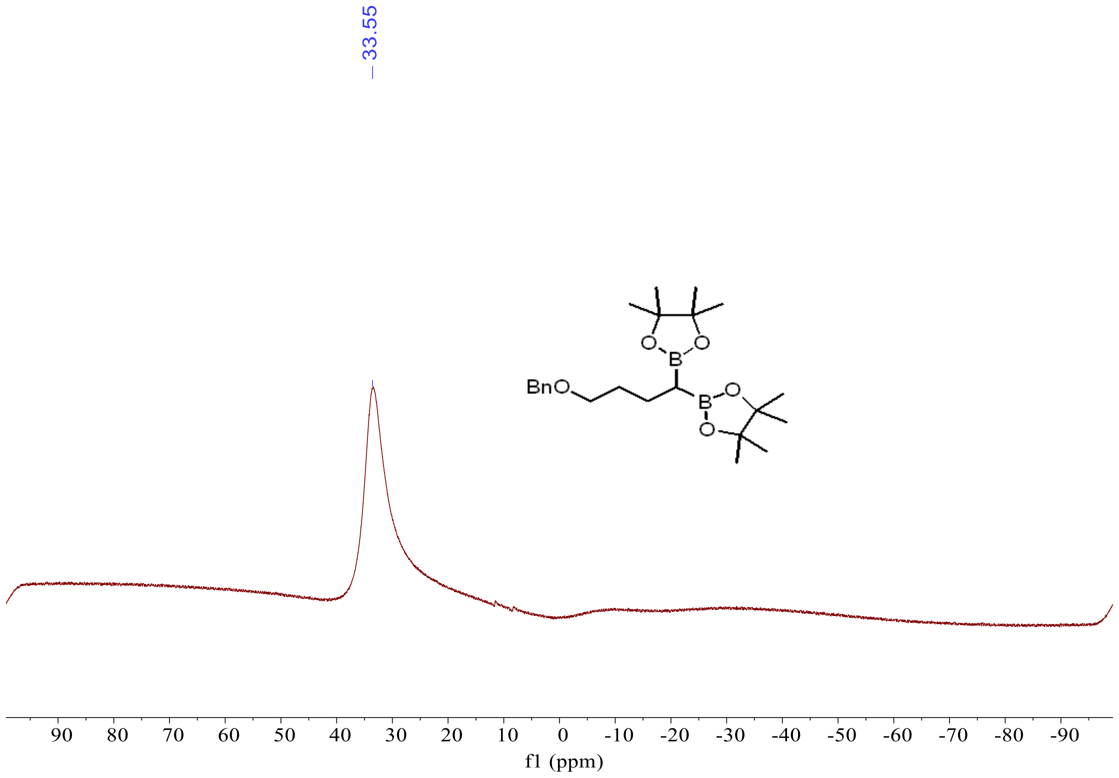

Supplementary Figure 111. ^{11}B NMR (128 MHz, $CDCl_3$) spectra for compound **17**

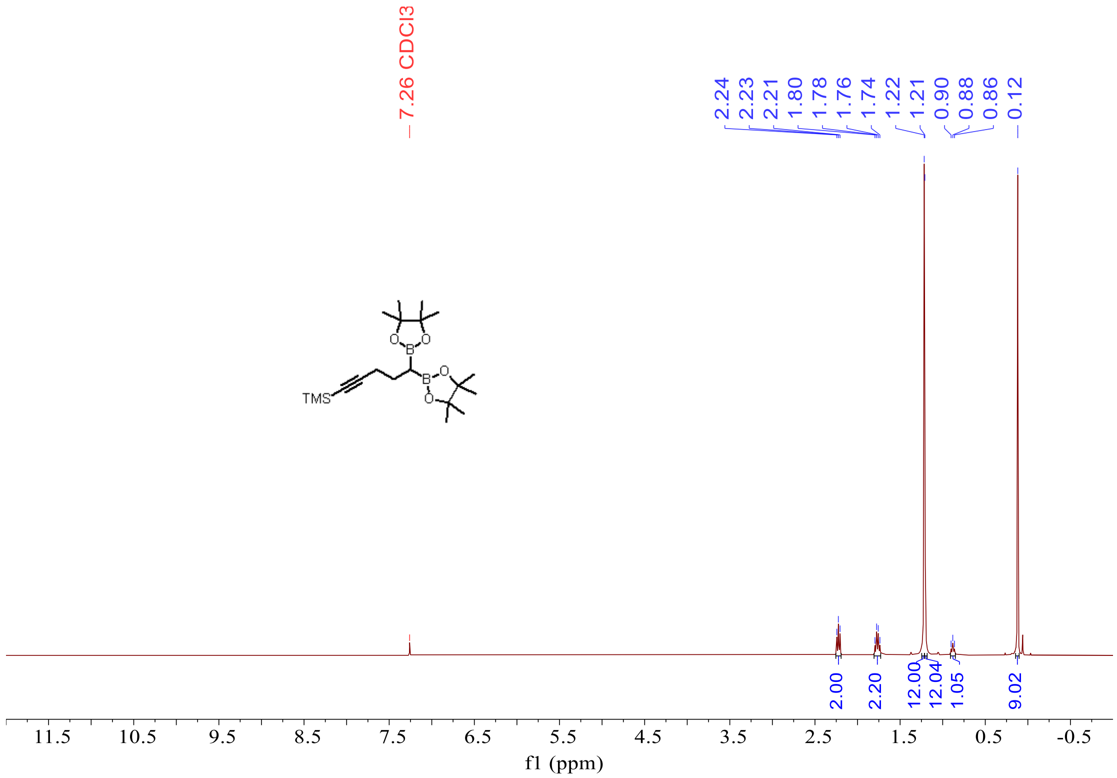

Supplementary Figure 112. ^{1}H NMR (400 MHz, $CDCl_3$) spectra for compound **18**

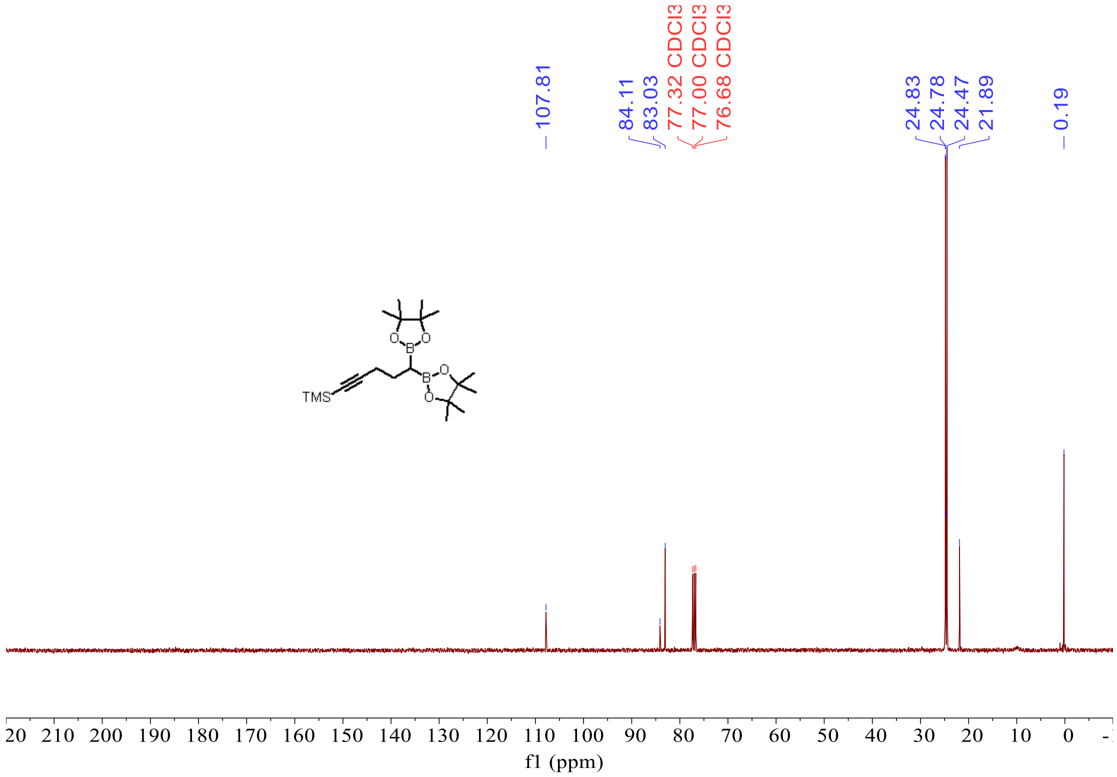

Supplementary Figure 113. ^{13}C NMR (101 MHz, $CDCl_3$) spectra for compound **18**

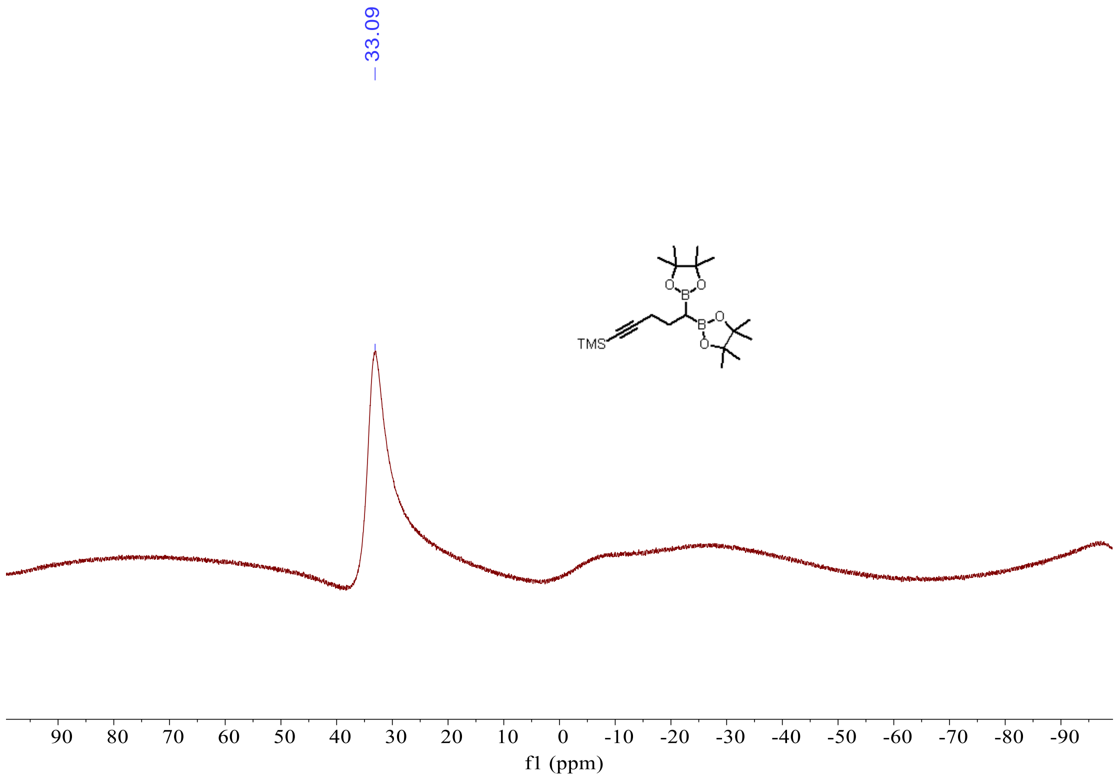

Supplementary Figure 114. ^{11}B NMR (128 MHz, $CDCl_3$) spectra for compound **18**

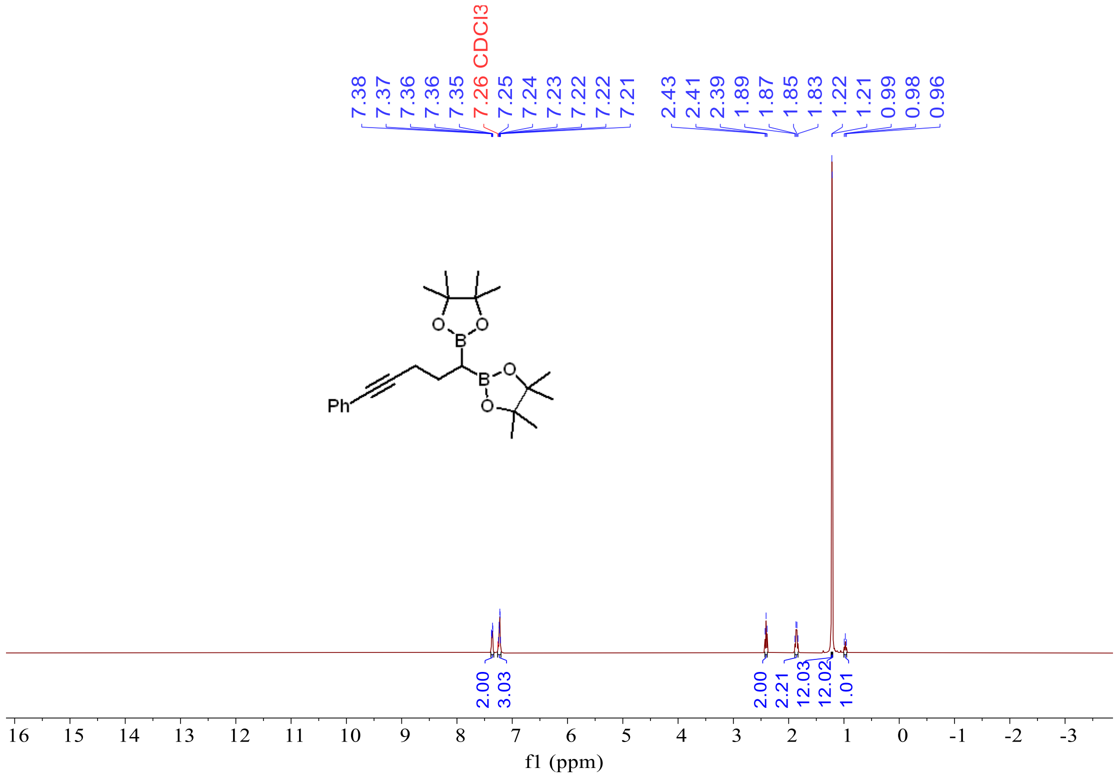

Supplementary Figure 115. ^{1}H NMR (400 MHz, $CDCl_3$) spectra for compound **19**

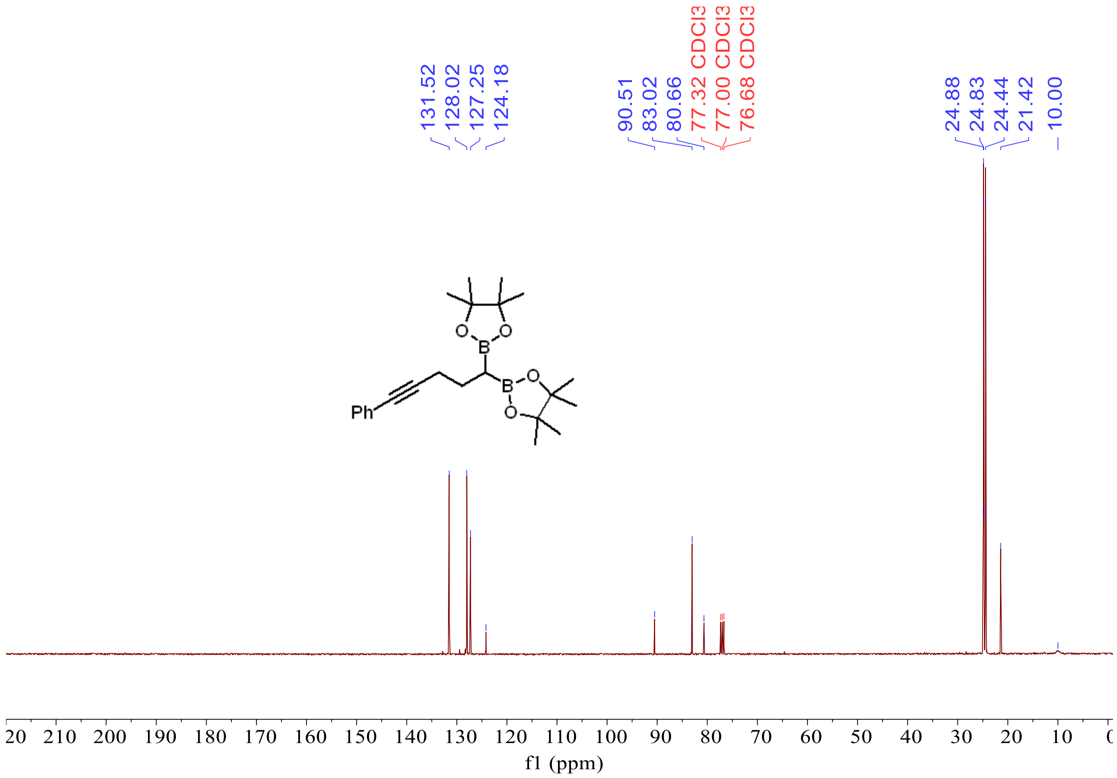

Supplementary Figure 116. ^{13}C NMR (101 MHz, $CDCl_3$) spectra for compound **19**

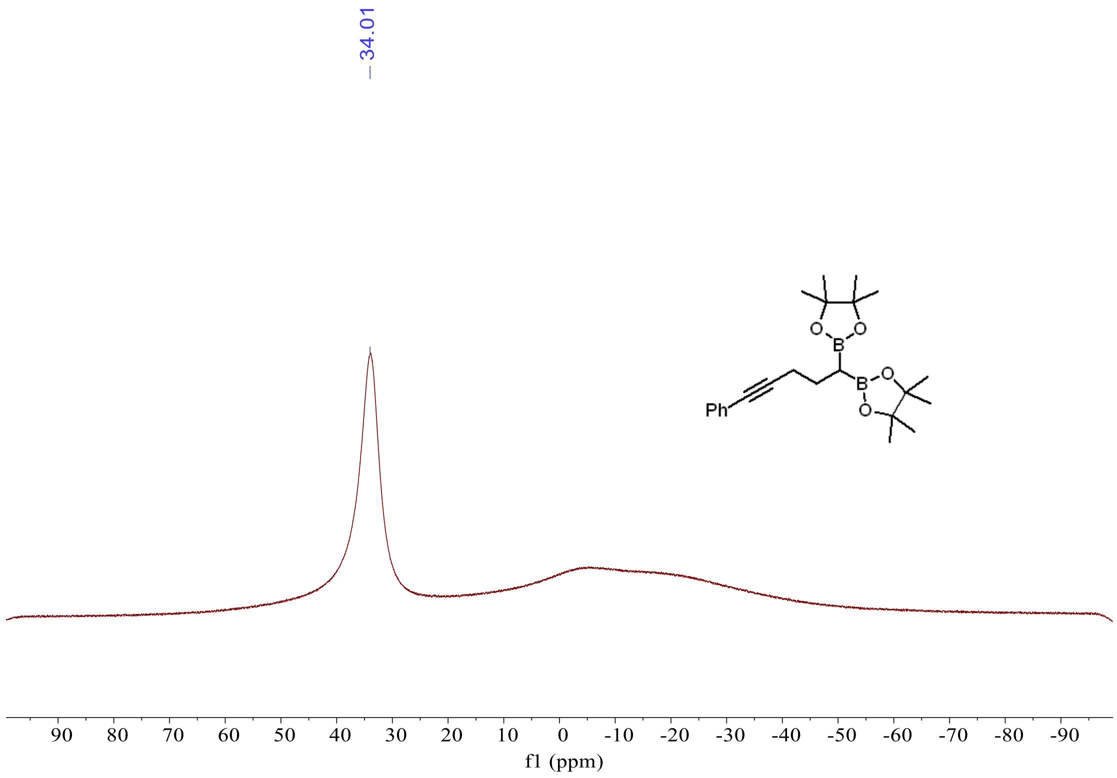

Supplementary Figure 117. ^{11}B NMR (128 MHz, $CDCl_3$) spectra for compound **19**

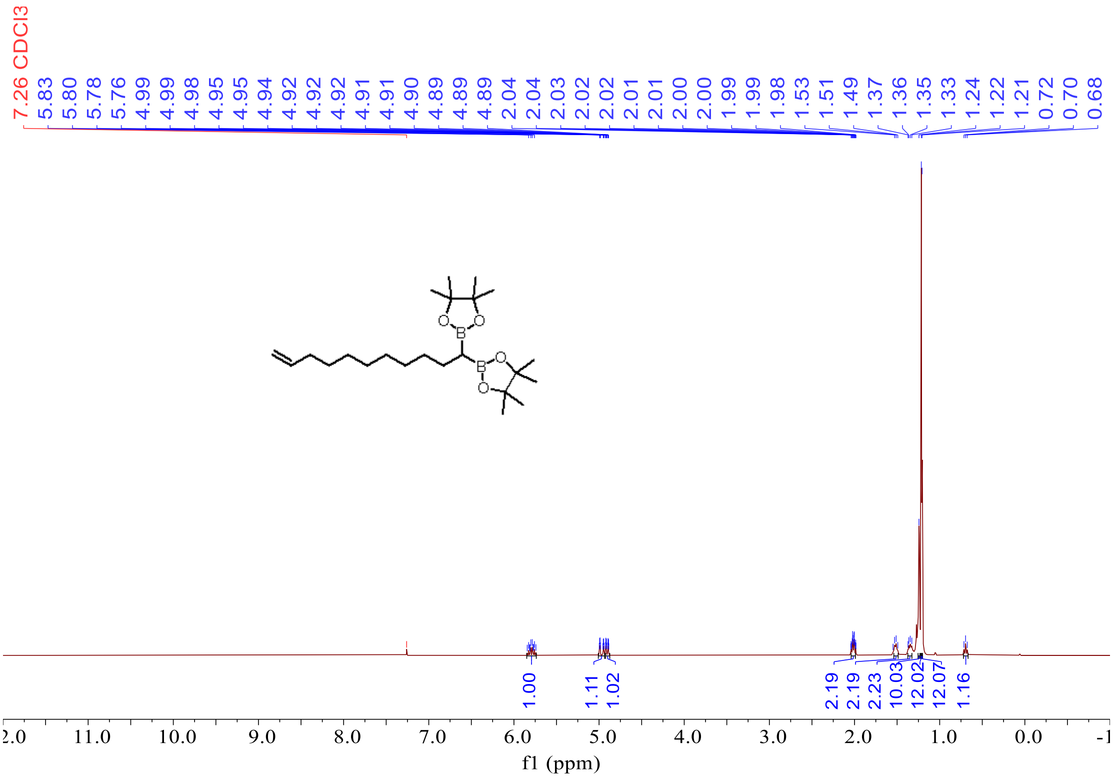

Supplementary Figure 118. ^{1}H NMR (400 MHz, $CDCl_3$) spectra for compound **20**

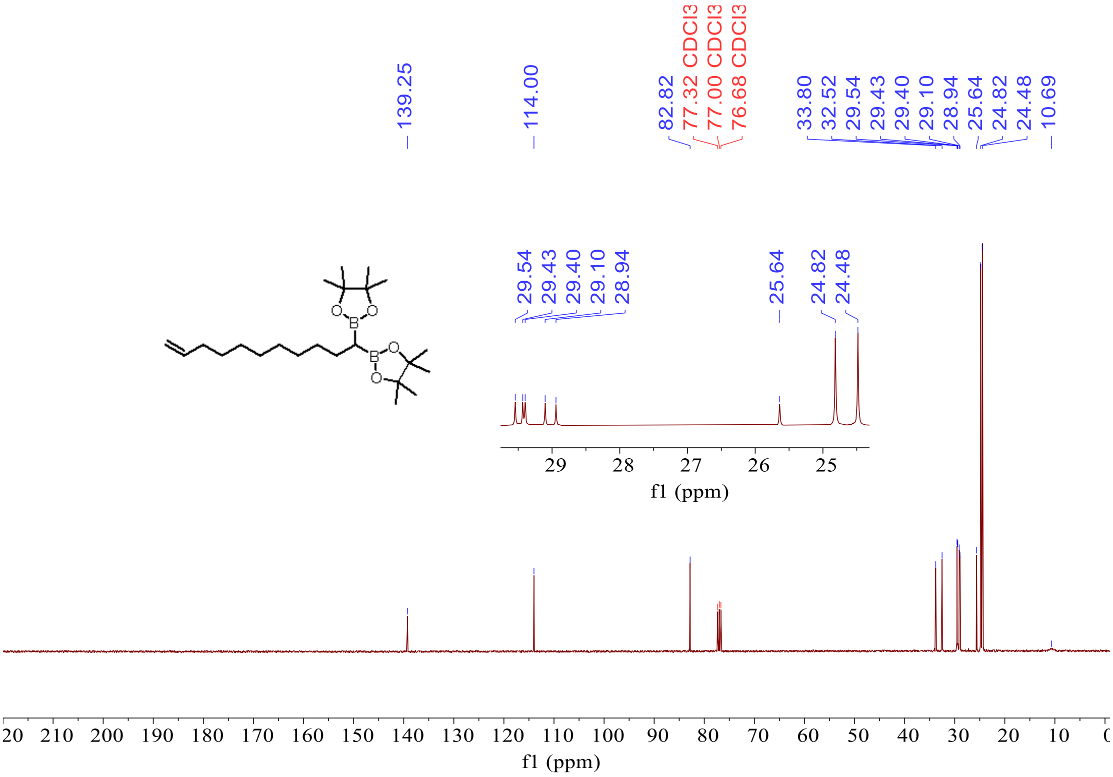

Supplementary Figure 119. ^{13}C NMR (101 MHz, $CDCl_3$) spectra for compound **20**

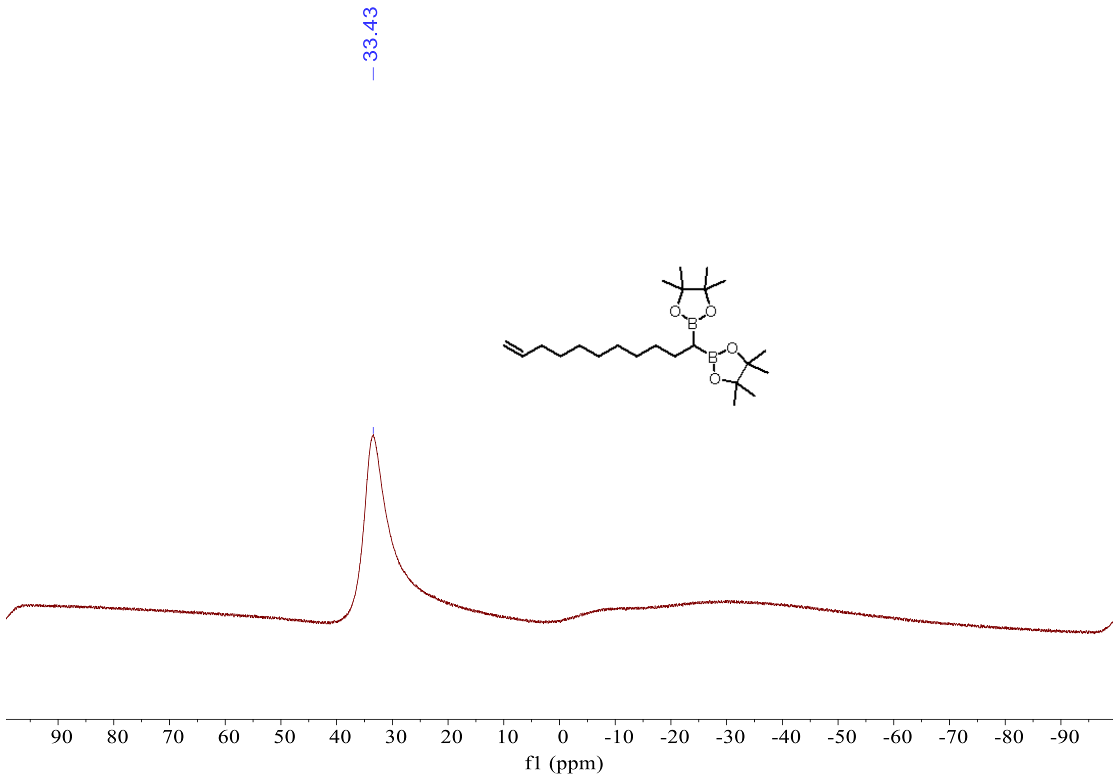

Supplementary Figure 120. ^{11}B NMR (128 MHz, $CDCl_3$) spectra for compound **20**

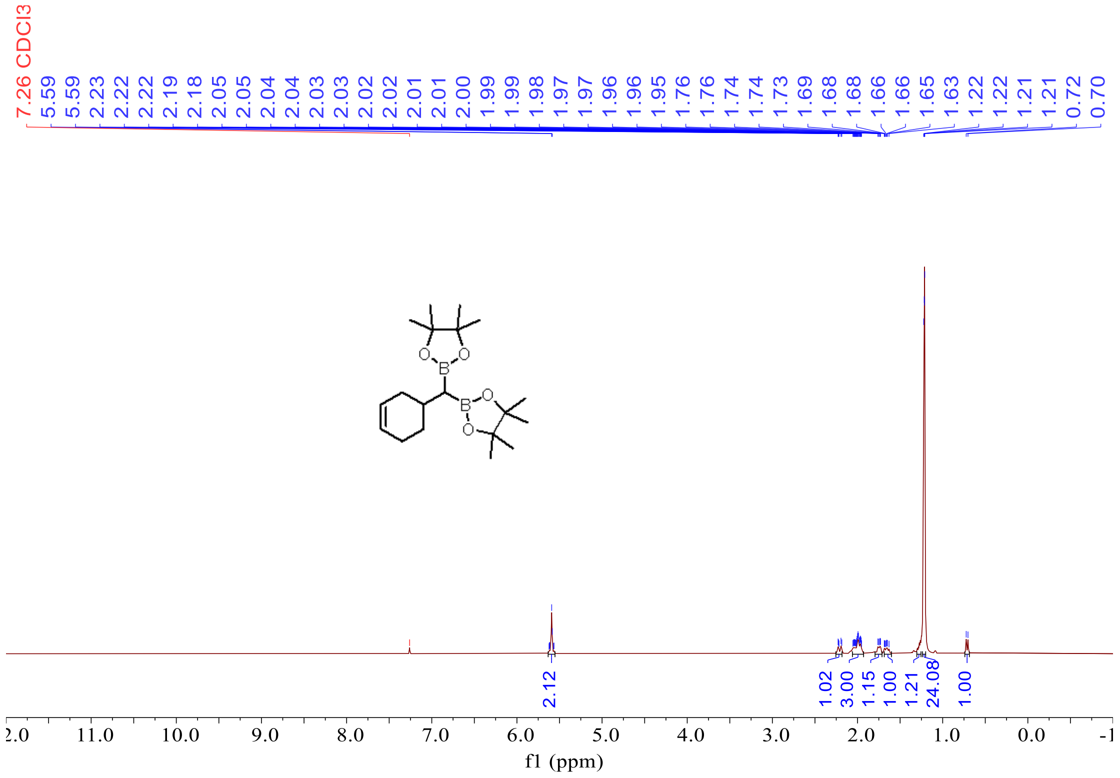

Supplementary Figure 121. ^{1}H NMR (500 MHz, $CDCl_3$) spectra for compound **21**

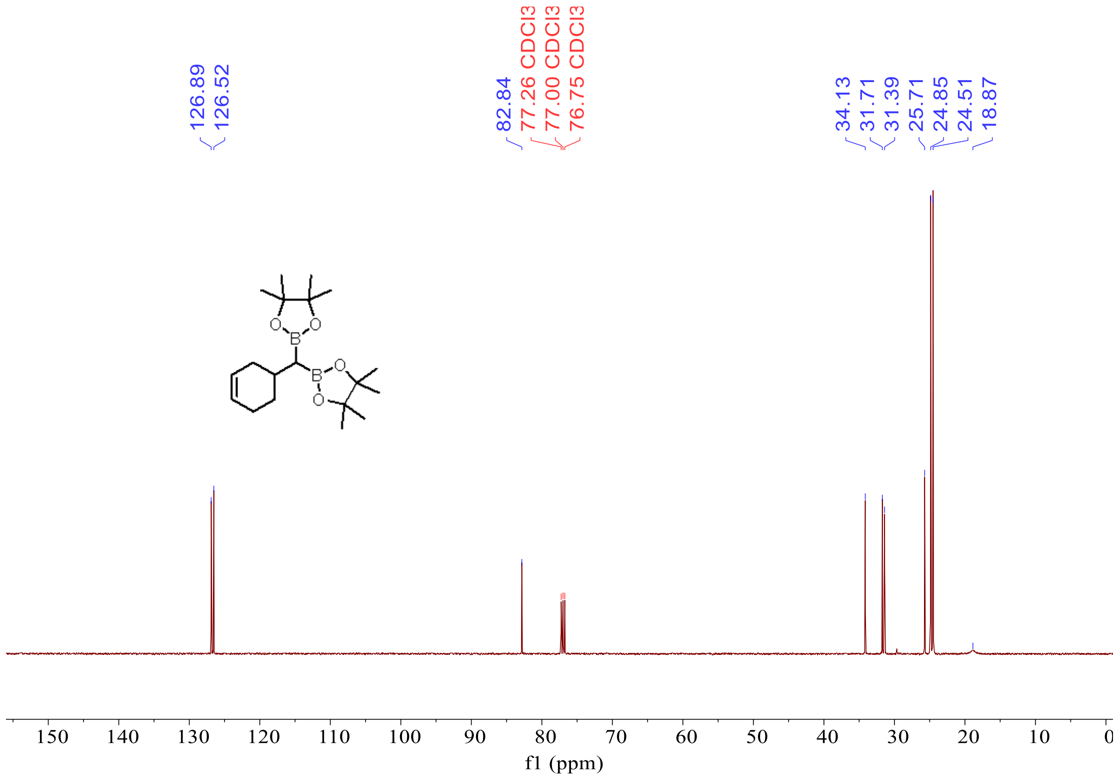

Supplementary Figure 122. ^{13}C NMR (126 MHz, $CDCl_3$) spectra for compound **21**

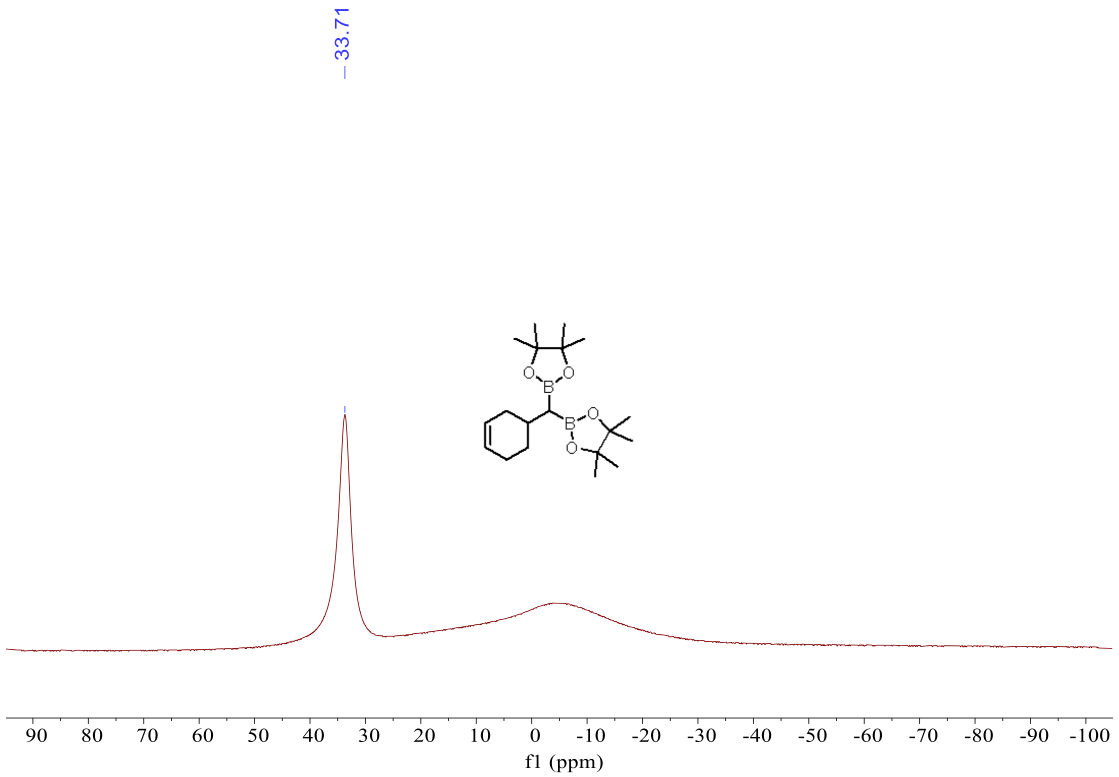

Supplementary Figure 123. ^{11}B NMR (160 MHz, $CDCl_3$) spectra for compound **21**

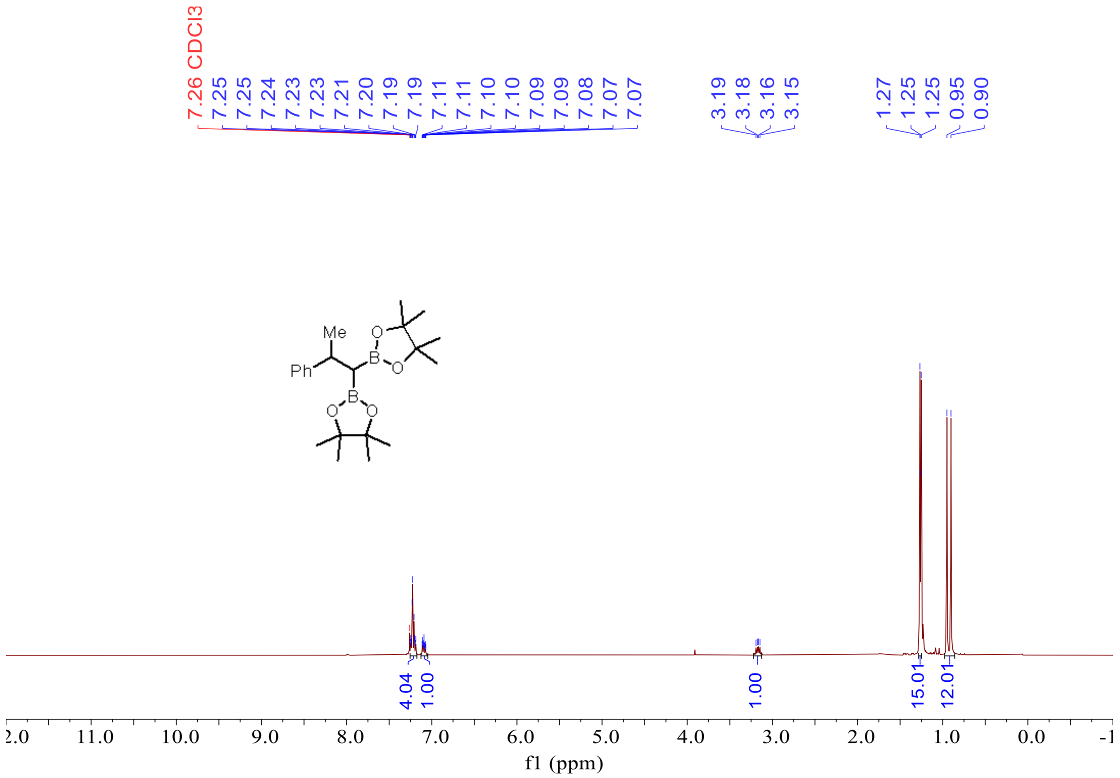

Supplementary Figure 124. ^{1}H NMR (400 MHz, $CDCl_3$) spectra for compound **22**

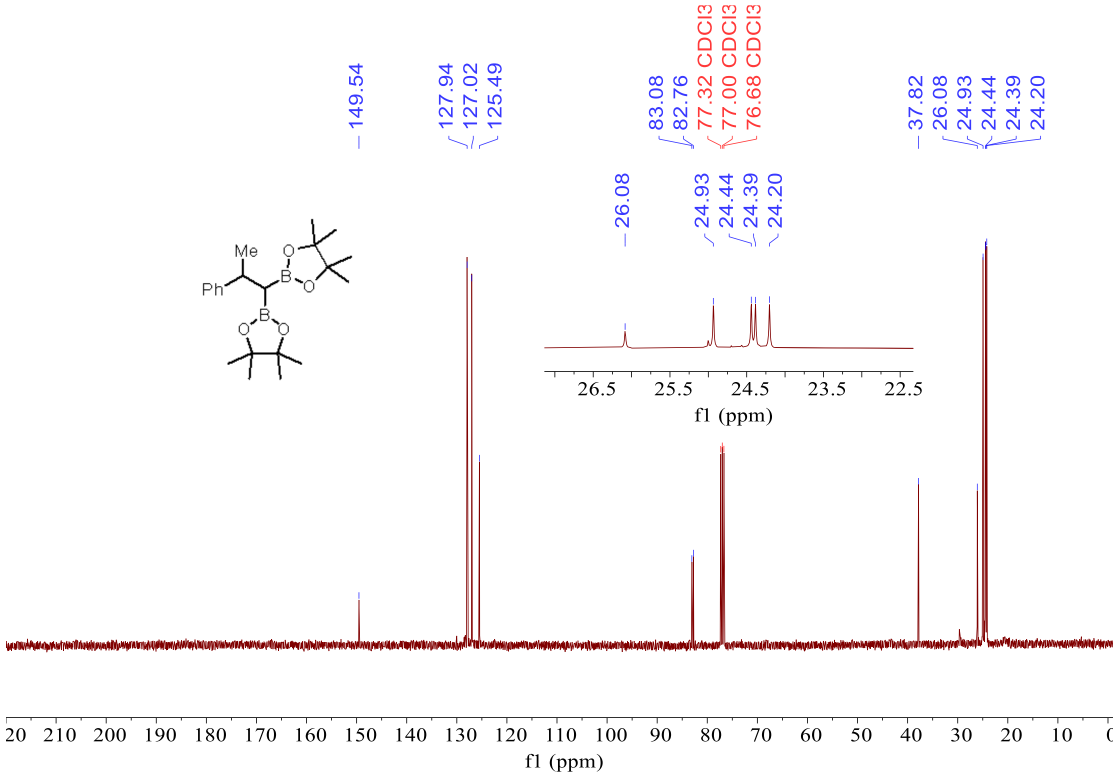

Supplementary Figure 125. ^{13}C NMR (101 MHz, $CDCl_3$) spectra for compound **22**

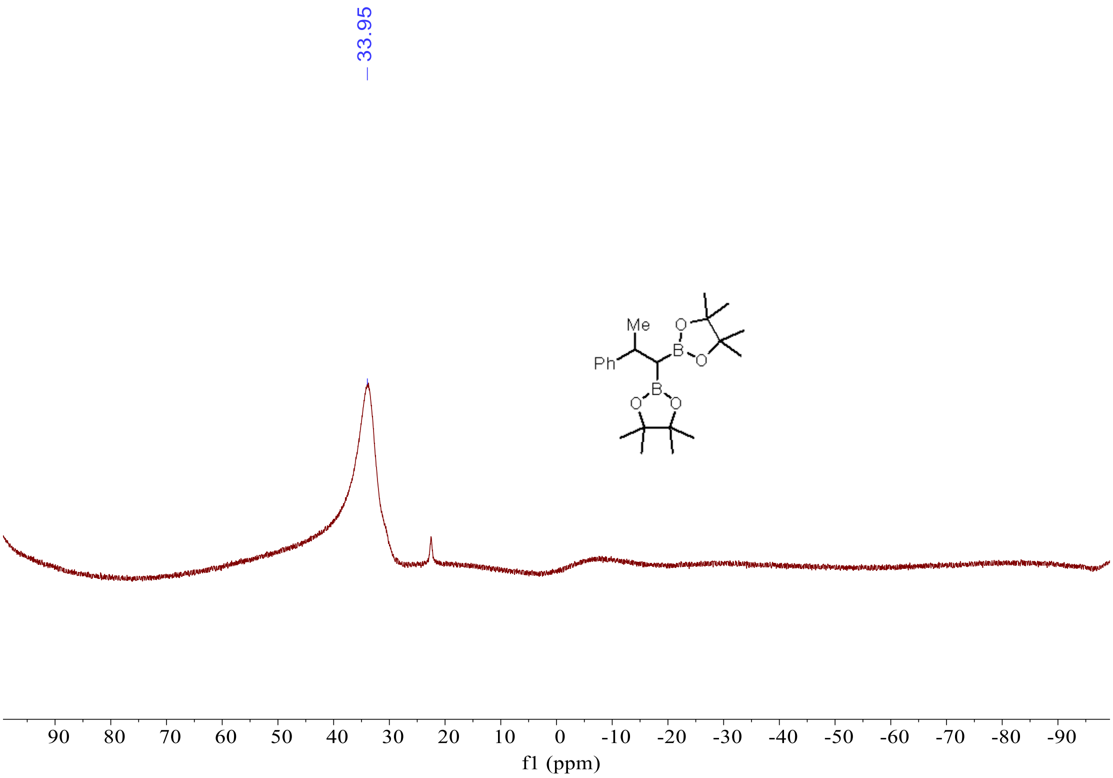

Supplementary Figure 126. ^{11}B NMR (128 MHz, $CDCl_3$) spectra for compound **22**

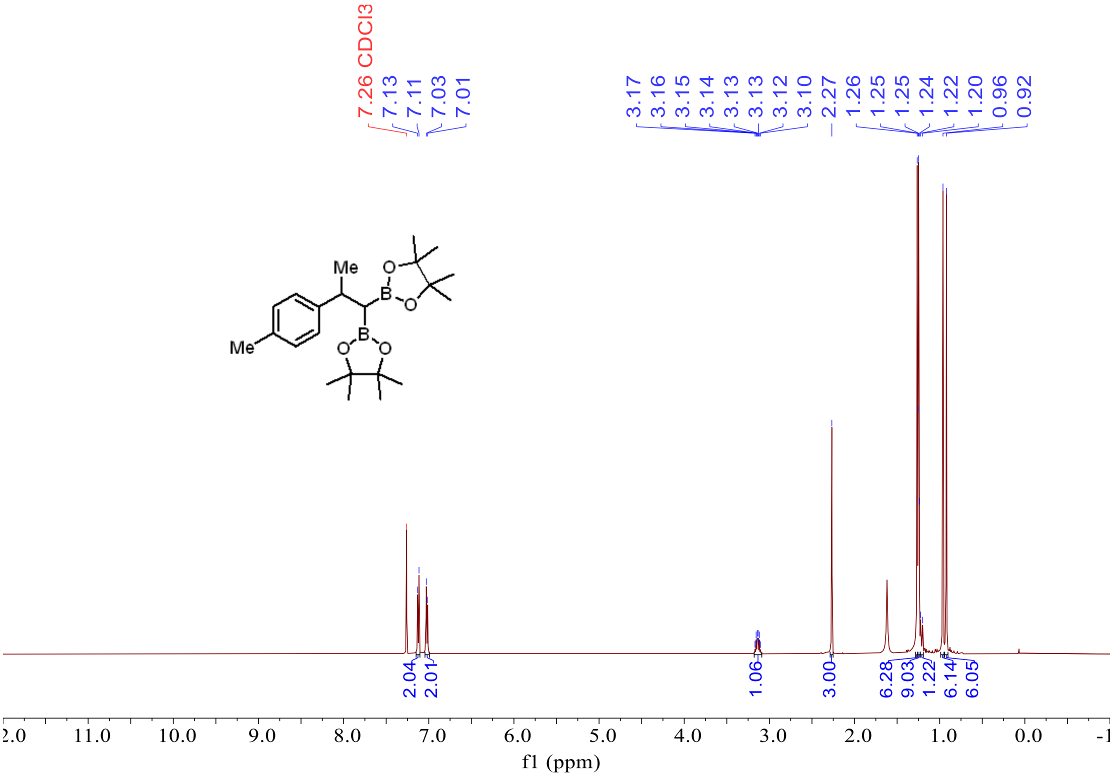

Supplementary Figure 127. ^{1}H NMR (500 MHz, $CDCl_3$) spectra for compound **23**

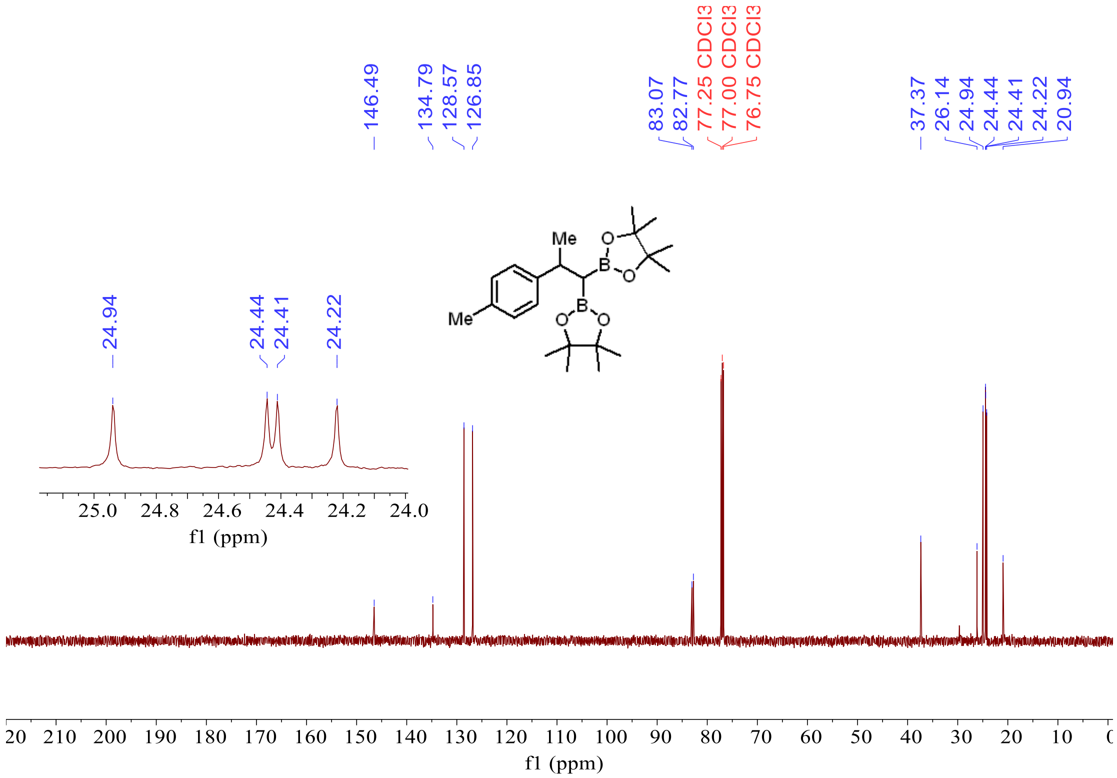

Supplementary Figure 128. ^{13}C NMR (126 MHz, $CDCl_3$) spectra for compound **23**

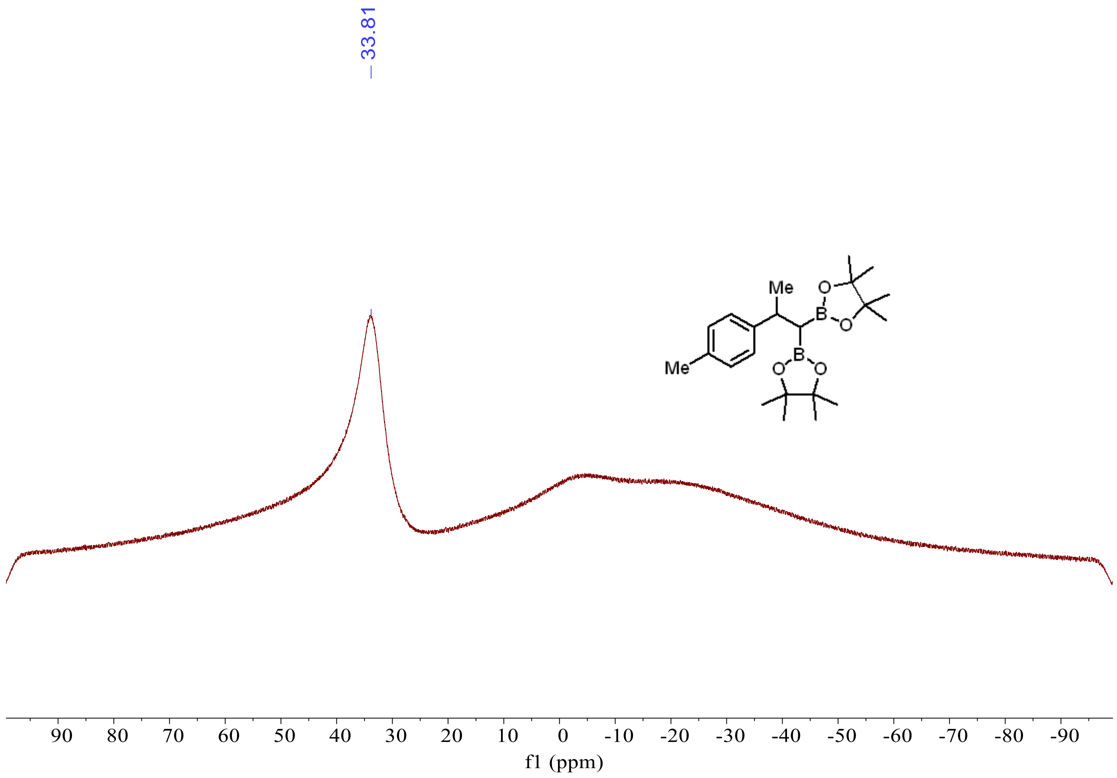

Supplementary Figure 129. ^{11}B NMR (128 MHz, $CDCl_3$) spectra for compound **23**

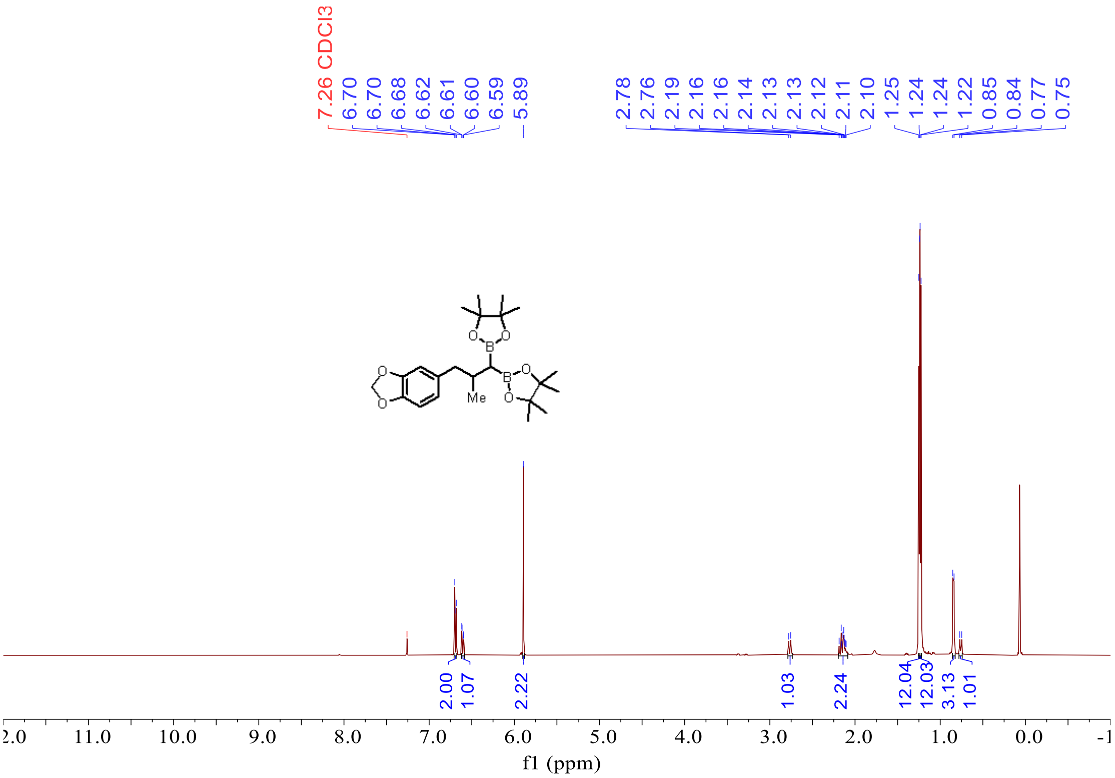

Supplementary Figure 130. ^{1}H NMR (400 MHz, $CDCl_3$) spectra for compound **24**

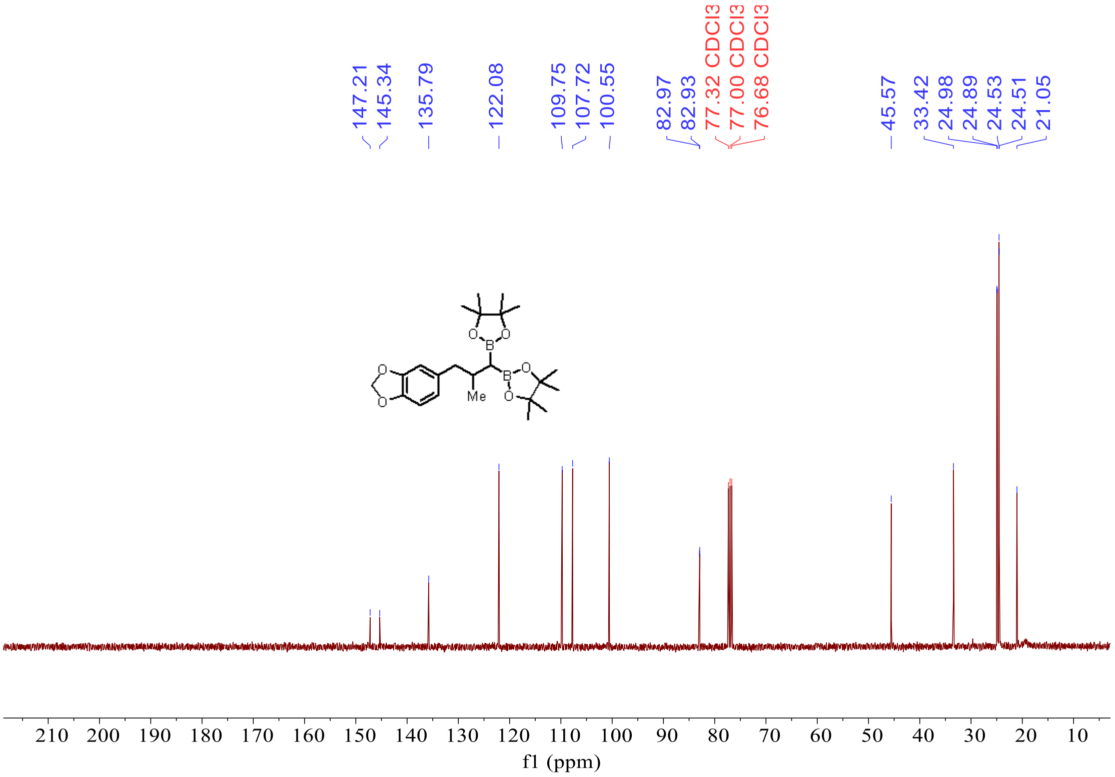

Supplementary Figure 131. ^{13}C NMR (101 MHz, $CDCl_3$) spectra for compound **24**

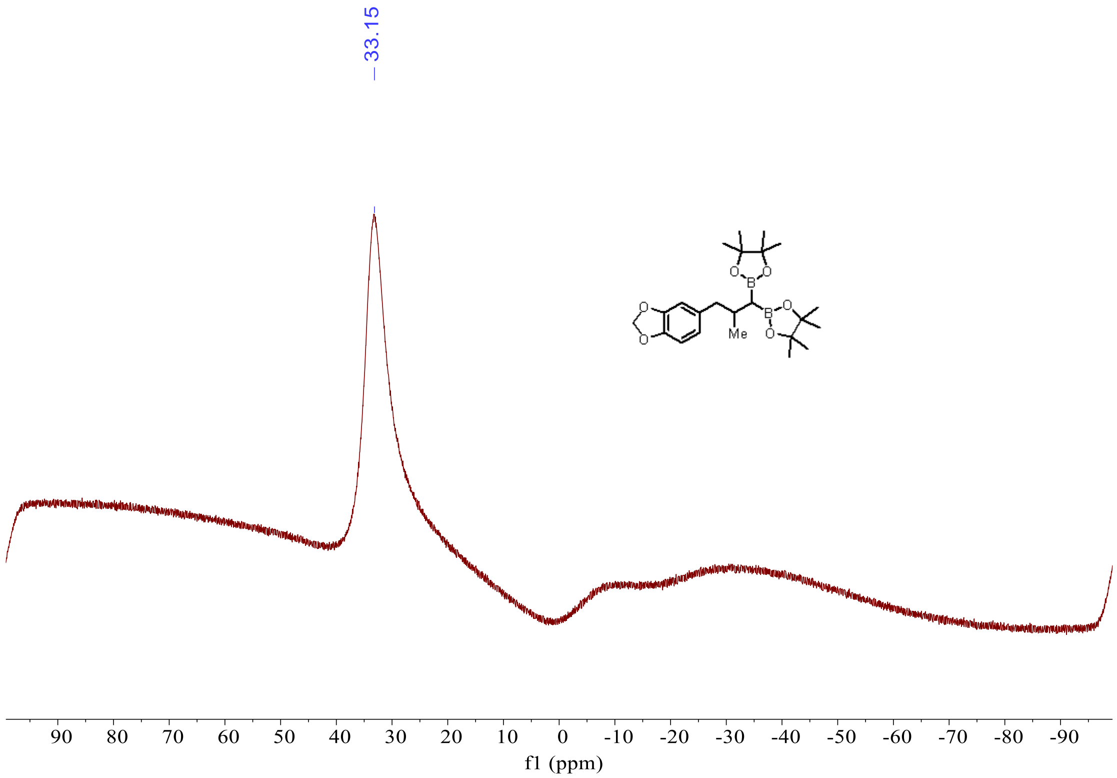

Supplementary Figure 132. ^{11}B NMR (128 MHz, $CDCl_3$) spectra for compound **24**

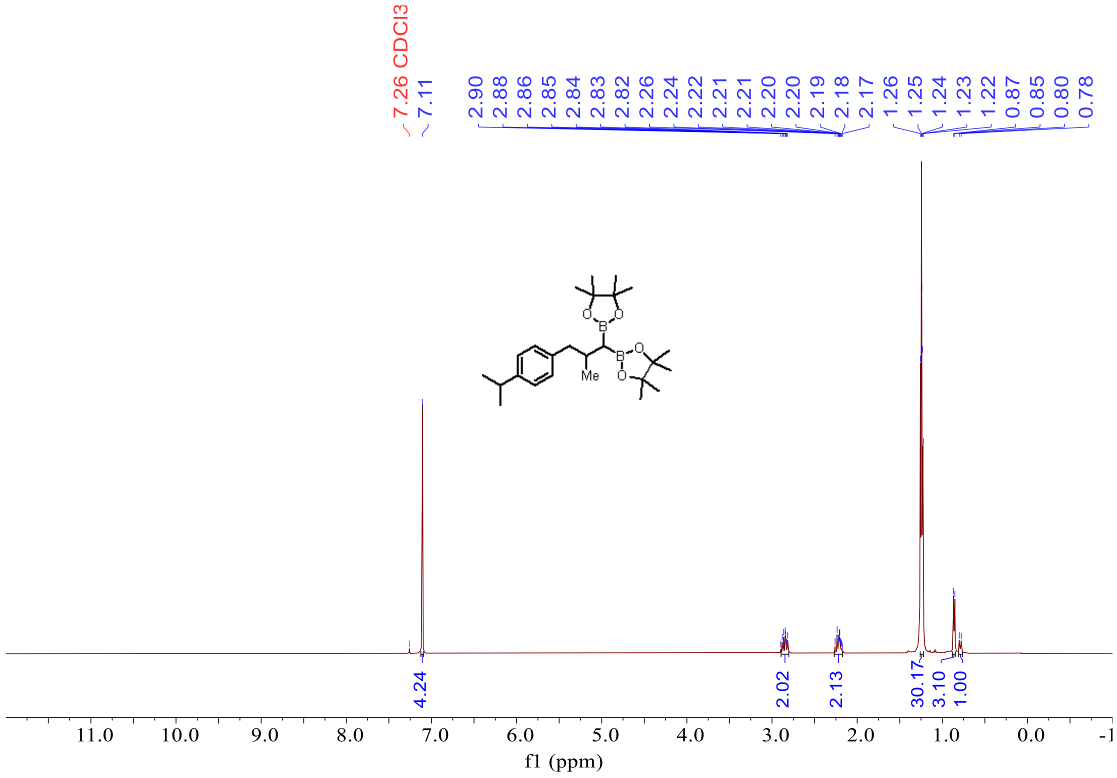

Supplementary Figure 133. ^{1}H NMR (400 MHz, $CDCl_3$) spectra for compound **25**

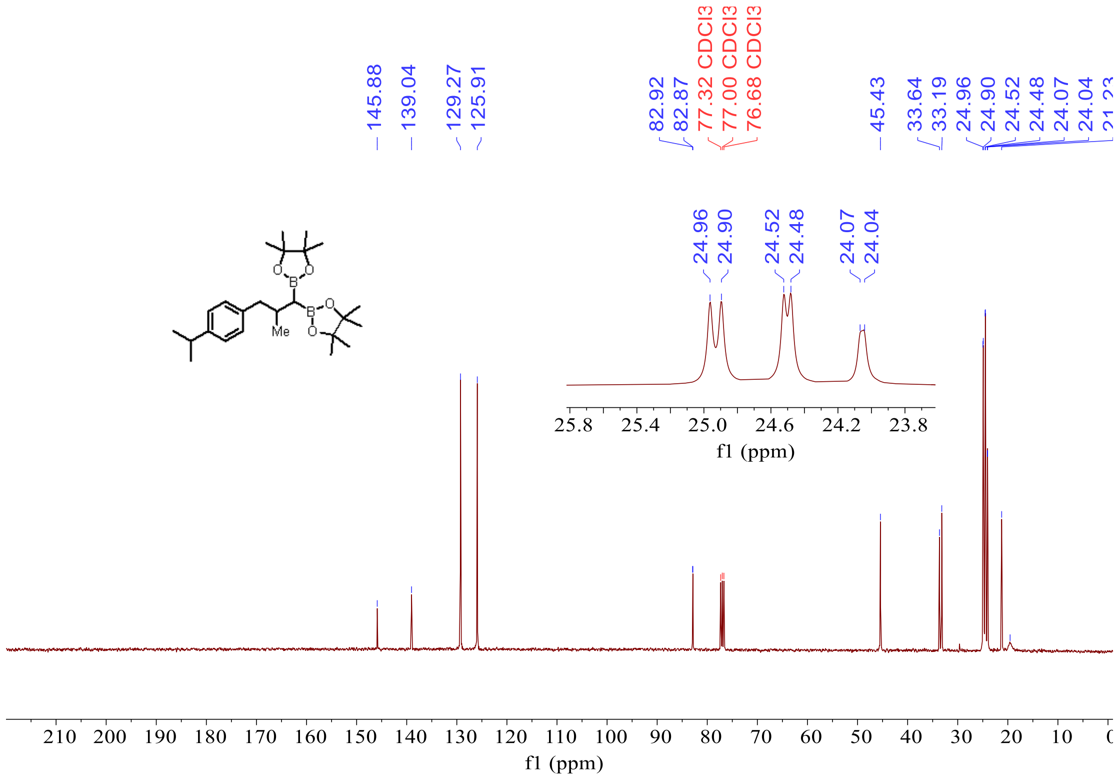

Supplementary Figure 134. ^{13}C NMR (101 MHz, $CDCl_3$) spectra for compound **25**

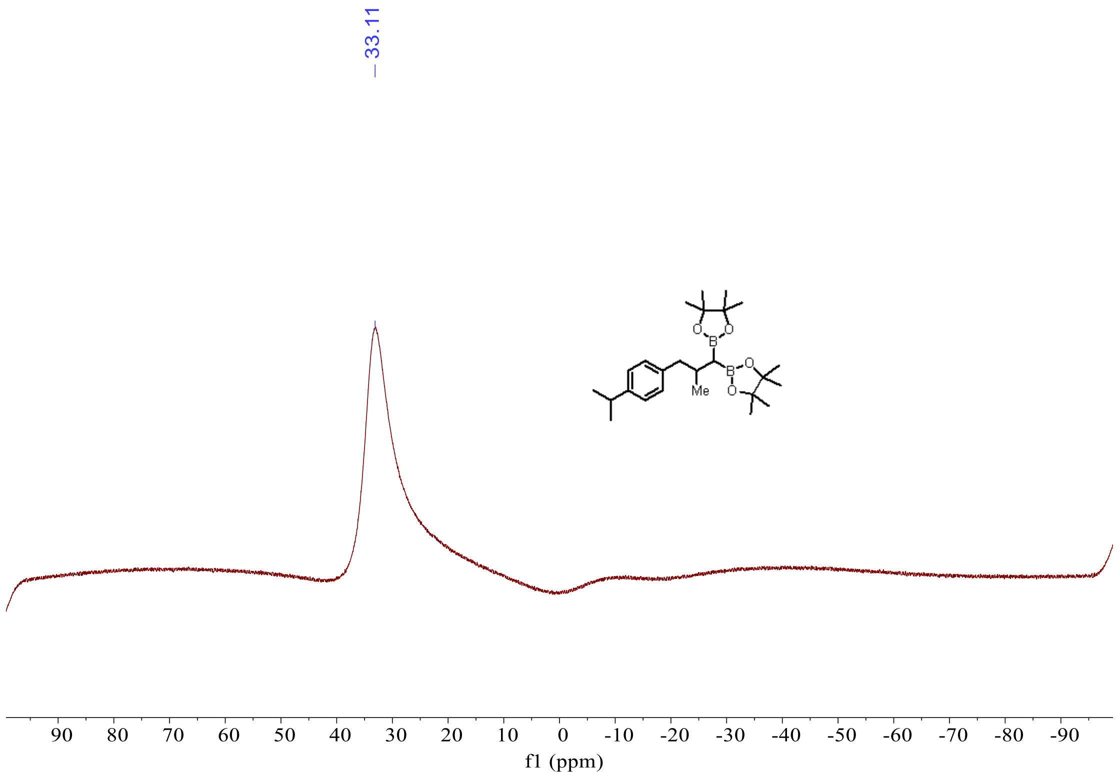

Supplementary Figure 135. ^{11}B NMR (128 MHz, $CDCl_3$) spectra for compound **25**

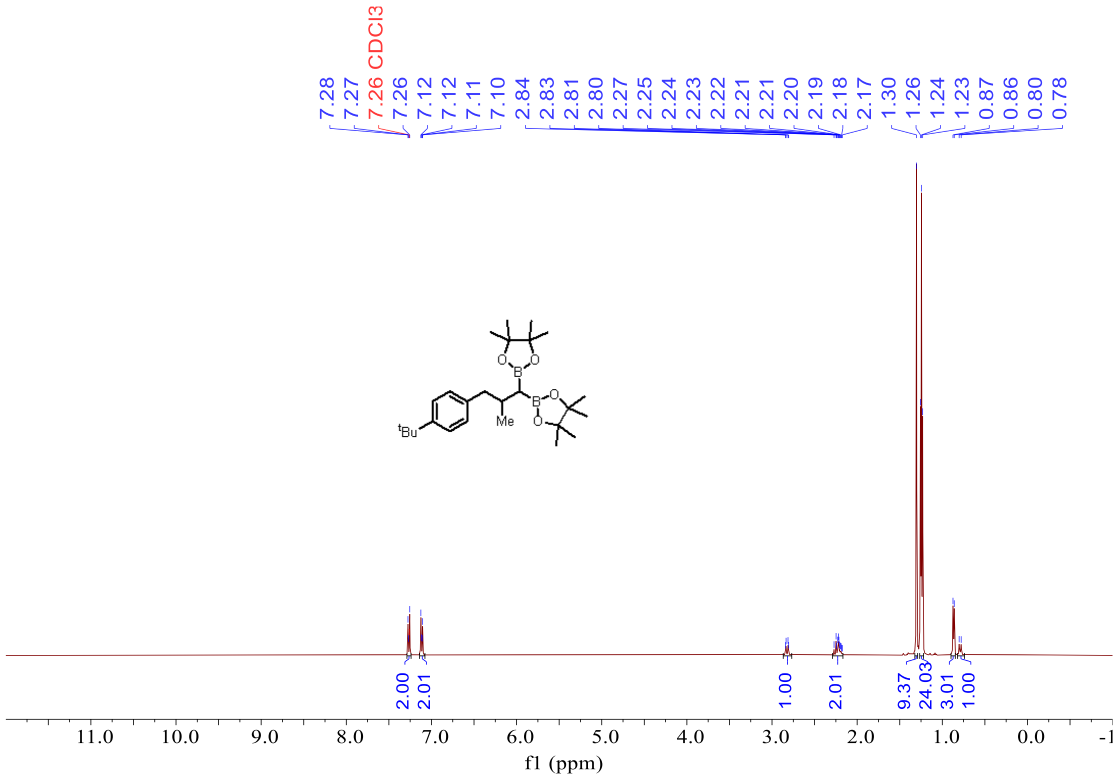

Supplementary Figure 136. ^{1}H NMR (400 MHz, $CDCl_3$) spectra for compound **26**

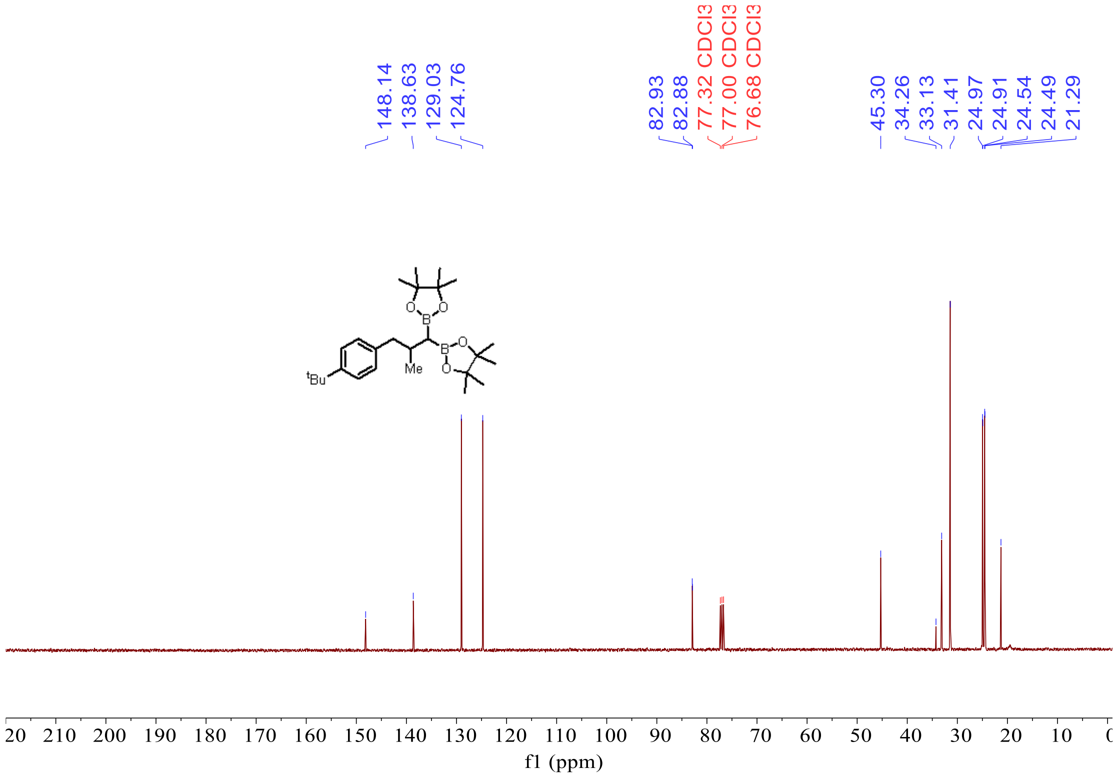

Supplementary Figure 137. ^{13}C NMR (101 MHz, $CDCl_3$) spectra for compound **26**

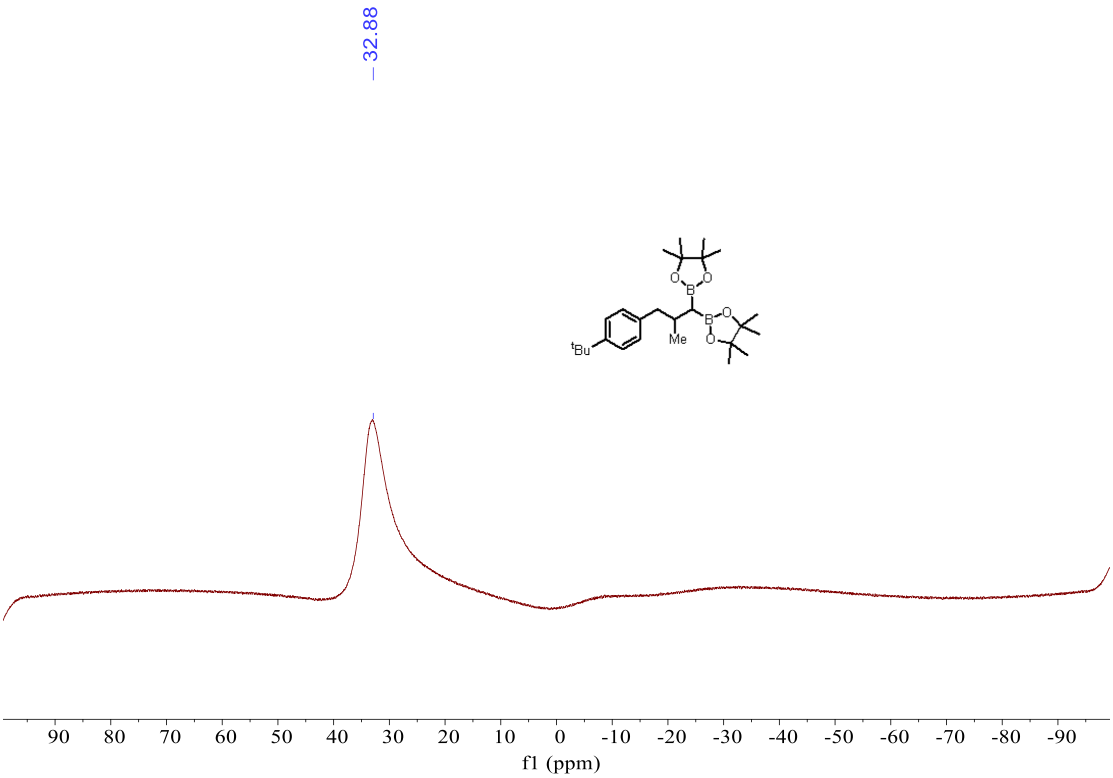

Supplementary Figure 138. ^{11}B NMR (128 MHz, $CDCl_3$) spectra for compound **26**

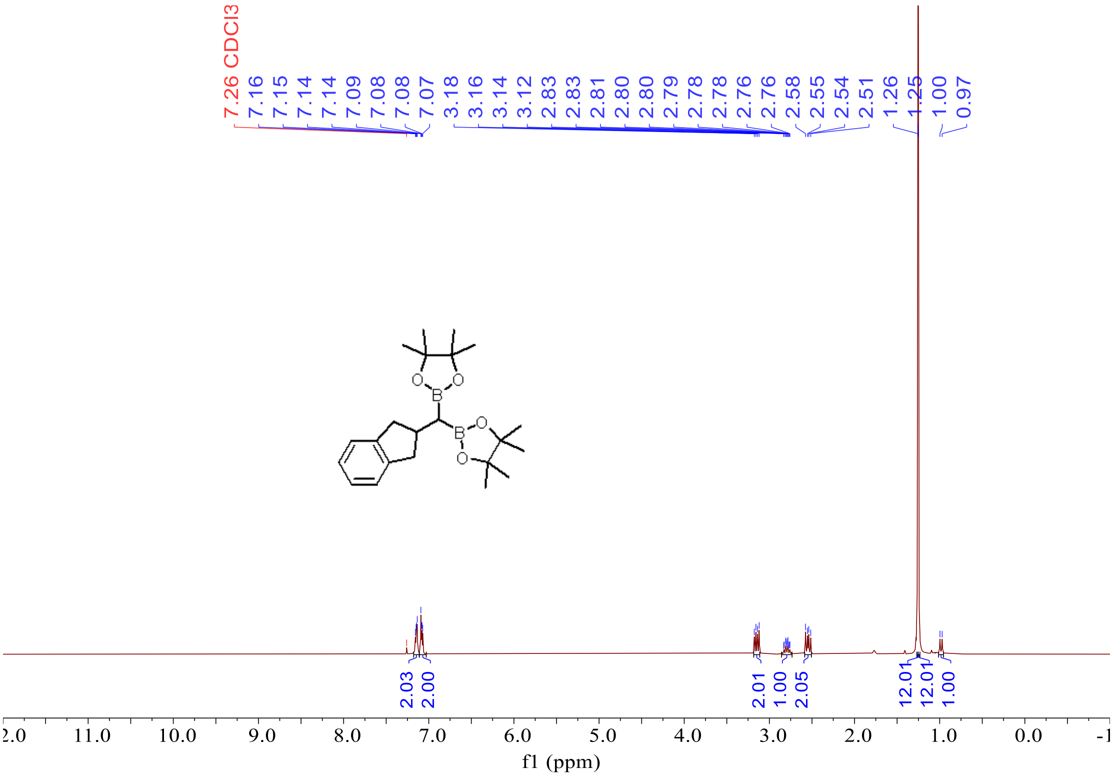

Supplementary Figure 139. ^{1}H NMR (400 MHz, $CDCl_3$) spectra for compound **27**

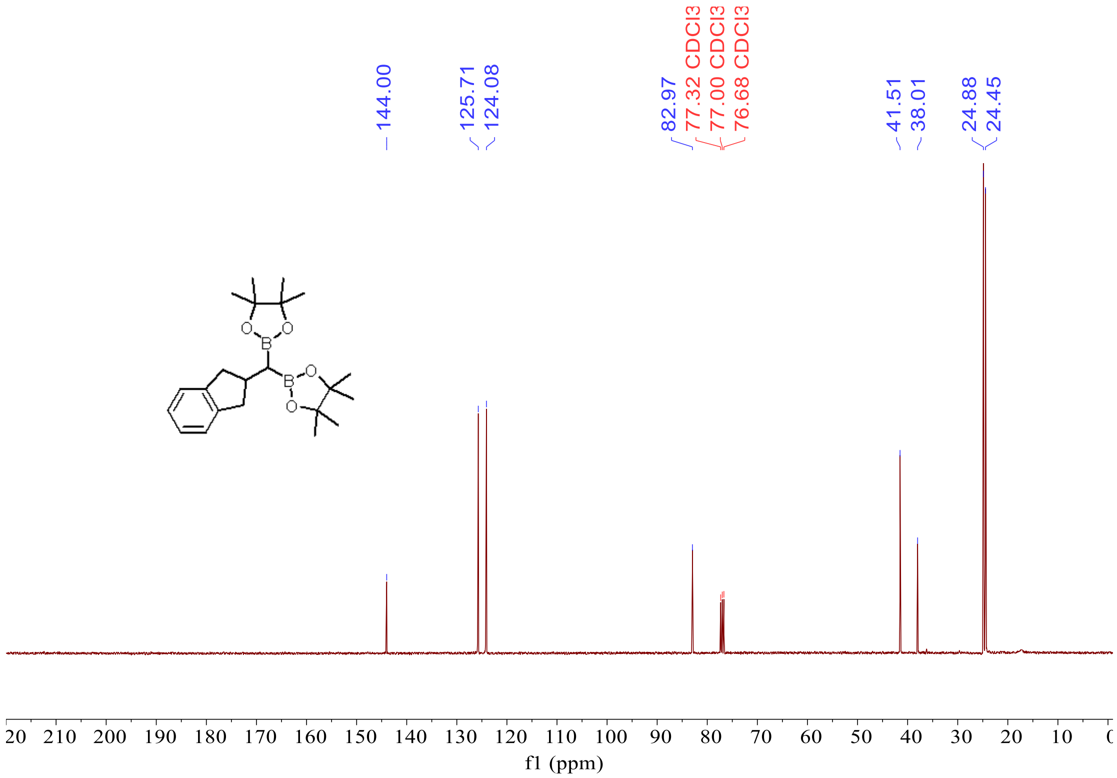

Supplementary Figure 140. ^{13}C NMR (101 MHz, $CDCl_3$) spectra for compound **27**

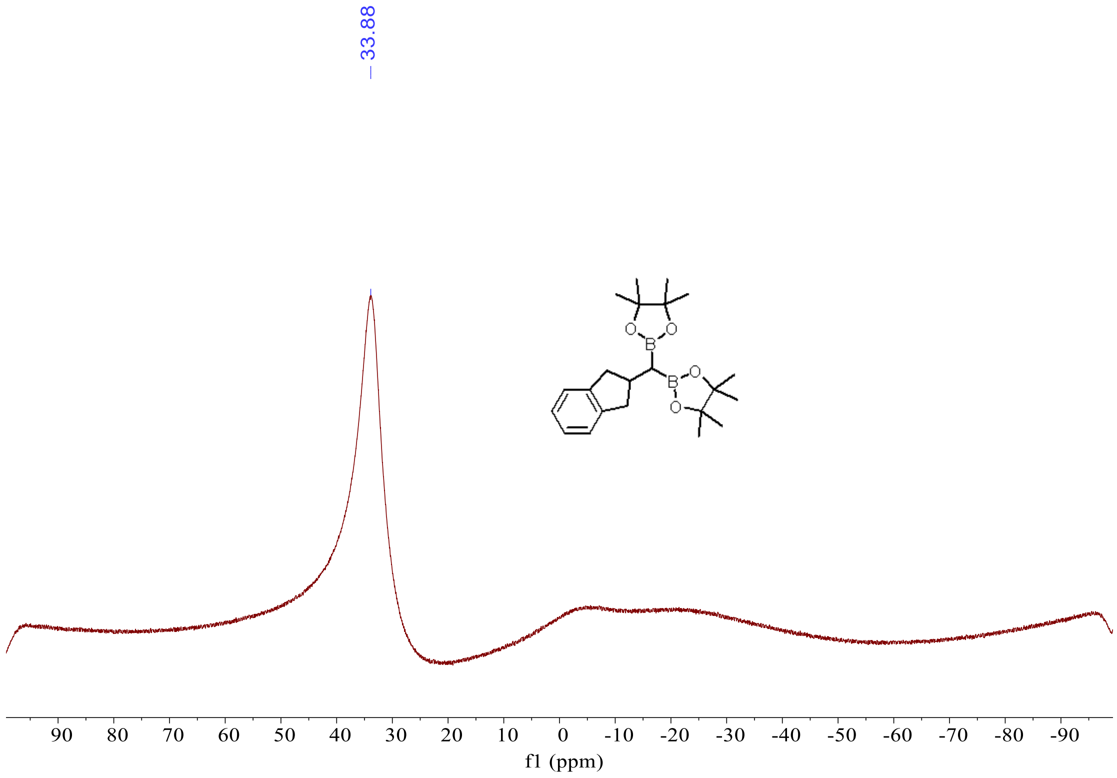

Supplementary Figure 141. ^{11}B NMR (128 MHz, $CDCl_3$) spectra for compound **27**

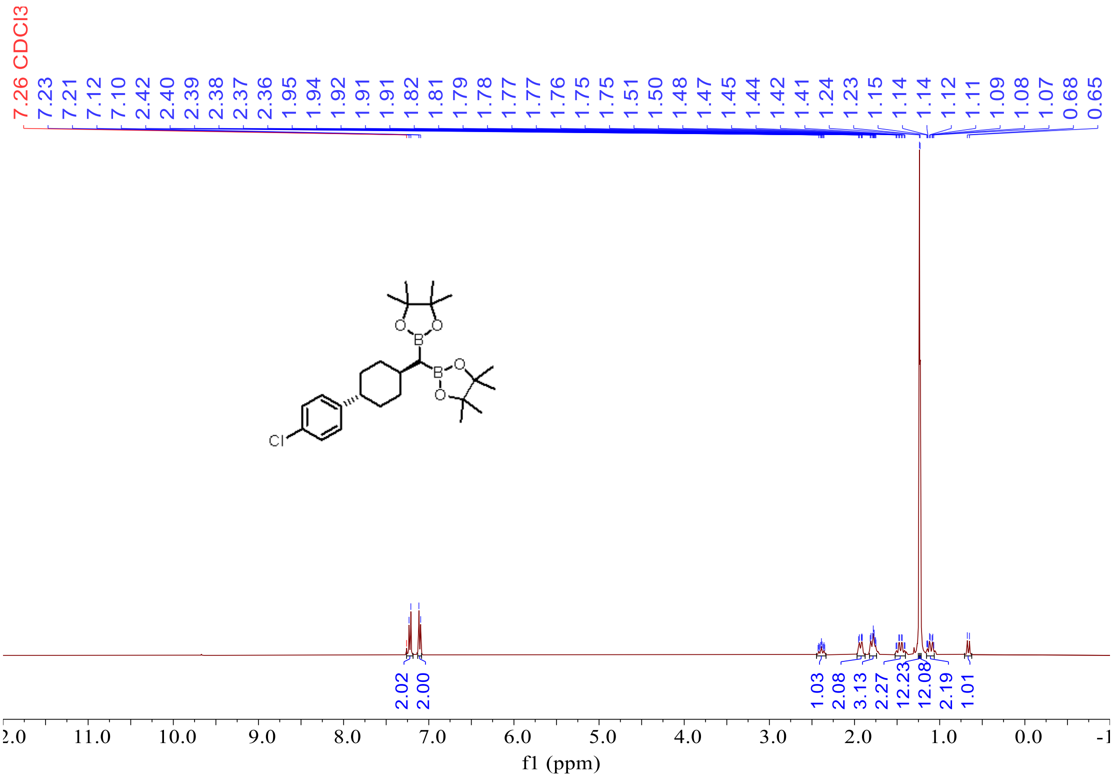

Supplementary Figure 142. ^{1}H NMR (400 MHz, $CDCl_3$) spectra for compound **28**

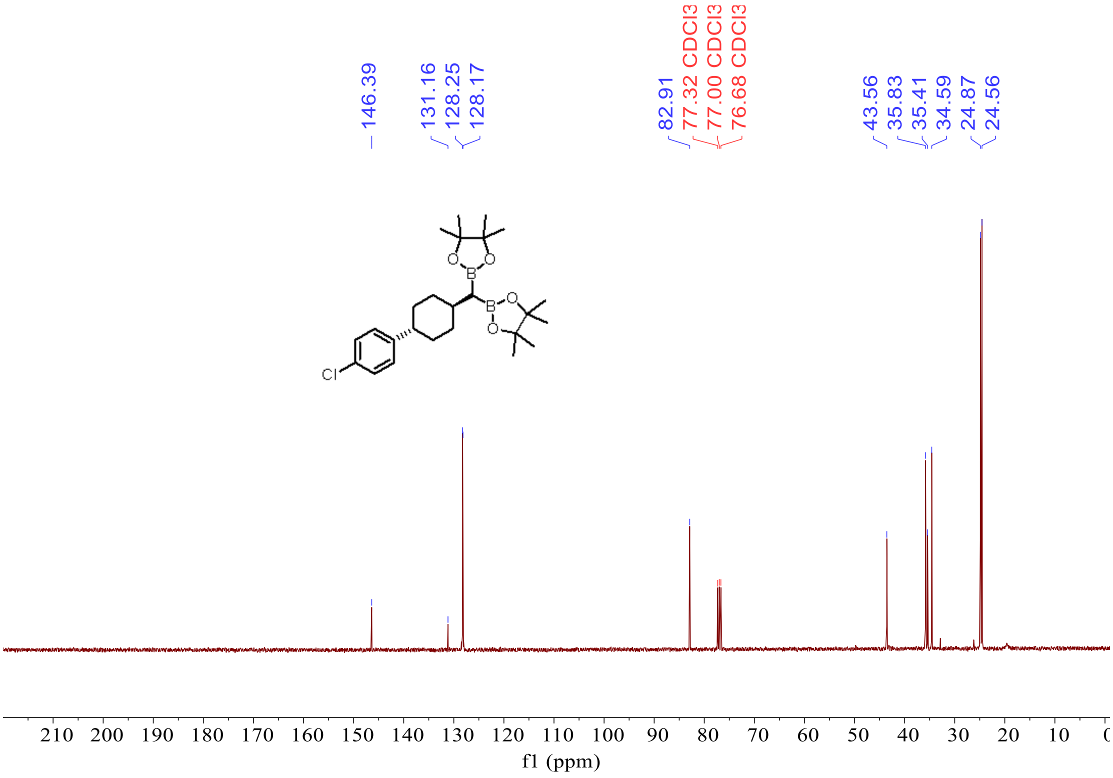

Supplementary Figure 143. ^{13}C NMR (101 MHz, $CDCl_3$) spectra for compound **28**

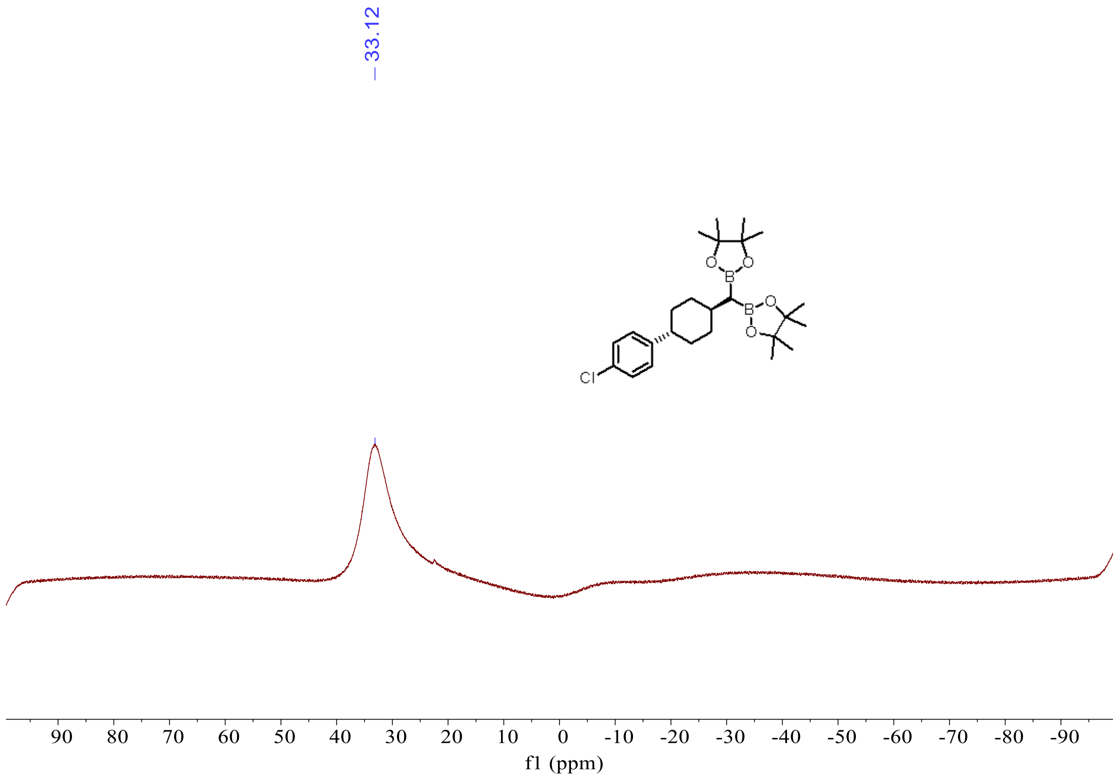

Supplementary Figure 144. ^{11}B NMR (128 MHz, $CDCl_3$) spectra for compound **28**

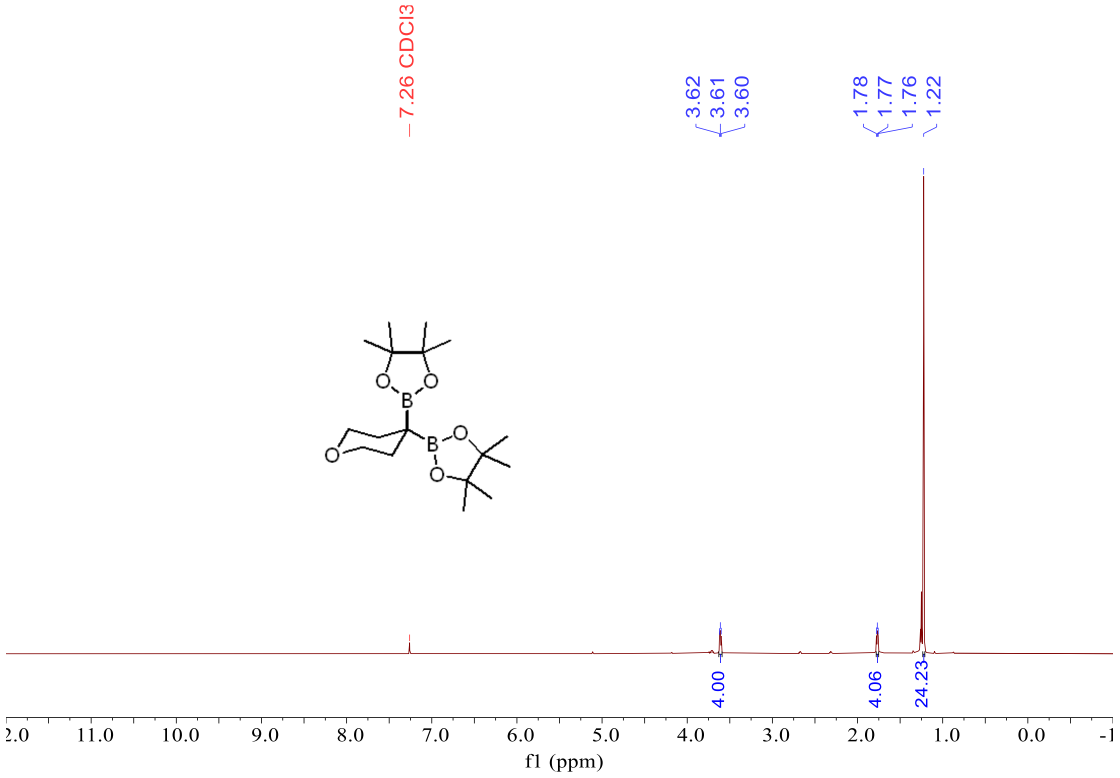

Supplementary Figure 145. ^{1}H NMR (500 MHz, $CDCl_3$) spectra for compound **29**

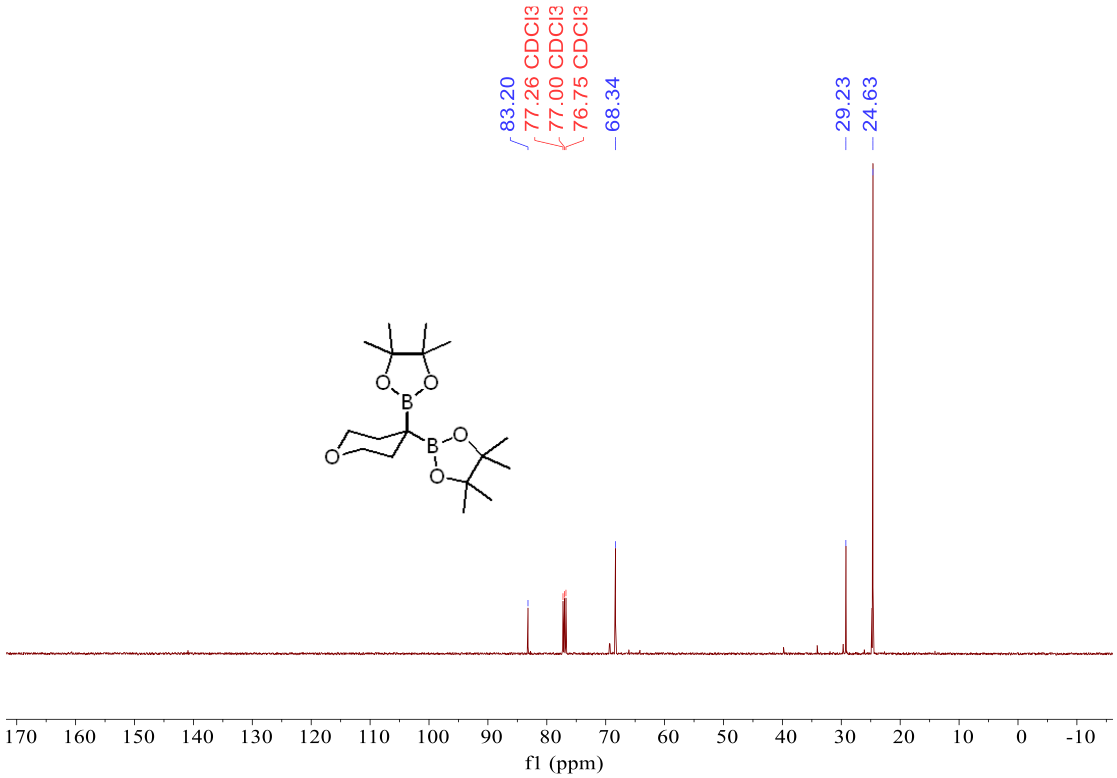

Supplementary Figure 146. ^{13}C NMR (126 MHz, $CDCl_3$) spectra for compound **29**

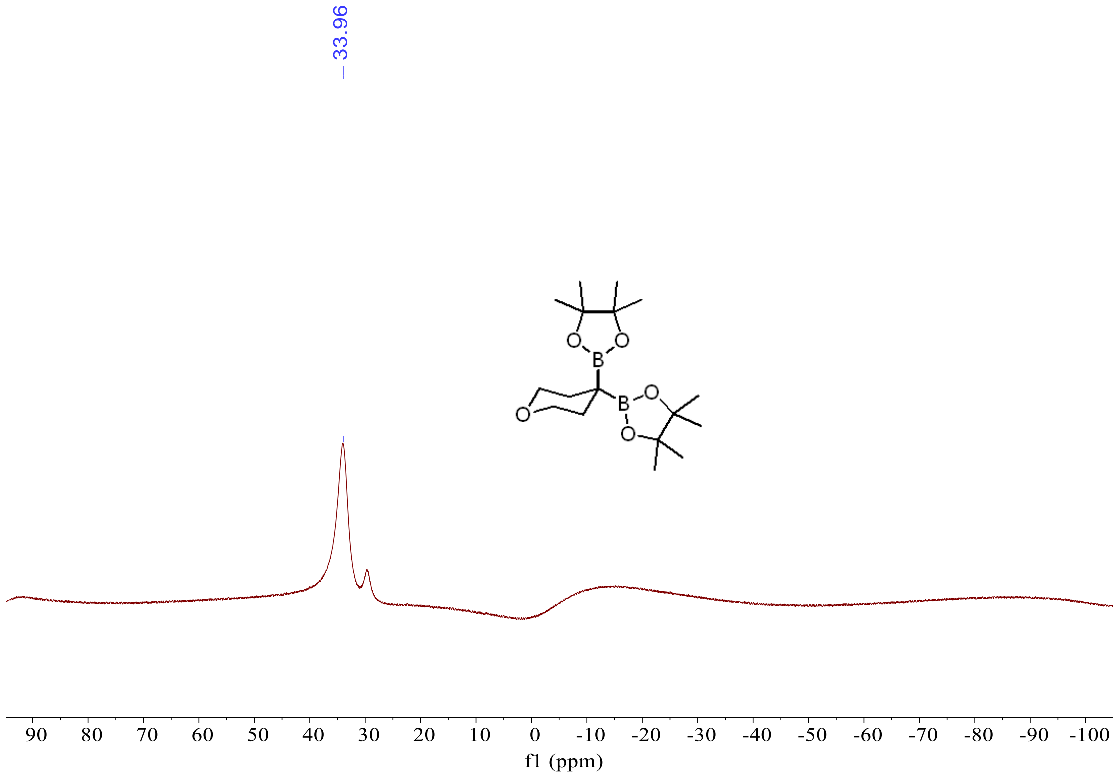

Supplementary Figure 147. ^{11}B NMR (160 MHz, $CDCl_3$) spectra for compound **29**

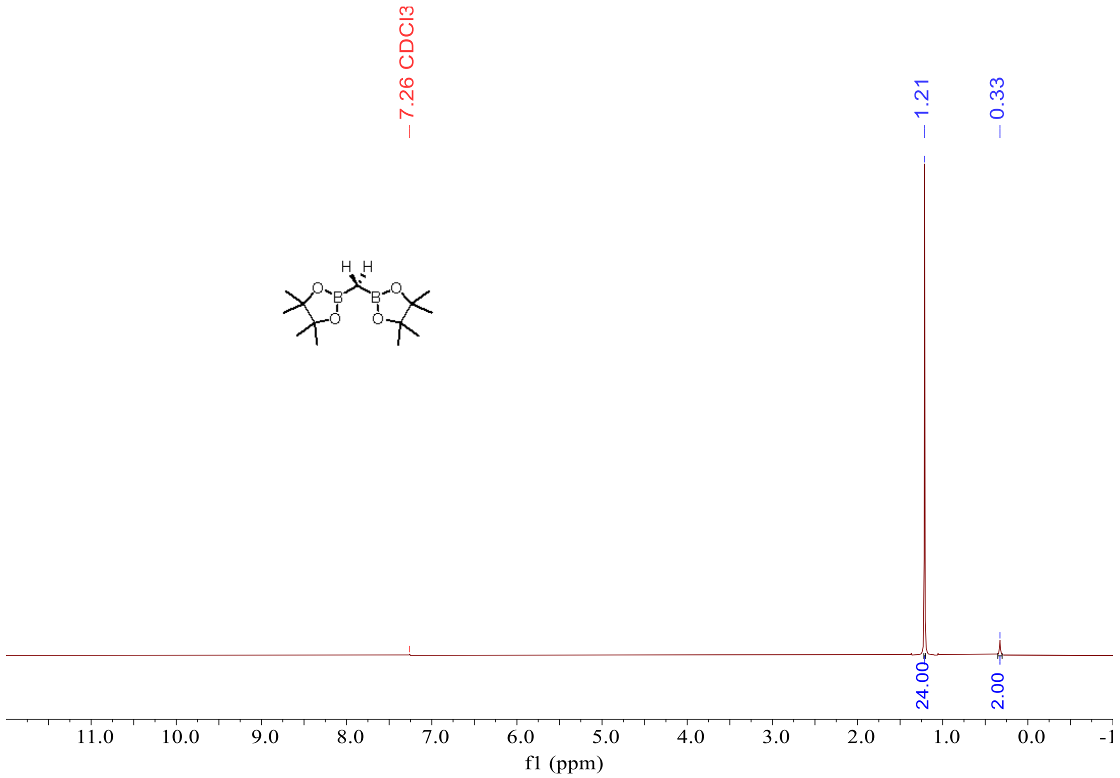

Supplementary Figure 148. ^{1}H NMR (400 MHz, $CDCl_3$) spectra for compound **30**

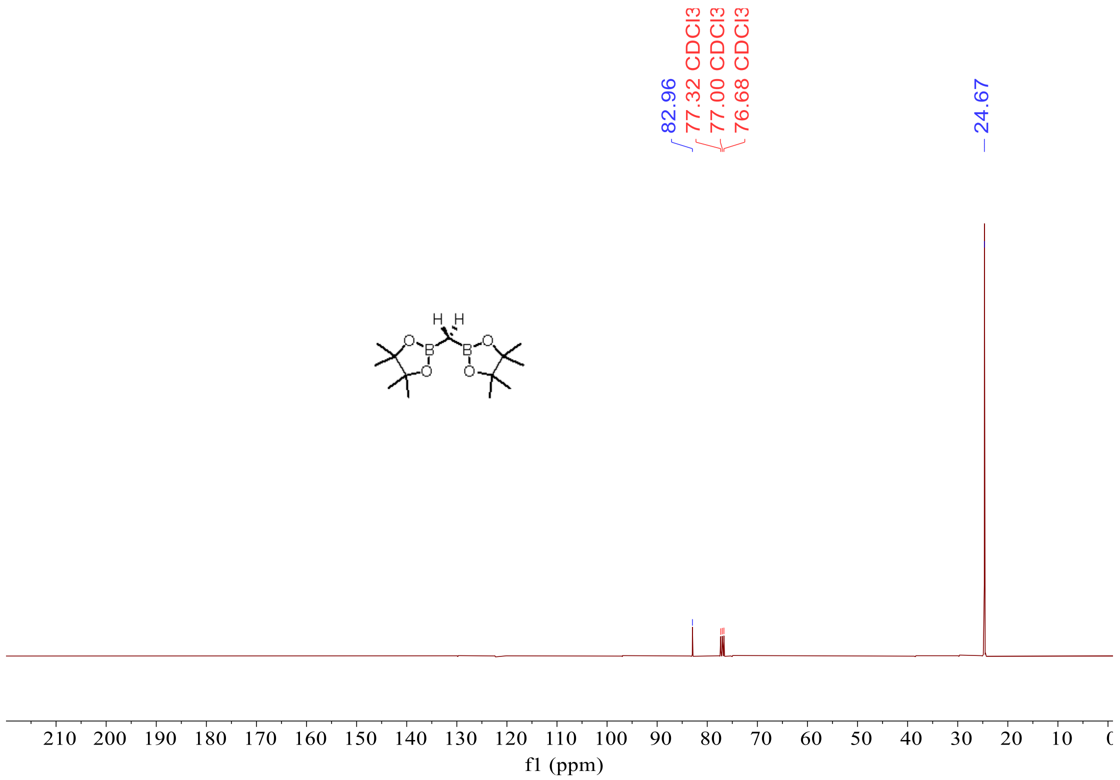

Supplementary Figure 149. ^{13}C NMR (101 MHz, $CDCl_3$) spectra for compound **30**

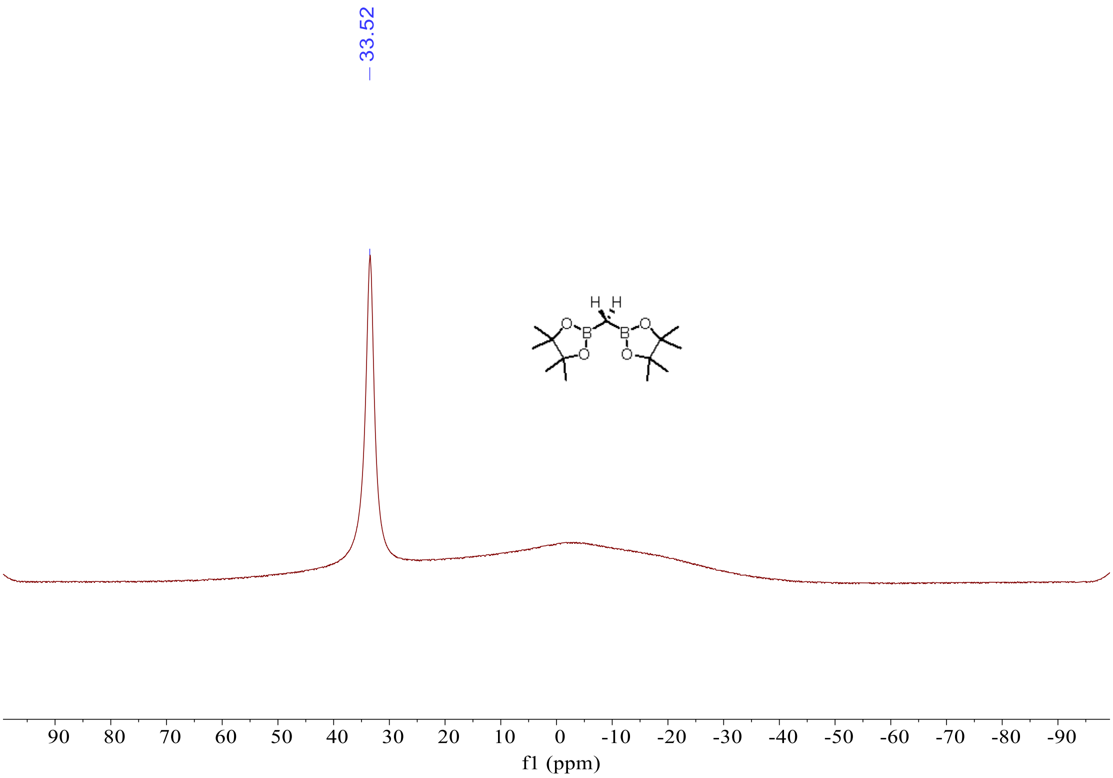

Supplementary Figure 150. ^{11}B NMR (128 MHz, $CDCl_3$) spectra for compound **30**

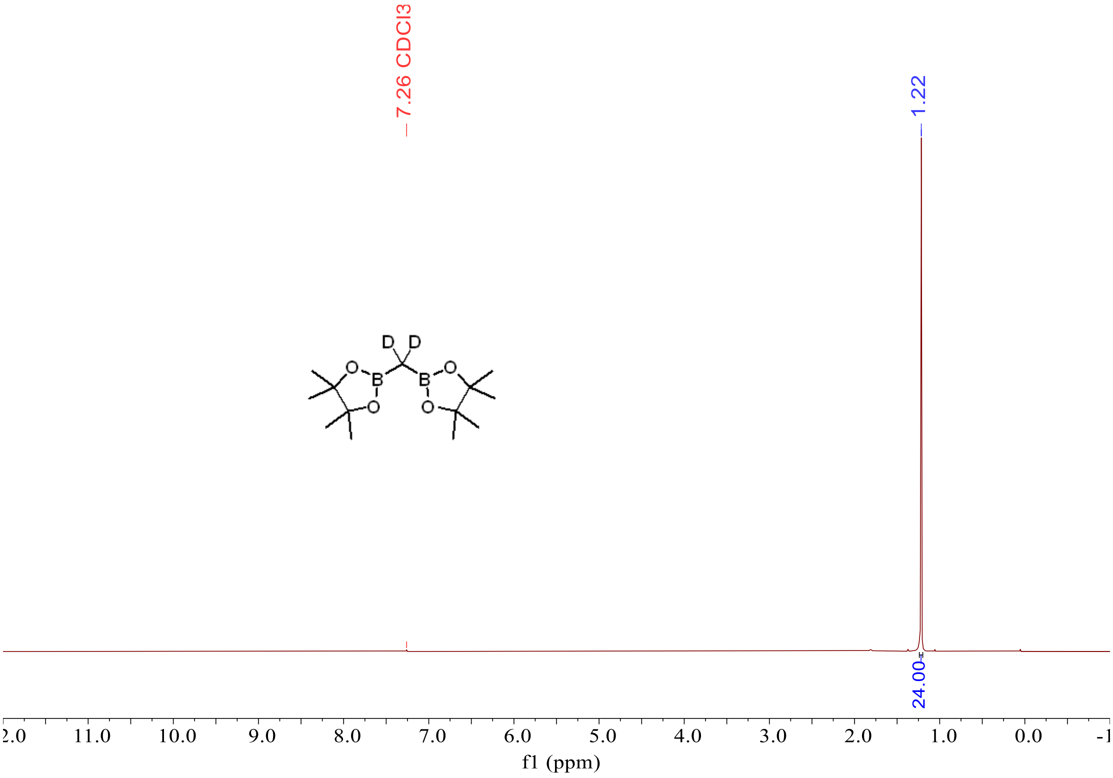

Supplementary Figure 151. ^{1}H NMR (400 MHz, $CDCl_3$) spectra for compound **31**

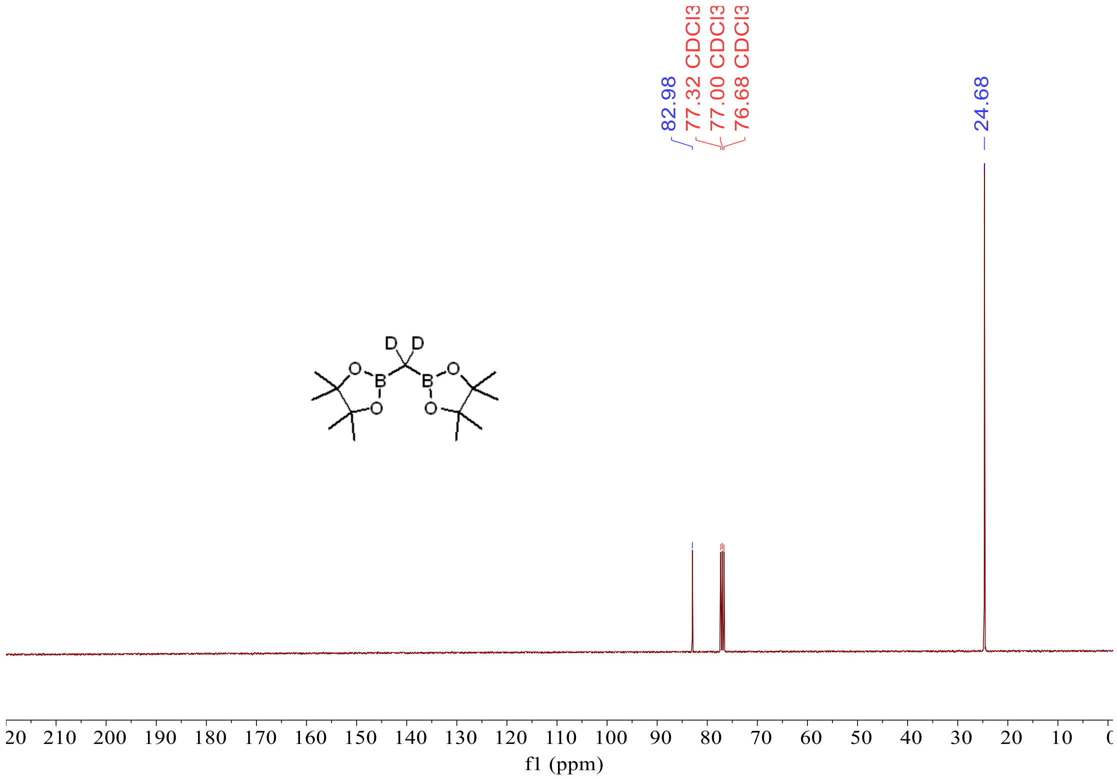

Supplementary Figure 152. ^{13}C NMR (101 MHz, $CDCl_3$) spectra for compound **31**

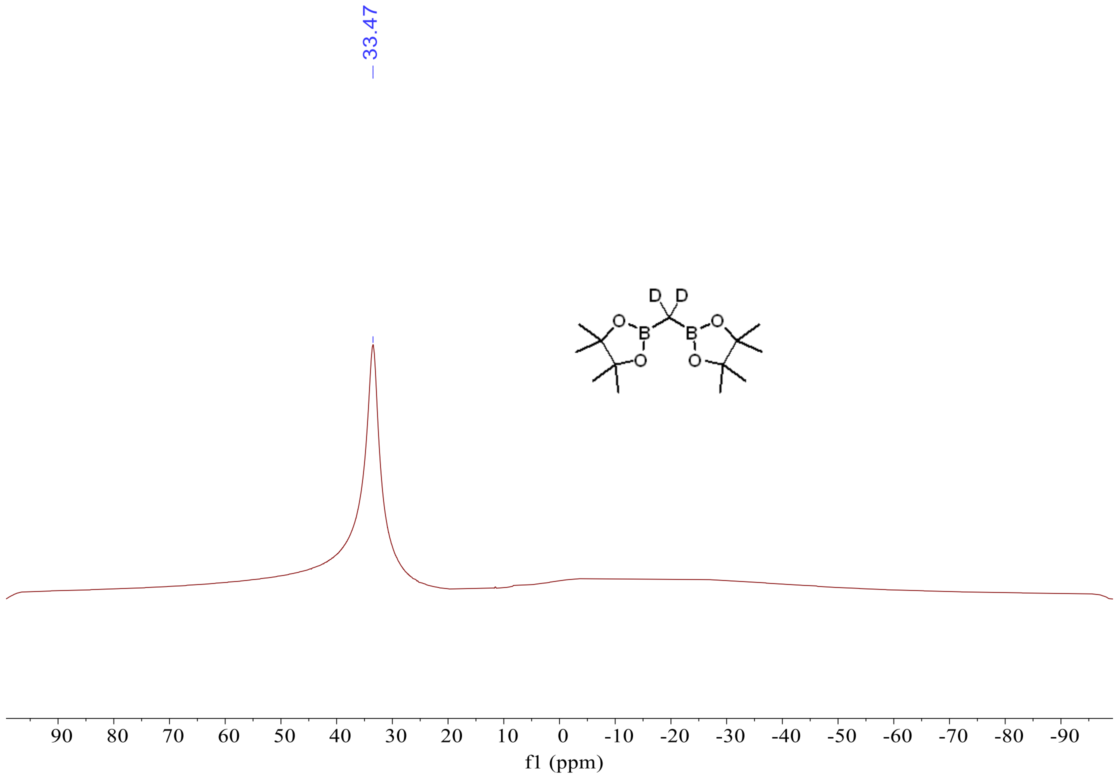

Supplementary Figure 153. ^{11}B NMR (128 MHz, $CDCl_3$) spectra for compound **31**

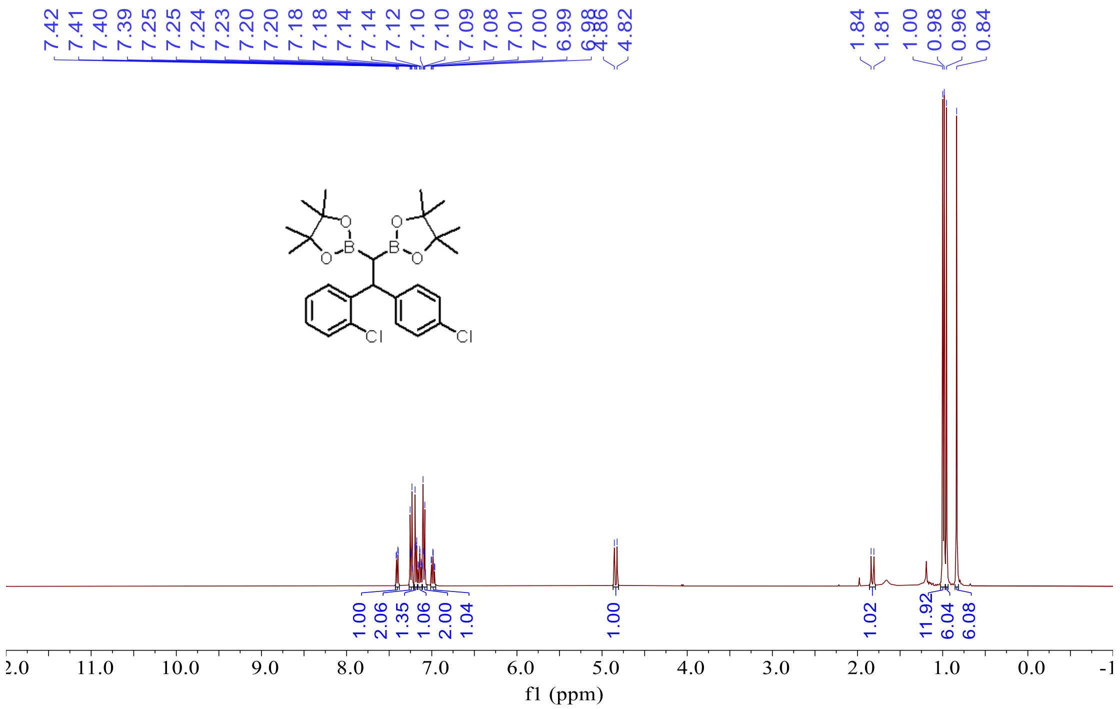

Supplementary Figure 154. ^{1}H NMR (400 MHz, $CDCl_3$) spectra for compound **32**

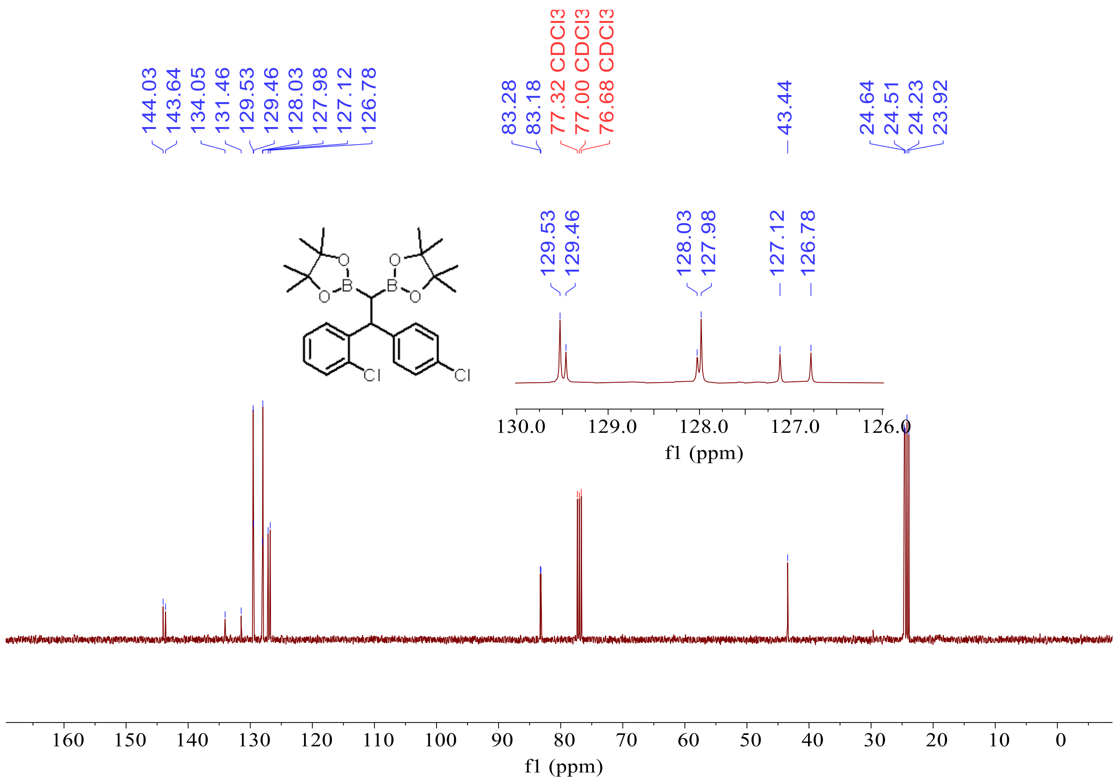

Supplementary Figure 155. ^{13}C NMR (101 MHz, $CDCl_3$) spectra for compound **32**

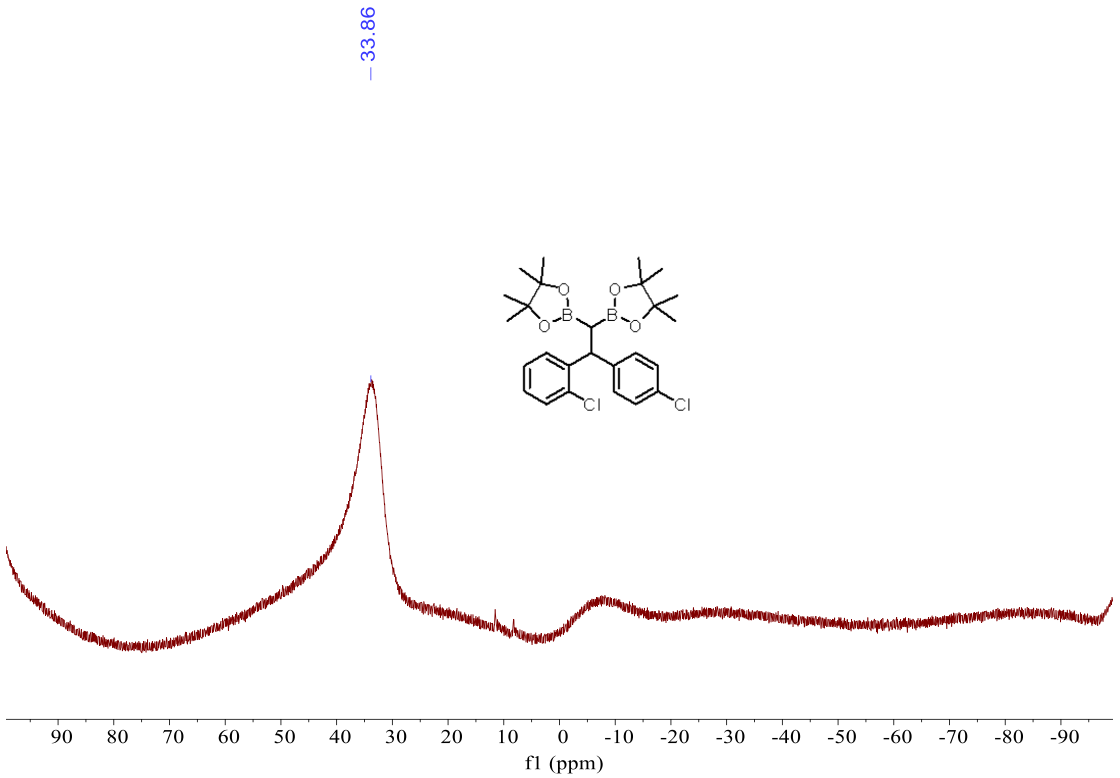

Supplementary Figure 156. ^{11}B NMR (128 MHz, $CDCl_3$) spectra for compound **32**

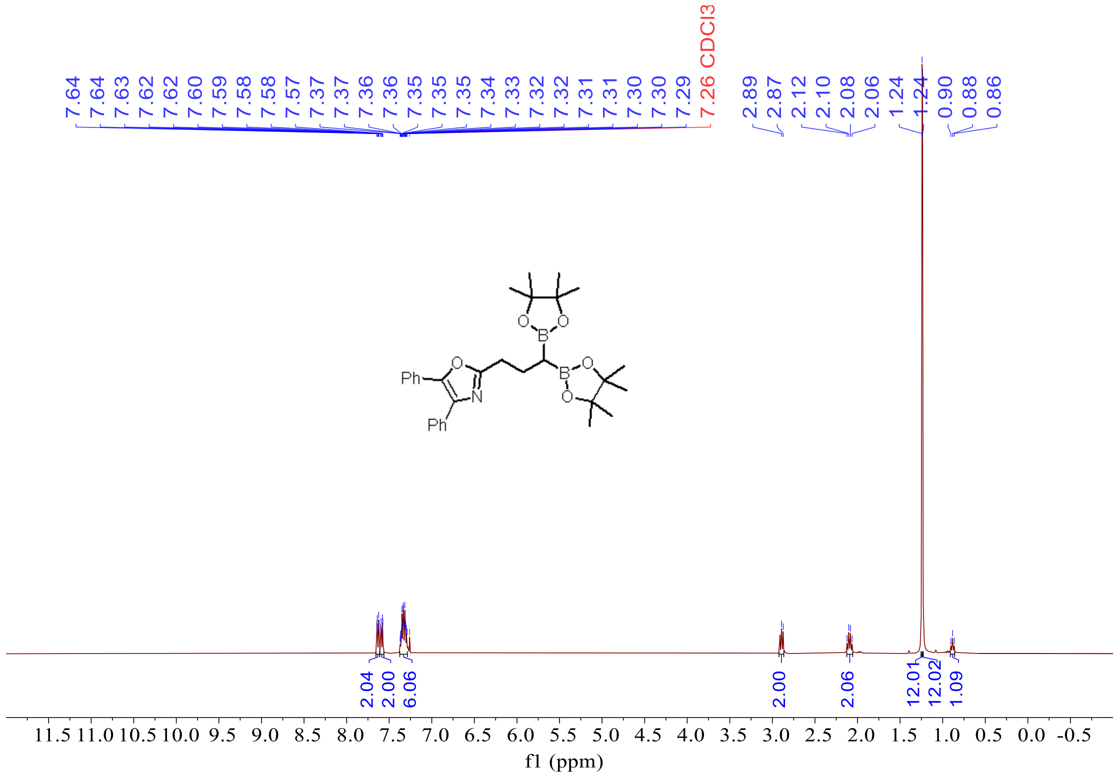

Supplementary Figure 157. ^{1}H NMR (400 MHz, $CDCl_3$) spectra for compound **33**

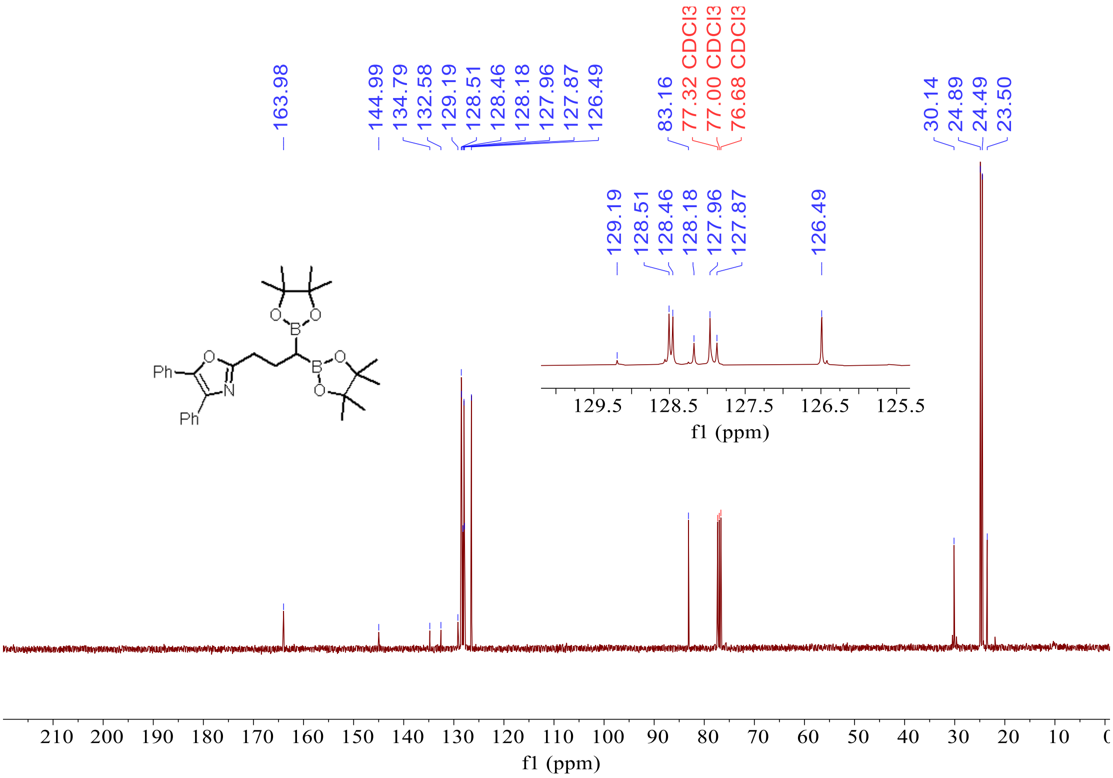

Supplementary Figure 158. ^{13}C NMR (101 MHz, $CDCl_3$) spectra for compound **33**

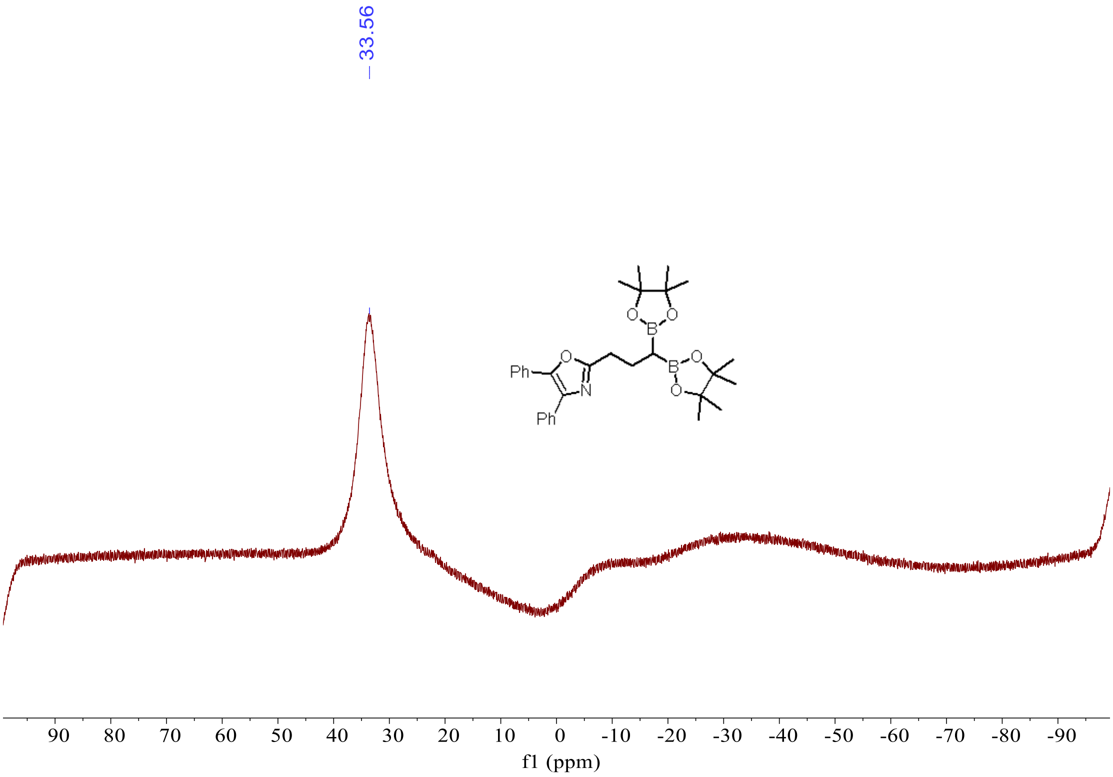

Supplementary Figure 159. ^{11}B NMR (128 MHz, $CDCl_3$) spectra for compound **33**

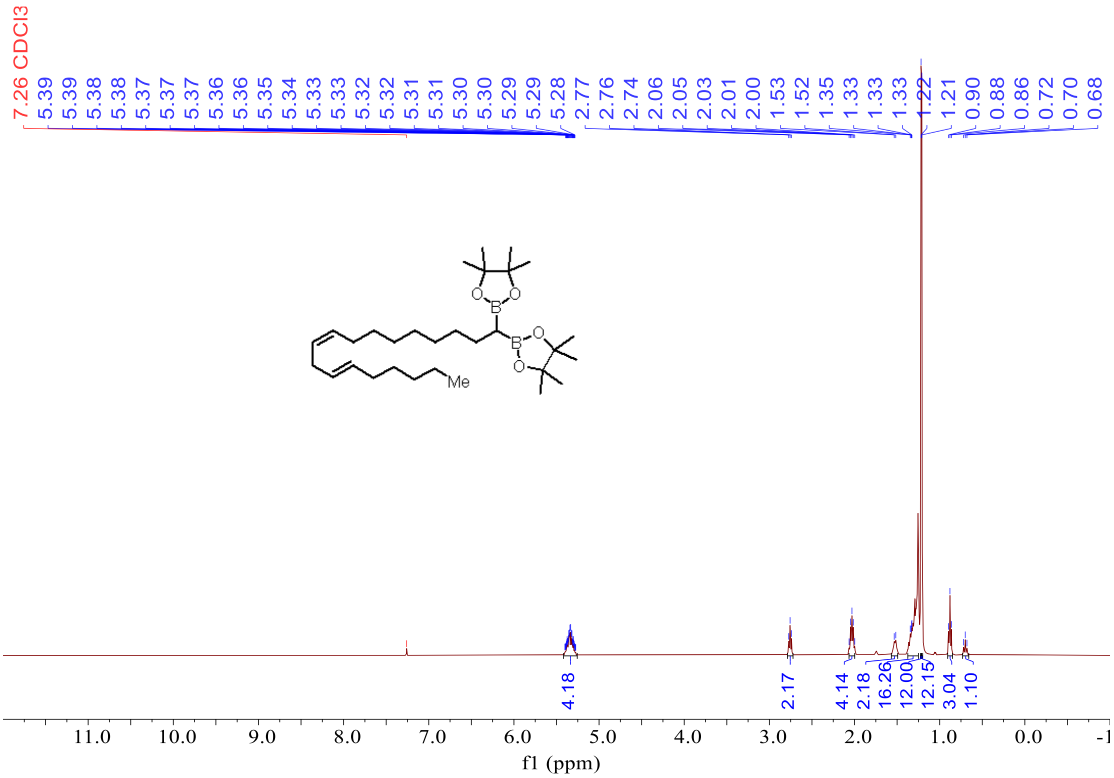

Supplementary Figure 160. ^{1}H NMR (400 MHz, $CDCl_3$) spectra for compound **34**

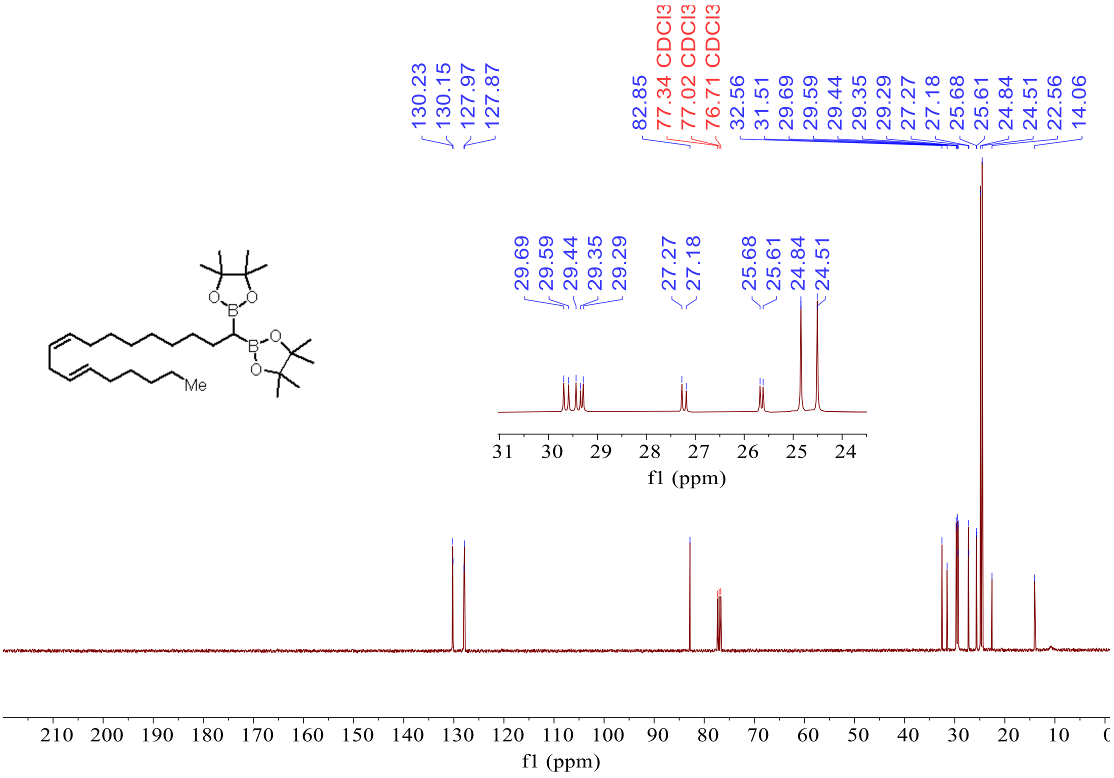

Supplementary Figure 161. ^{13}C NMR (101 MHz, $CDCl_3$) spectra for compound **34**

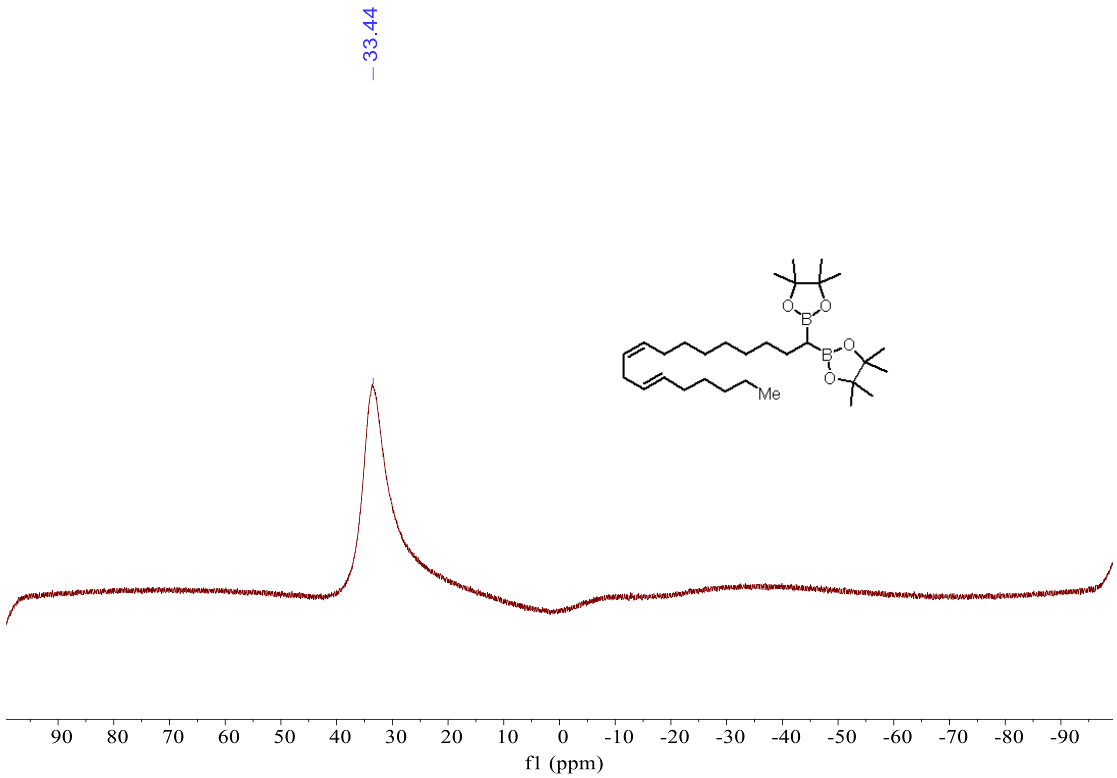

Supplementary Figure 162. ^{11}B NMR (128 MHz, $CDCl_3$) spectra for compound **34**

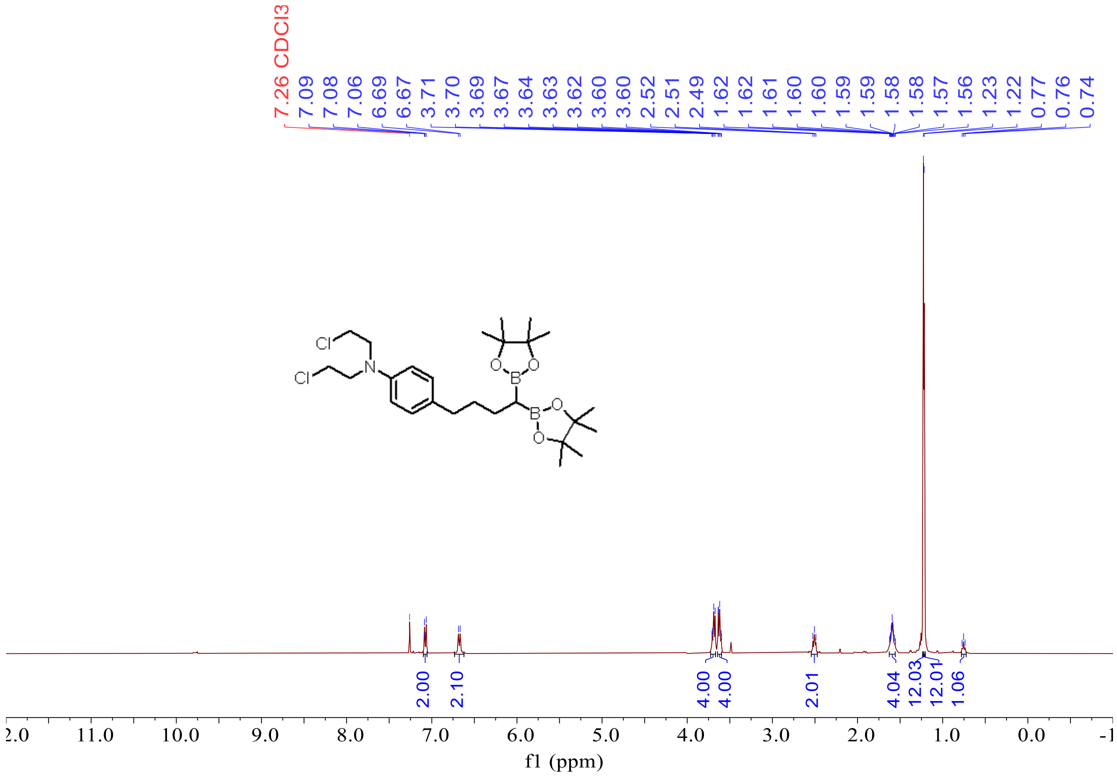

Supplementary Figure 163. ^{1}H NMR (400 MHz, $CDCl_3$) spectra for compound **35**

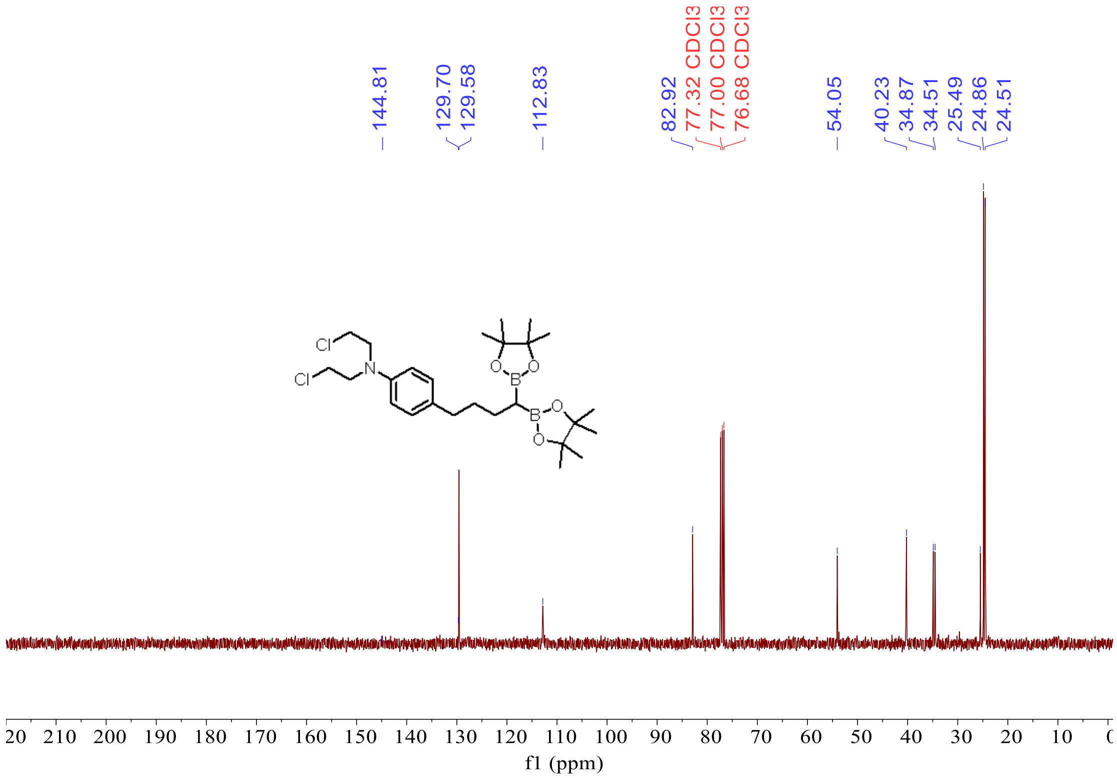

Supplementary Figure 164. ^{13}C NMR (101 MHz, $CDCl_3$) spectra for compound **35**

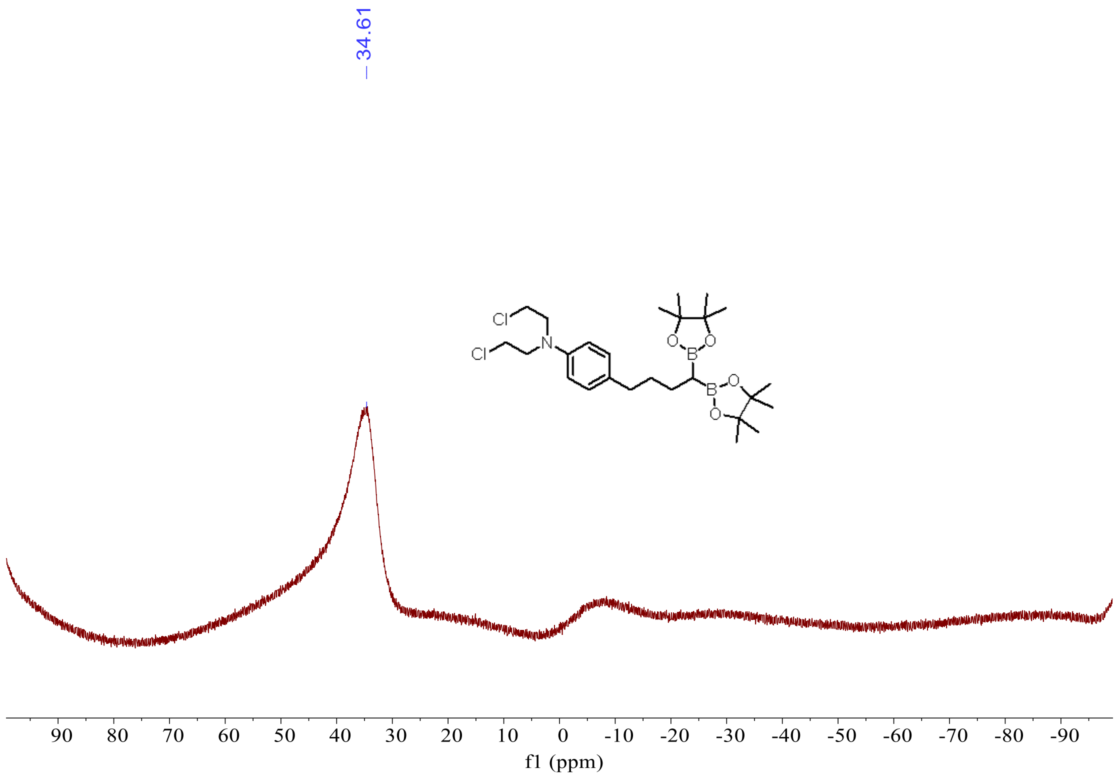

Supplementary Figure 165. ^{11}B NMR (128 MHz, $CDCl_3$) spectra for compound **35**

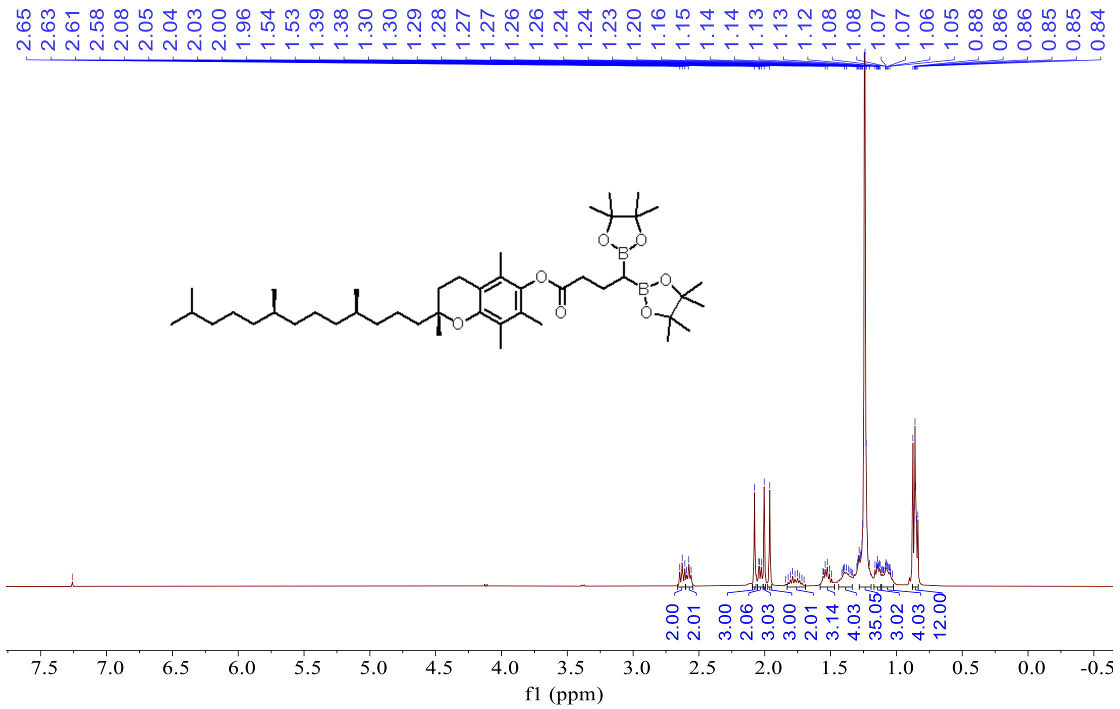

Supplementary Figure 166. ^{1}H NMR (400 MHz, $CDCl_3$) spectra for compound **36**

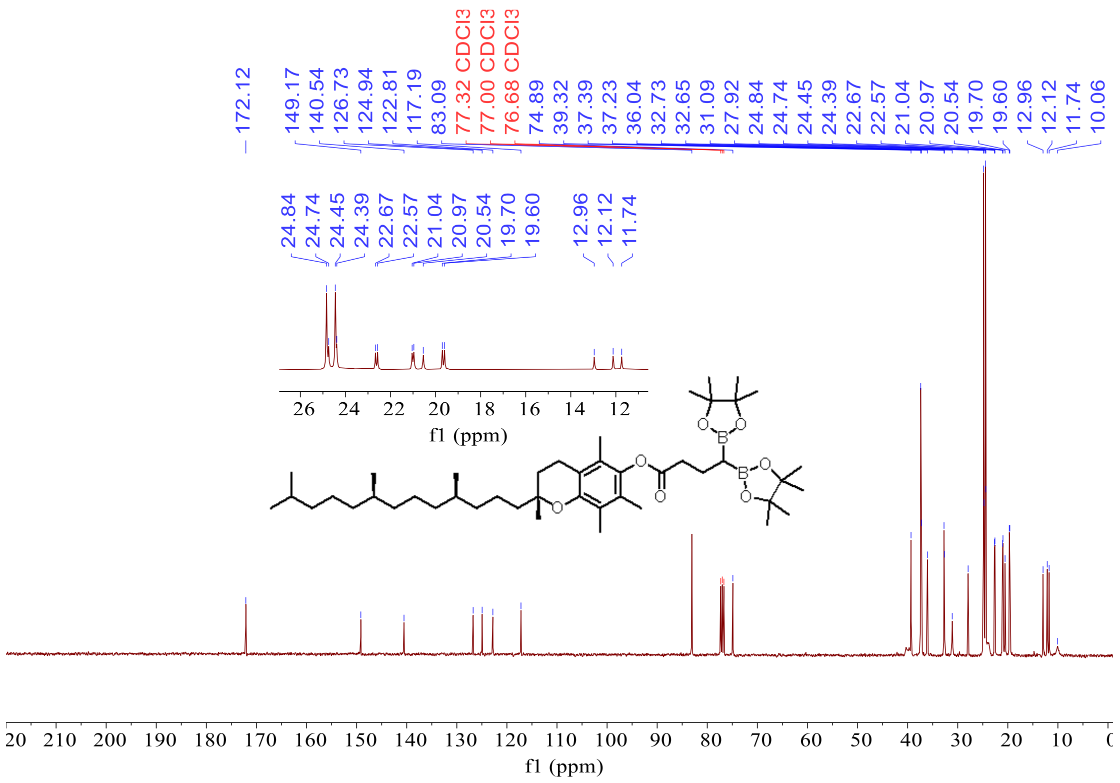

Supplementary Figure 167. ^{13}C NMR (101 MHz, $CDCl_3$) spectra for compound **36**

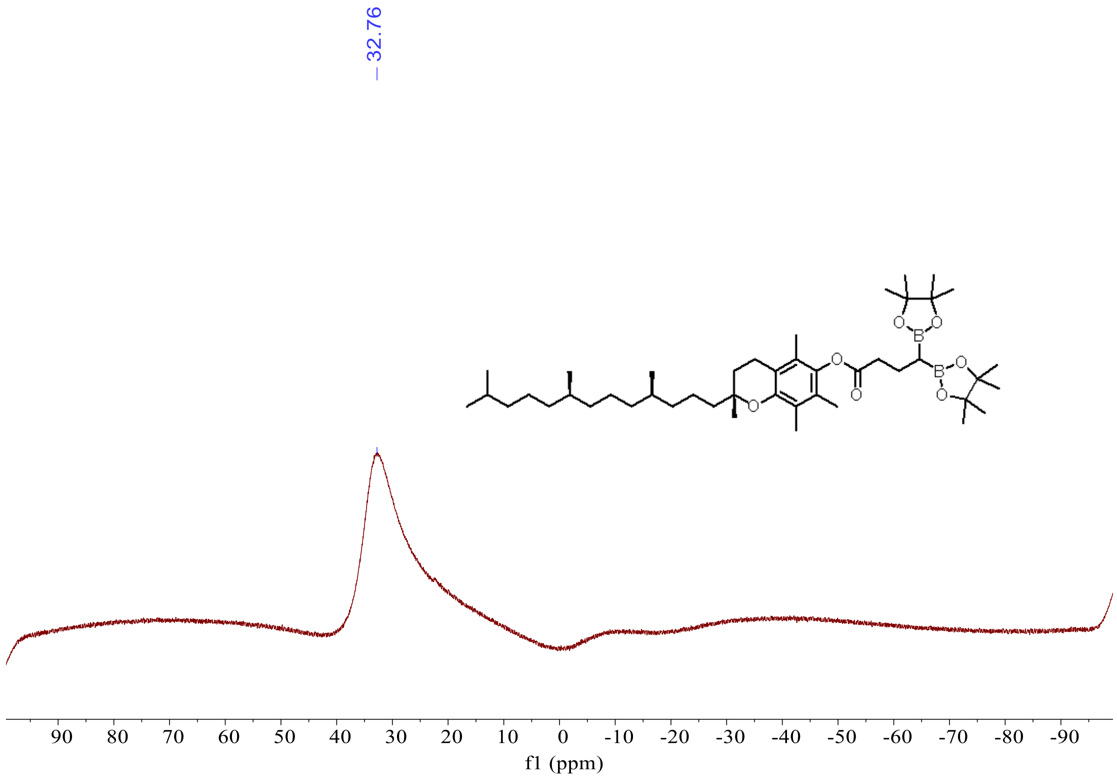

Supplementary Figure 168. ^{11}B NMR (128 MHz, $CDCl_3$) spectra for compound **36**

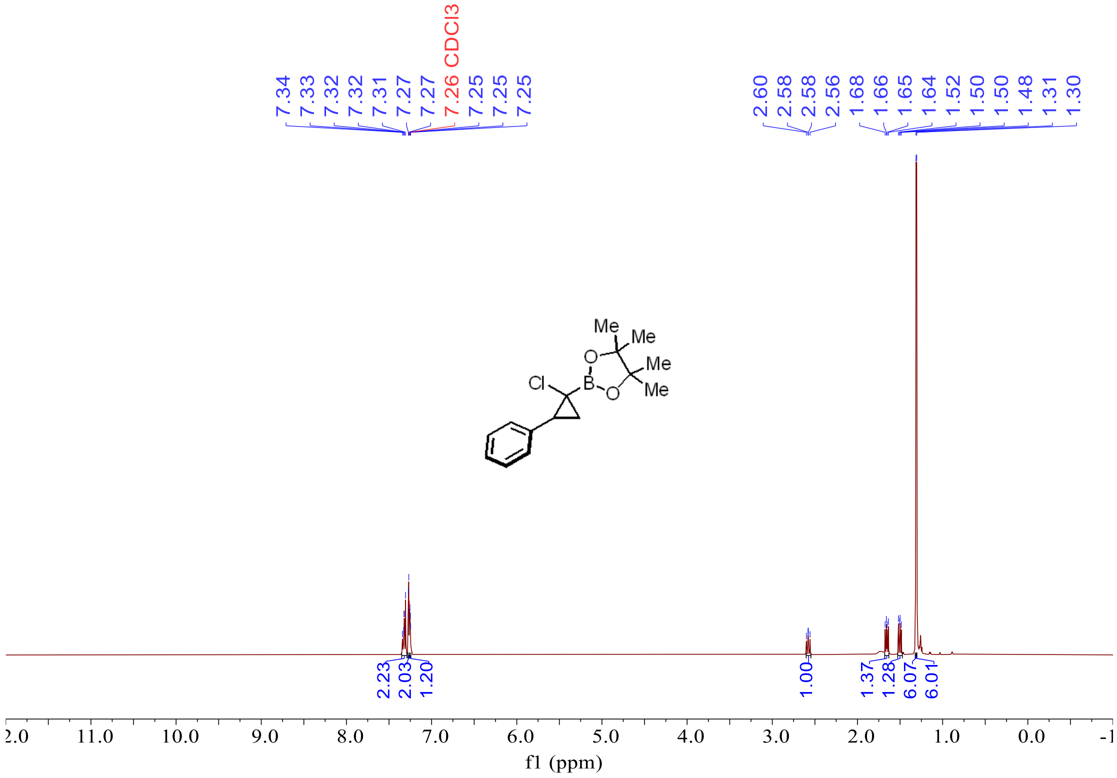

Supplementary Figure 169. ^{1}H NMR (400 MHz, $CDCl_3$) spectra for compound **37**

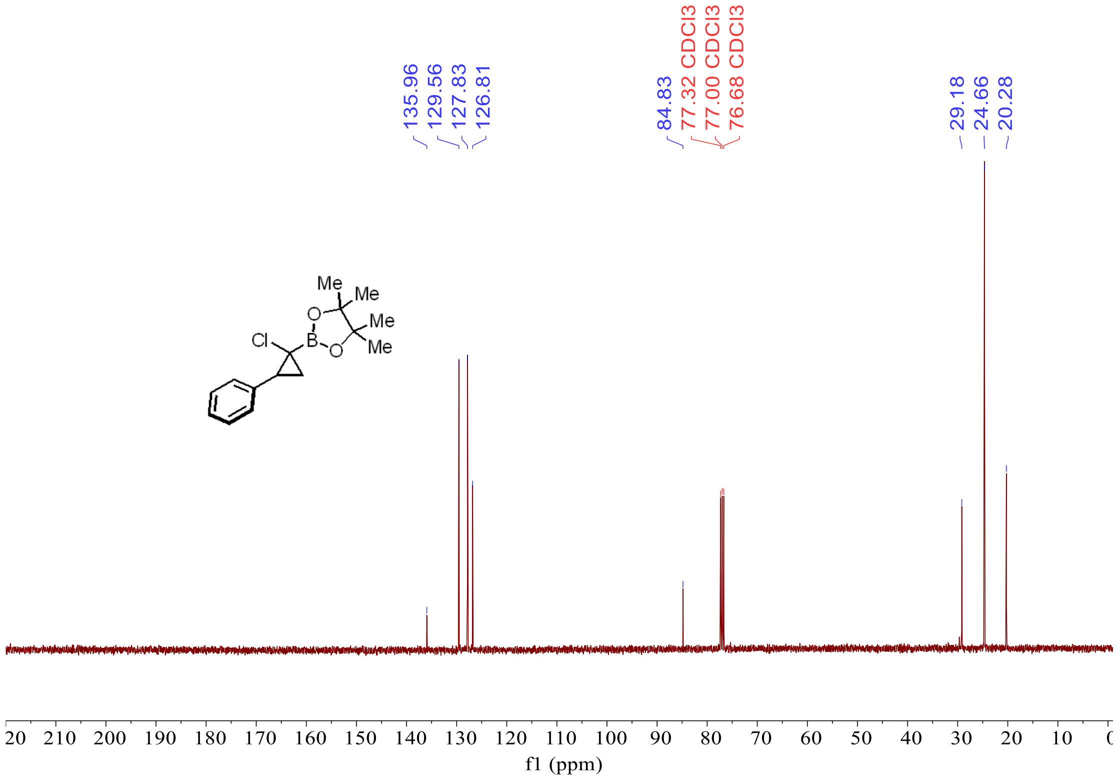

Supplementary Figure 170. ^{13}C NMR (101 MHz, $CDCl_3$) spectra for compound **37**

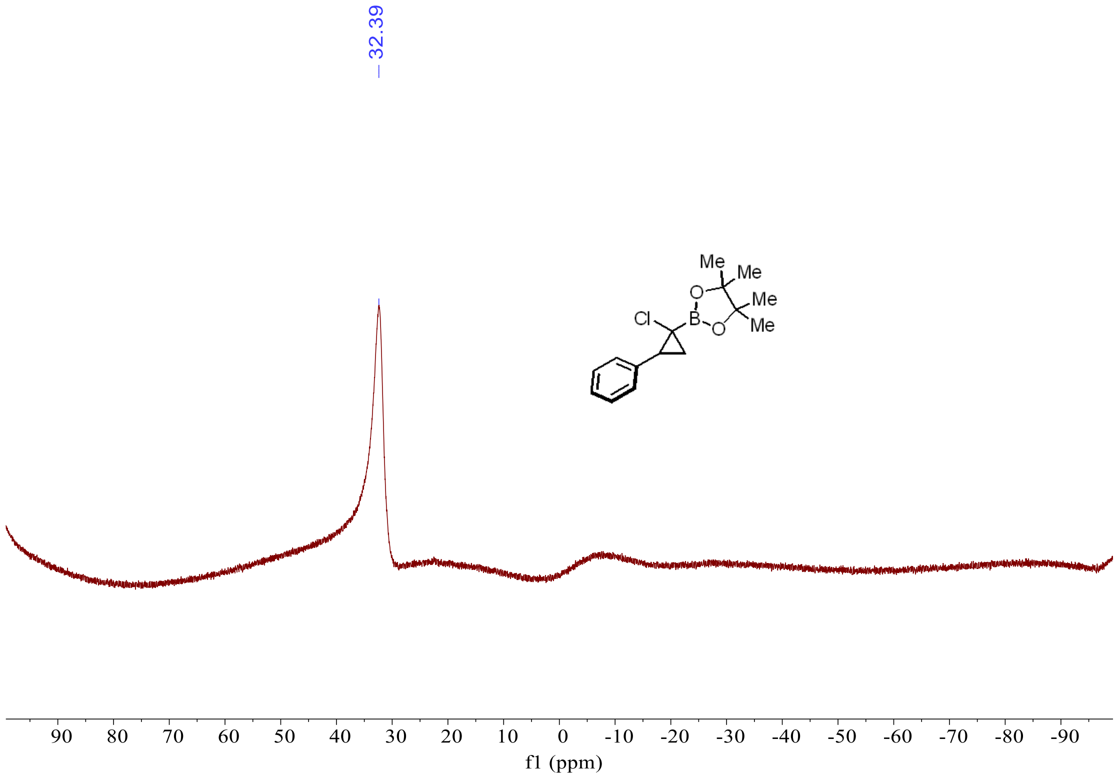

Supplementary Figure 171. ^{11}B NMR (128 MHz, $CDCl_3$) spectra for compound **37**

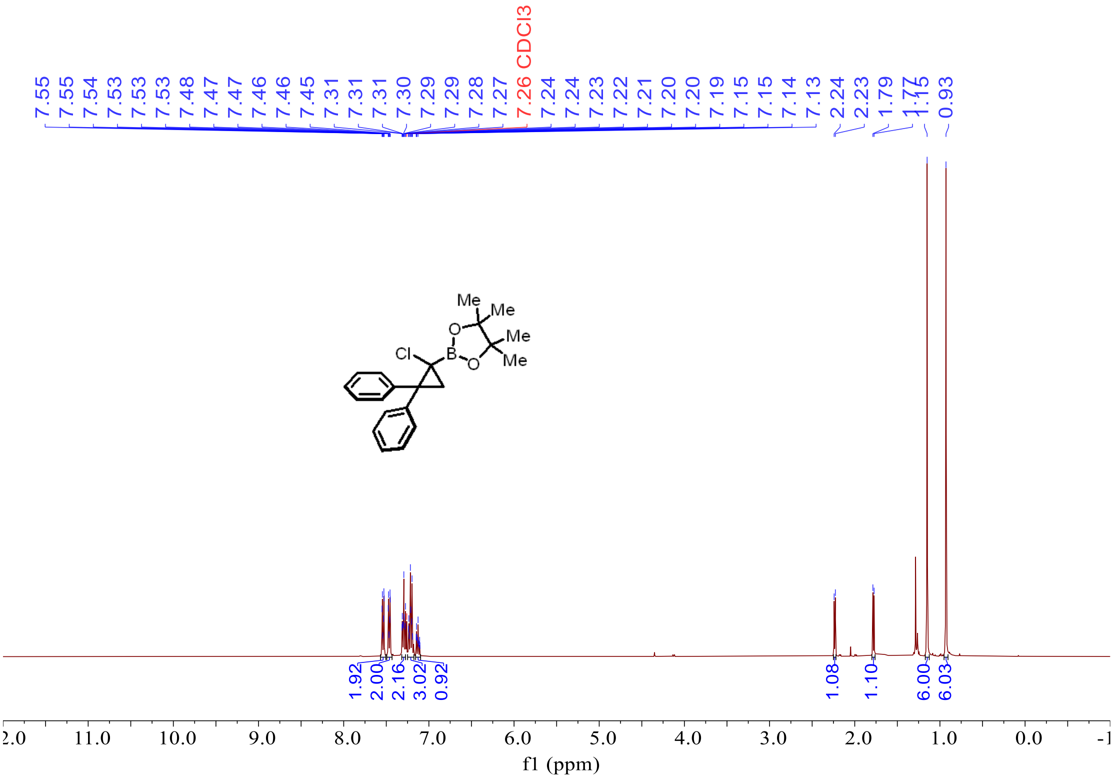

Supplementary Figure 172. ^{1}H NMR (400 MHz, $CDCl_3$) spectra for compound **38**

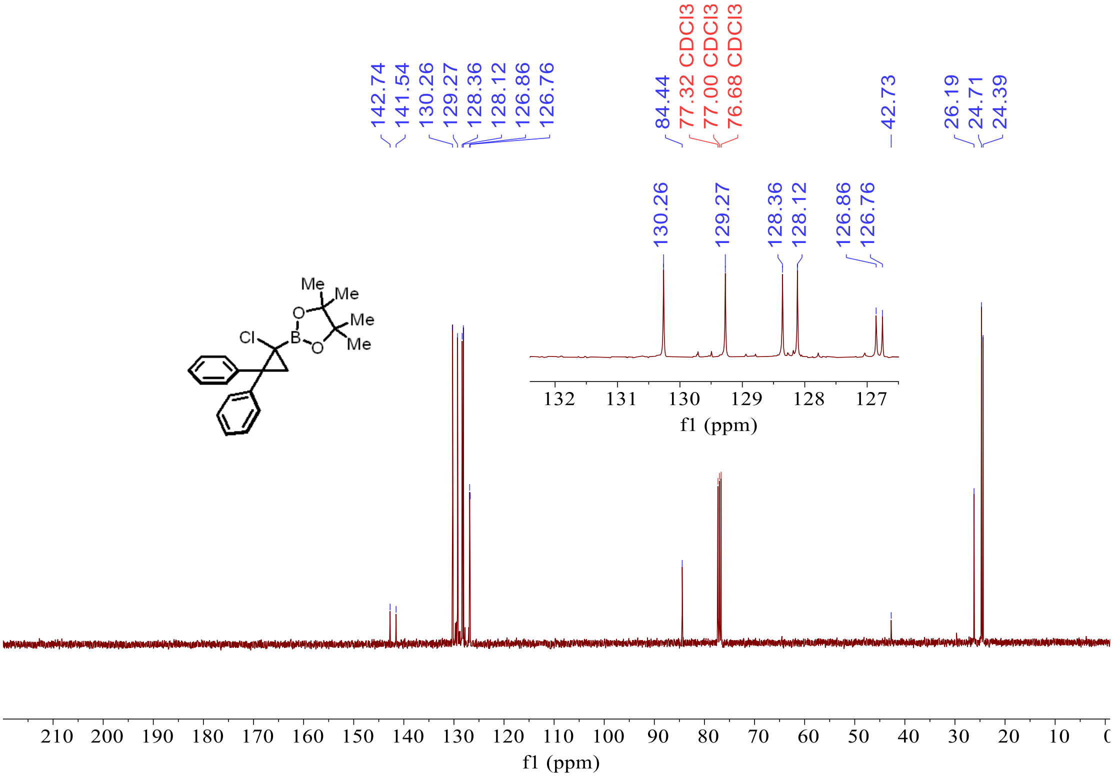

Supplementary Figure 173. ^{13}C NMR (101 MHz, $CDCl_3$) spectra for compound **38**

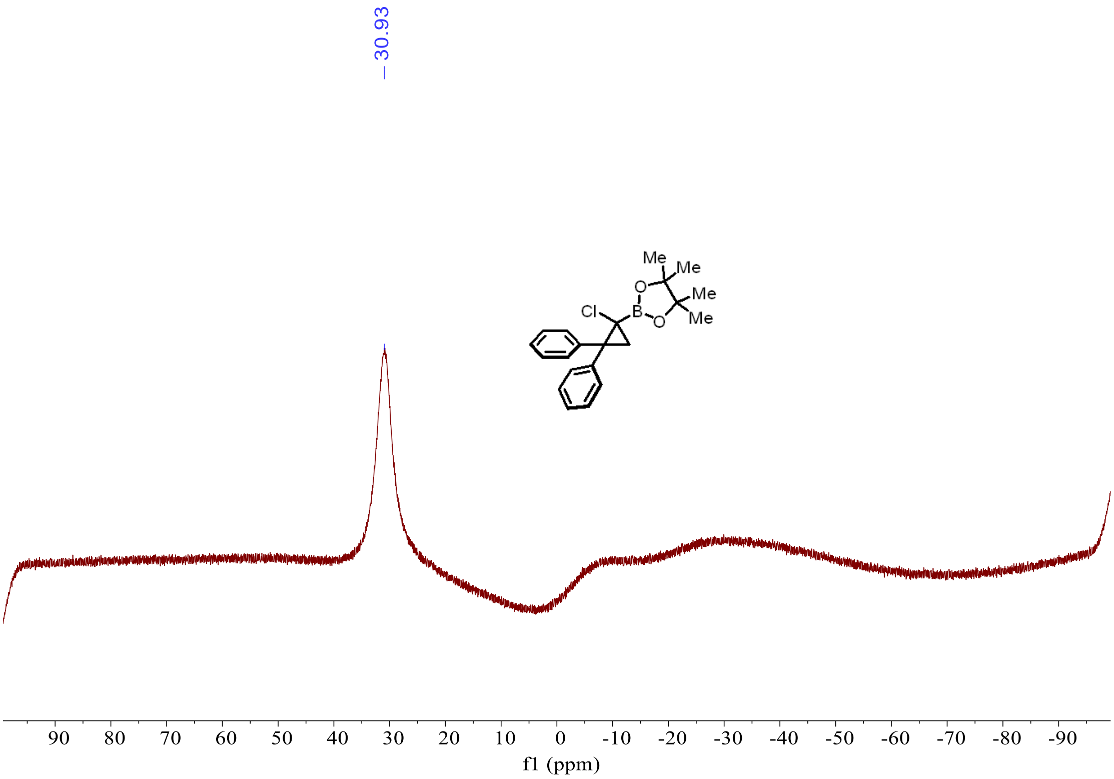

Supplementary Figure 174. ^{11}B NMR (128 MHz, $CDCl_3$) spectra for compound **38**

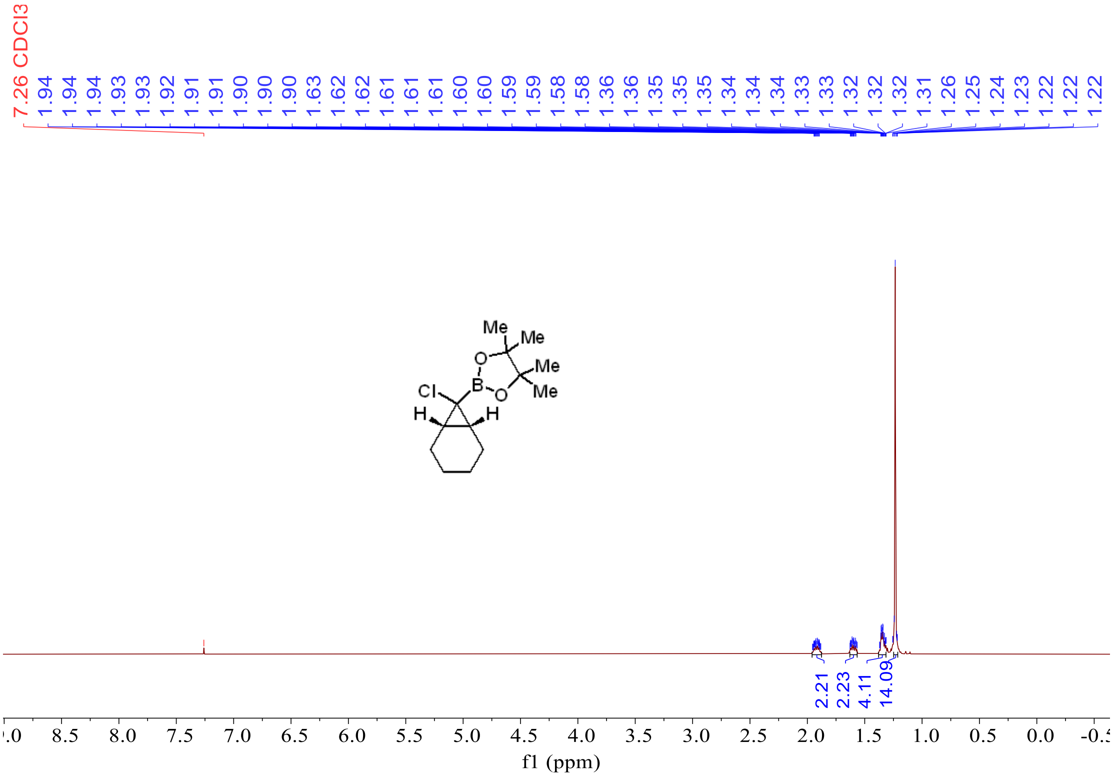

Supplementary Figure 175. ^{1}H NMR (500 MHz, $CDCl_3$) spectra for compound **39**

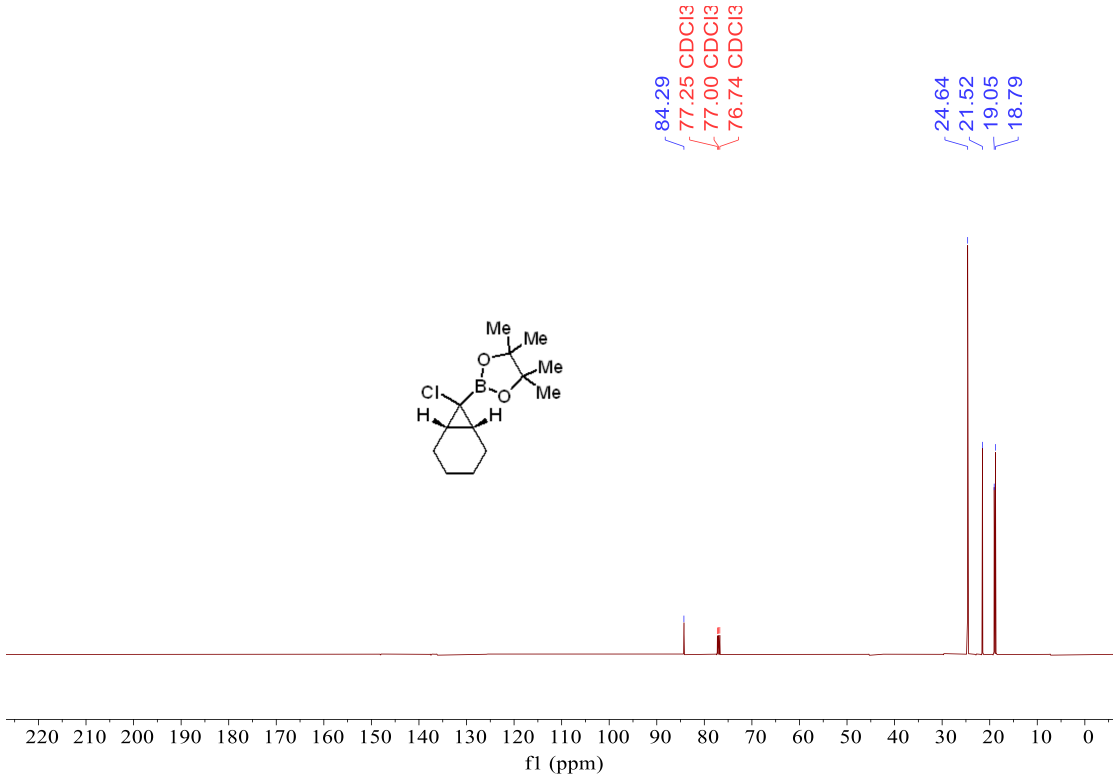

Supplementary Figure 176. ^{13}C NMR (126 MHz, $CDCl_3$) spectra for compound **39**

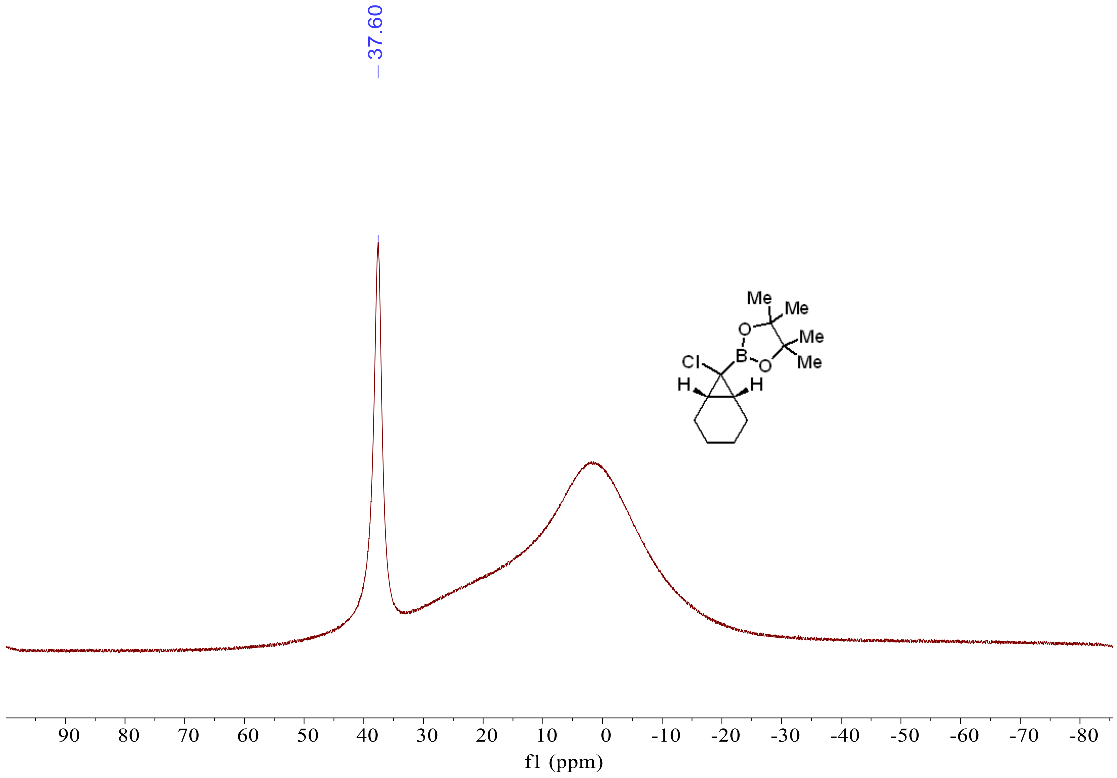

Supplementary Figure 177. ^{11}B NMR (160 MHz, $CDCl_3$) spectra for compound **39**

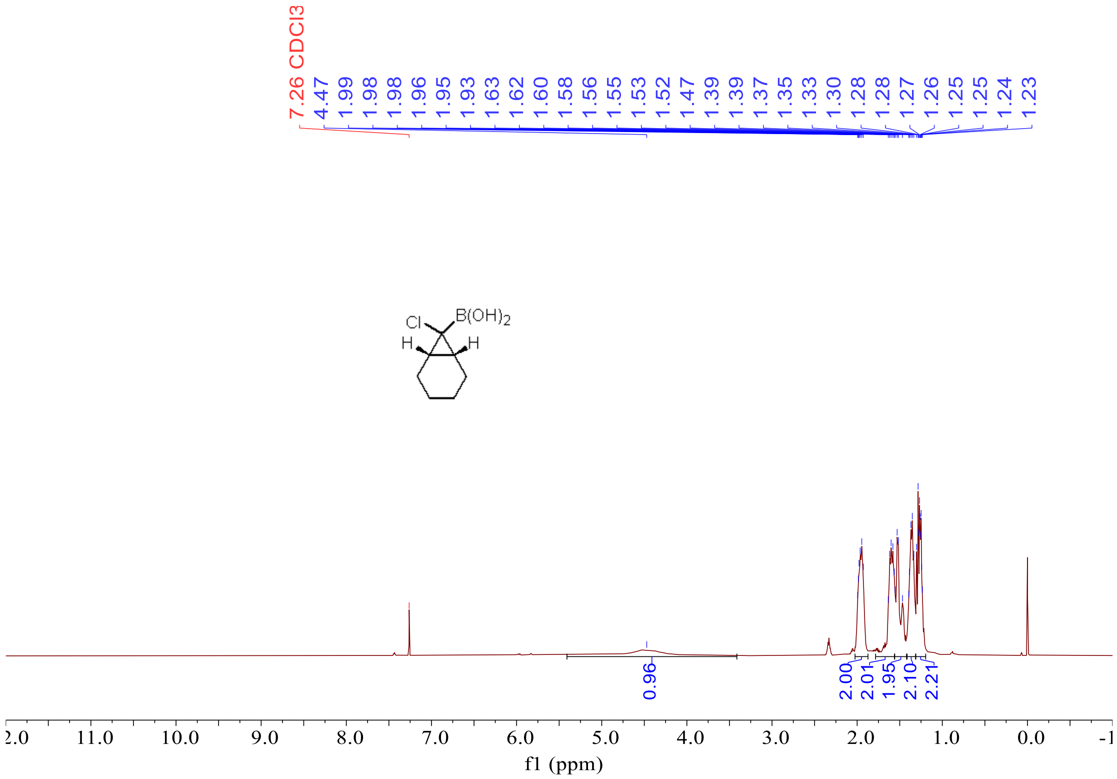

Supplementary Figure 178. ^{1}H NMR (400 MHz, $CDCl_3$) spectra for compound **39a**

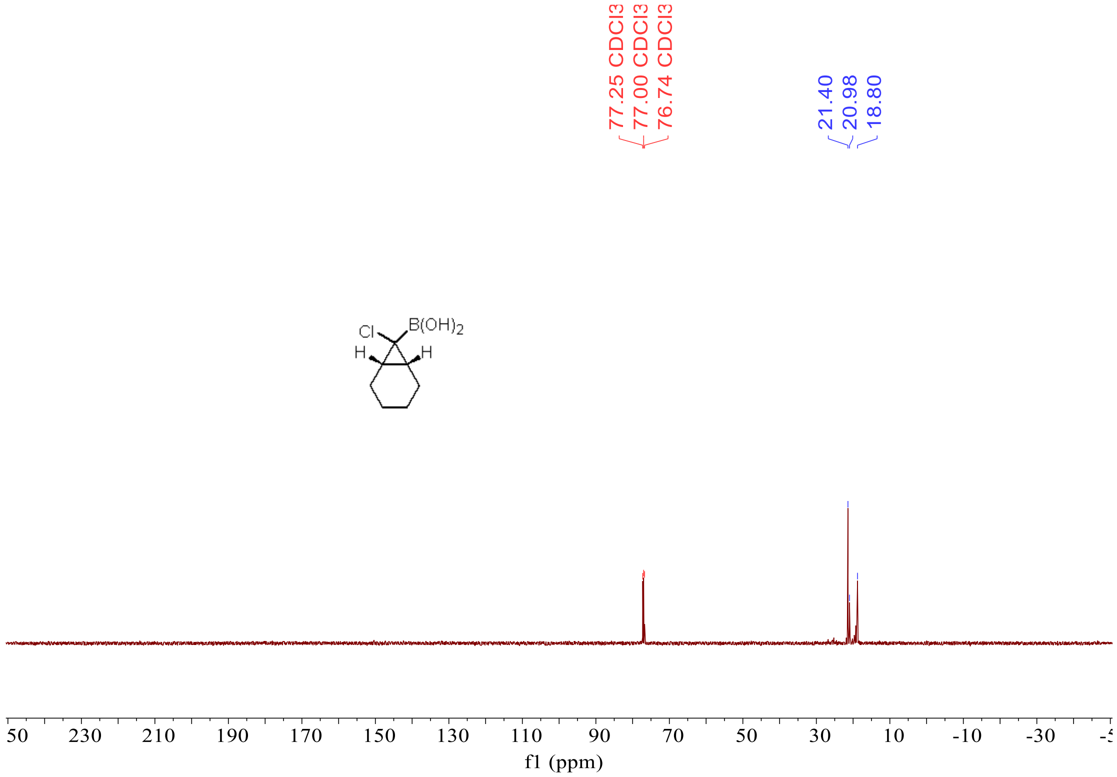

Supplementary Figure 179. ^{13}C NMR (126 MHz, $CDCl_3$) spectra for compound **39a**

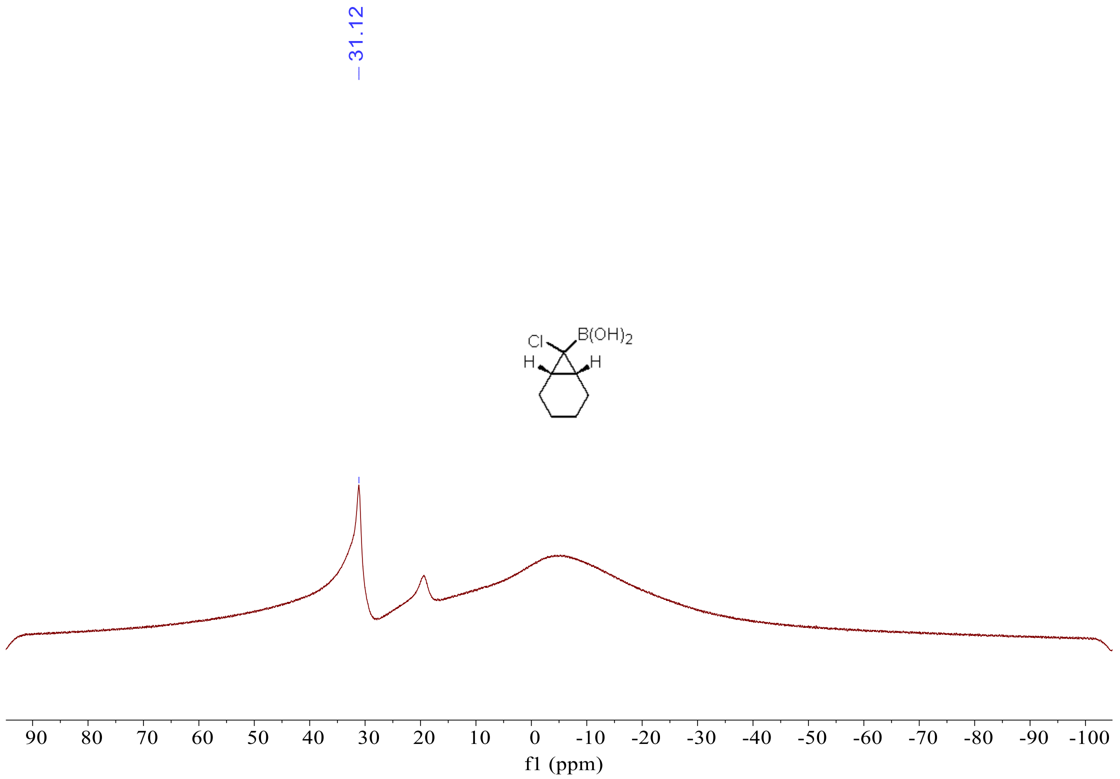

Supplementary Figure 180. ^{11}B NMR (160 MHz, $CDCl_3$) spectra for compound **39a**

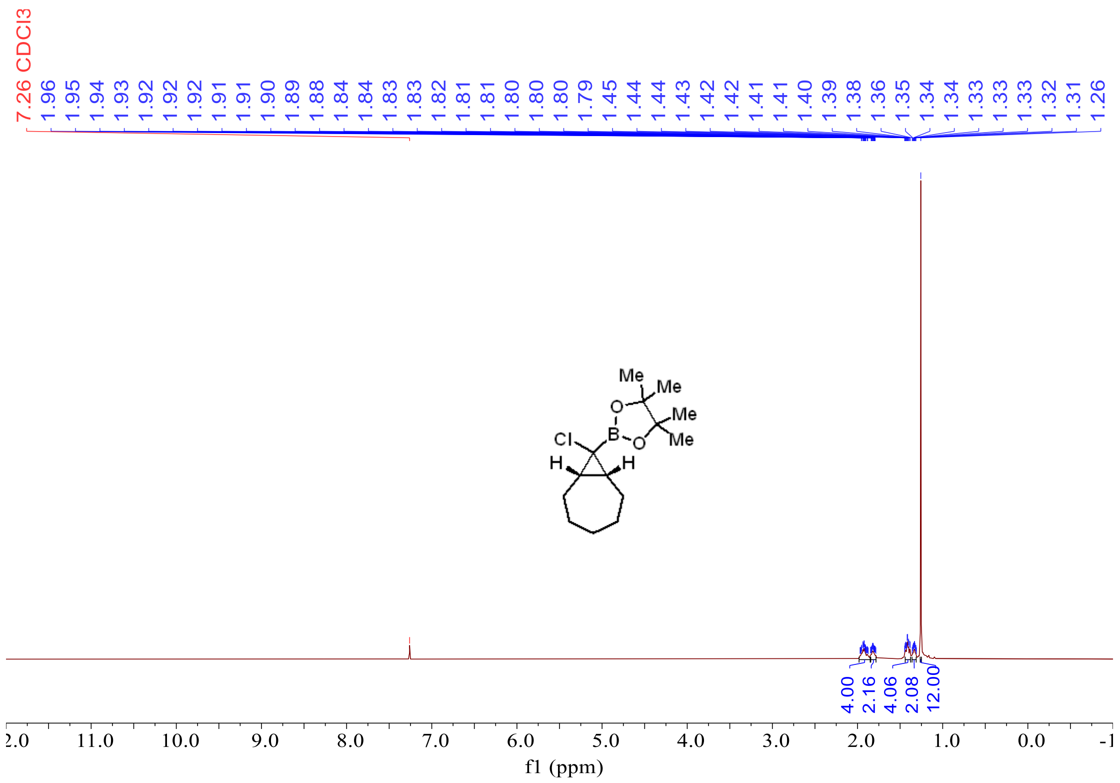

Supplementary Figure 181. ^{1}H NMR (400 MHz, $CDCl_3$) spectra for compound **40**

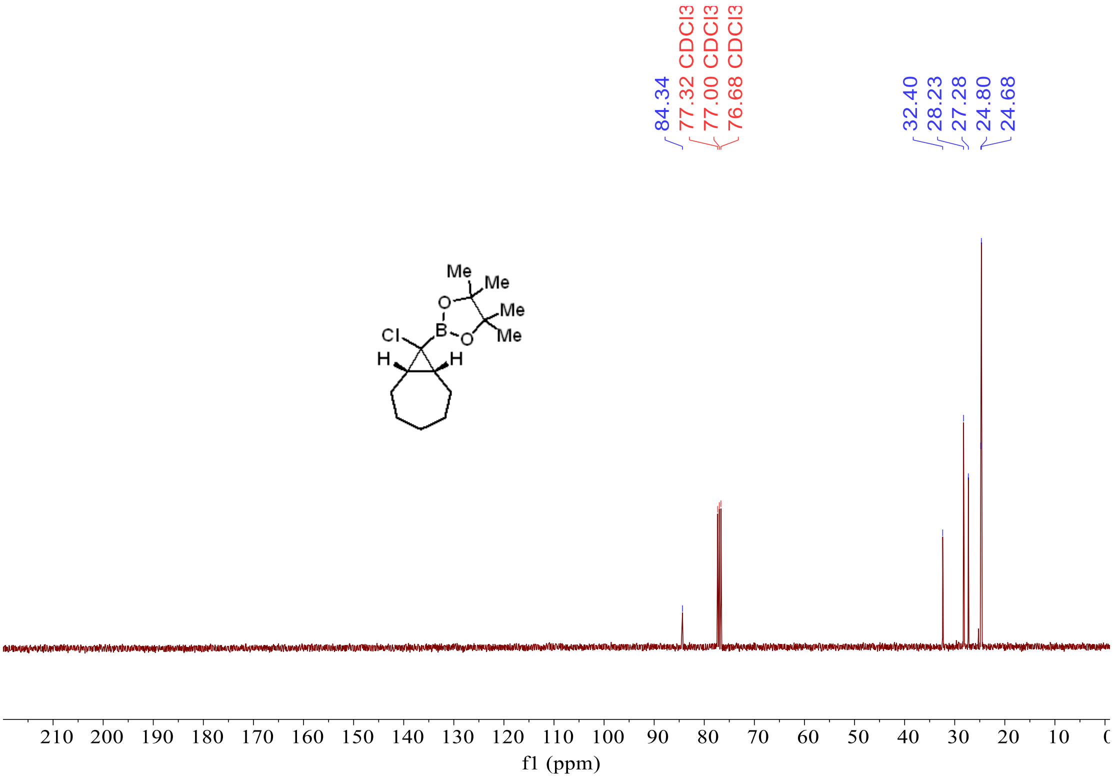

Supplementary Figure 182. ^{13}C NMR (101 MHz, $CDCl_3$) spectra for compound **40**

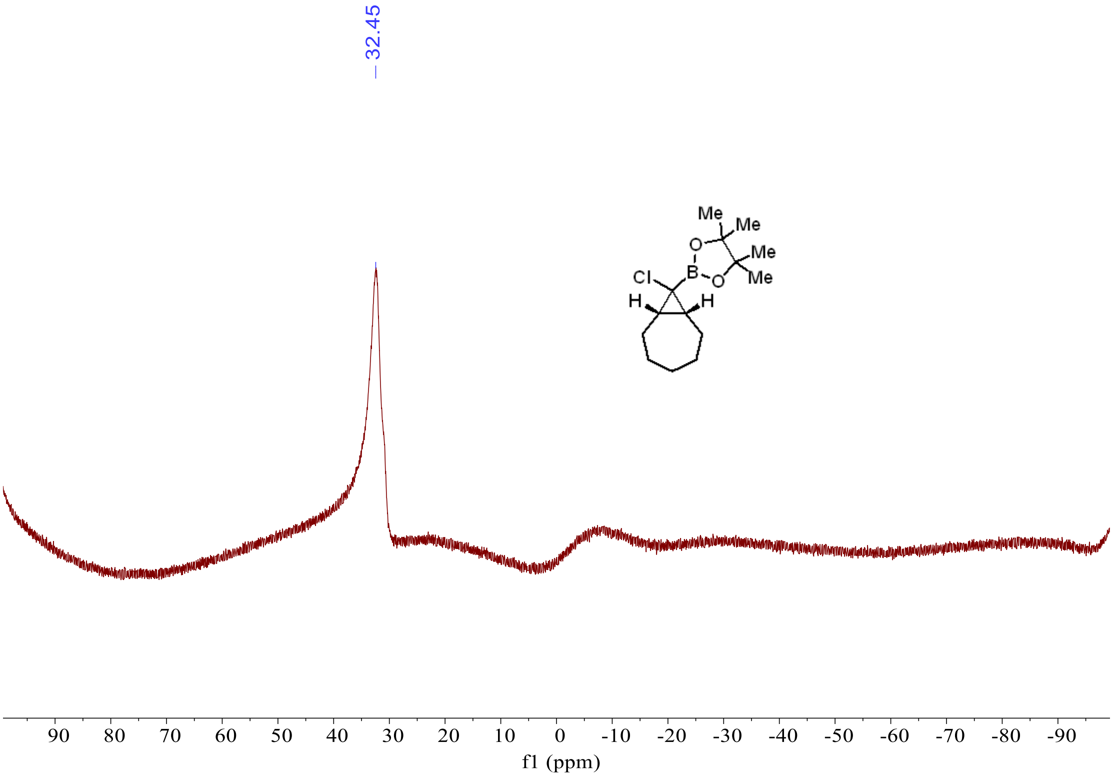

Supplementary Figure 183. ^{11}B NMR (128 MHz, $CDCl_3$) spectra for compound **40**

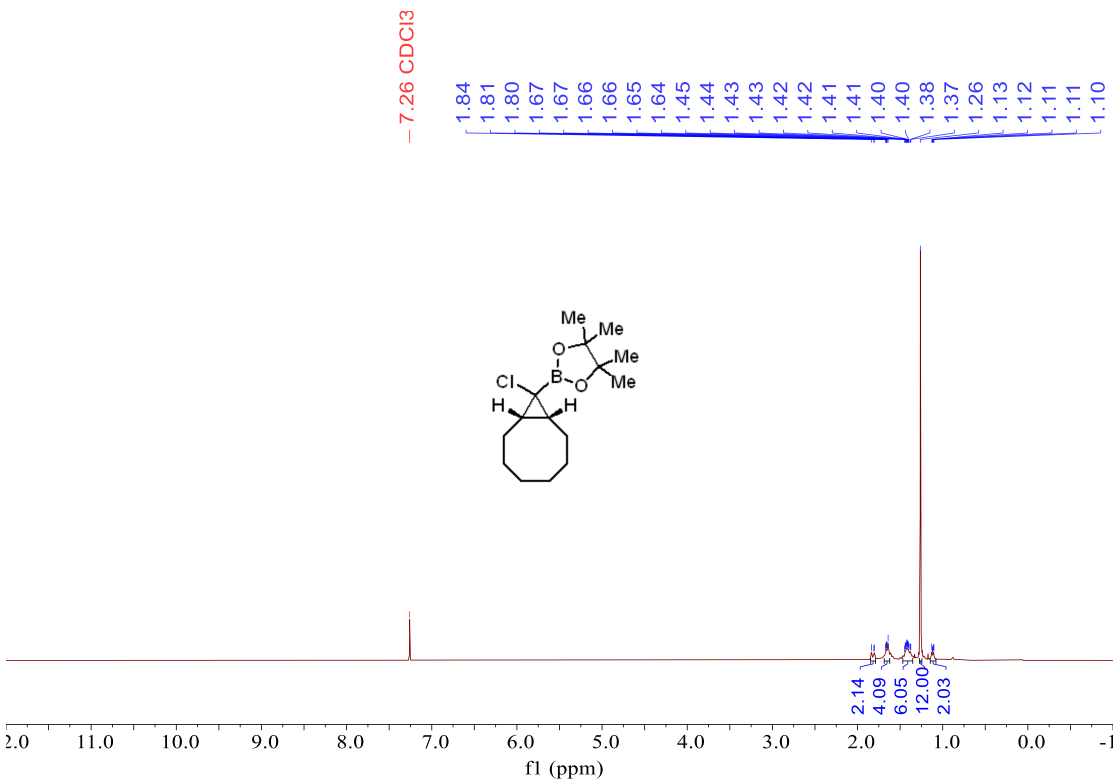

Supplementary Figure 184. ^{1}H NMR (400 MHz, $CDCl_3$) spectra for compound **41**

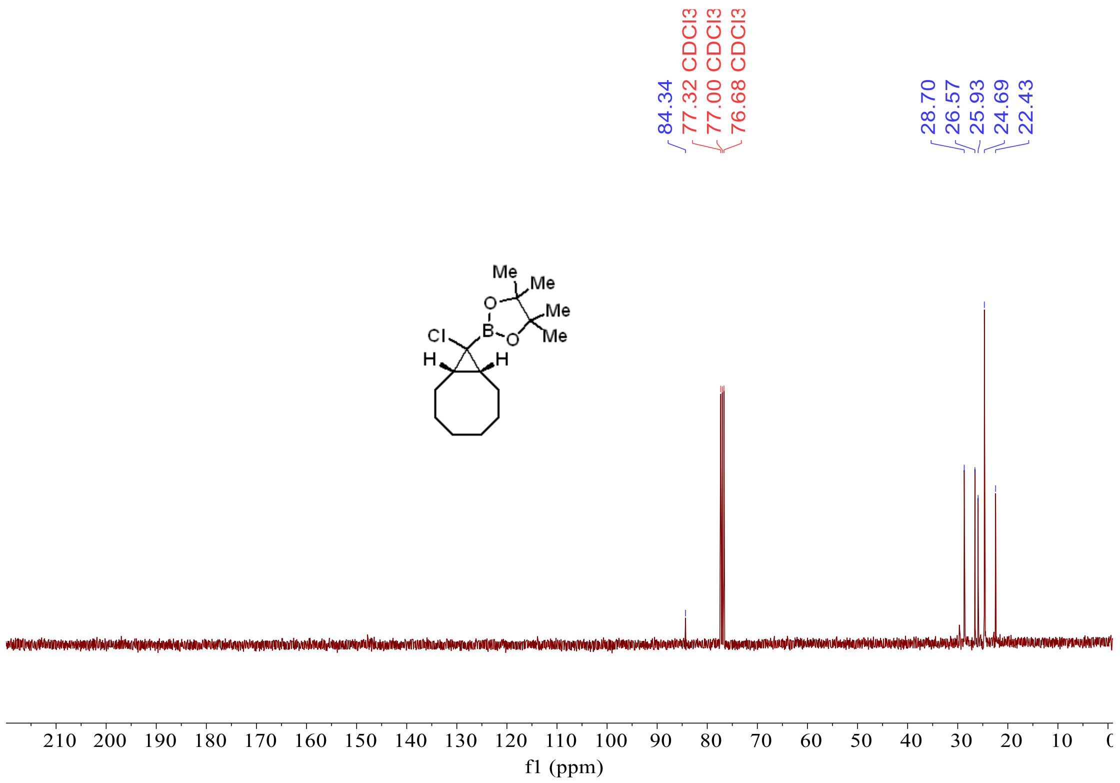

Supplementary Figure 185. ^{13}C NMR (101 MHz, $CDCl_3$) spectra for compound **41**

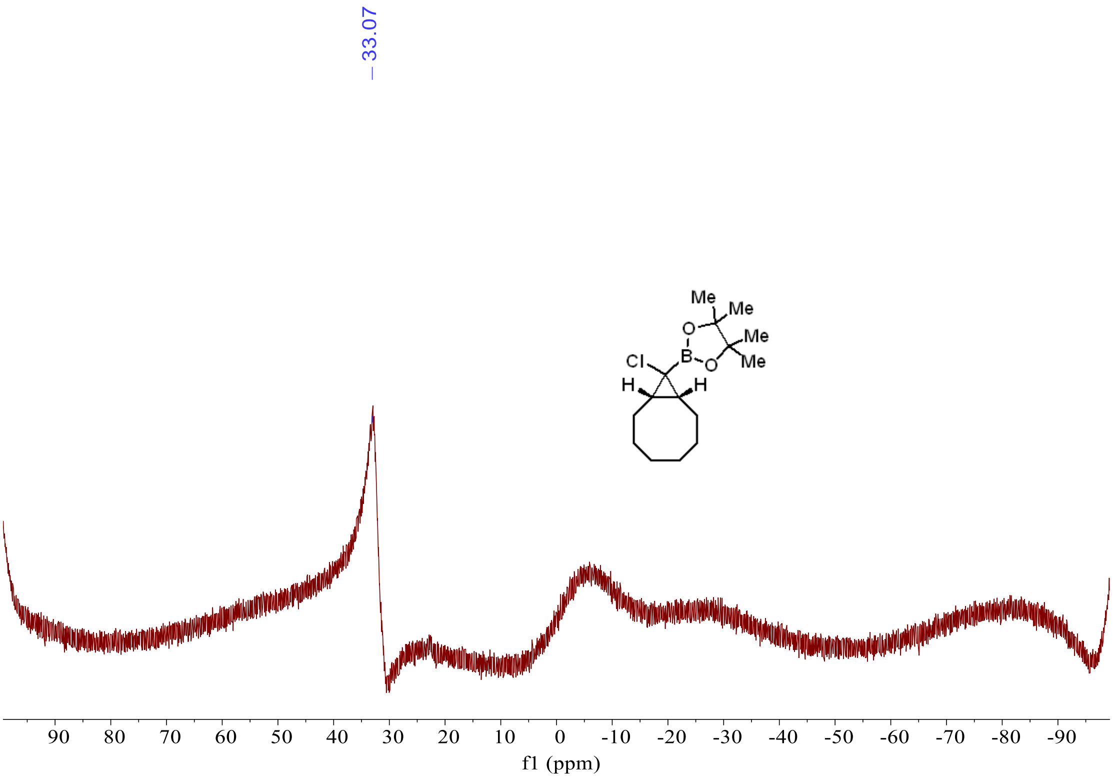

Supplementary Figure 186. ^{11}B NMR (128 MHz, $CDCl_3$) spectra for compound **41**

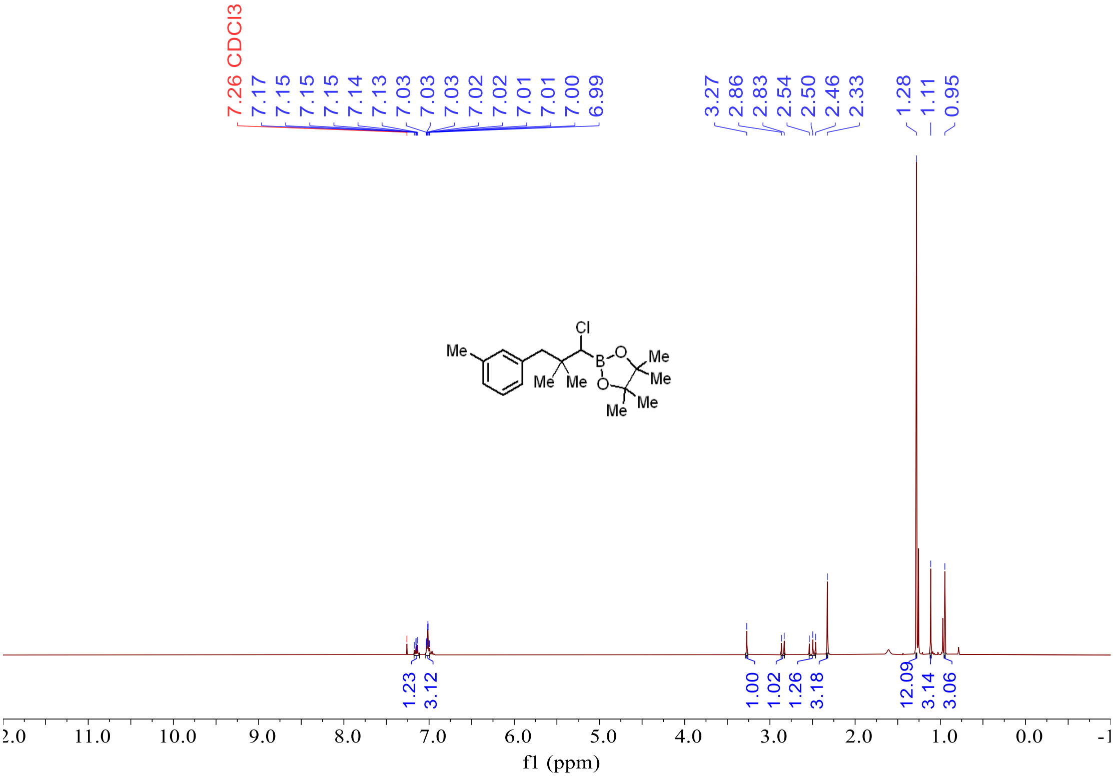

Supplementary Figure 187. ^{1}H NMR (400 MHz, $CDCl_3$) spectra for compound **42**

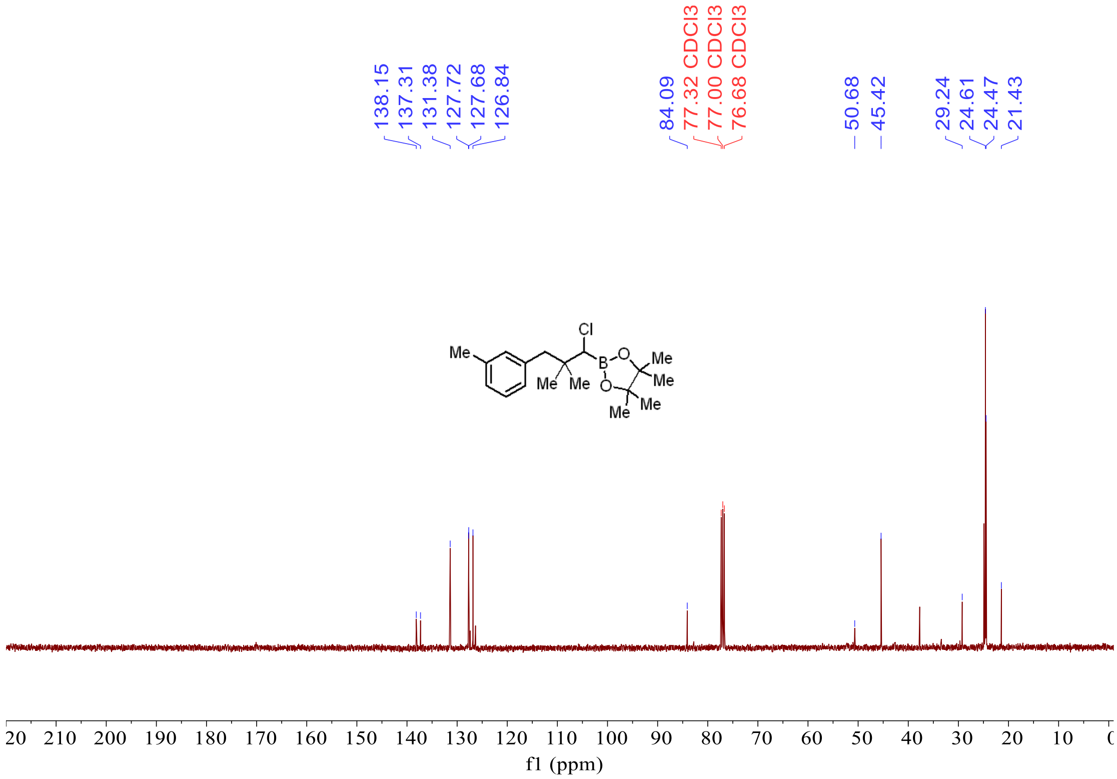

Supplementary Figure 188. ^{13}C NMR (101 MHz, $CDCl_3$) spectra for compound **42**

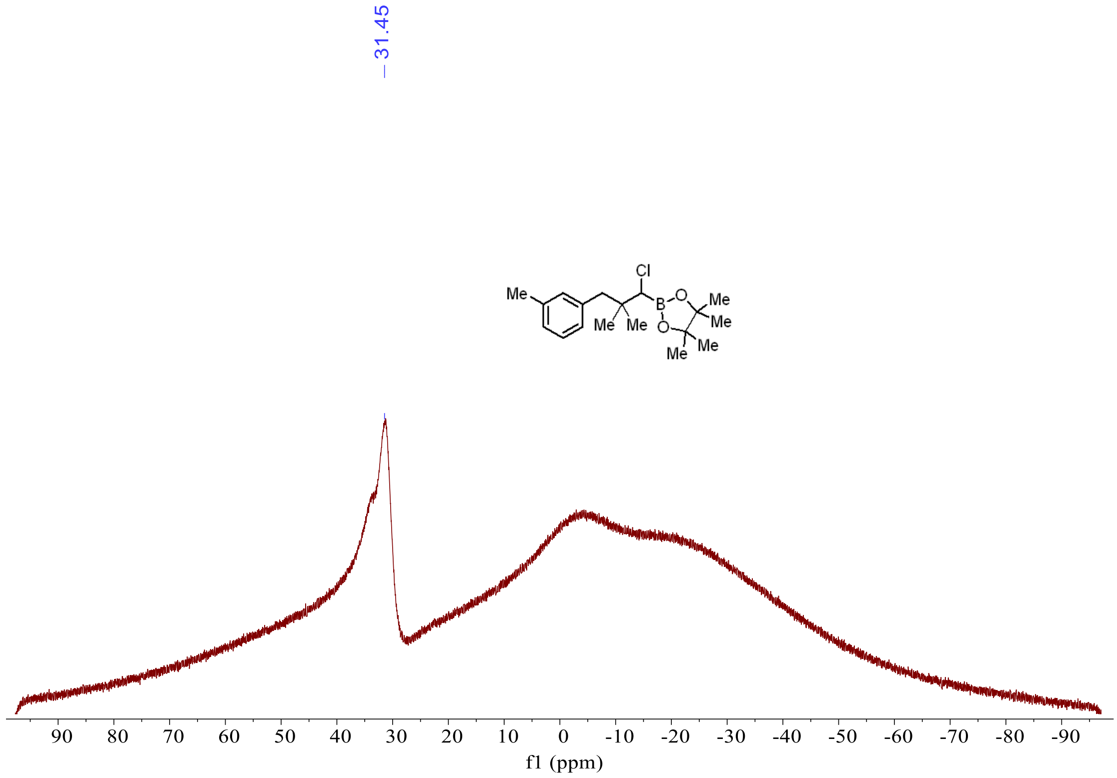

Supplementary Figure 189. ^{11}B NMR (128 MHz, $CDCl_3$) spectra for compound **42**

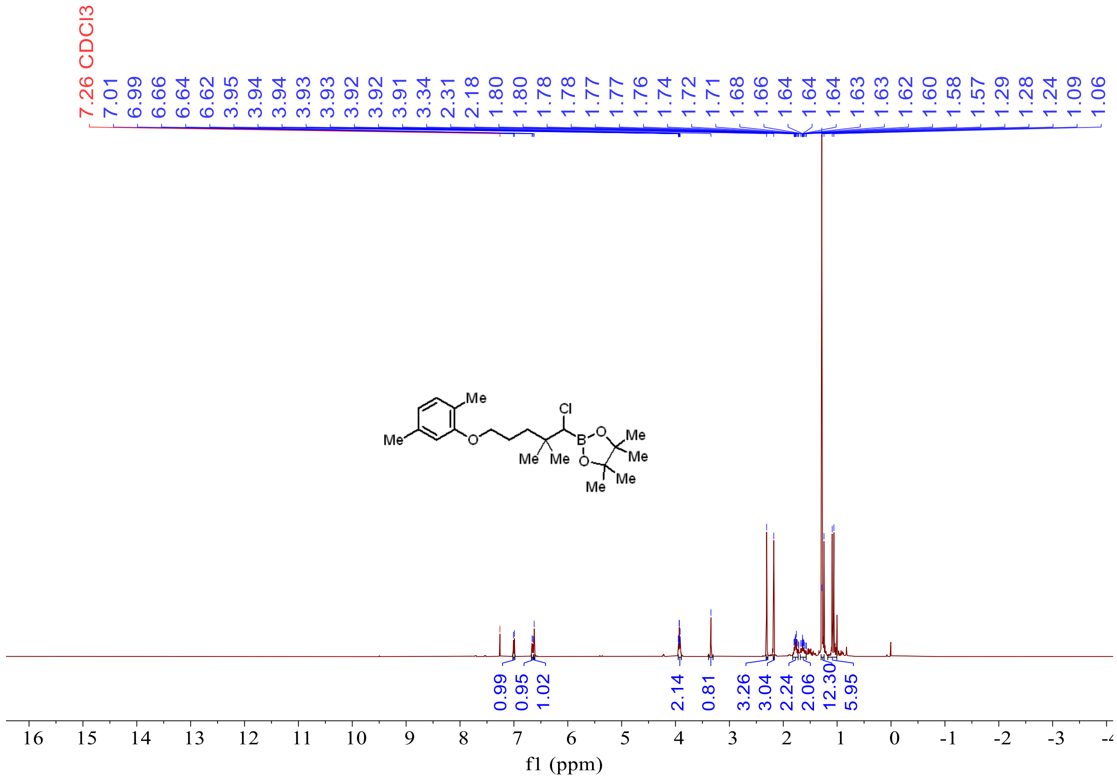

Supplementary Figure 190. ^{1}H NMR (400 MHz, $CDCl_3$) spectra for compound **43**

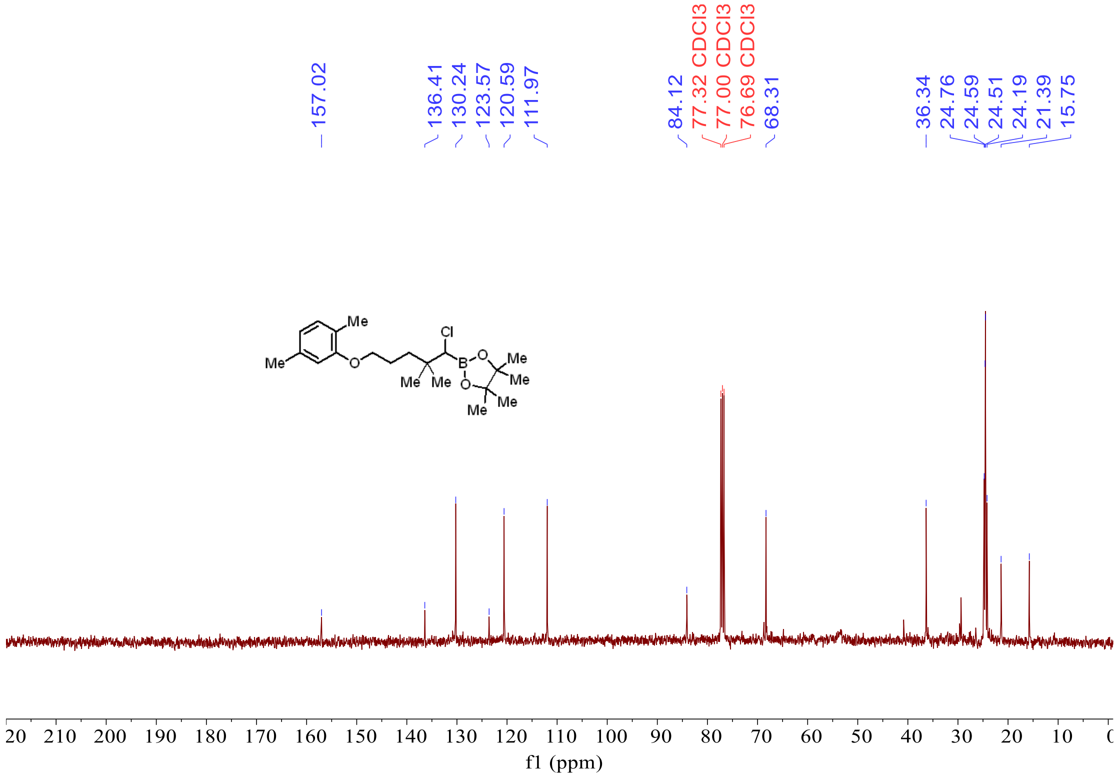

Supplementary Figure 191. ^{13}C NMR (101 MHz, $CDCl_3$) spectra for compound **43**

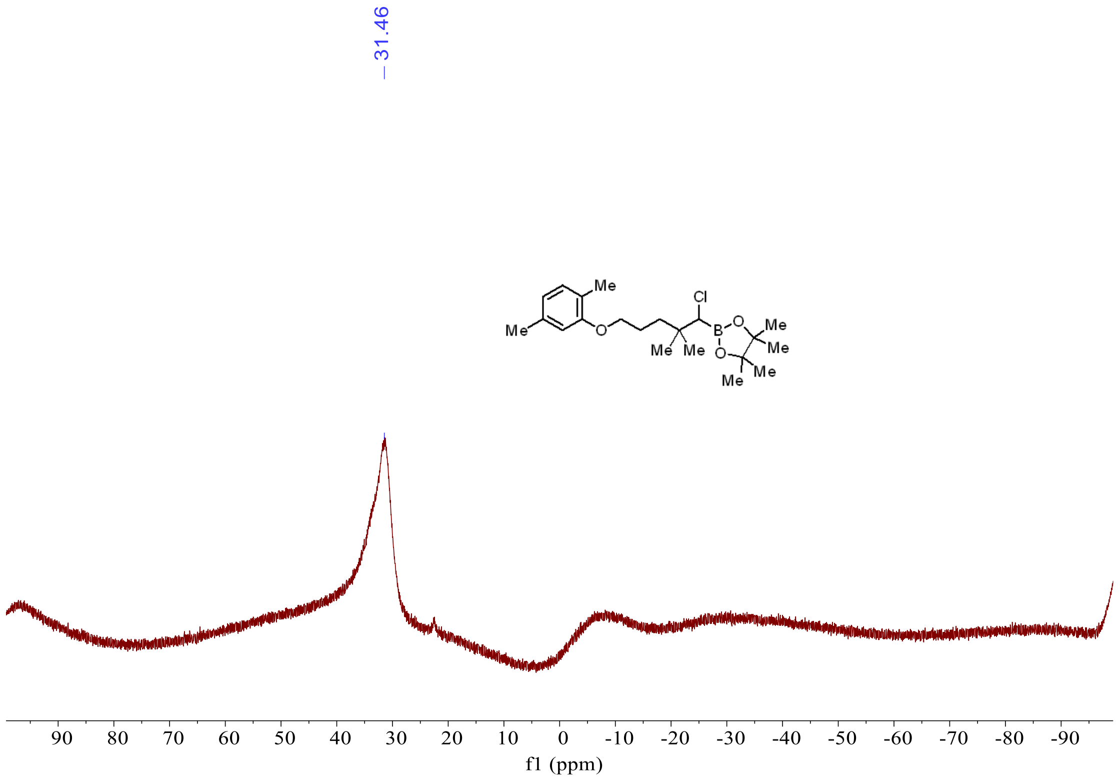

Supplementary Figure 192. ^{11}B NMR (128 MHz, $CDCl_3$) spectra for compound **43**

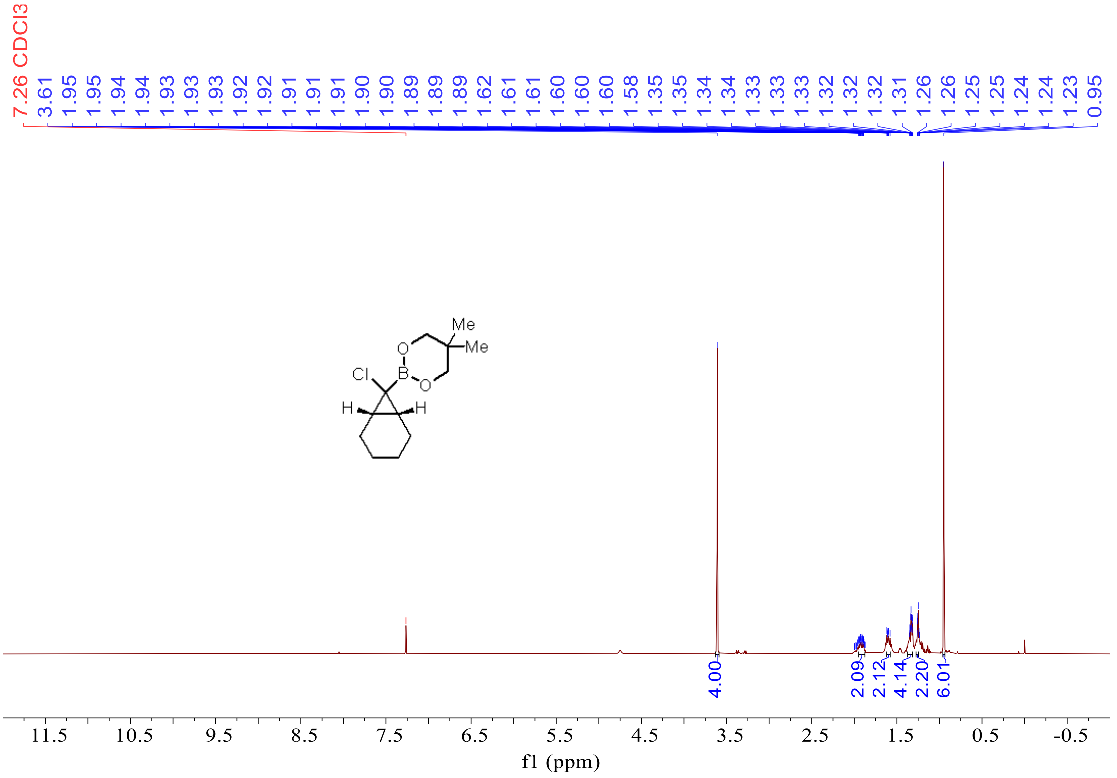

Supplementary Figure 193. ^{1}H NMR (400 MHz, $CDCl_3$) spectra for compound **44**

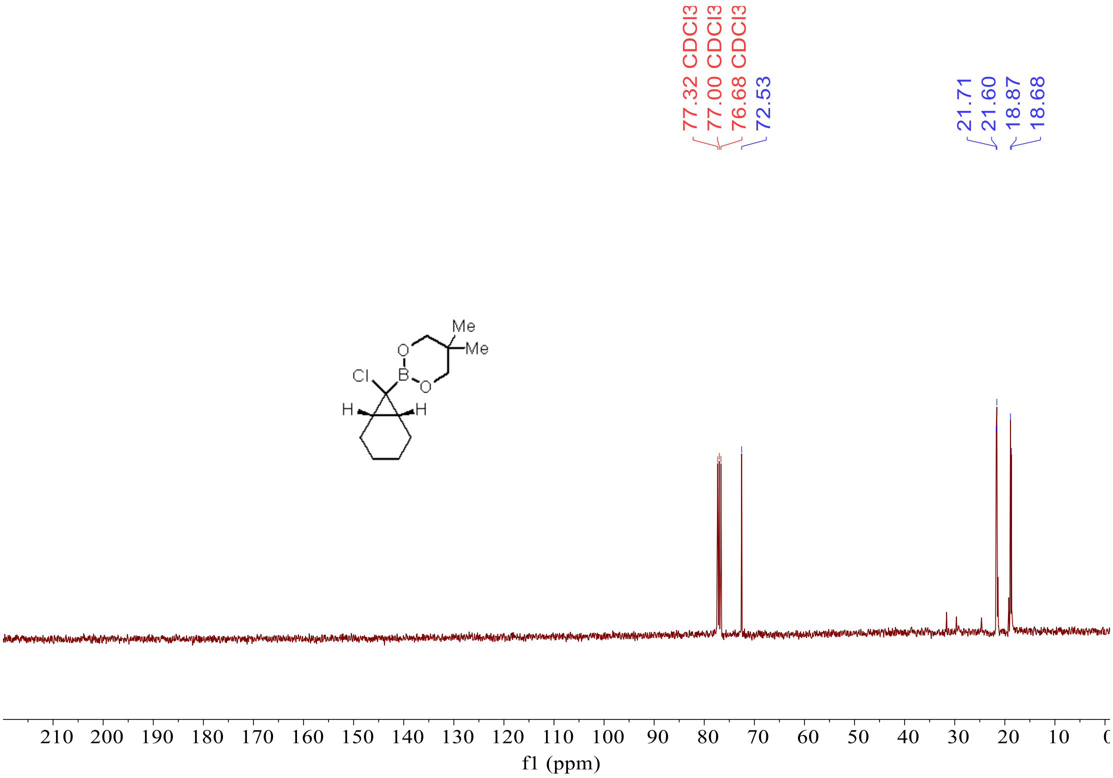

Supplementary Figure 194. ^{13}C NMR (101 MHz, $CDCl_3$) spectra for compound **44**

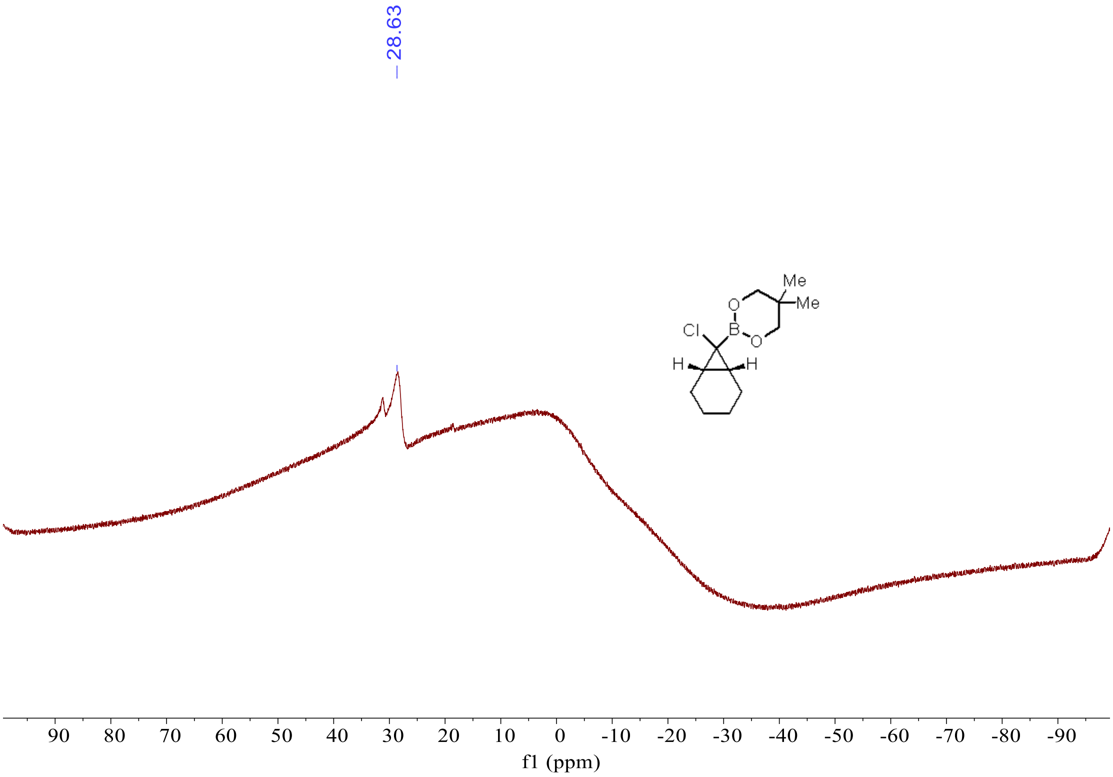

Supplementary Figure 195. ^{11}B NMR (128 MHz, $CDCl_3$) spectra for compound **44**

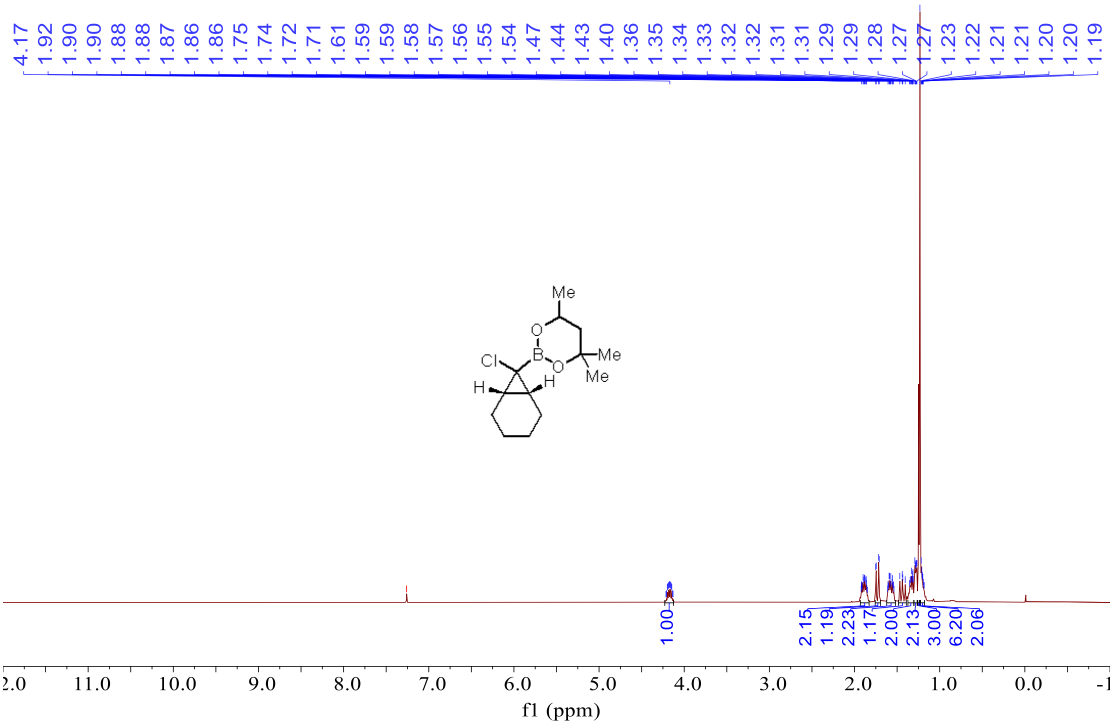

Supplementary Figure 196. ^{1}H NMR (400 MHz, $CDCl_3$) spectra for compound **45**

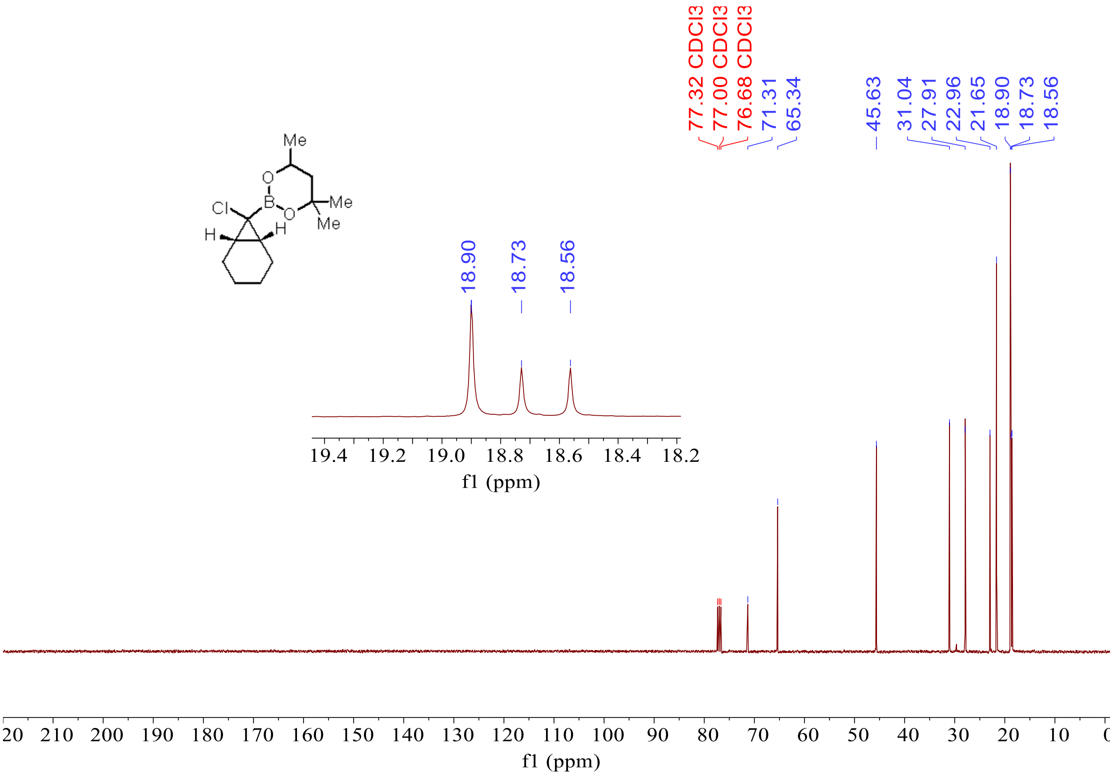

Supplementary Figure 197. ^{13}C NMR (101 MHz, $CDCl_3$) spectra for compound **45**

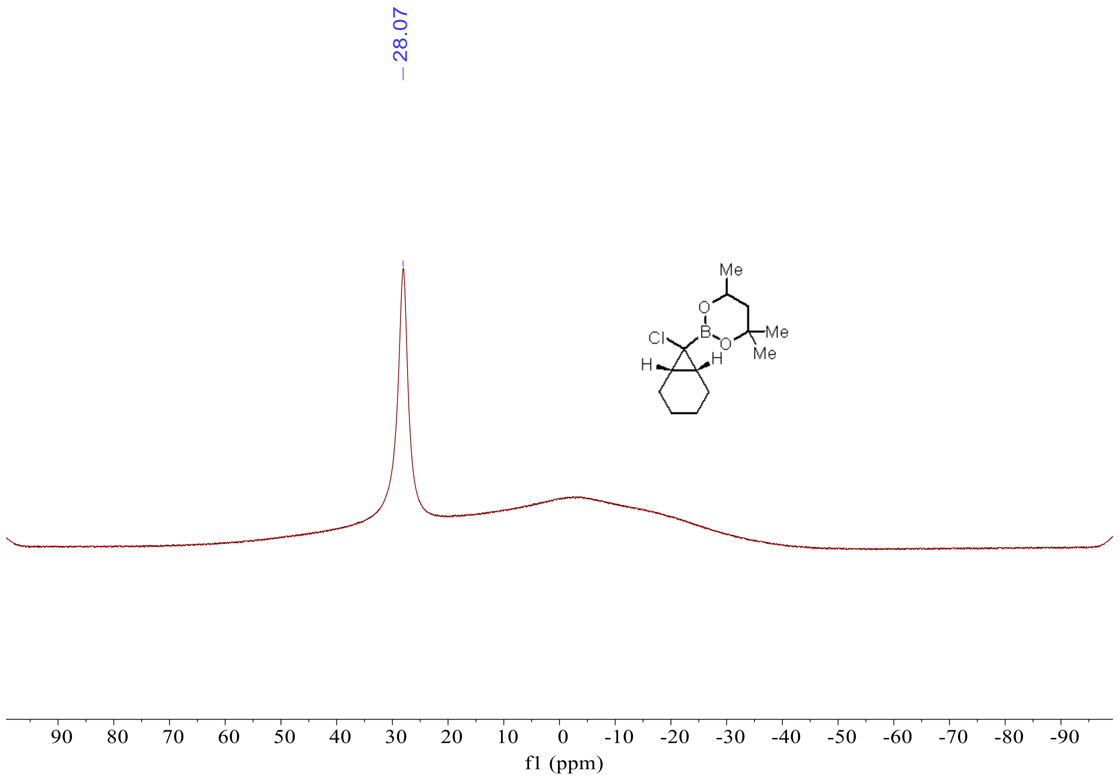

Supplementary Figure 198. ^{11}B NMR (128 MHz, $CDCl_3$) spectra for compound **45**

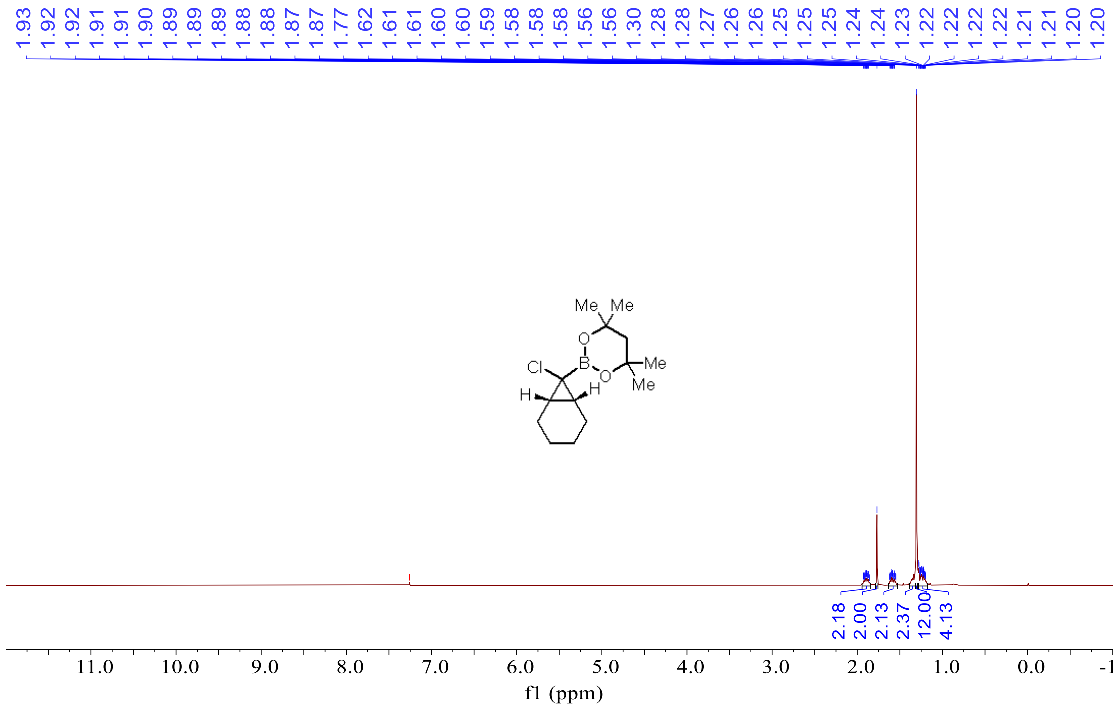

Supplementary Figure 199. ^{1}H NMR (400 MHz, $CDCl_3$) spectra for compound **46**

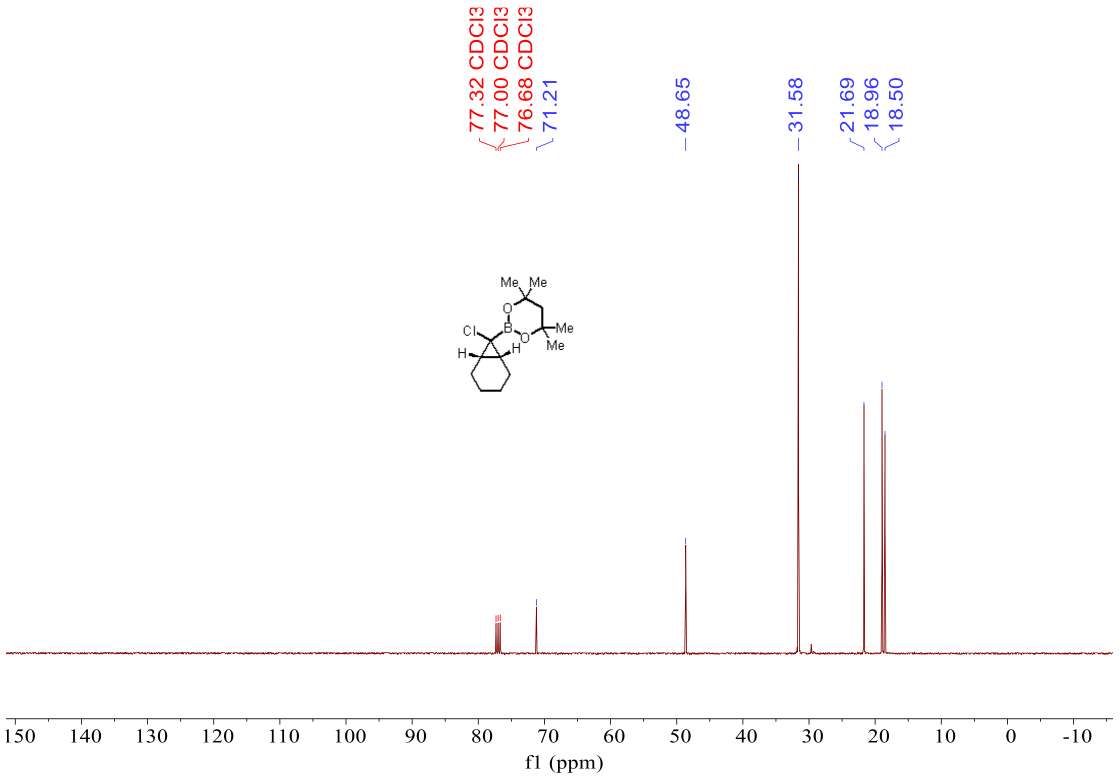

Supplementary Figure 200. ^{13}C NMR (101 MHz, $CDCl_3$) spectra for compound **46**

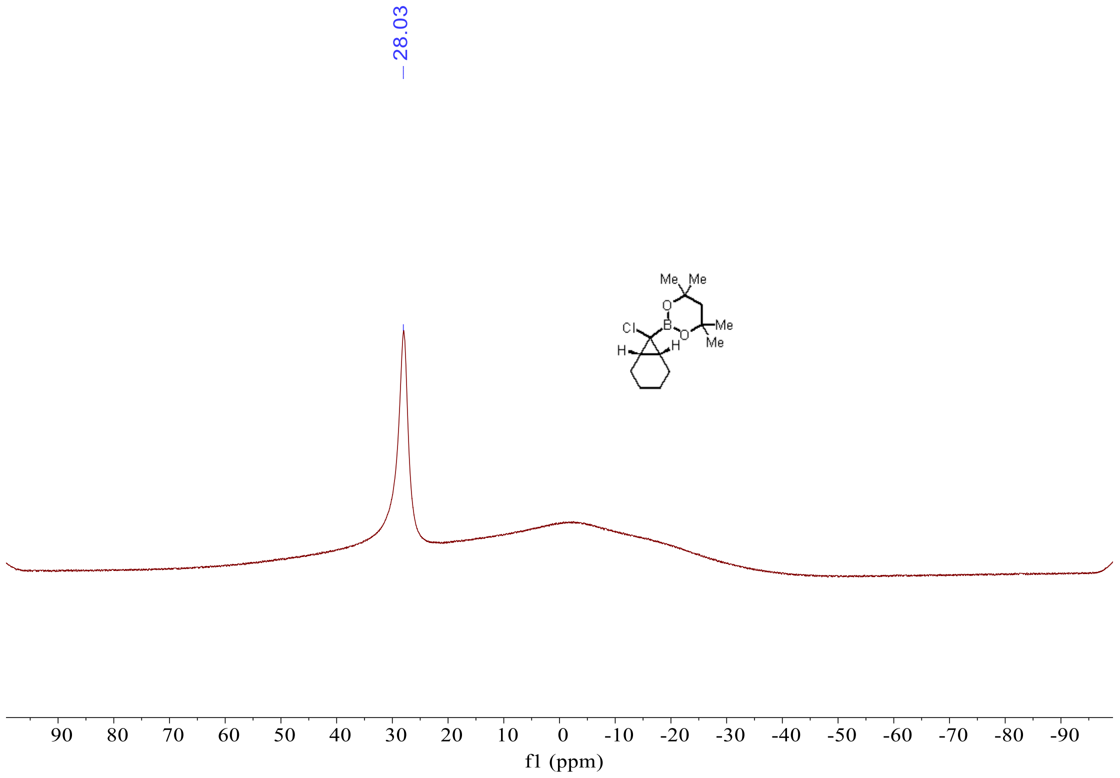

Supplementary Figure 201. ^{11}B NMR (128 MHz, $CDCl_3$) spectra for compound **46**

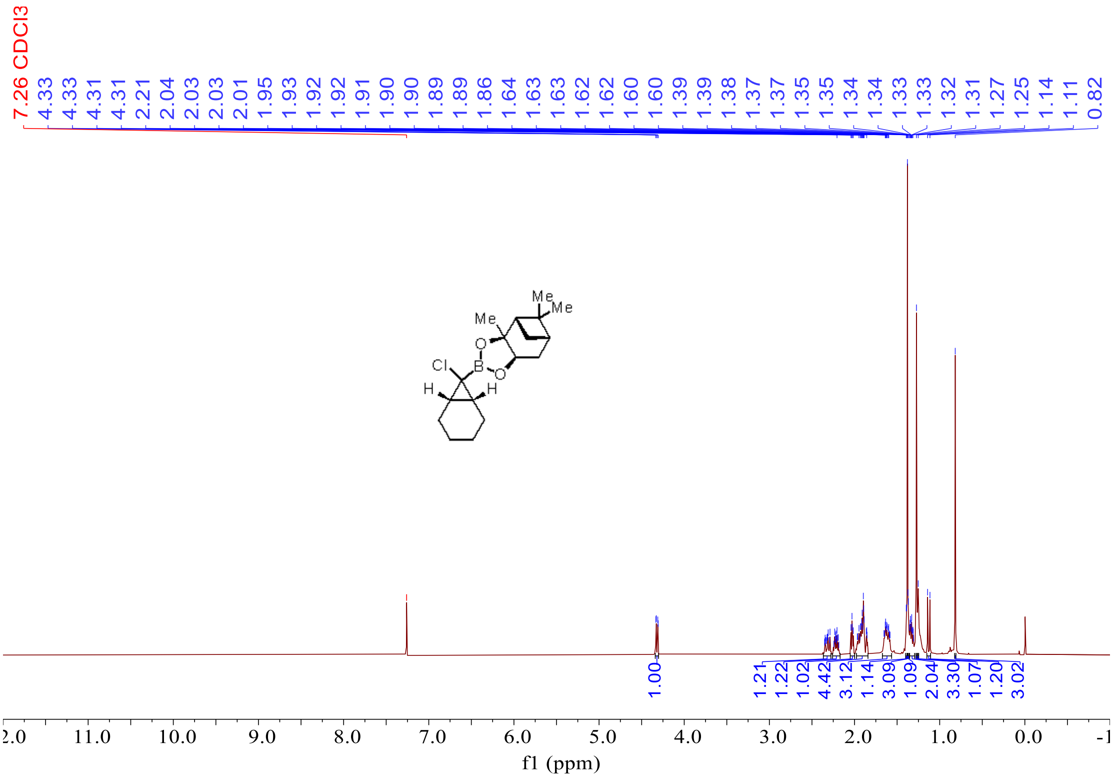

Supplementary Figure 202. ^{1}H NMR (400 MHz, $CDCl_3$) spectra for compound **47**

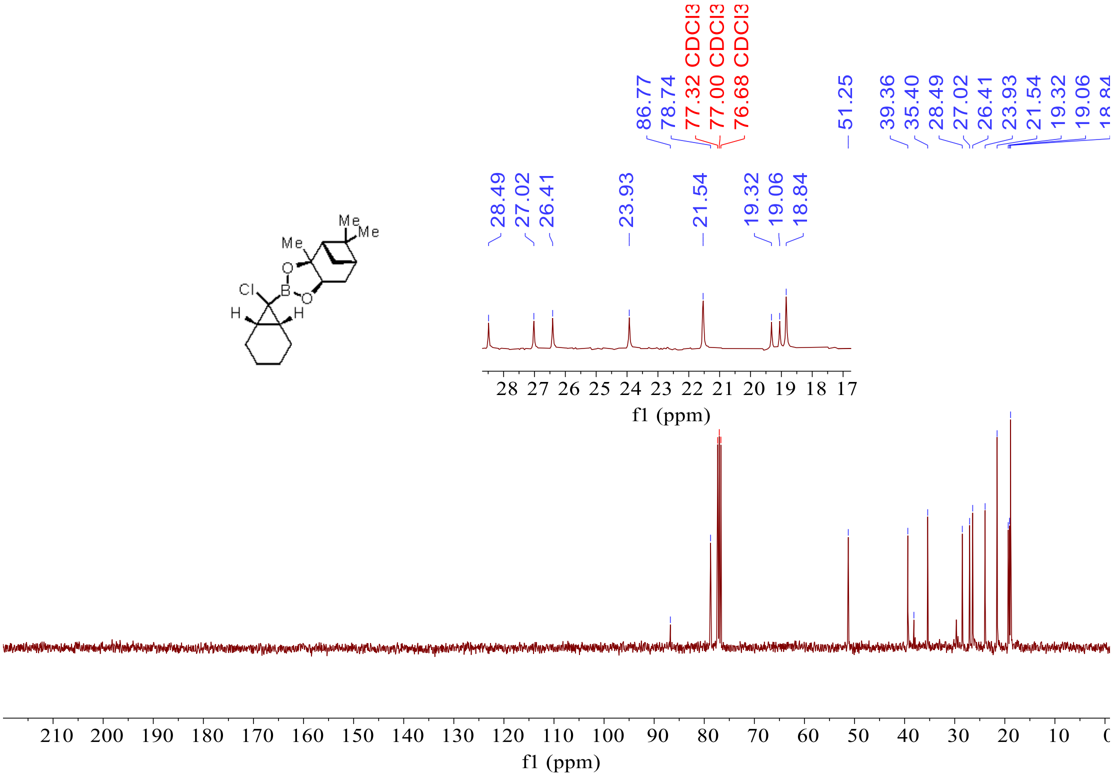

Supplementary Figure 203. ^{13}C NMR (101 MHz, $CDCl_3$) spectra for compound **47**

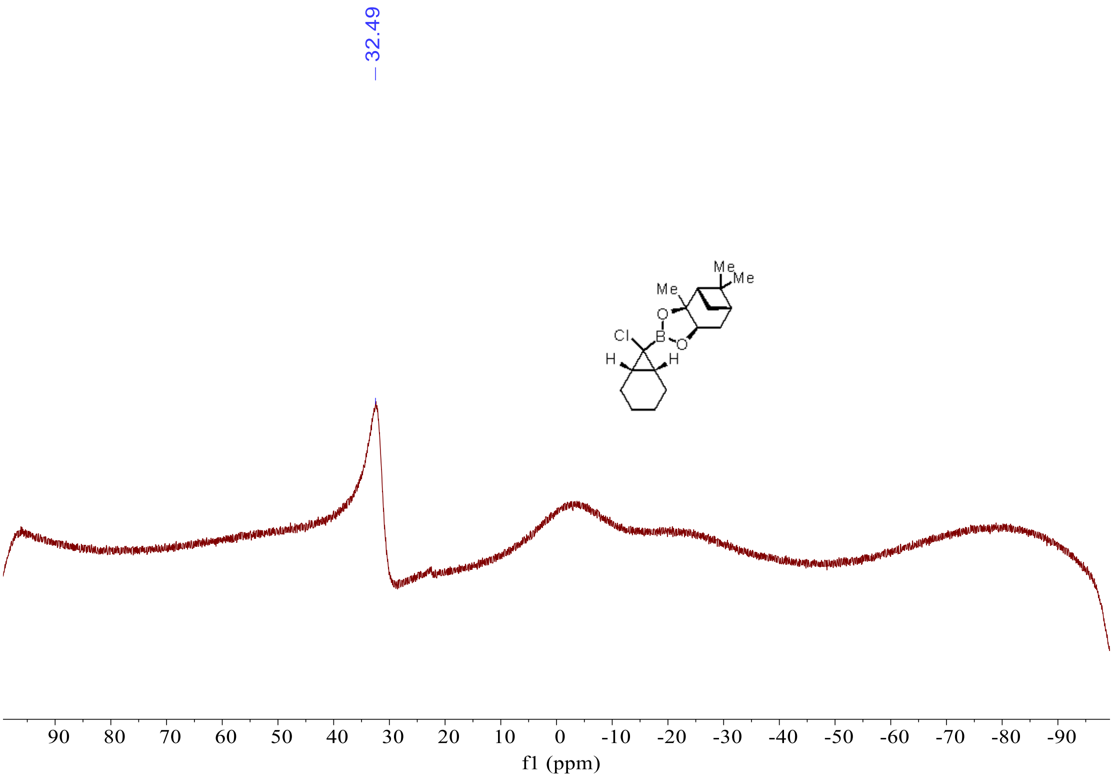

Supplementary Figure 204. ^{11}B NMR (128 MHz, $CDCl_3$) spectra for compound **47**

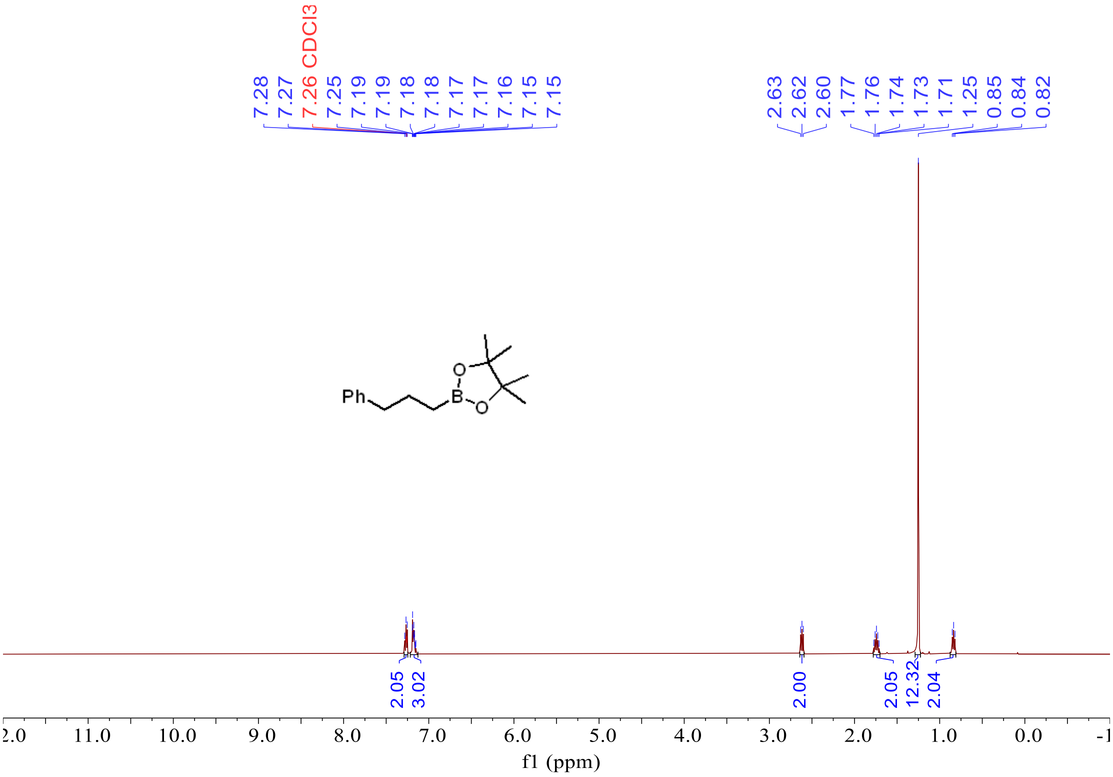

Supplementary Figure 205. ^{1}H NMR (500 MHz, $CDCl_3$) spectra for compound **48**

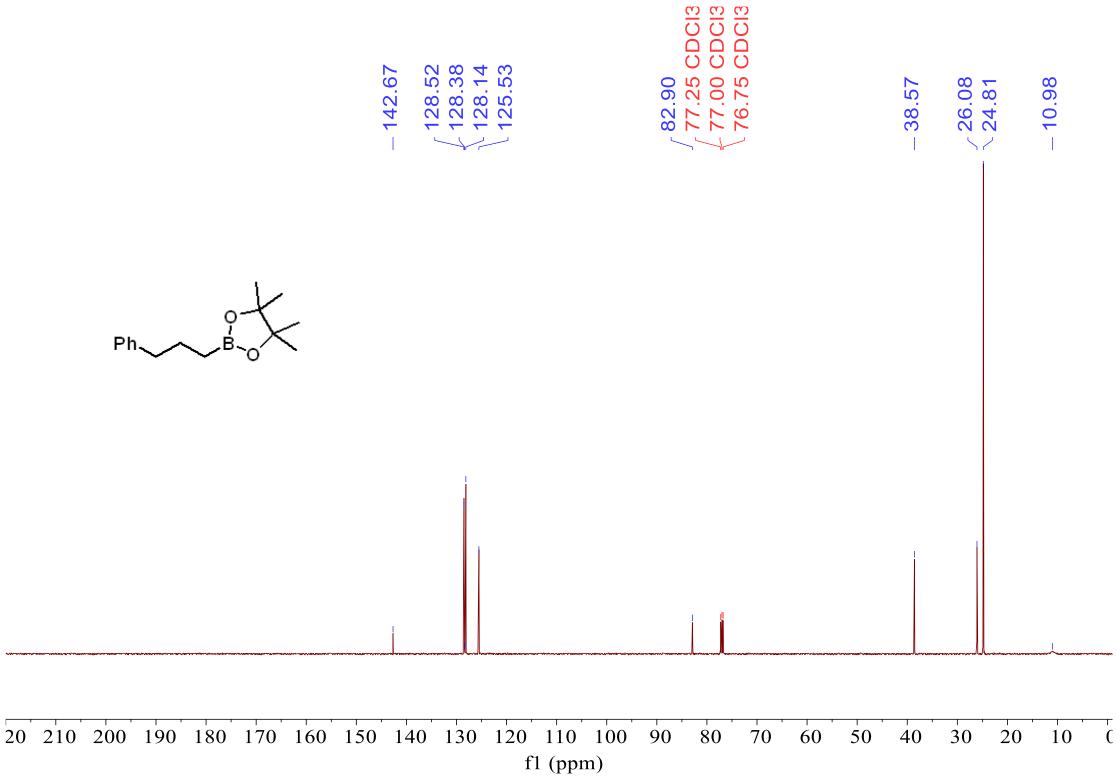

Supplementary Figure 206. ^{13}C NMR (126 MHz, $CDCl_3$) spectra for compound **48**

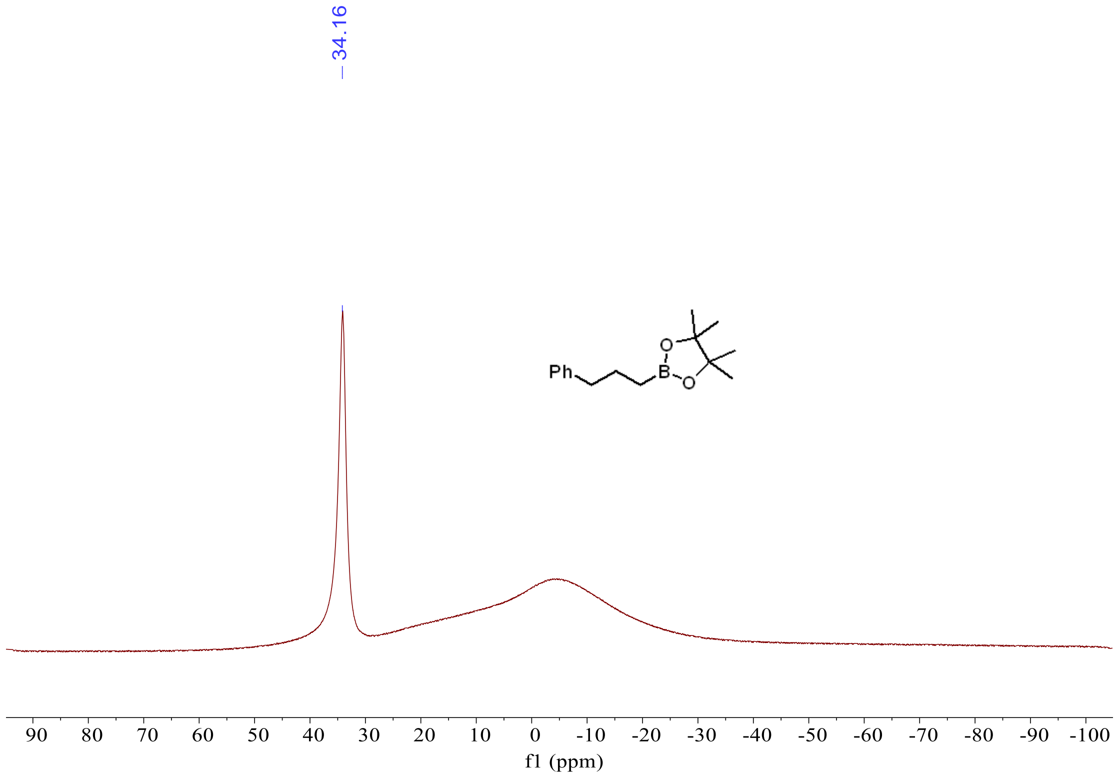

Supplementary Figure 207. ^{11}B NMR (160 MHz, $CDCl_3$) spectra for compound **48**

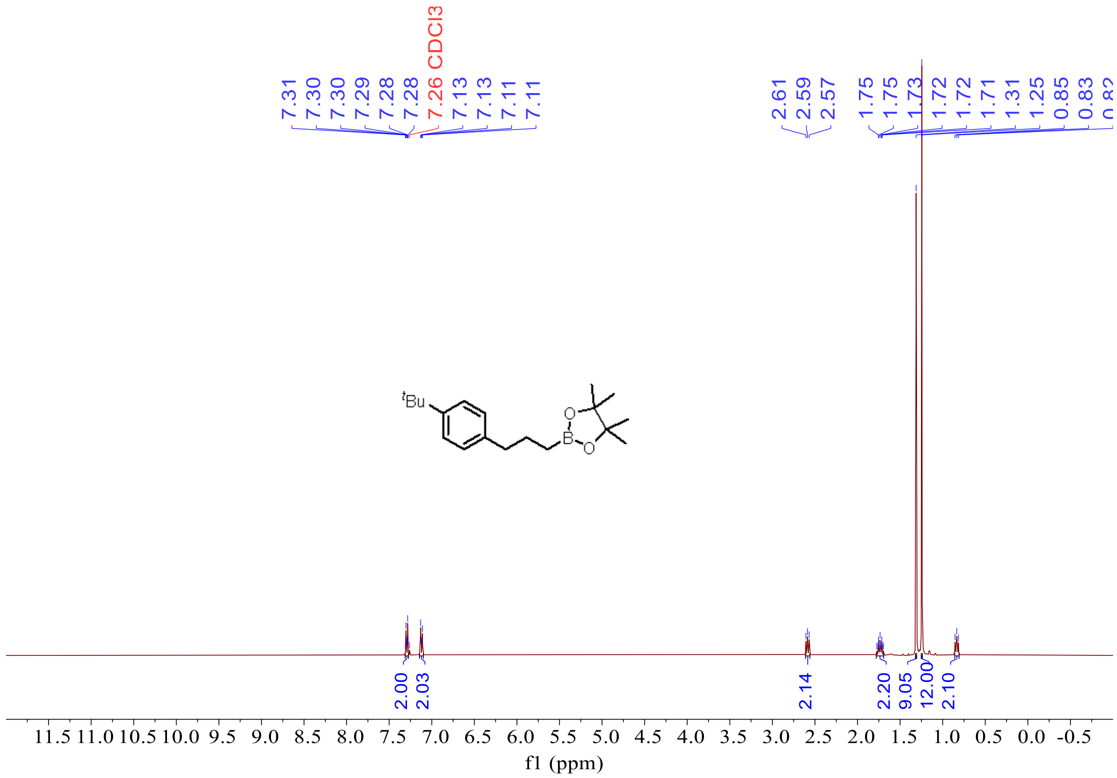

Supplementary Figure 208. ^{1}H NMR (400 MHz, $CDCl_3$) spectra for compound **49**

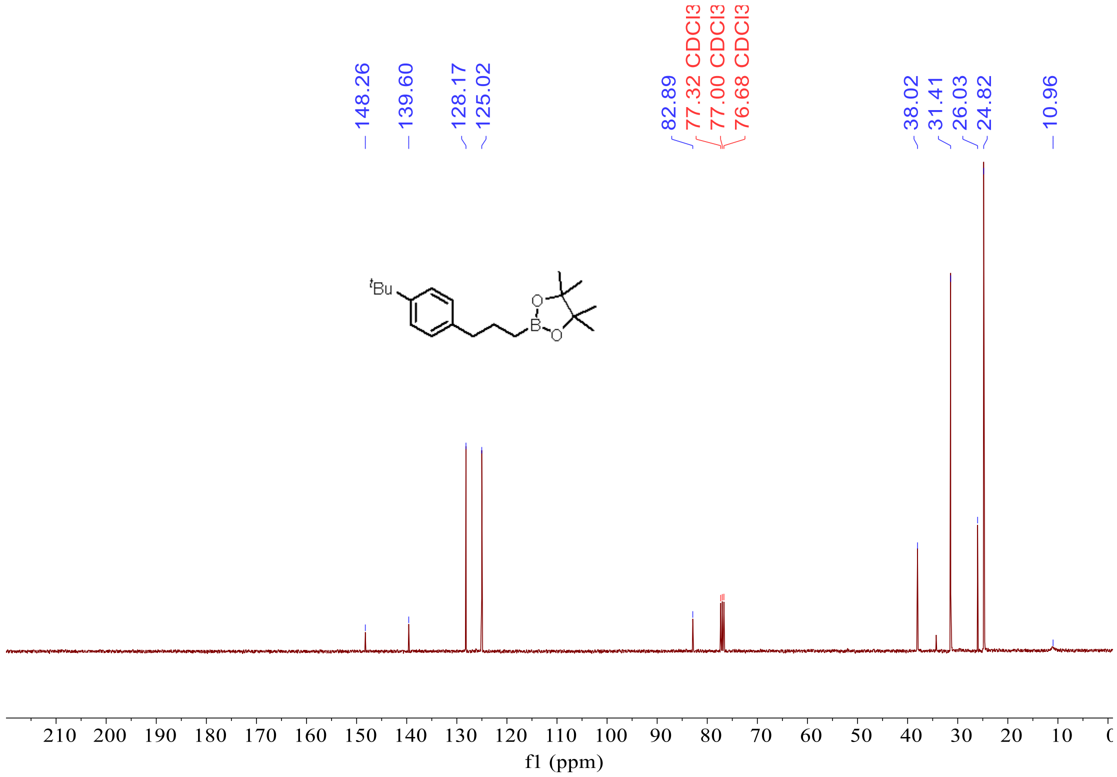

Supplementary Figure 209. ^{13}C NMR (101 MHz, $CDCl_3$) spectra for compound **49**

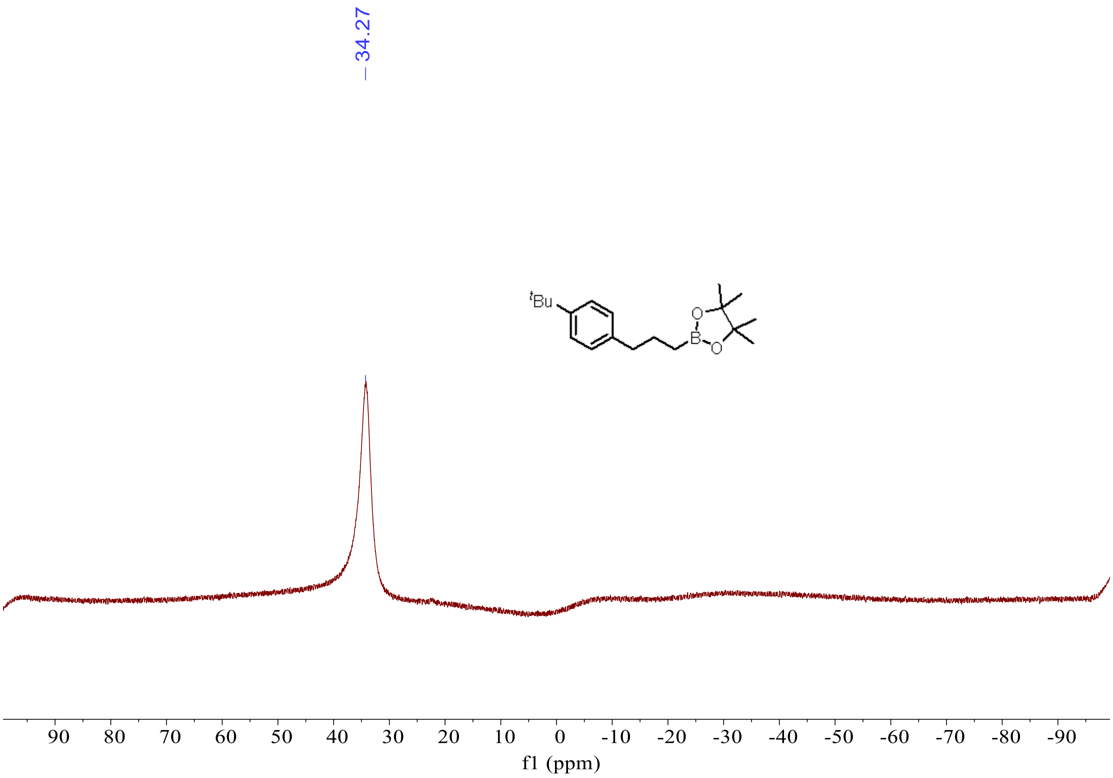

Supplementary Figure 210. ^{11}B NMR (128 MHz, $CDCl_3$) spectra for compound **49**

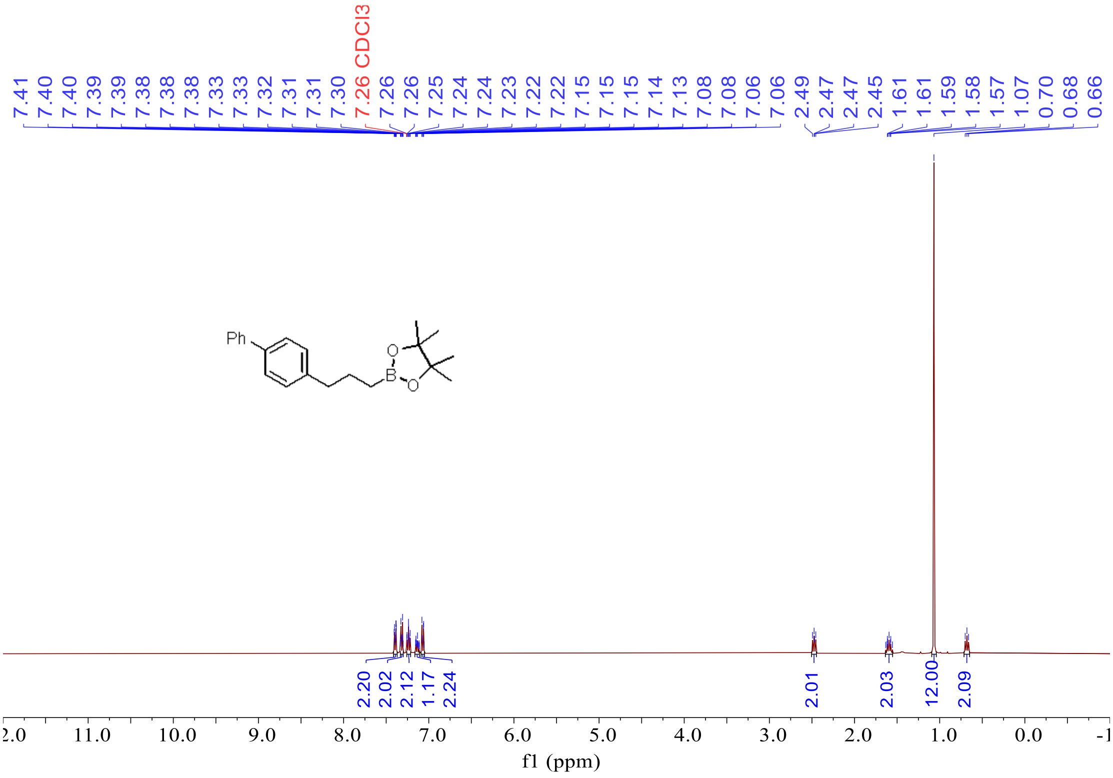

Supplementary Figure 211. ^{1}H NMR (400 MHz, $CDCl_3$) spectra for compound **50**

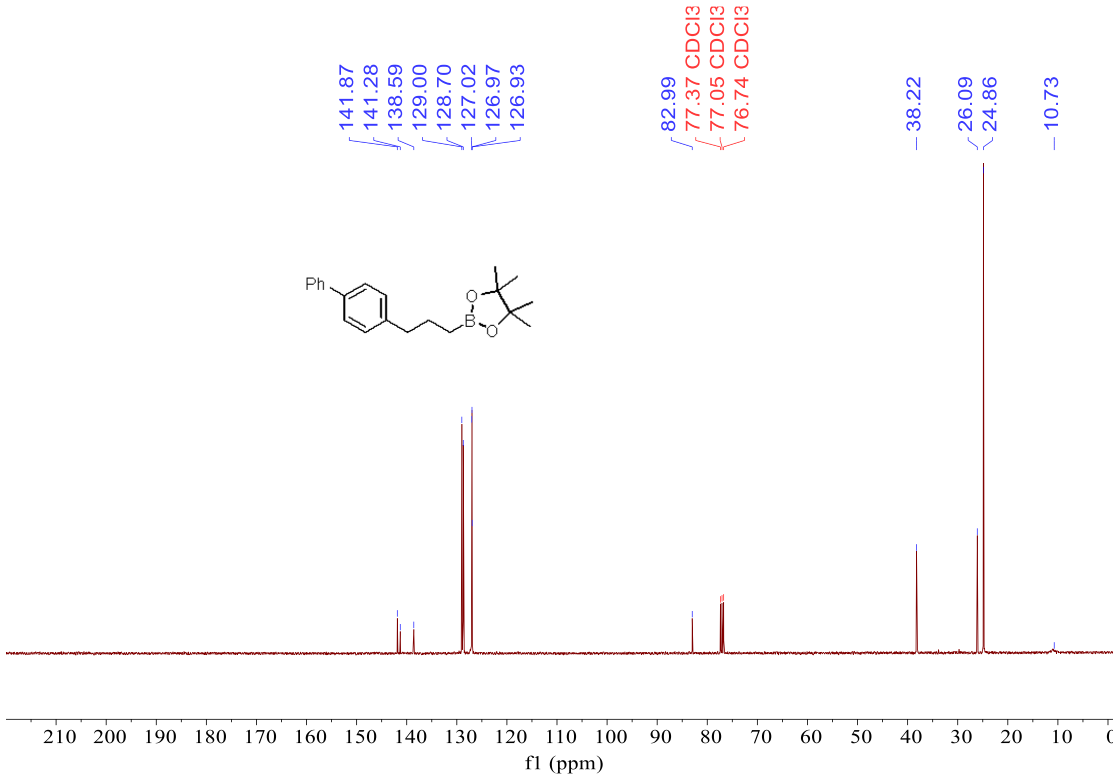

Supplementary Figure 212. ^{13}C NMR (101 MHz, $CDCl_3$) spectra for compound **50**

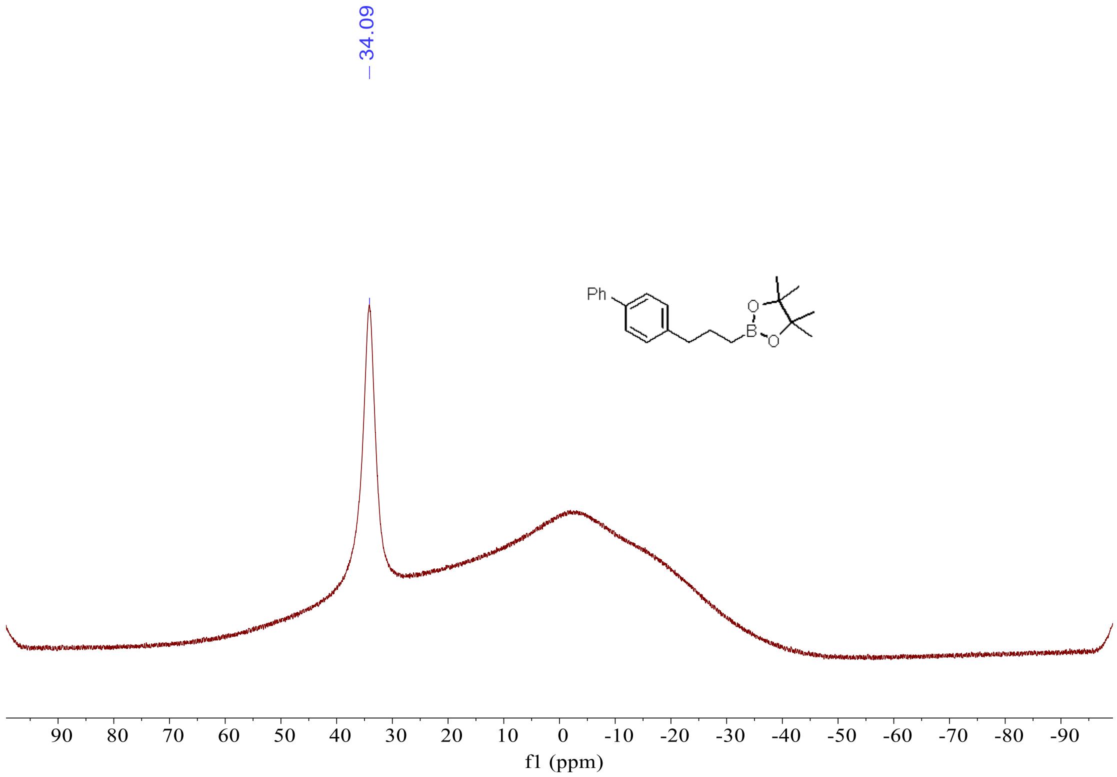

Supplementary Figure 213. ^{11}B NMR (128 MHz, $CDCl_3$) spectra for compound **50**

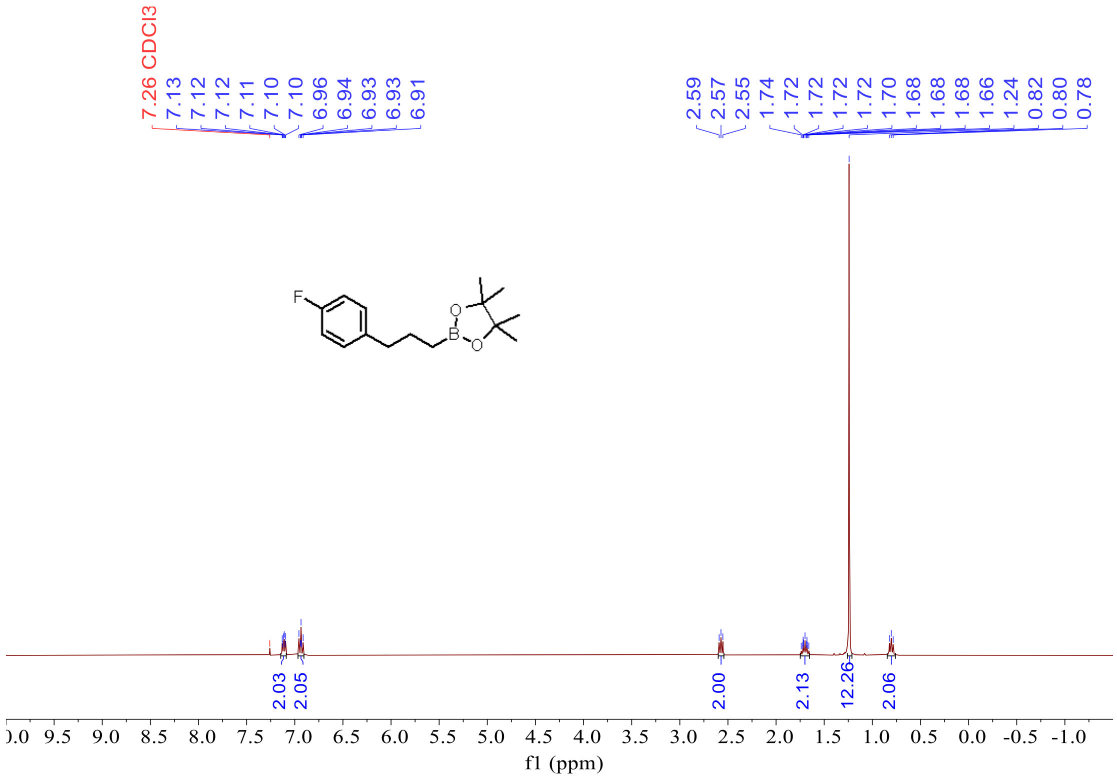

Supplementary Figure 214. ^{1}H NMR (400 MHz, $CDCl_3$) spectra for compound **51**

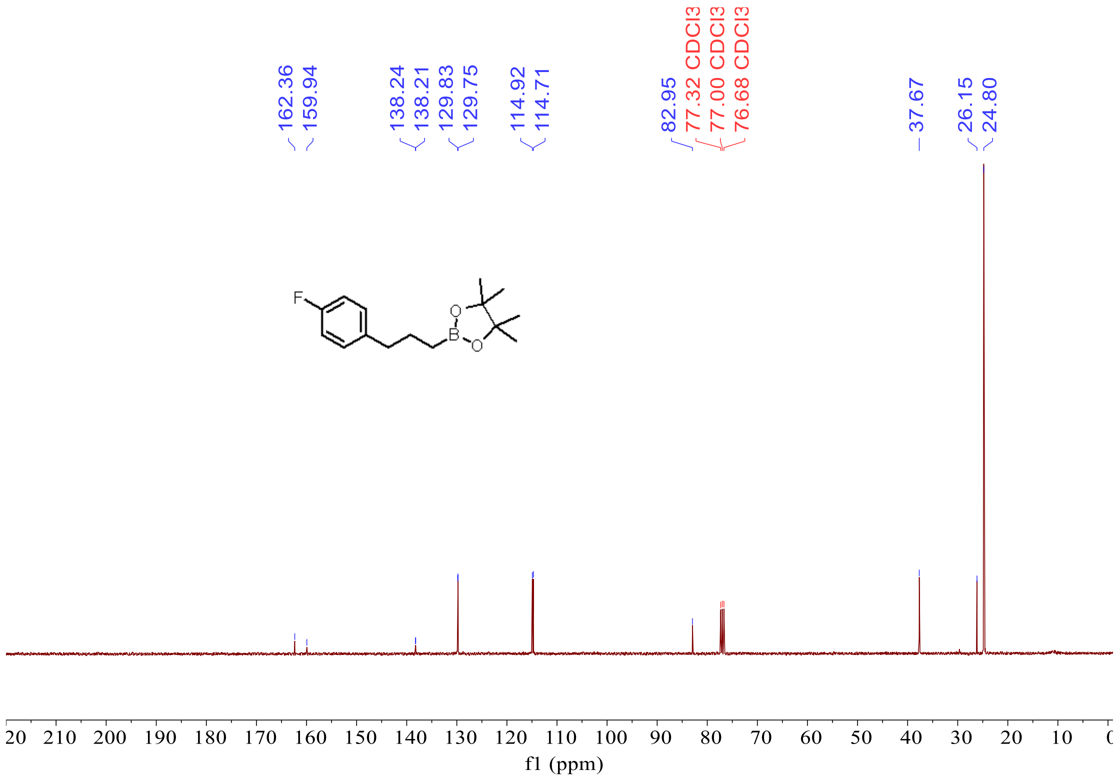

Supplementary Figure 215. ^{13}C NMR (101 MHz, $CDCl_3$) spectra for compound **51**

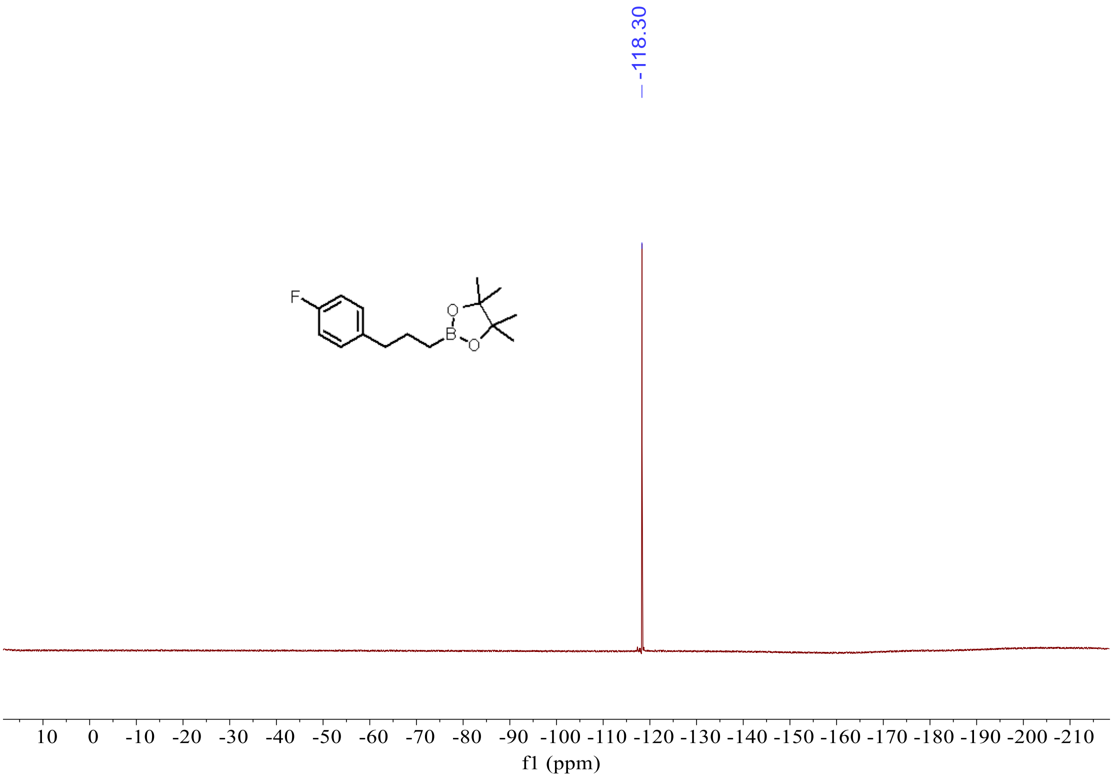

Supplementary Figure 216. ^{19}F NMR (376 MHz, $CDCl_3$) spectra for compound **51**

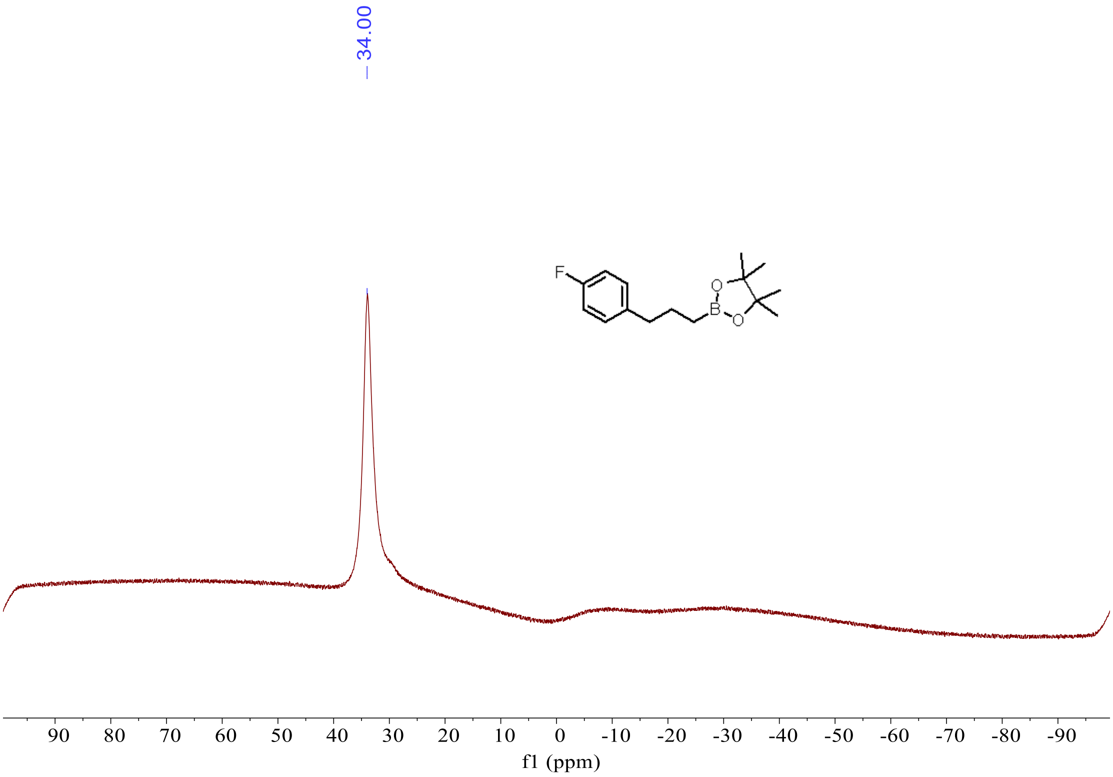

Supplementary Figure 217. ^{11}B NMR (128 MHz, $CDCl_3$) spectra for compound **51**

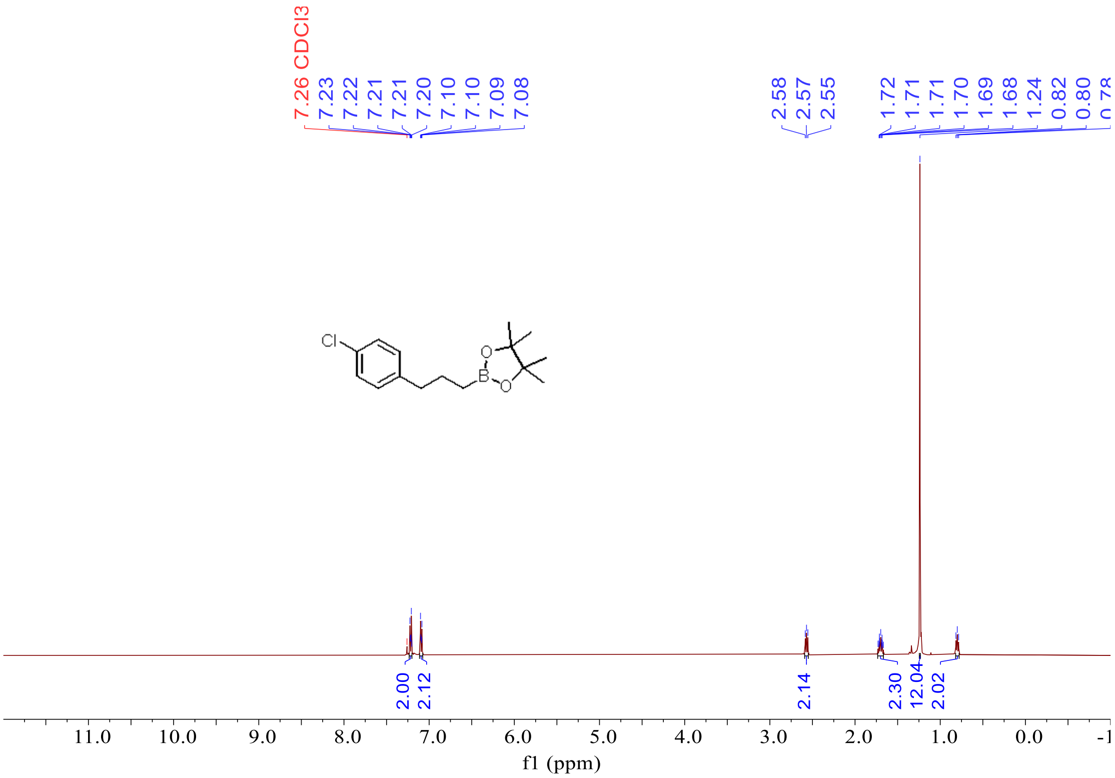

Supplementary Figure 218. ^{1}H NMR (500 MHz, $CDCl_3$) spectra for compound **52**

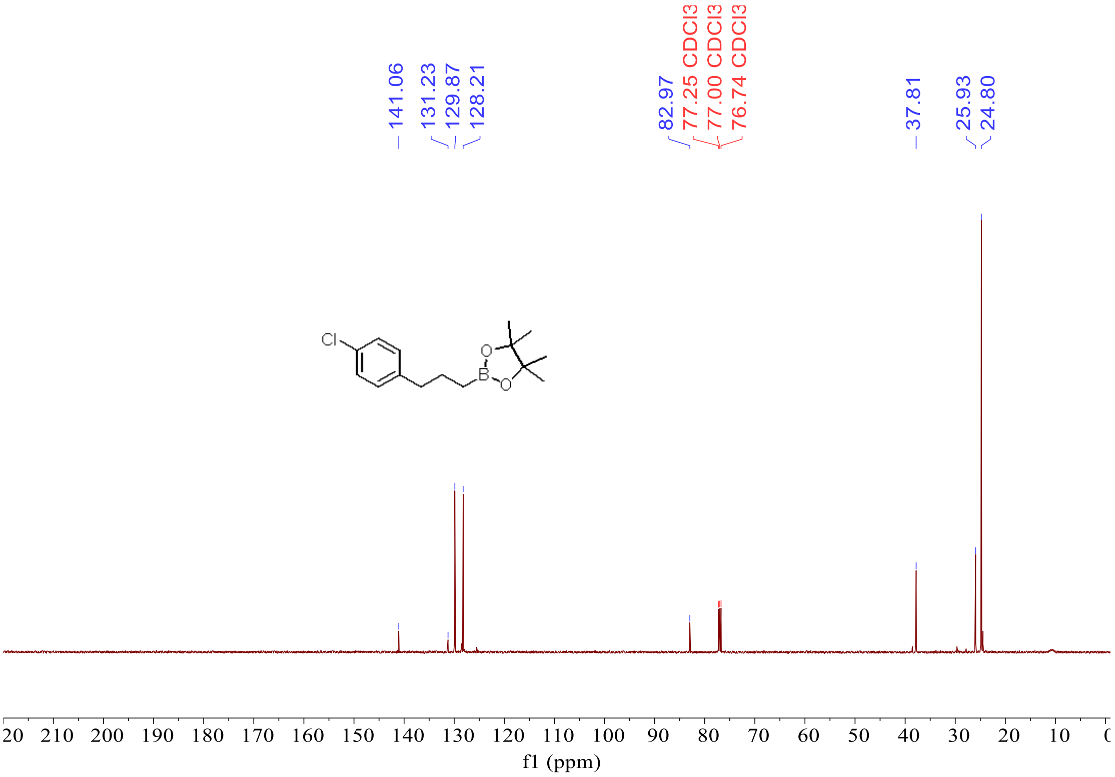

Supplementary Figure 219. ^{13}C NMR (126 MHz, $CDCl_3$) spectra for compound **52**

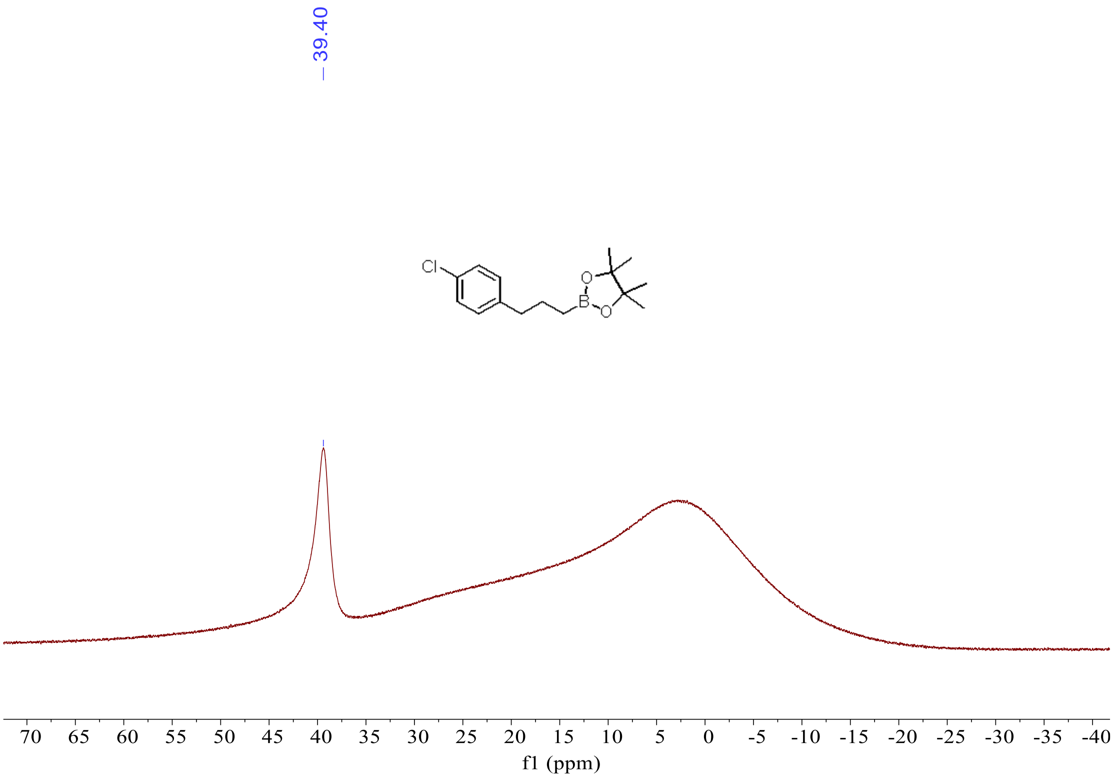

Supplementary Figure 220. ^{11}B NMR (160 MHz, $CDCl_3$) spectra for compound **52**

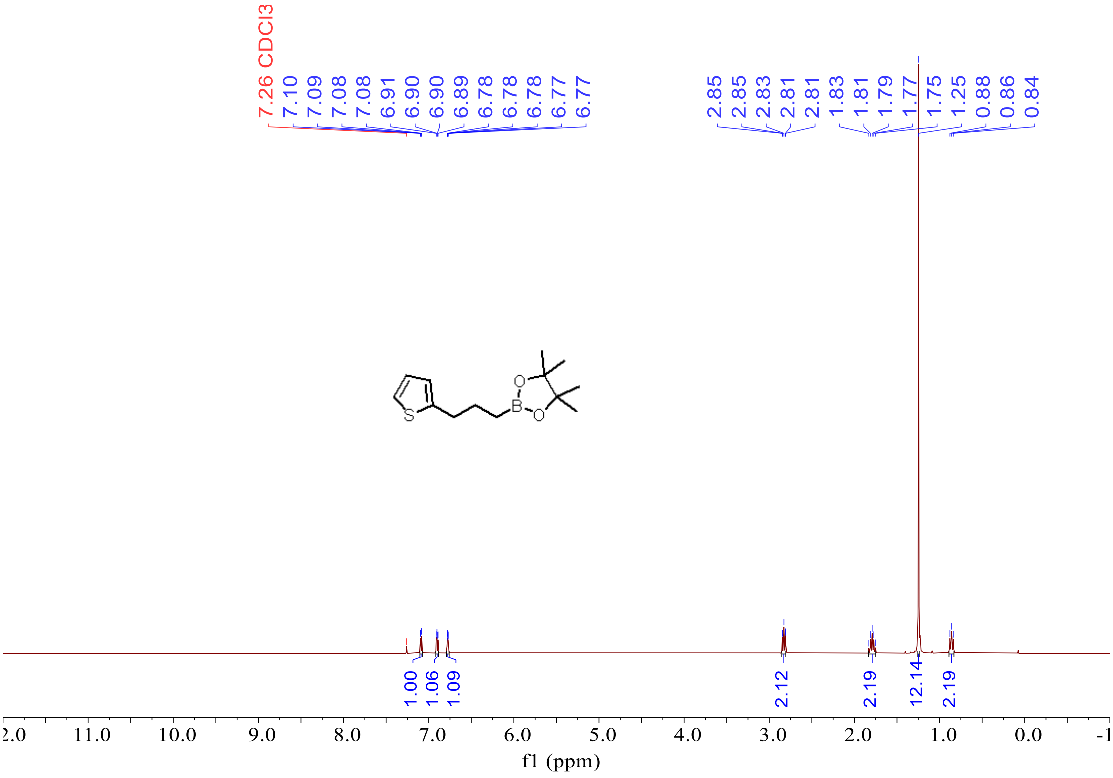

Supplementary Figure 221. ^{1}H NMR (400 MHz, $CDCl_3$) spectra for compound **53**

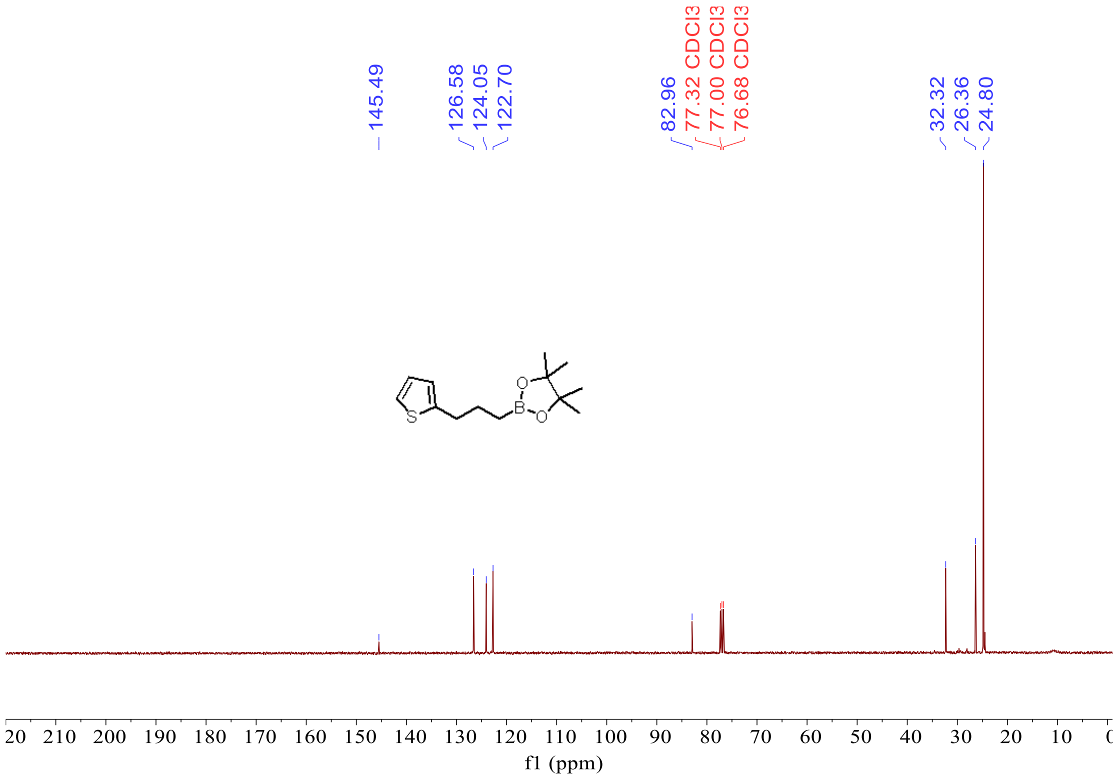

Supplementary Figure 222. ^{13}C NMR (101 MHz, $CDCl_3$) spectra for compound **53**

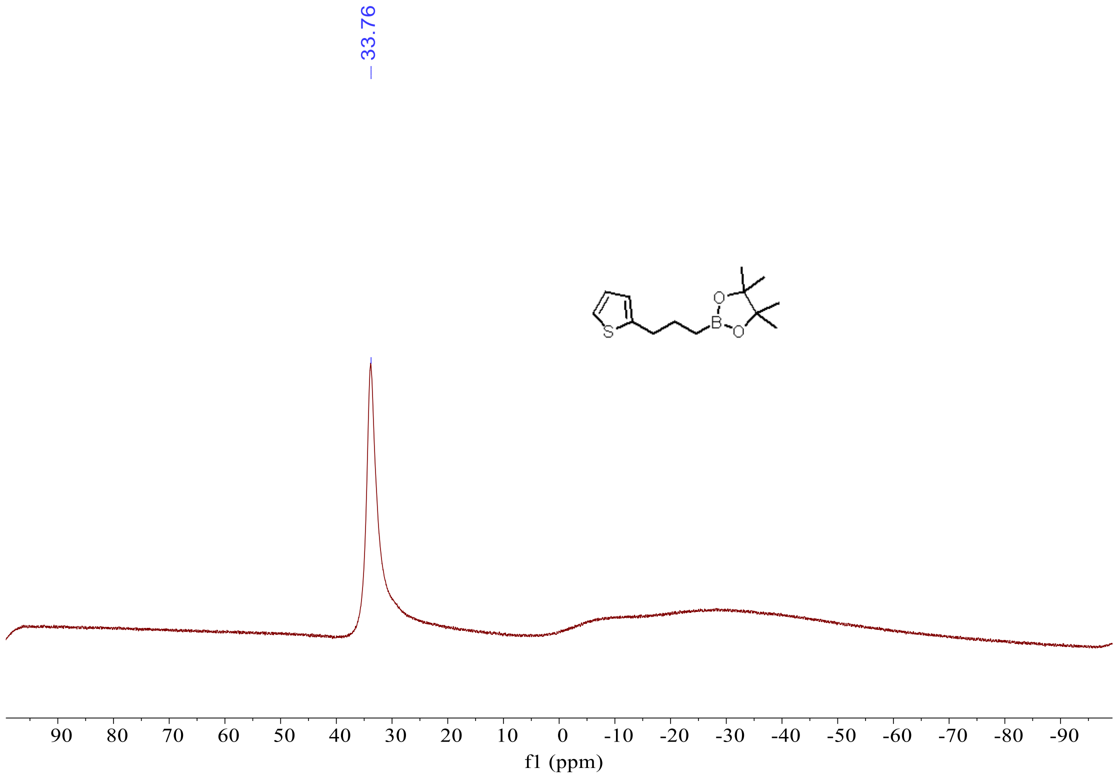

Supplementary Figure 223. ^{11}B NMR (128 MHz, $CDCl_3$) spectra for compound **53**

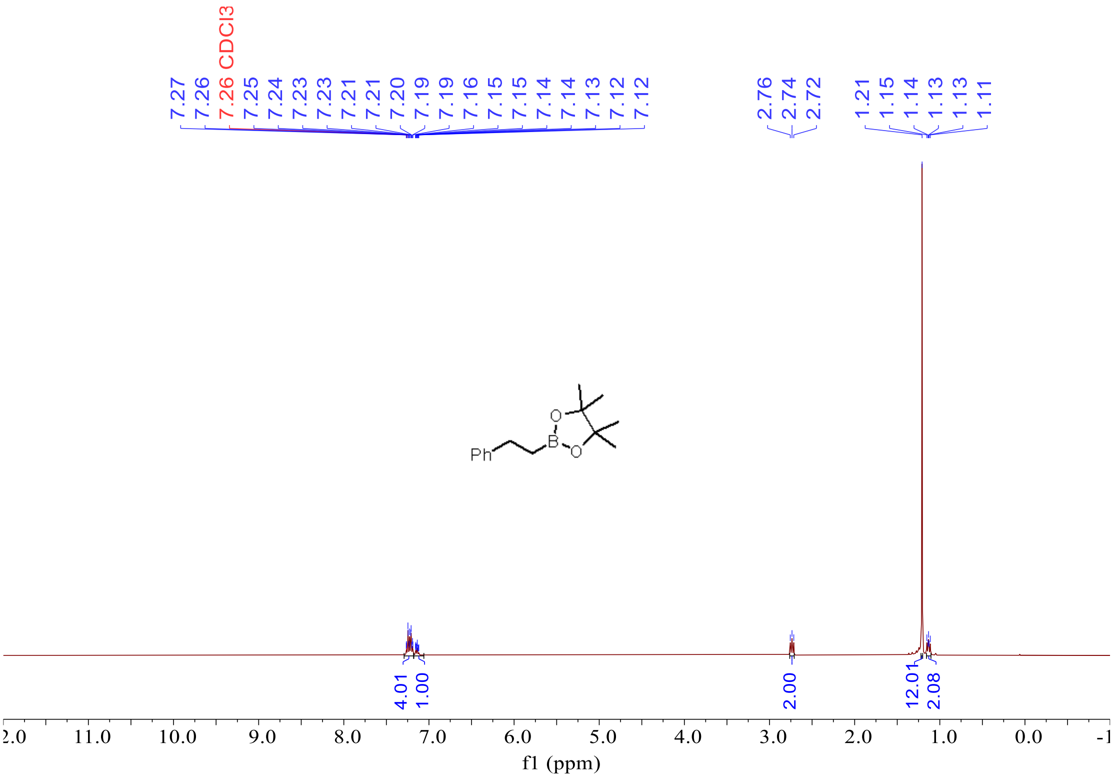

Supplementary Figure 224. ^{1}H NMR (400 MHz, $CDCl_3$) spectra for compound **54**

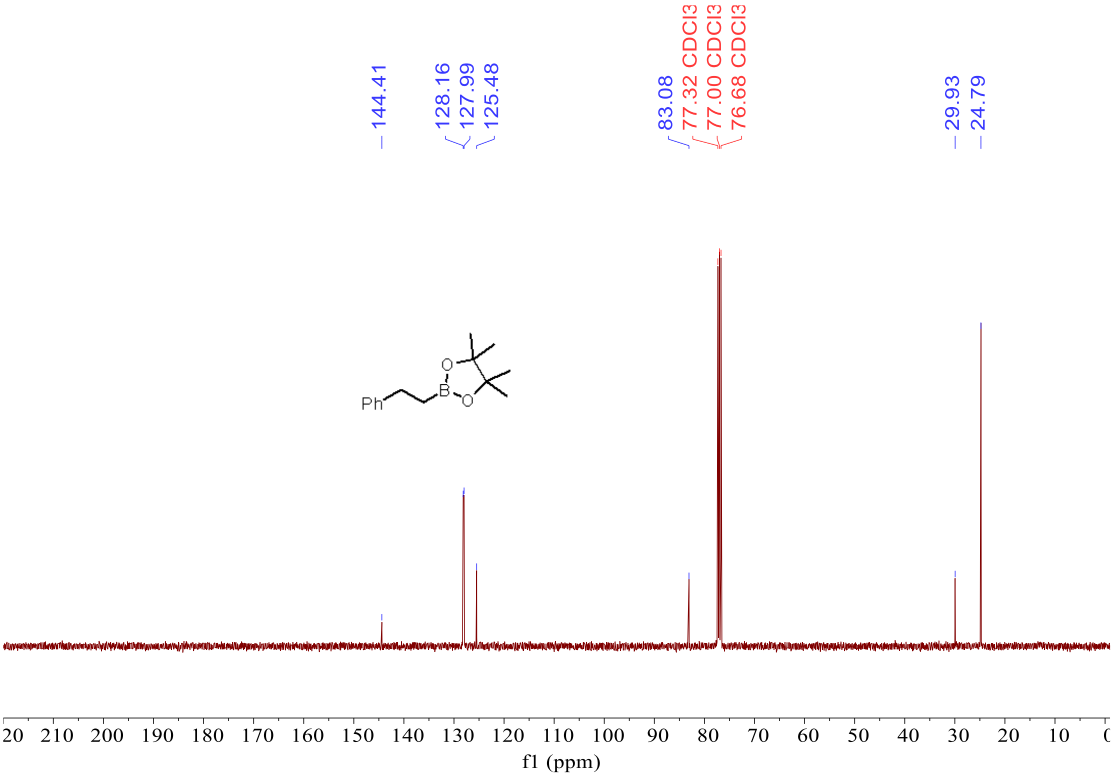

Supplementary Figure 225. ^{13}C NMR (101 MHz, $CDCl_3$) spectra for compound **54**

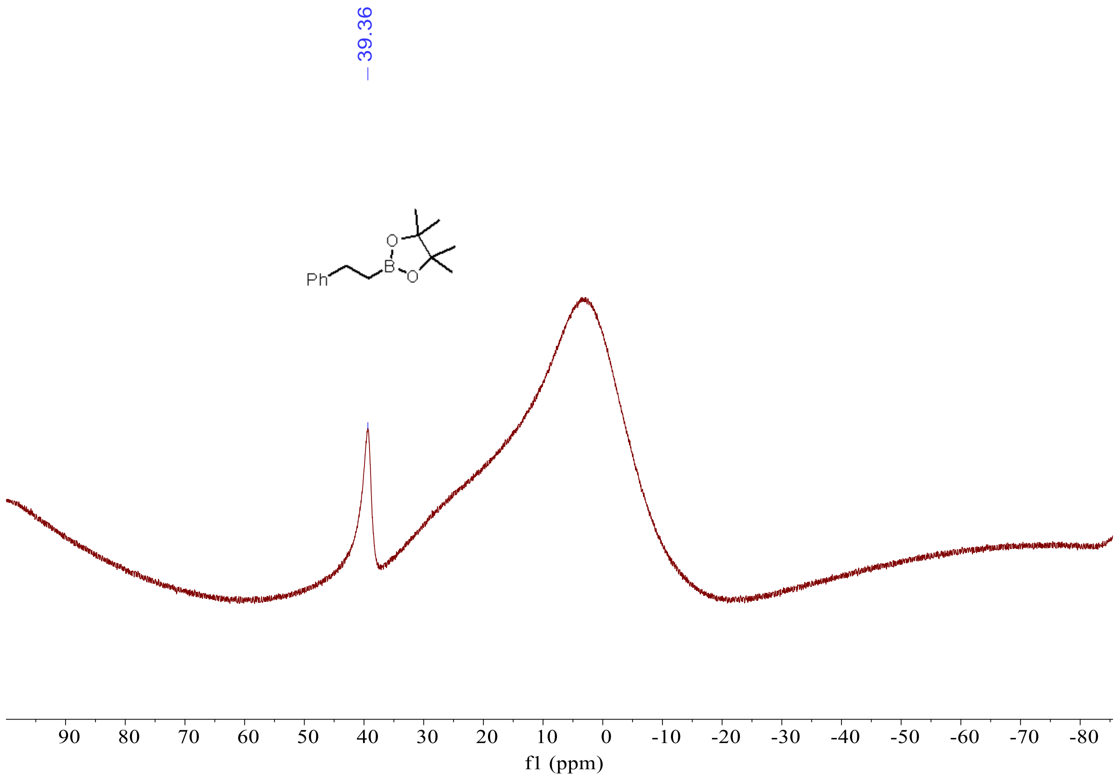

Supplementary Figure 226. ^{11}B NMR (160 MHz, $CDCl_3$) spectra for compound **54**

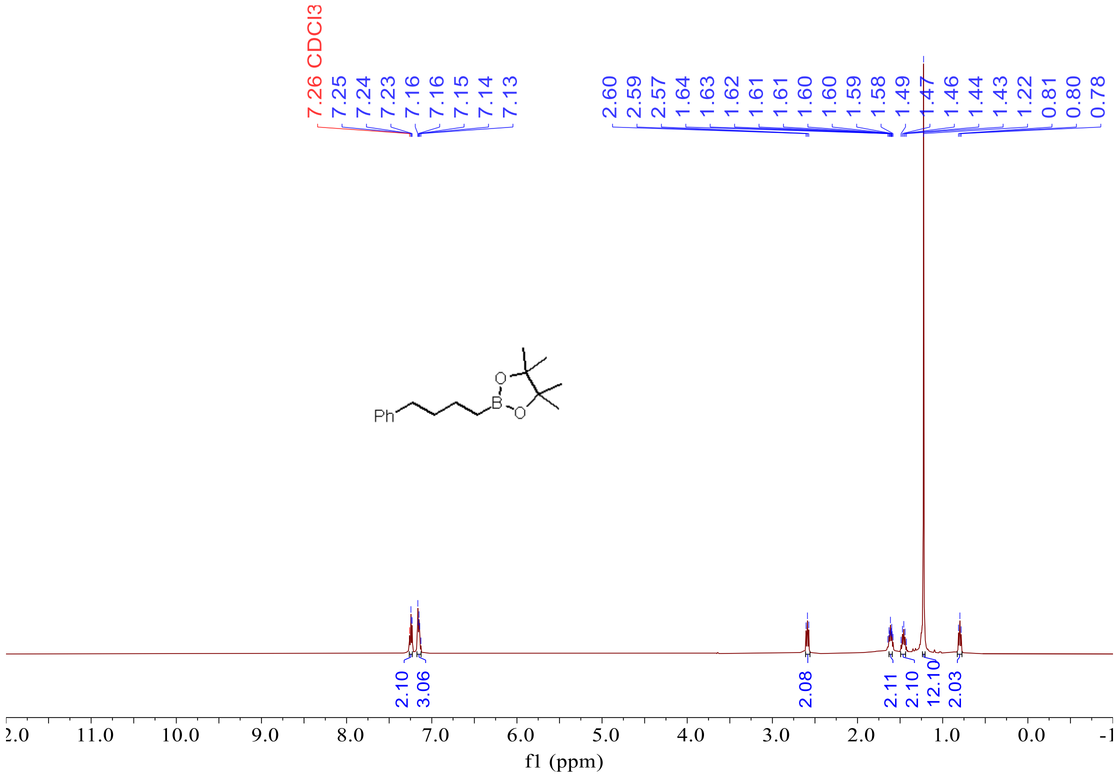

Supplementary Figure 227. ^{1}H NMR (500 MHz, $CDCl_3$) spectra for compound **55**

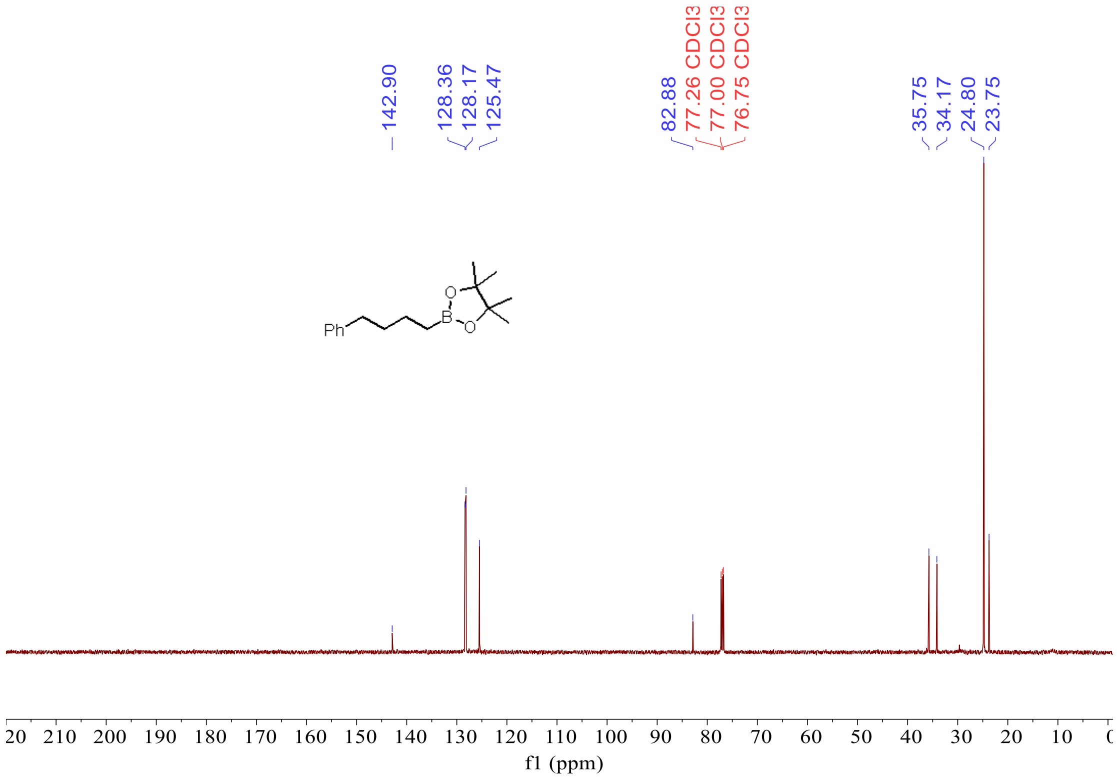

Supplementary Figure 228. ^{13}C NMR (126 MHz, $CDCl_3$) spectra for compound **55**

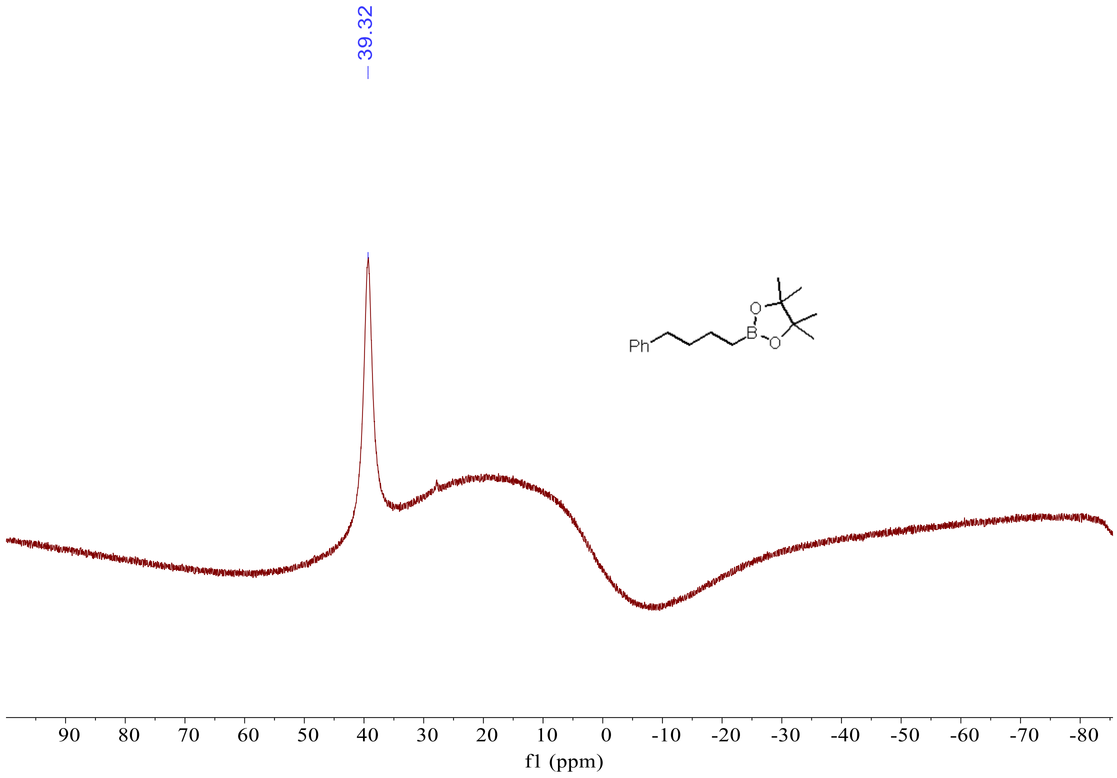

Supplementary Figure 229. ^{11}B NMR (160 MHz, $CDCl_3$) spectra for compound **55**

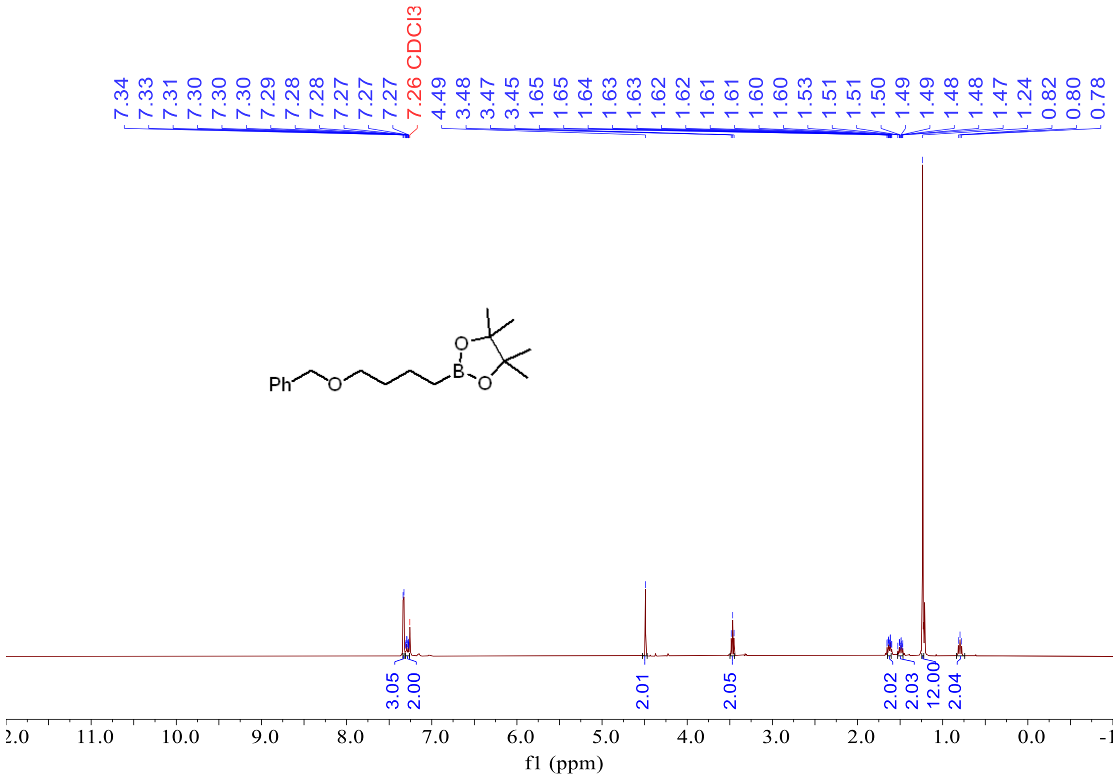

Supplementary Figure 230. ^{1}H NMR (400 MHz, $CDCl_3$) spectra for compound **56**

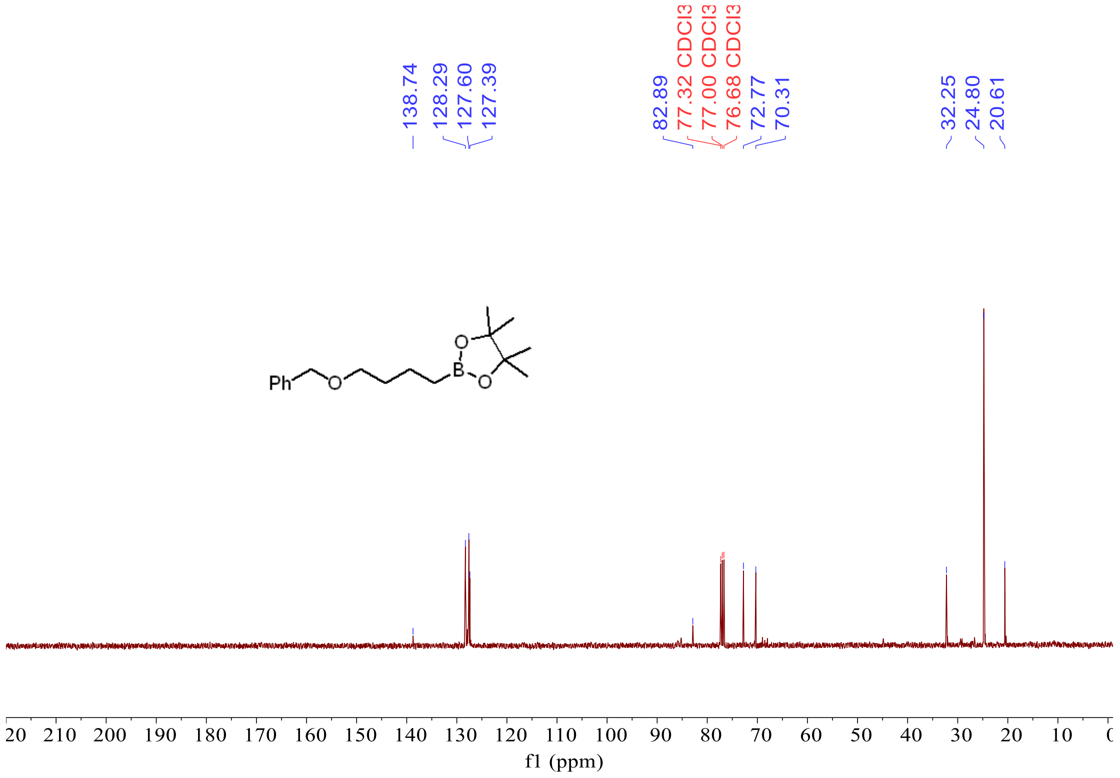

Supplementary Figure 231. ^{13}C NMR (101 MHz, $CDCl_3$) spectra for compound **56**

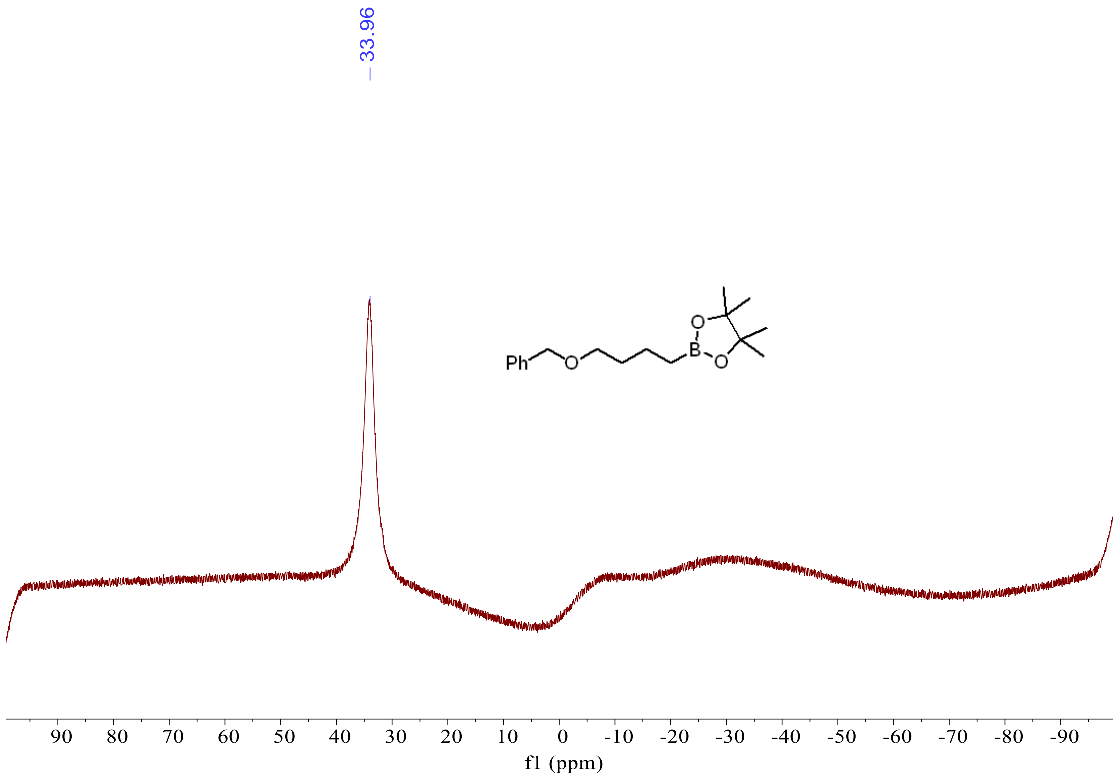

Supplementary Figure 232. ^{11}B NMR (128 MHz, $CDCl_3$) spectra for compound **56**

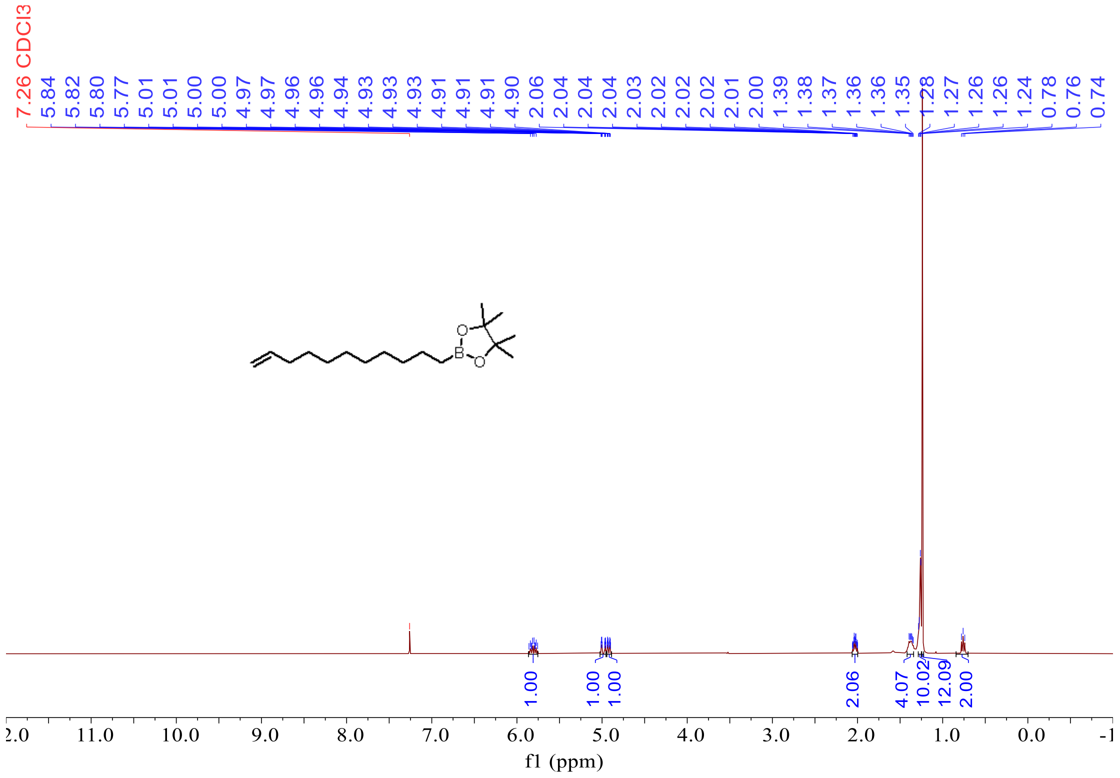

Supplementary Figure 233. ^{1}H NMR (400 MHz, $CDCl_3$) spectra for compound **57**

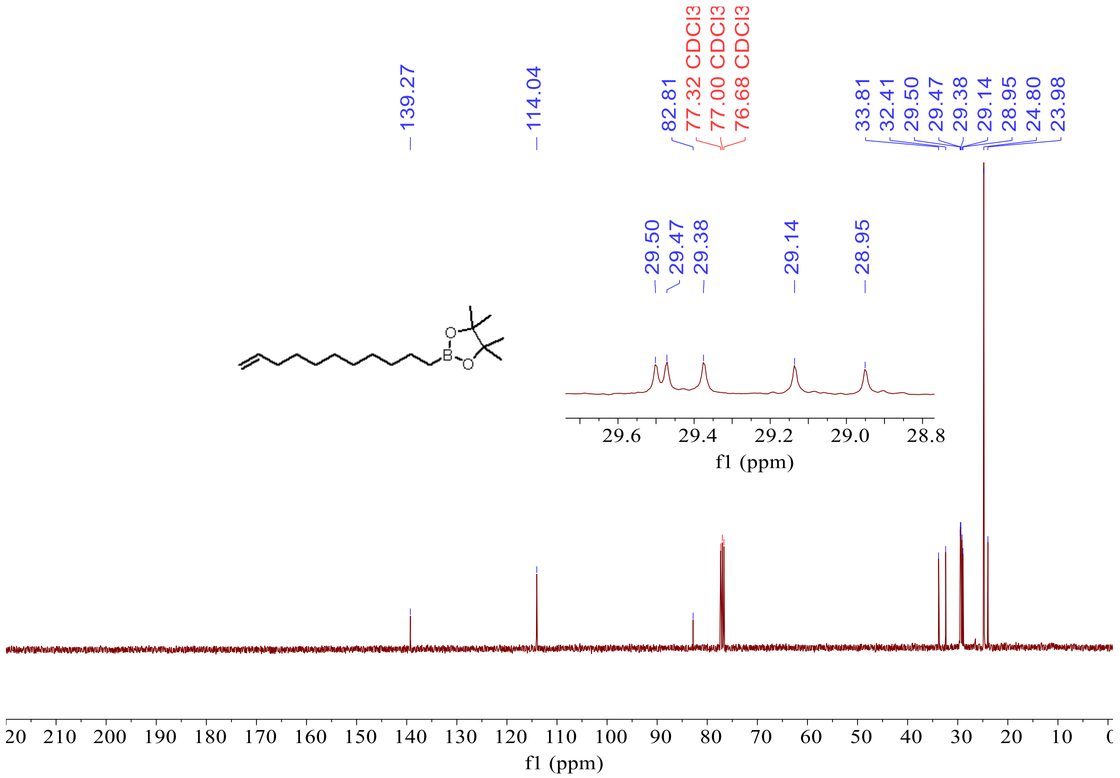

Supplementary Figure 234. ^{13}C NMR (101 MHz, $CDCl_3$) spectra for compound **57**

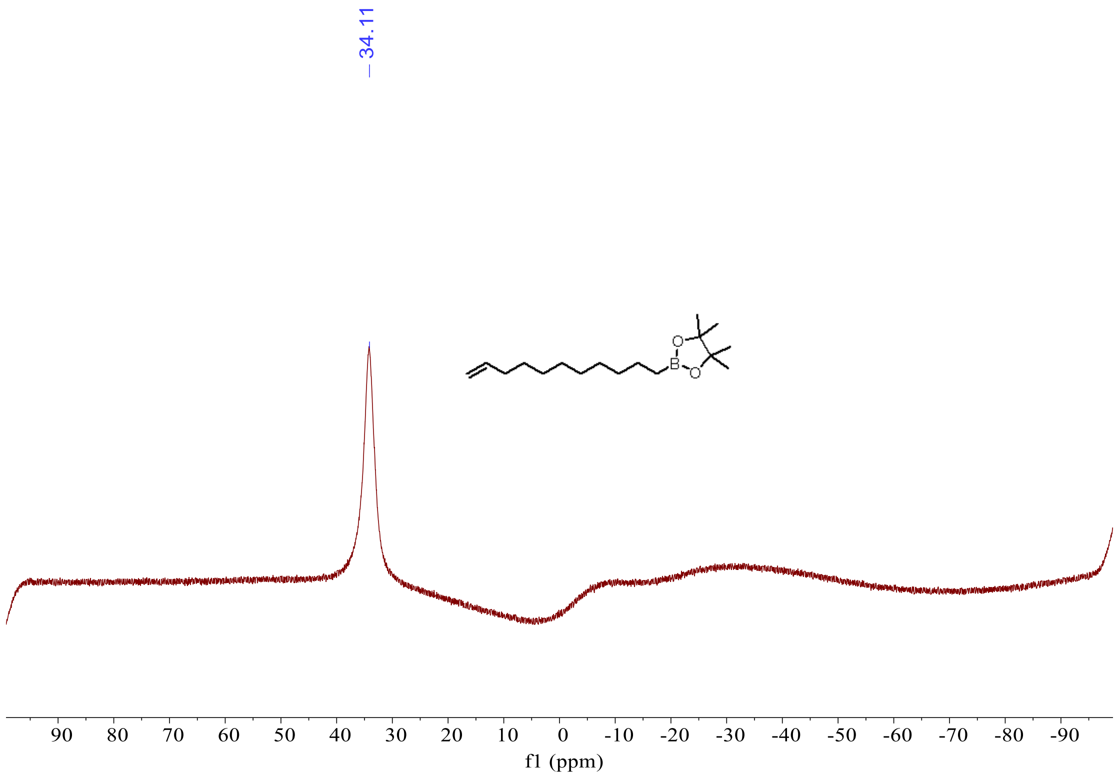

Supplementary Figure 235. ^{11}B NMR (128 MHz, $CDCl_3$) spectra for compound **57**

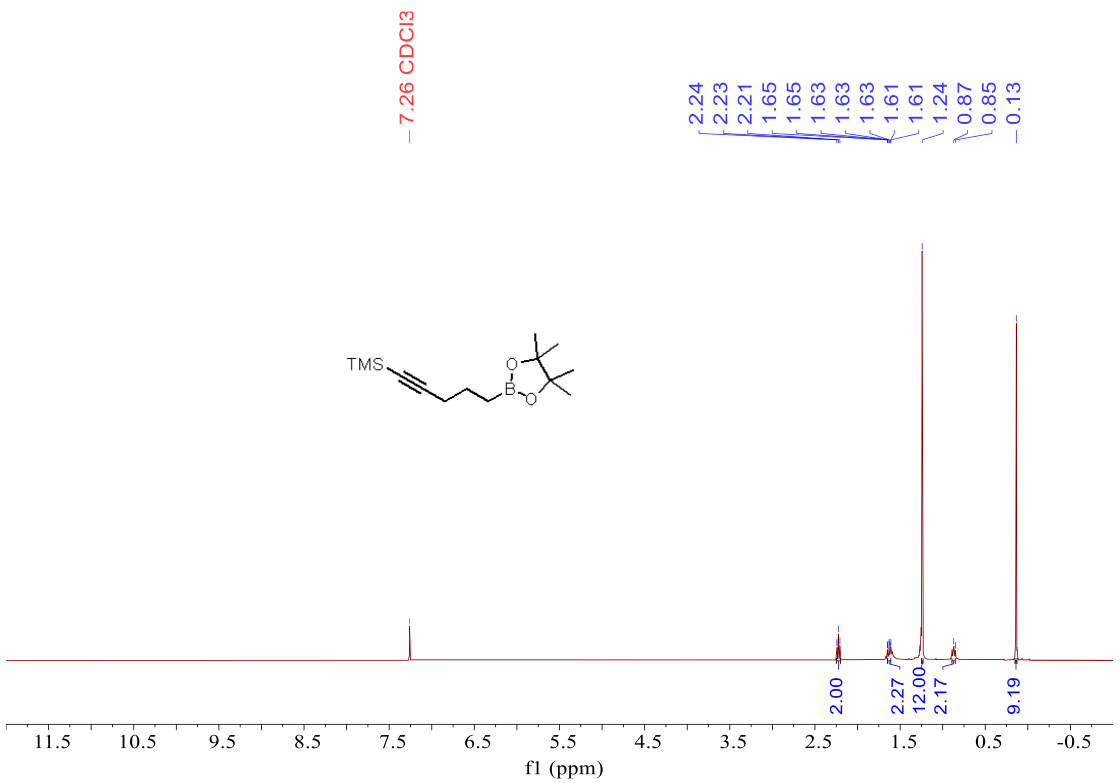

Supplementary Figure 236. ^{1}H NMR (400 MHz, $CDCl_3$) spectra for compound **58**

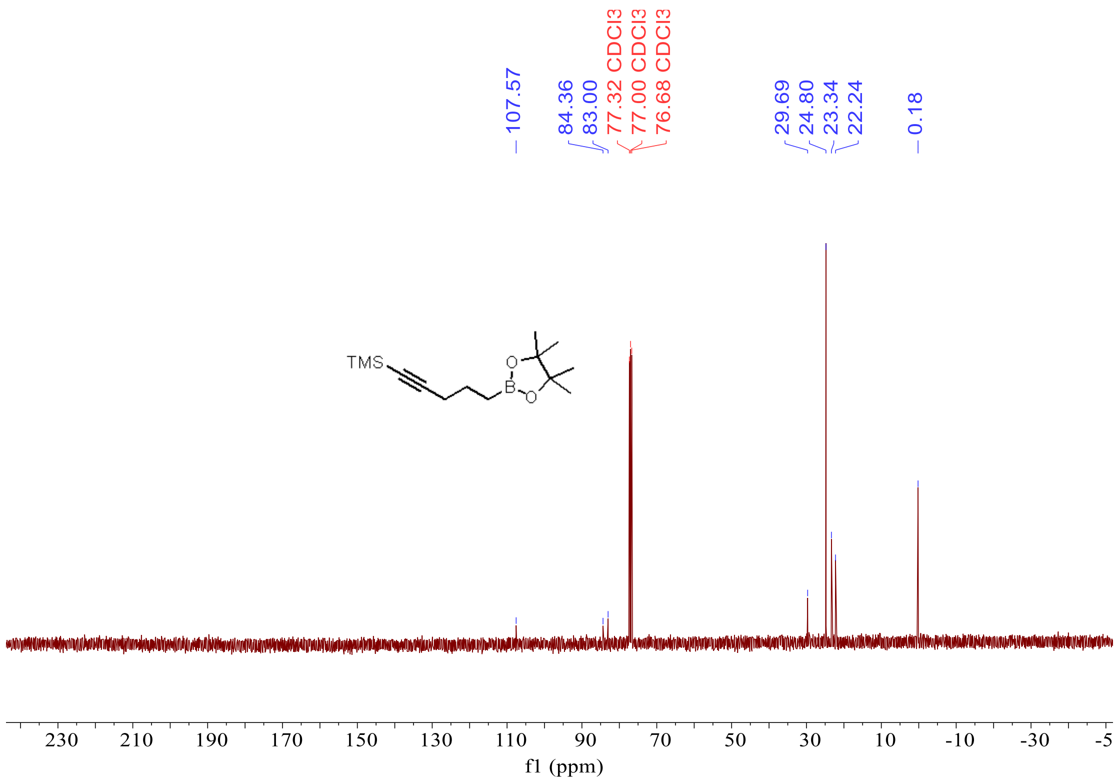

Supplementary Figure 237. ^{13}C NMR (101 MHz, $CDCl_3$) spectra for compound **58**

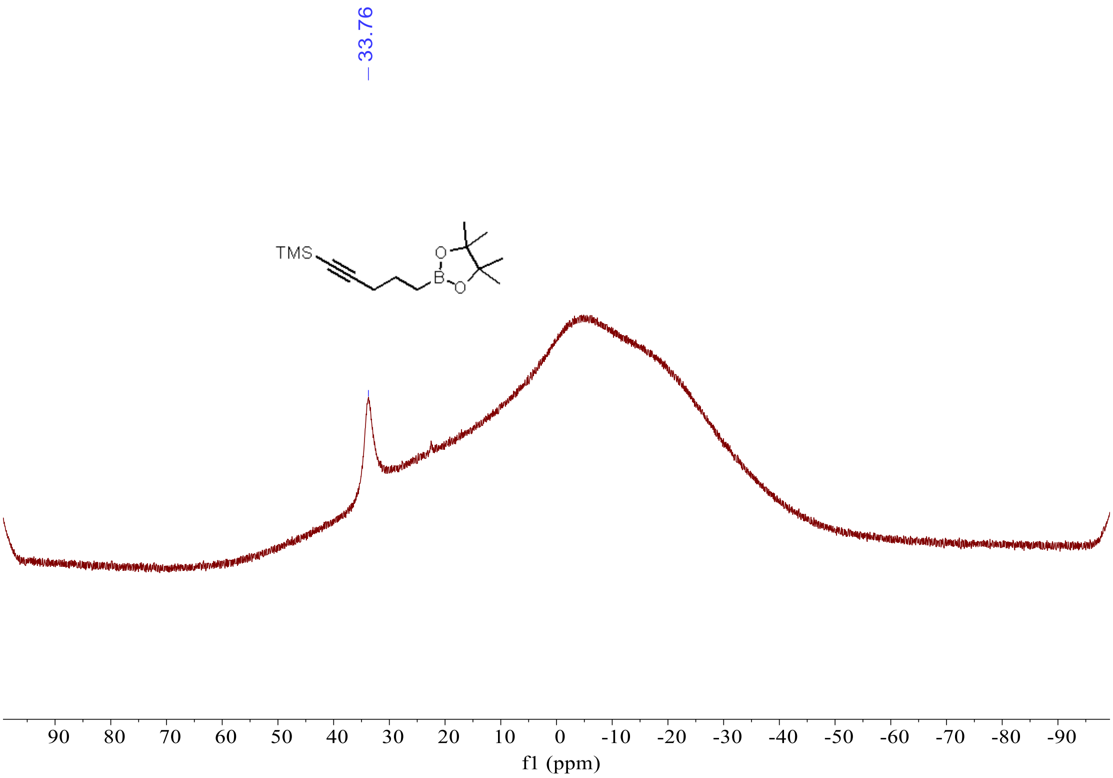

Supplementary Figure 238. ^{11}B NMR (128 MHz, $CDCl_3$) spectra for compound **58**

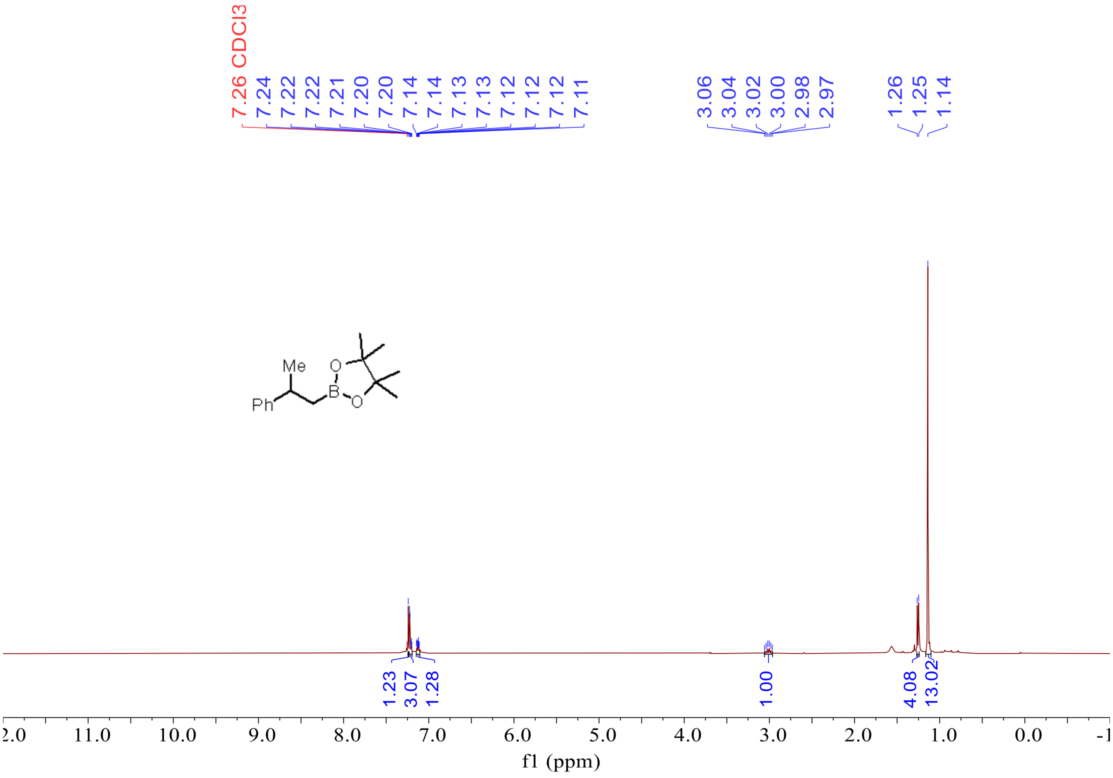

Supplementary Figure 239. ^{1}H NMR (400 MHz, $CDCl_3$) spectra for compound **59**

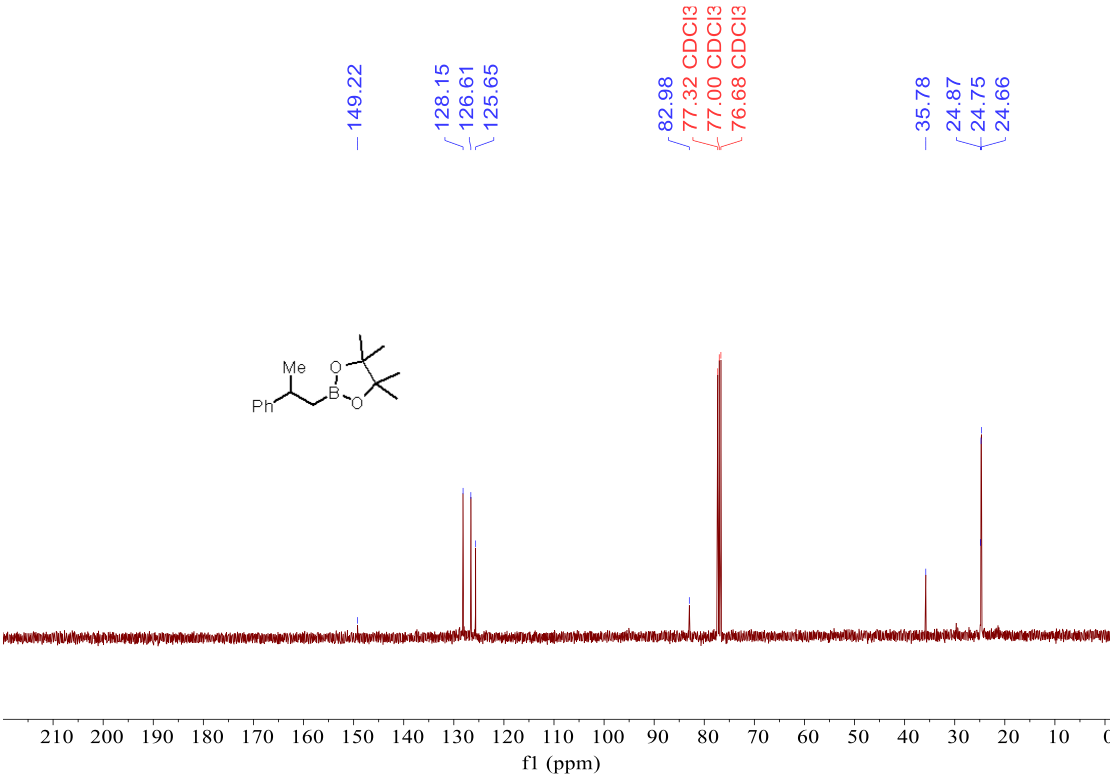

Supplementary Figure 240. ^{13}C NMR (101 MHz, $CDCl_3$) spectra for compound **59**

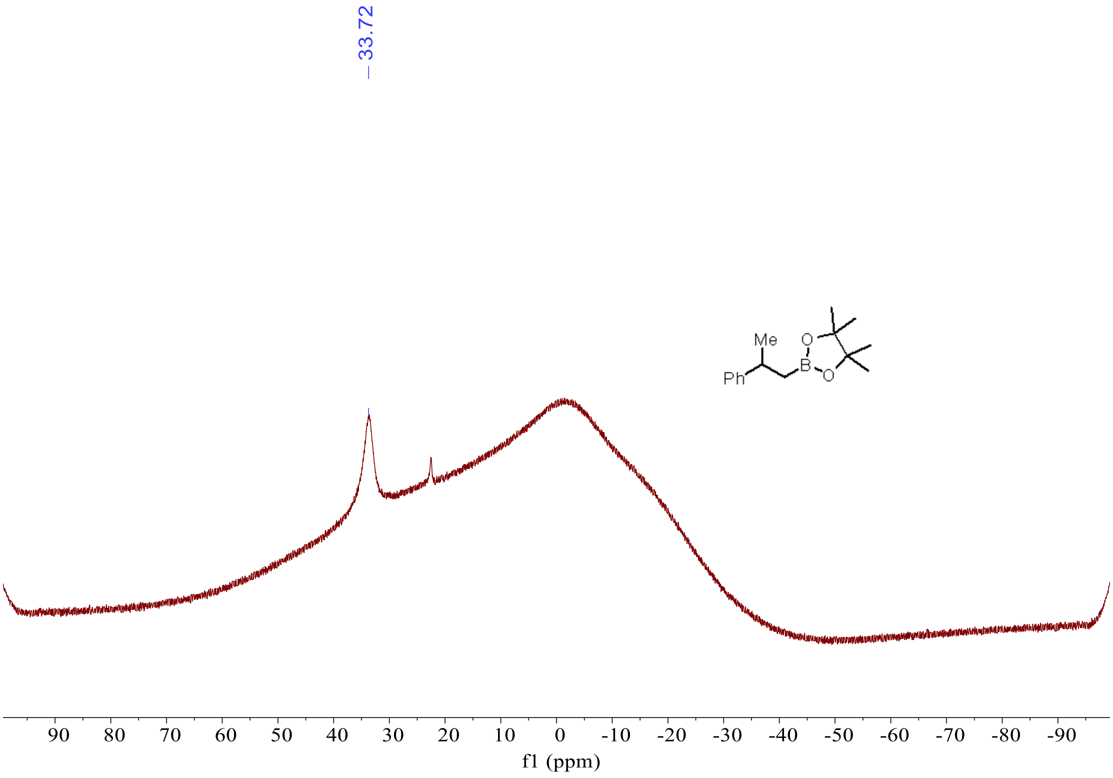

Supplementary Figure 241. ^{11}B NMR (128 MHz, $CDCl_3$) spectra for compound **59**

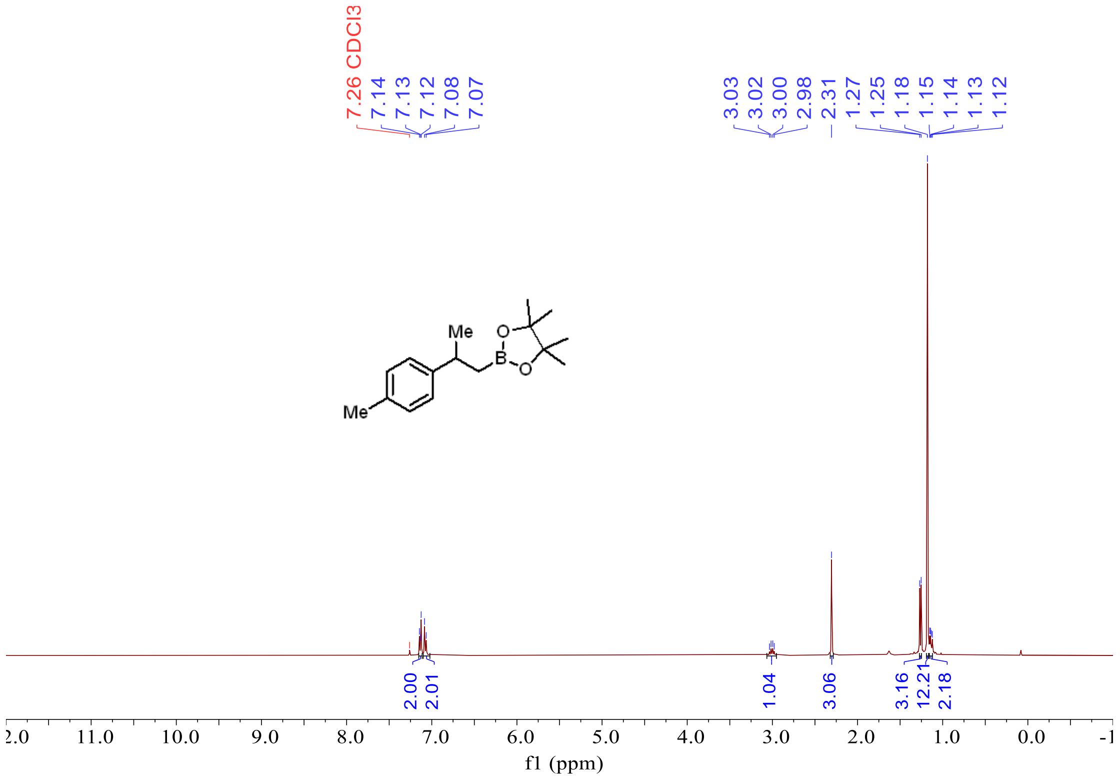

Supplementary Figure 242. ^{1}H NMR (400 MHz, $CDCl_3$) spectra for compound **60**

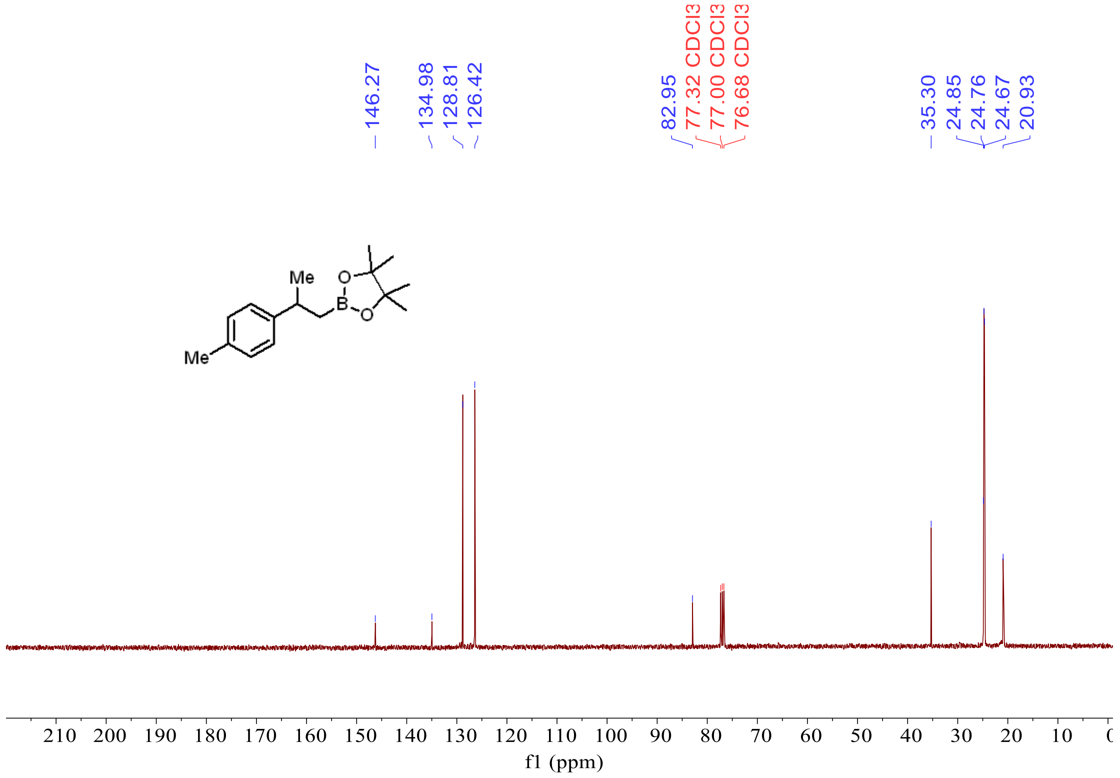

Supplementary Figure 243. ^{13}C NMR (101 MHz, $CDCl_3$) spectra for compound **60**

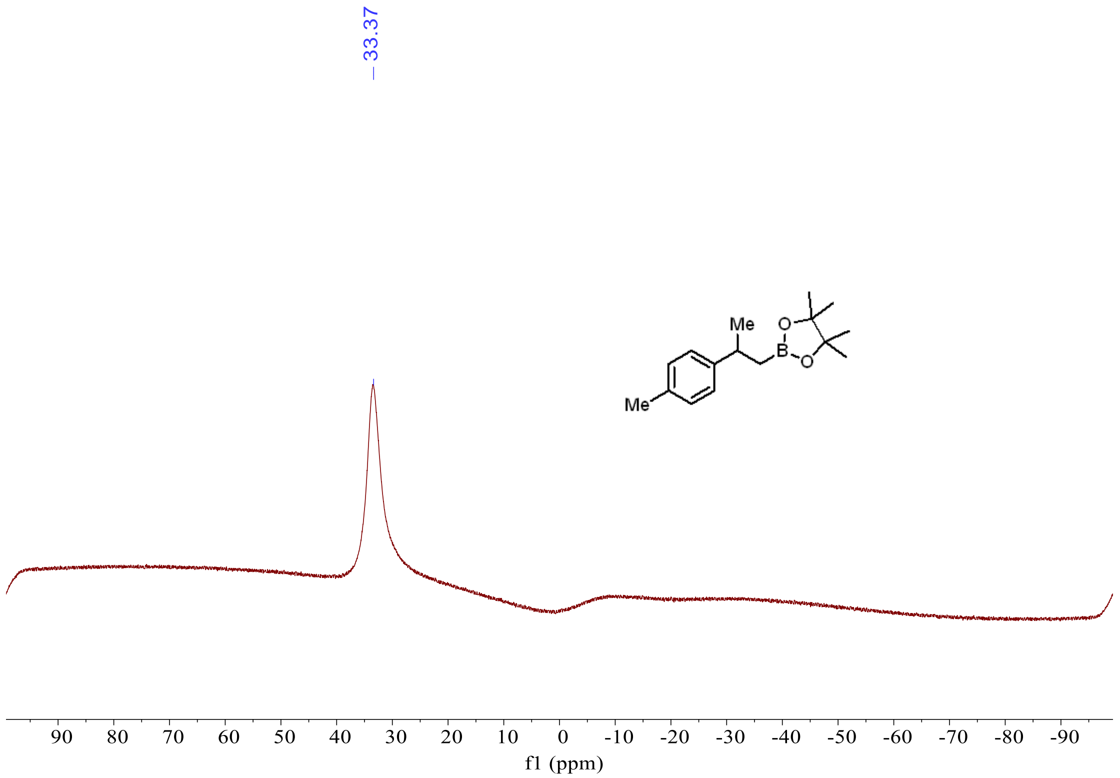

Supplementary Figure 244. ^{11}B NMR (128 MHz, $CDCl_3$) spectra for compound **60**

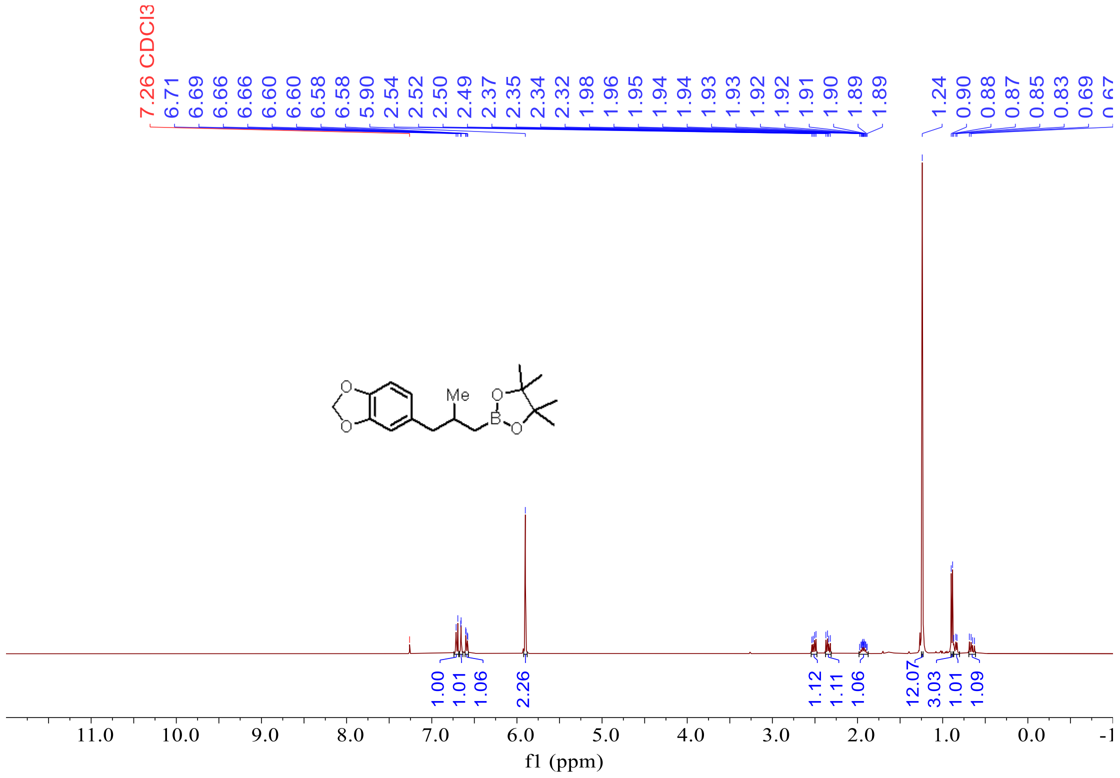

Supplementary Figure 245. ^{1}H NMR (400 MHz, $CDCl_3$) spectra for compound **61**

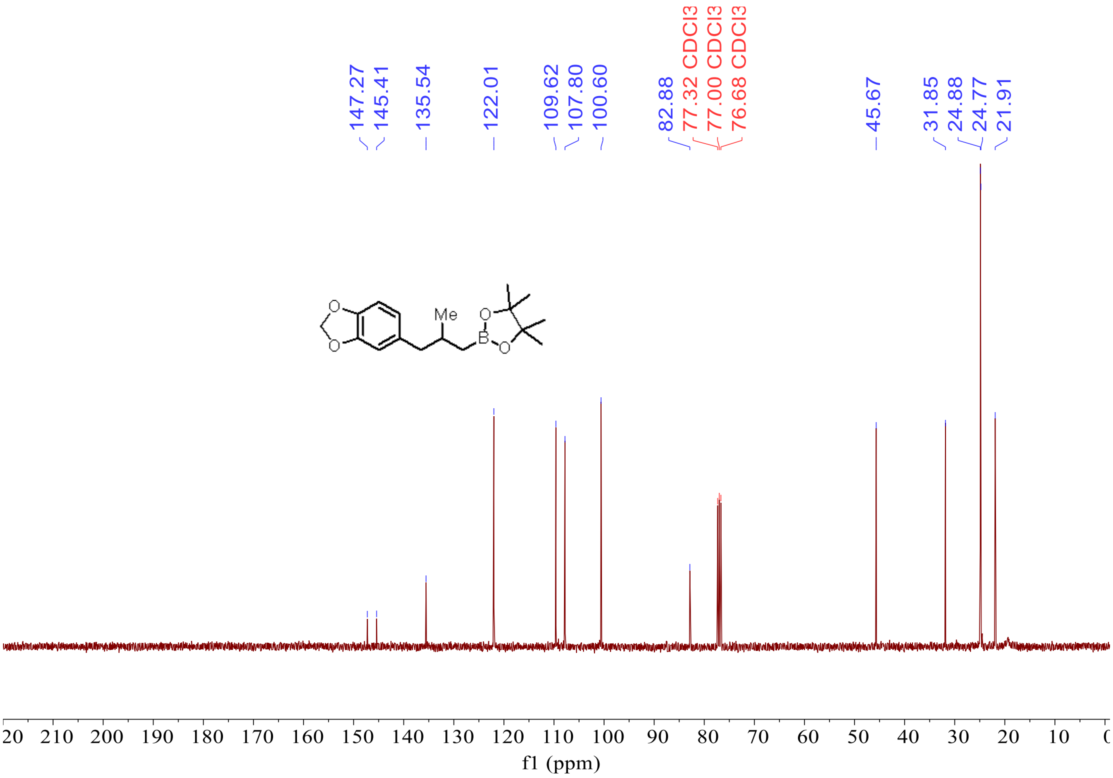

Supplementary Figure 246. ^{13}C NMR (101 MHz, $CDCl_3$) spectra for compound **61**

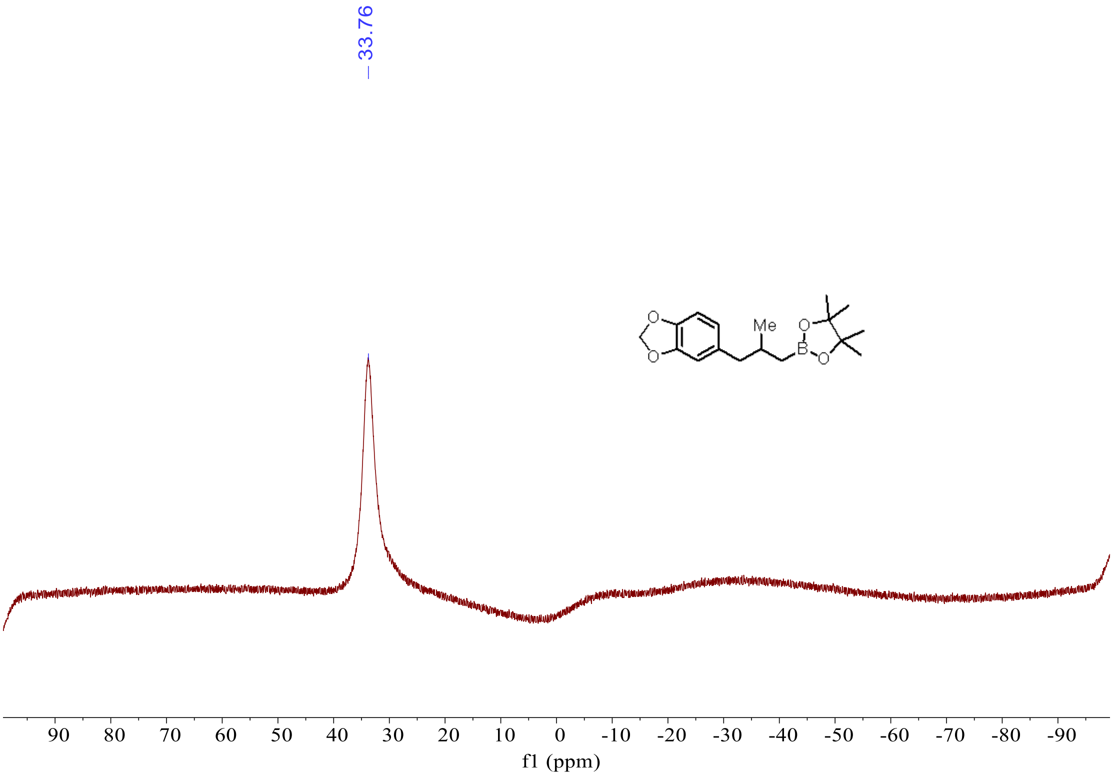

Supplementary Figure 247. ^{11}B NMR (128 MHz, $CDCl_3$) spectra for compound **61**

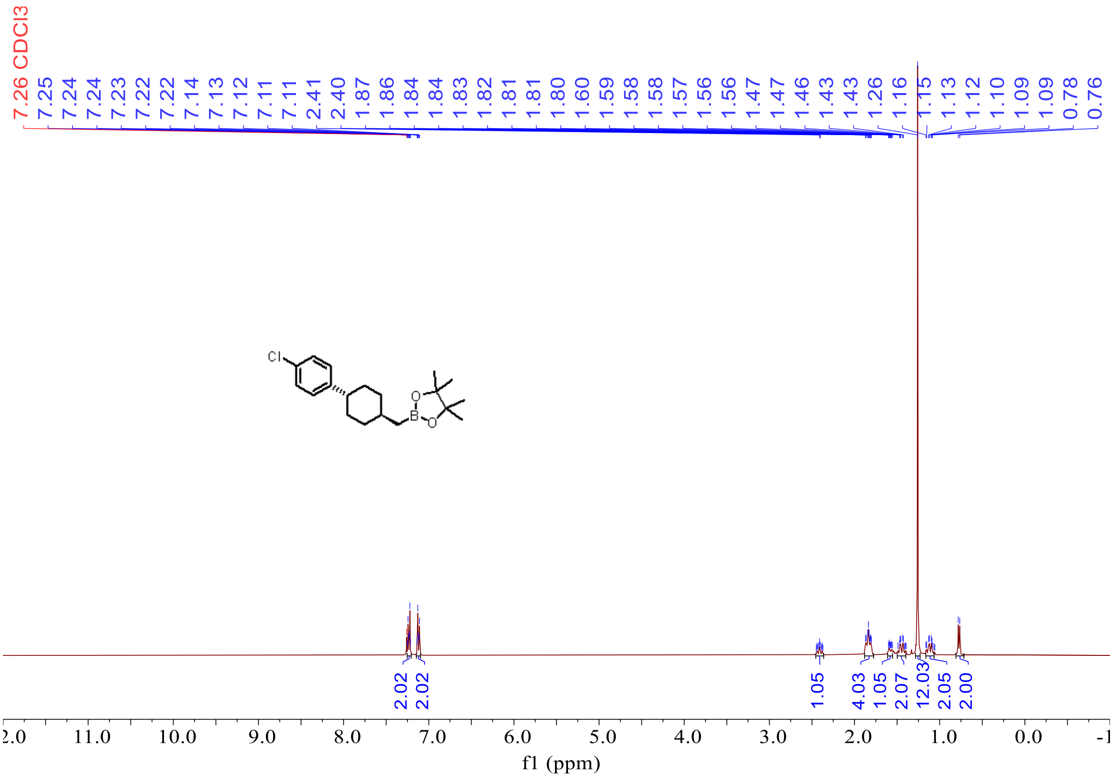

Supplementary Figure 248. ^{1}H NMR (400 MHz, $CDCl_3$) spectra for compound **62**

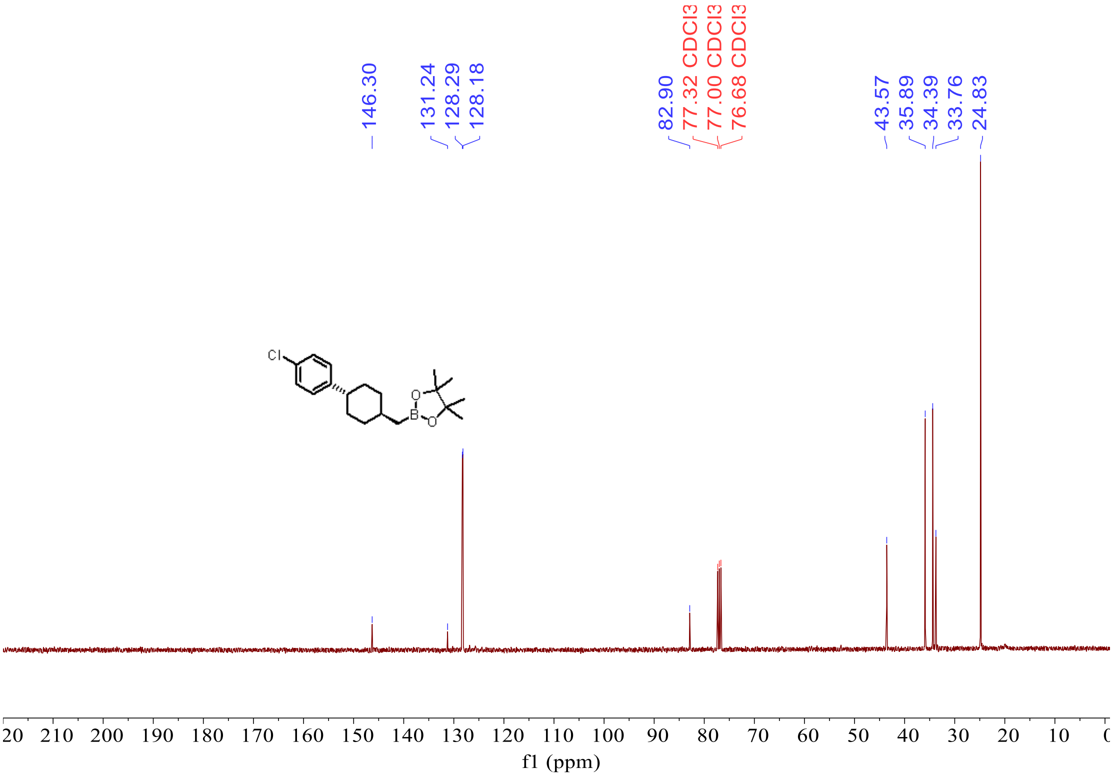

Supplementary Figure 249. ^{13}C NMR (101 MHz, $CDCl_3$) spectra for compound **62**

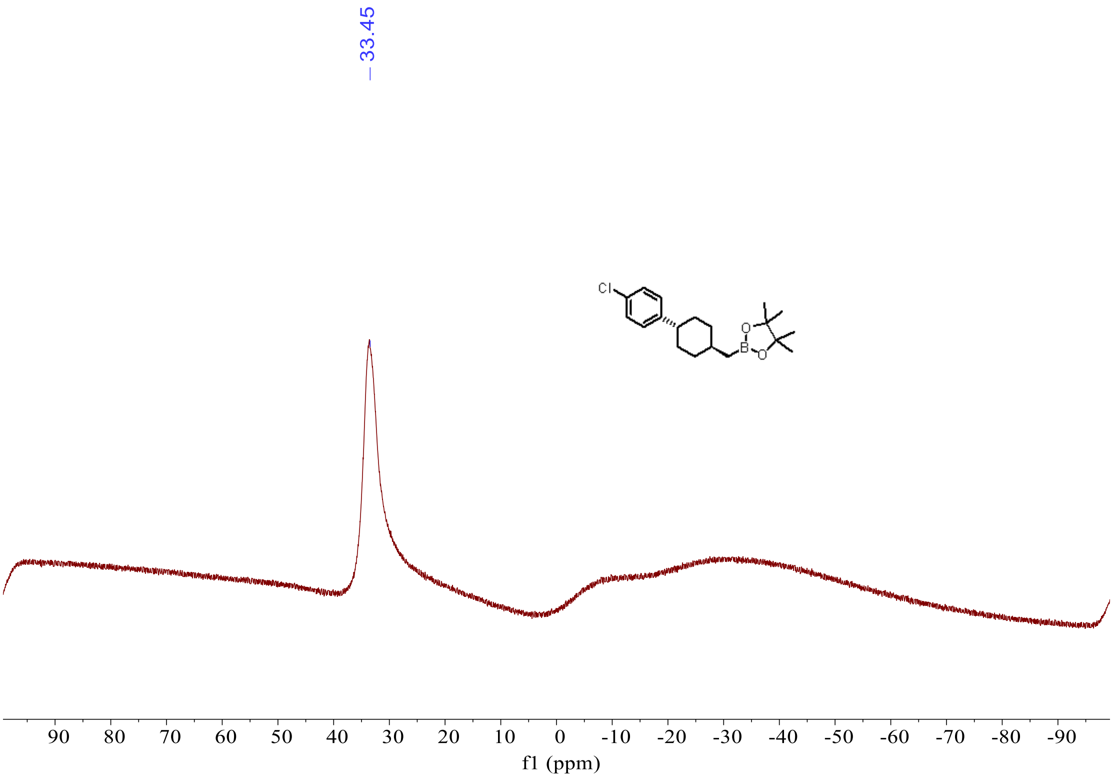

Supplementary Figure 250. ^{11}B NMR (128 MHz, $CDCl_3$) spectra for compound **62**

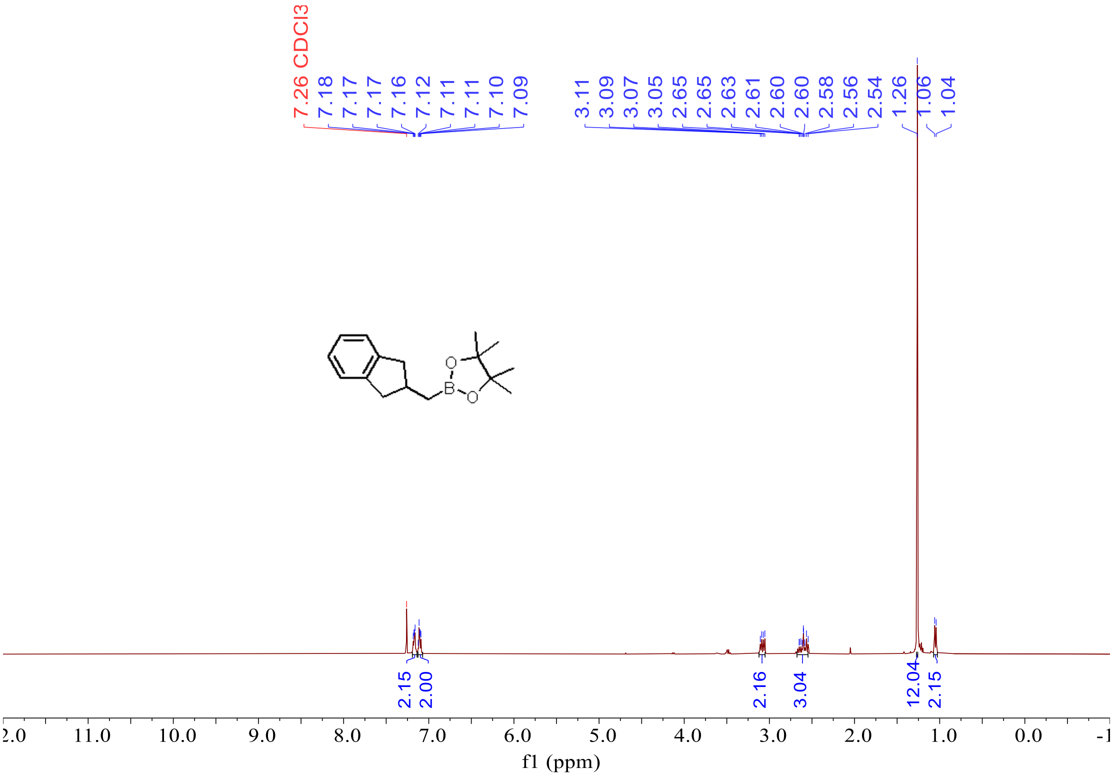

Supplementary Figure 251. ^{1}H NMR (400 MHz, $CDCl_3$) spectra for compound **63**

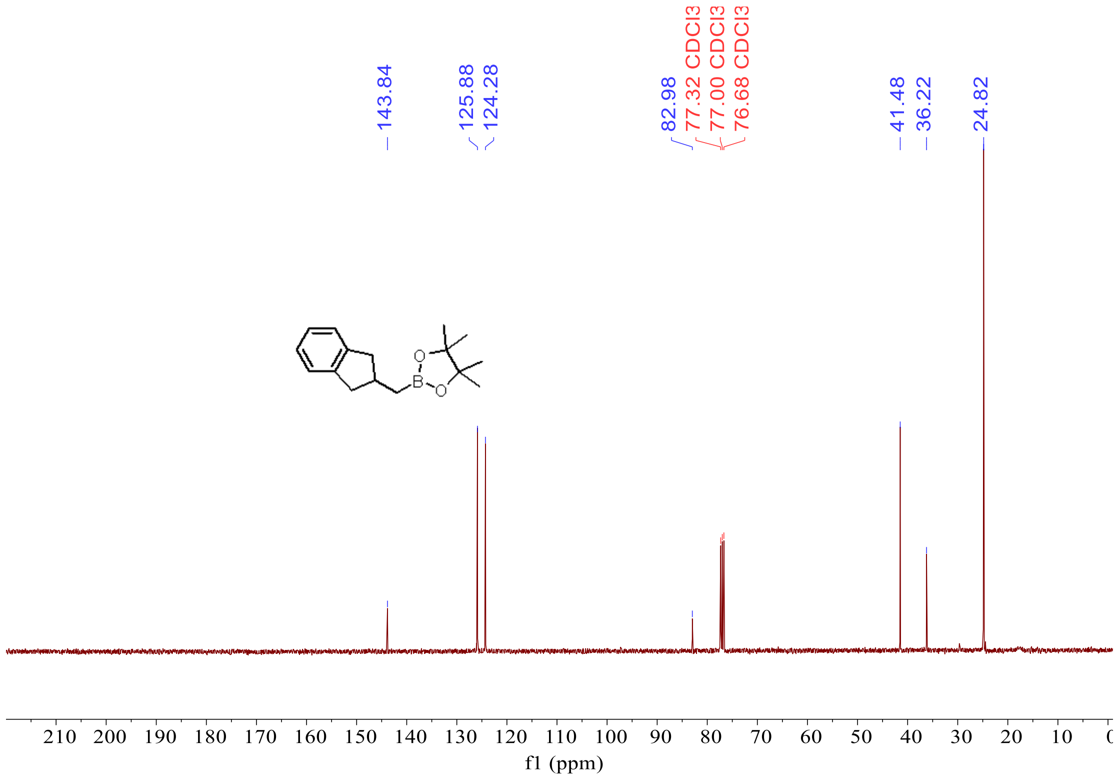

Supplementary Figure 252. ^{13}C NMR (101 MHz, $CDCl_3$) spectra for compound **63**

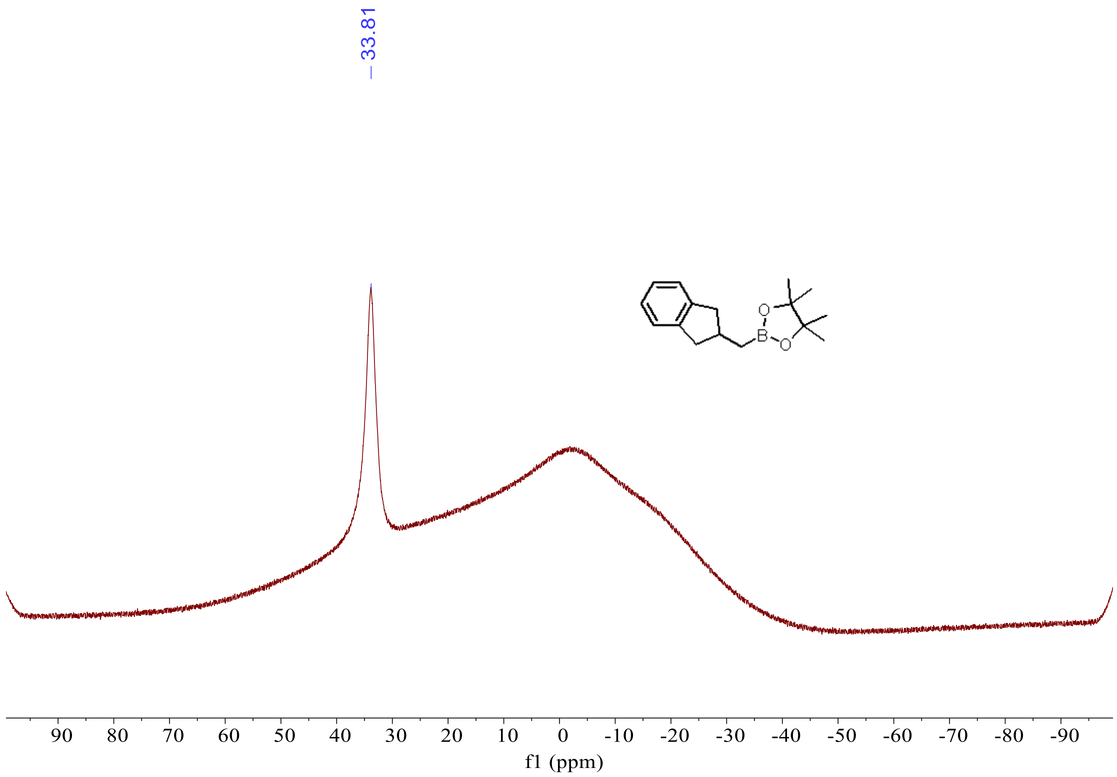

Supplementary Figure 253. ^{11}B NMR (128 MHz, $CDCl_3$) spectra for compound **63**

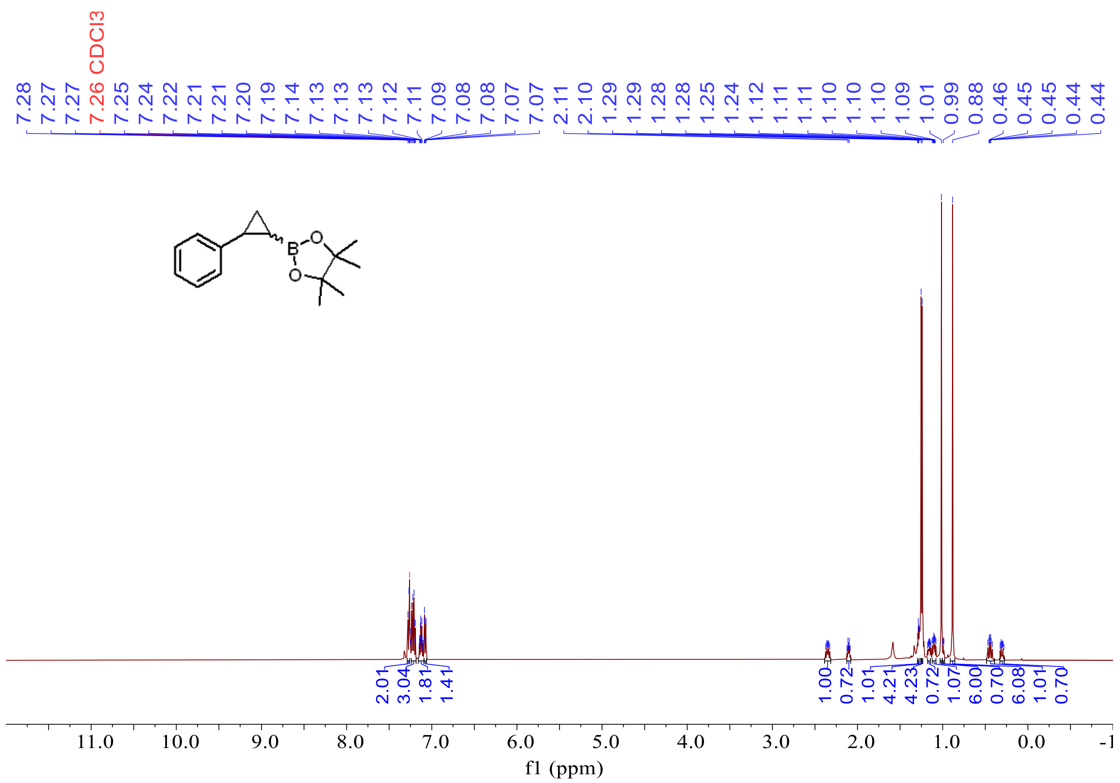

Supplementary Figure 254. ^{1}H NMR (500 MHz, $CDCl_3$) spectra for compound **64**

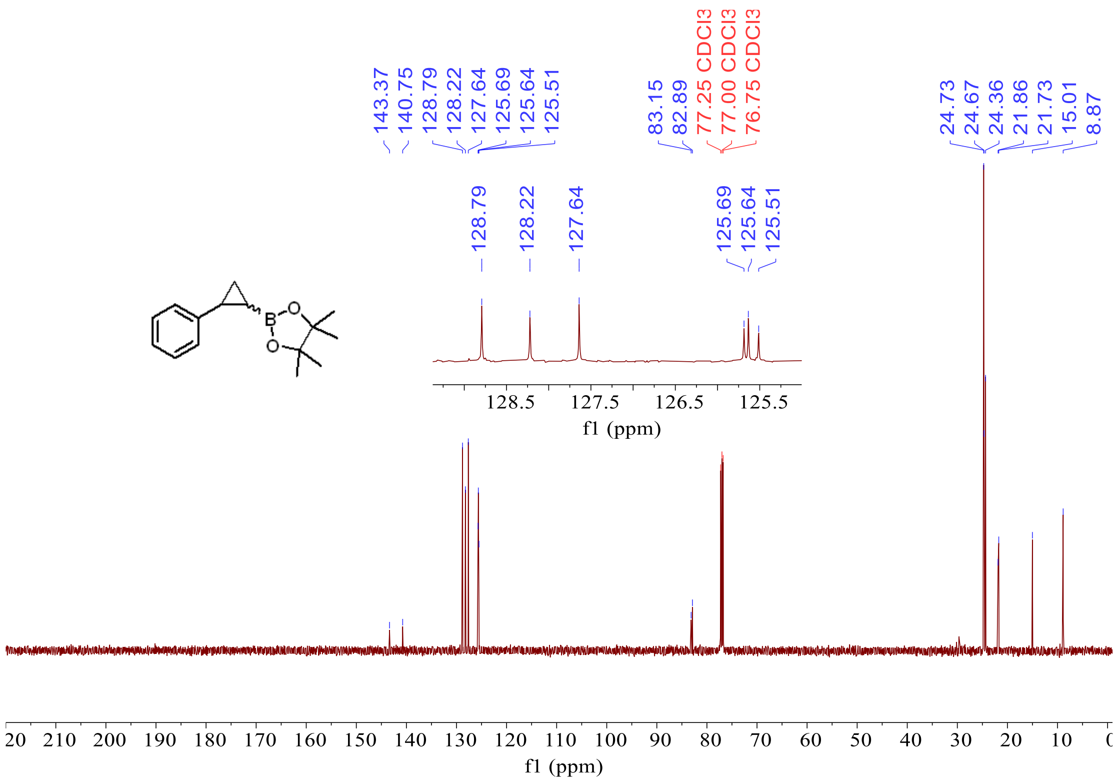

Supplementary Figure 255. ^{13}C NMR (126 MHz, $CDCl_3$) spectra for compound **64**

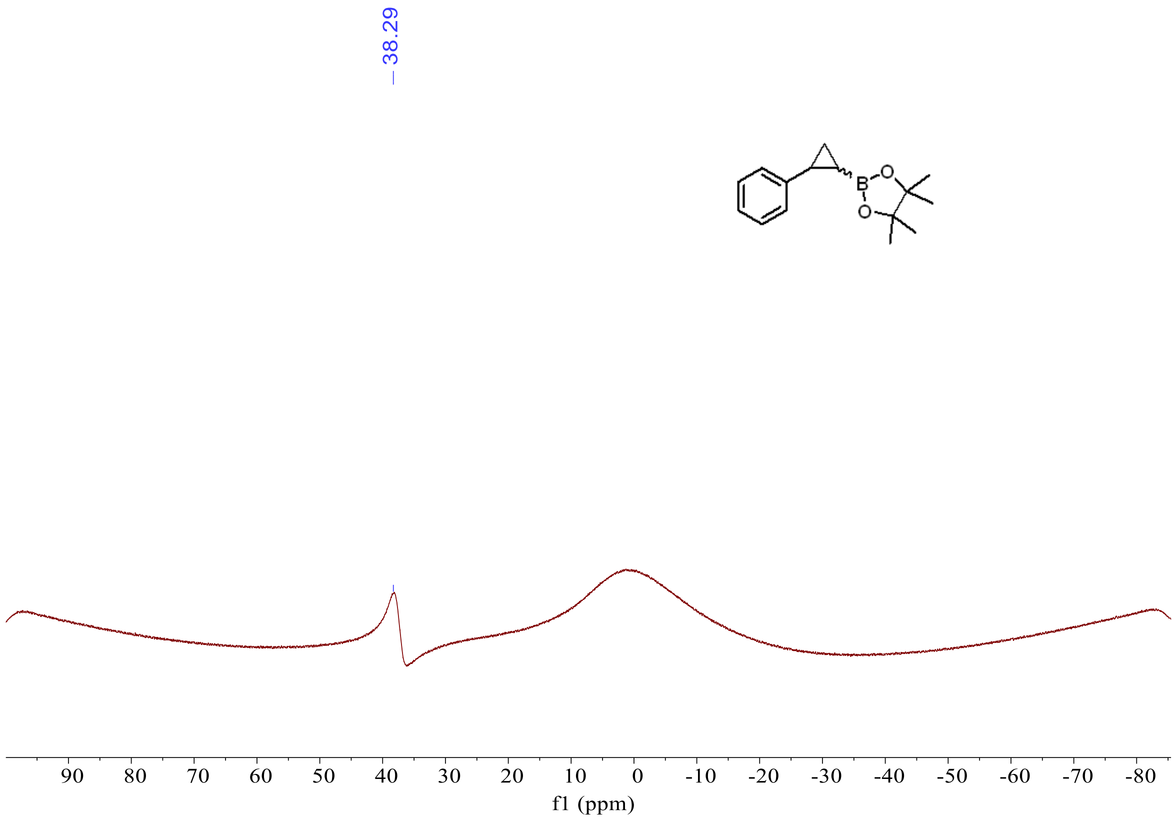

Supplementary Figure 256. ^{11}B NMR (160 MHz, $CDCl_3$) spectra for compound **64**

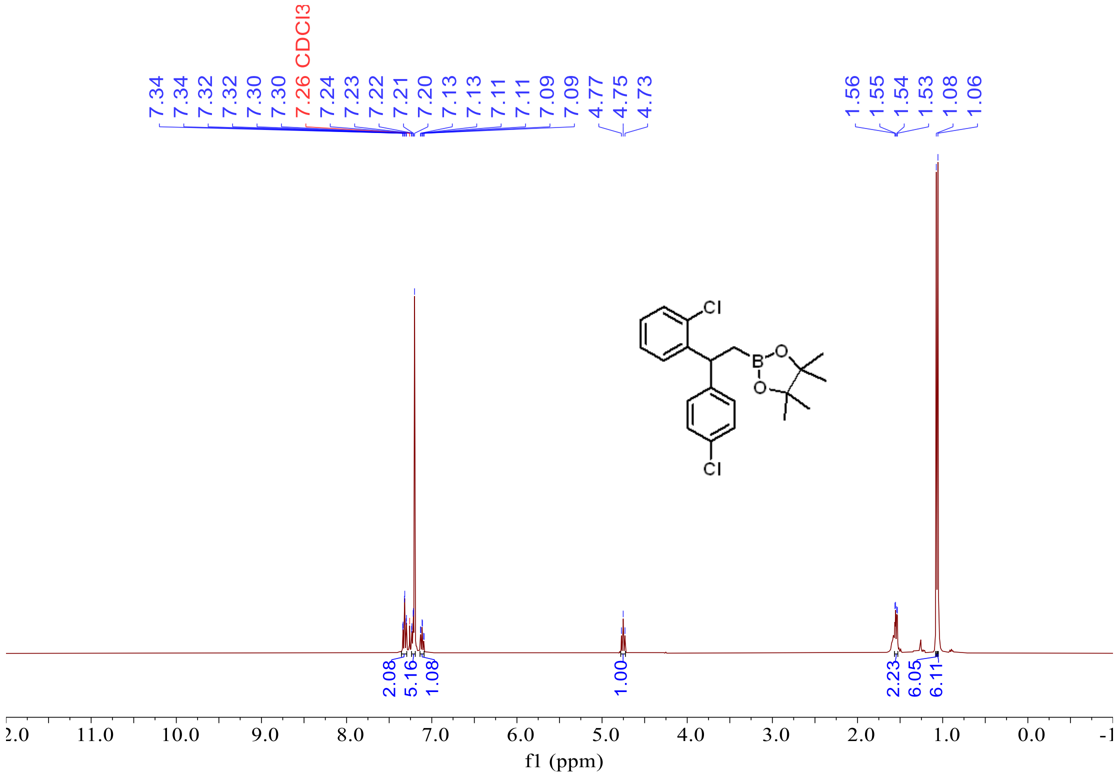

Supplementary Figure 257. ^{1}H NMR (500 MHz, $CDCl_3$) spectra for compound **65**

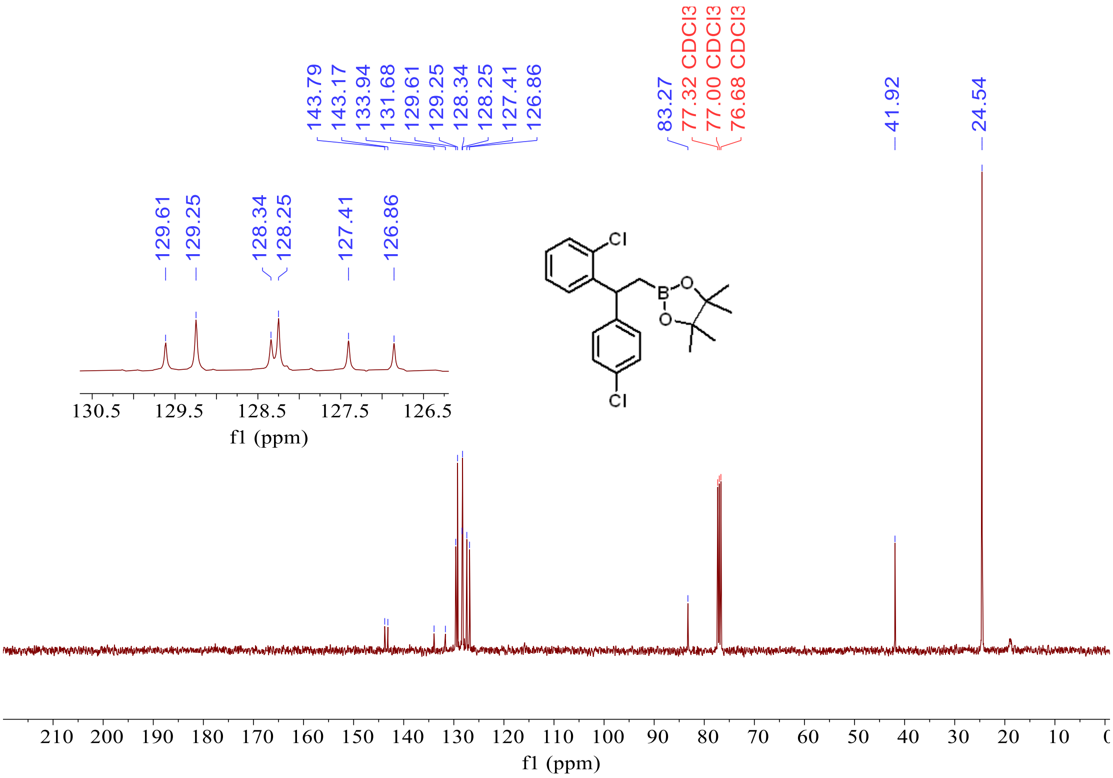

Supplementary Figure 258. ^{13}C NMR (126 MHz, $CDCl_3$) spectra for compound **65**

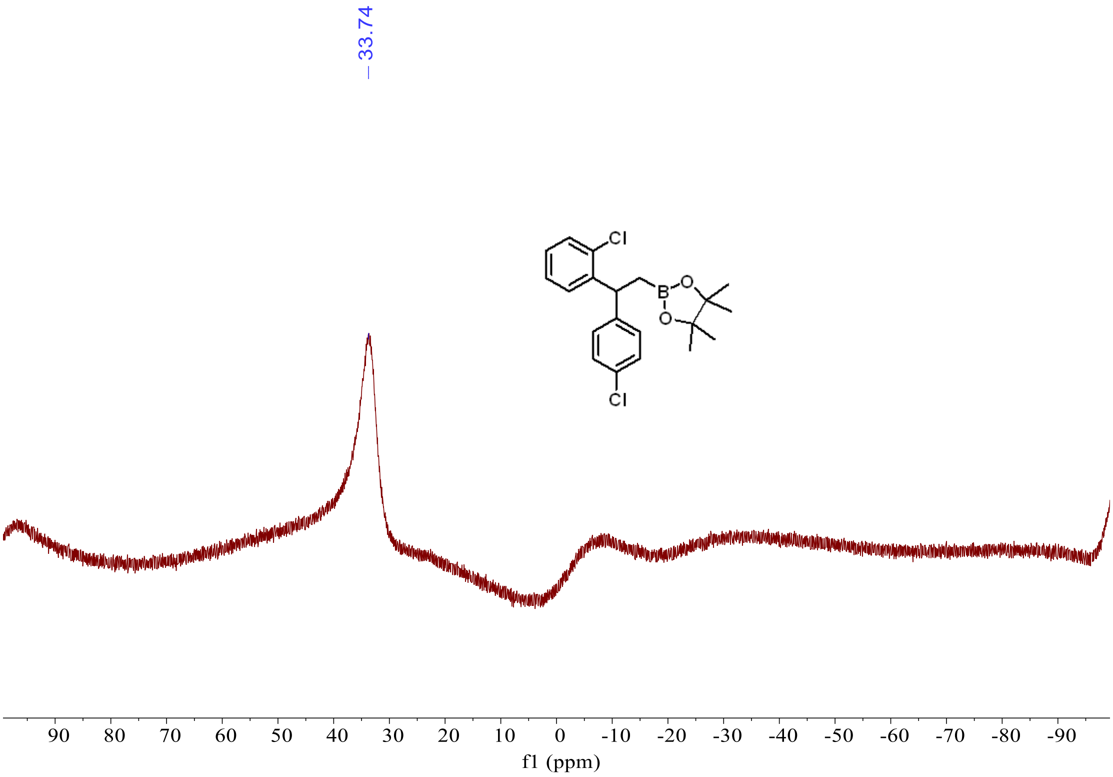

Supplementary Figure 259. ^{11}B NMR (128 MHz, $CDCl_3$) spectra for compound **65**

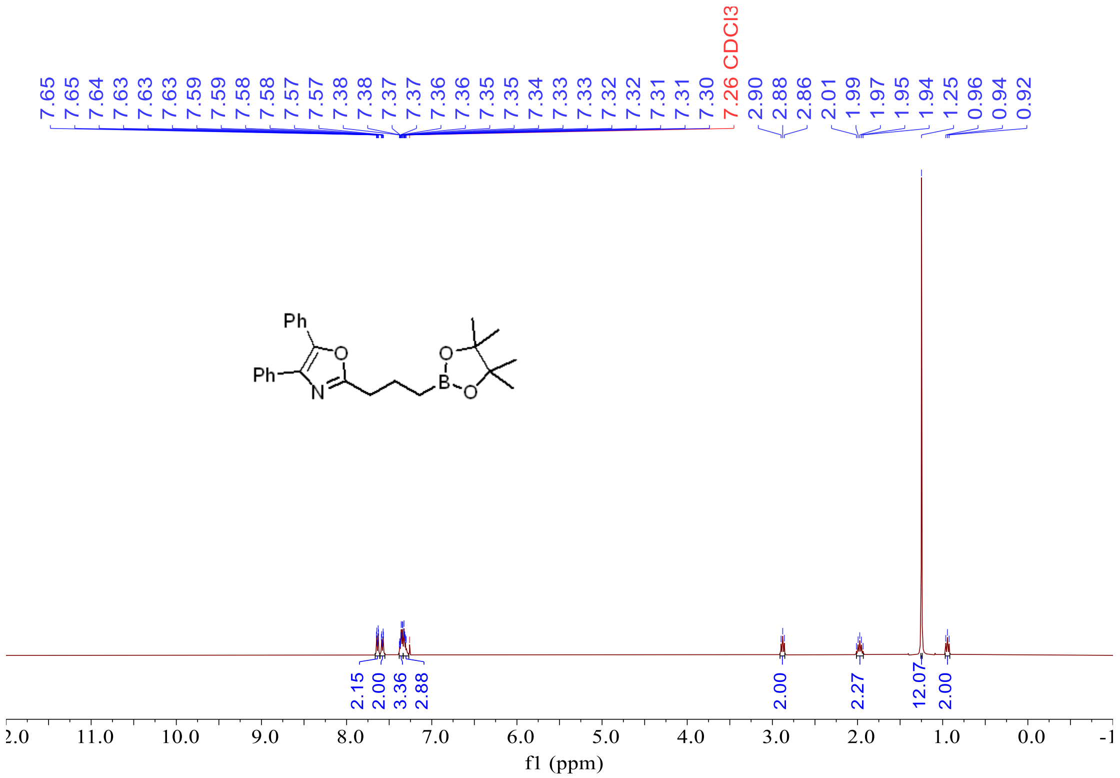

Supplementary Figure 260. ^{1}H NMR (400 MHz, $CDCl_3$) spectra for compound **66**

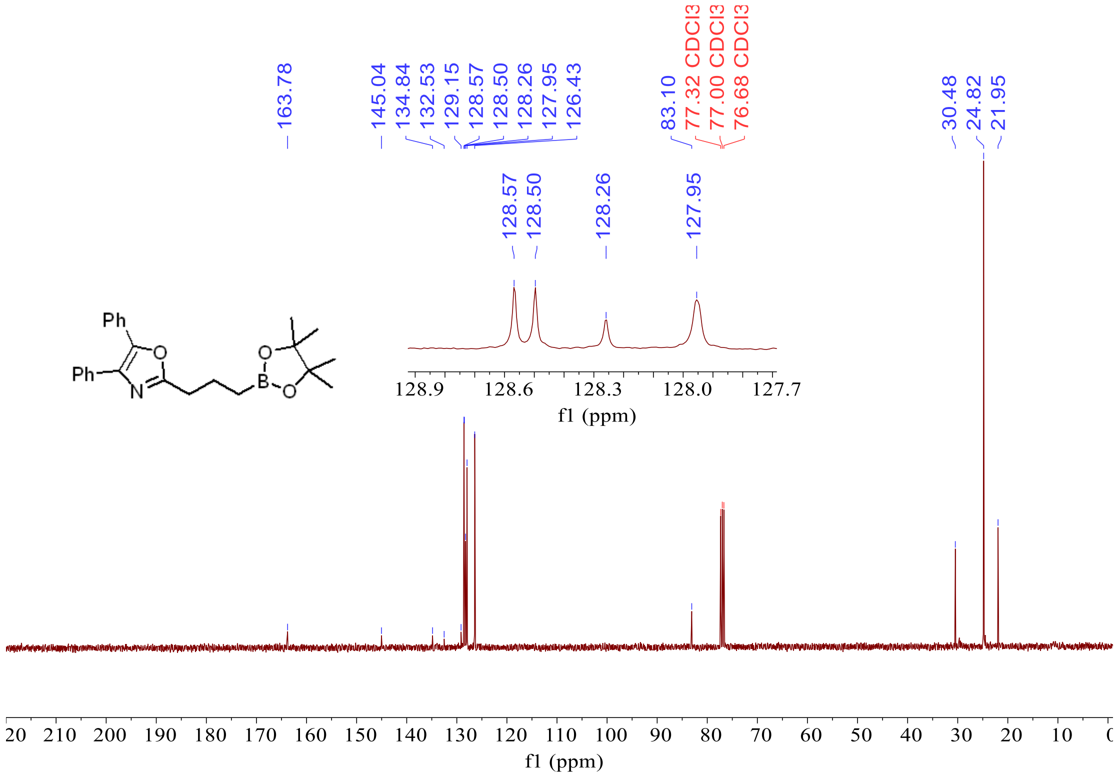

Supplementary Figure 261. ^{13}C NMR (101 MHz, $CDCl_3$) spectra for compound **66**

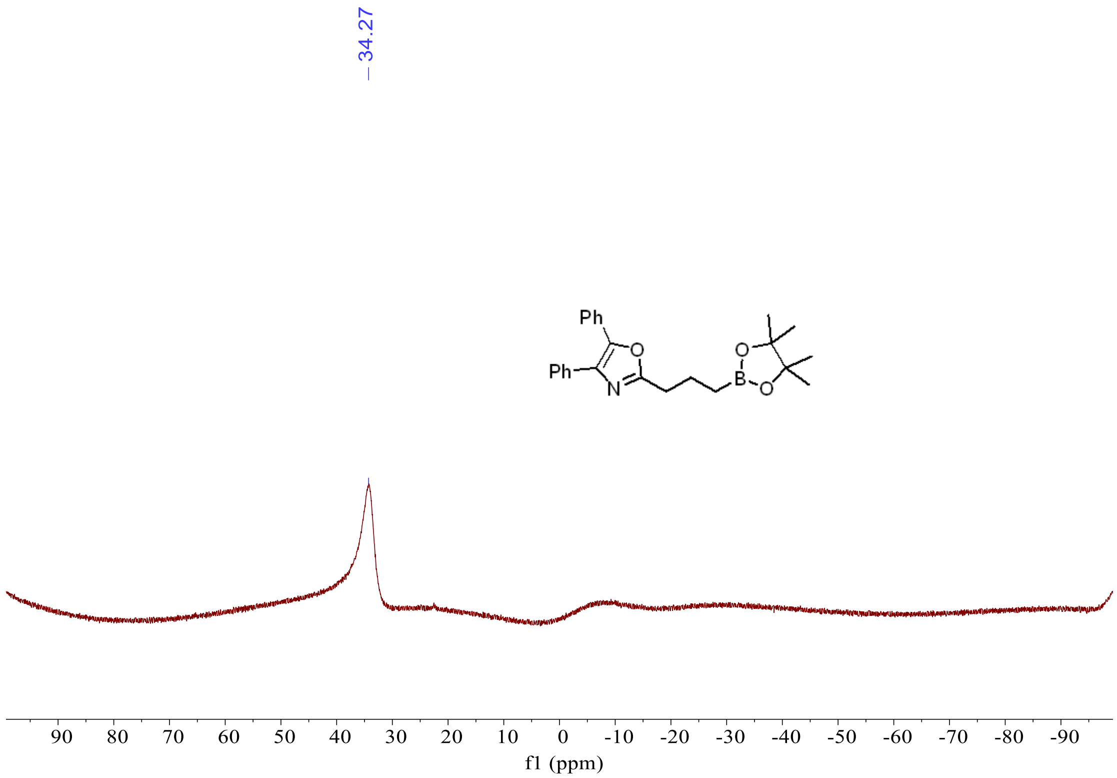

Supplementary Figure 262. ^{11}B NMR (128 MHz, $CDCl_3$) spectra for compound **66**

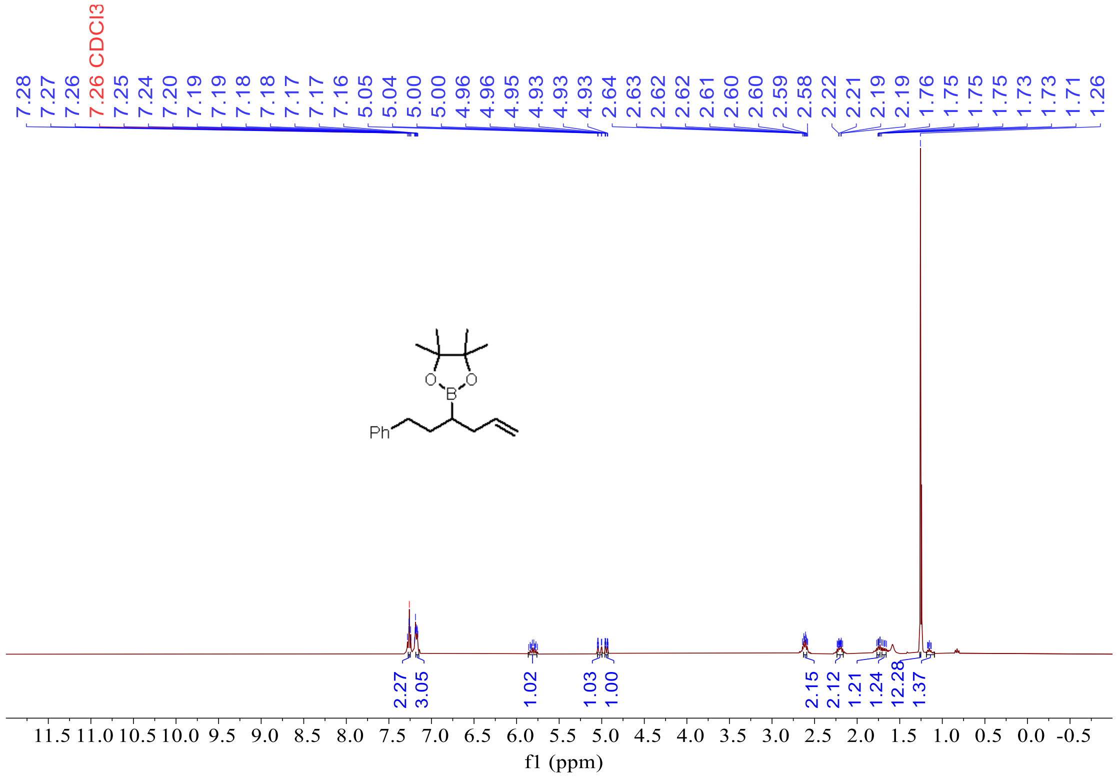

Supplementary Figure 263. ^{1}H NMR (400 MHz, $CDCl_3$) spectra for compound **67**

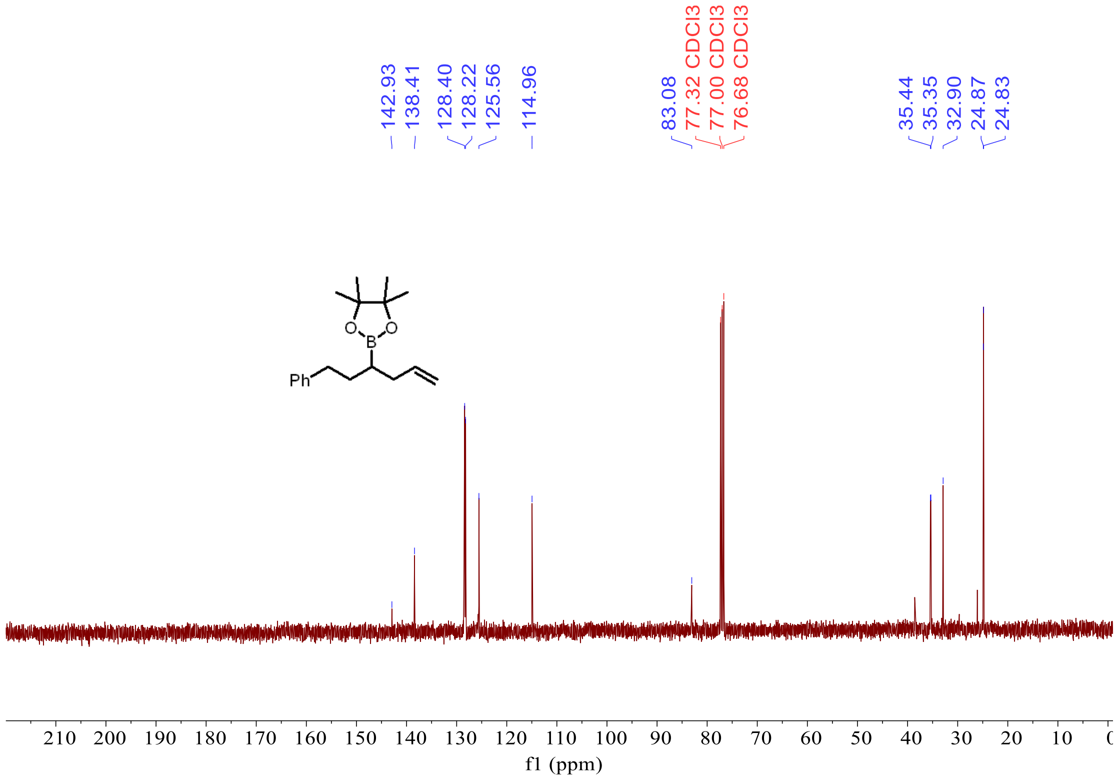

Supplementary Figure 264. ^{13}C NMR (101 MHz, $CDCl_3$) spectra for compound **67**

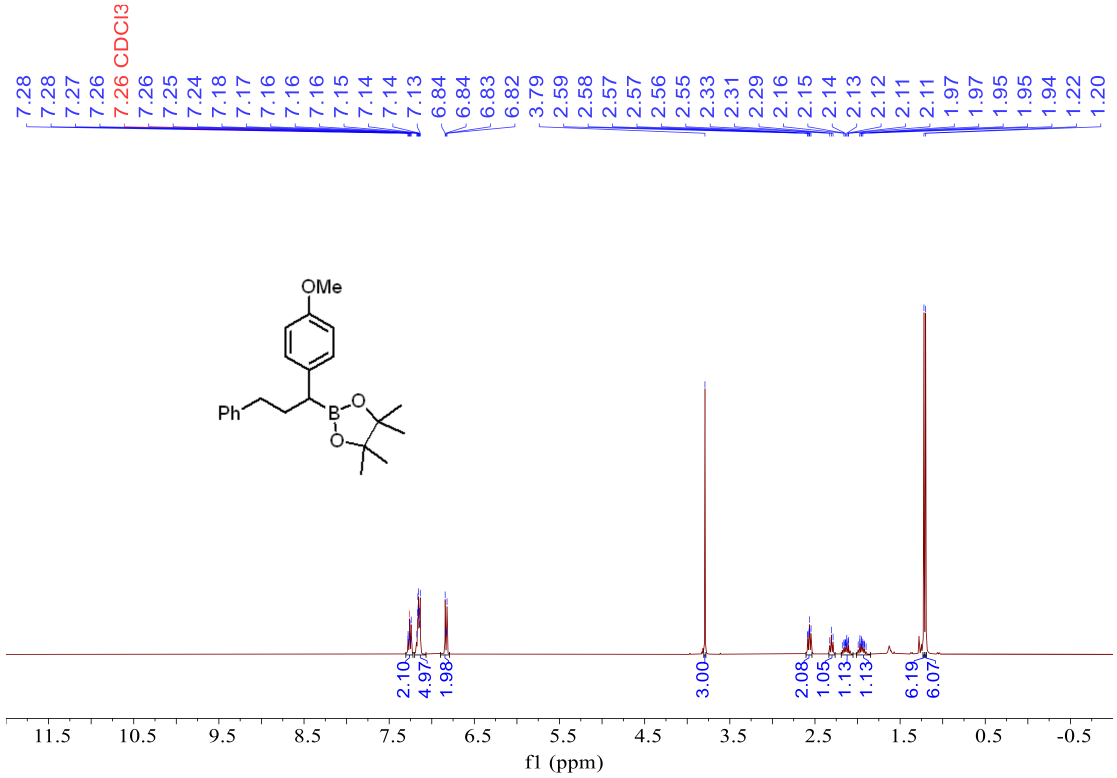

Supplementary Figure 265. ^{1}H NMR (400 MHz, $CDCl_3$) spectra for compound **68**

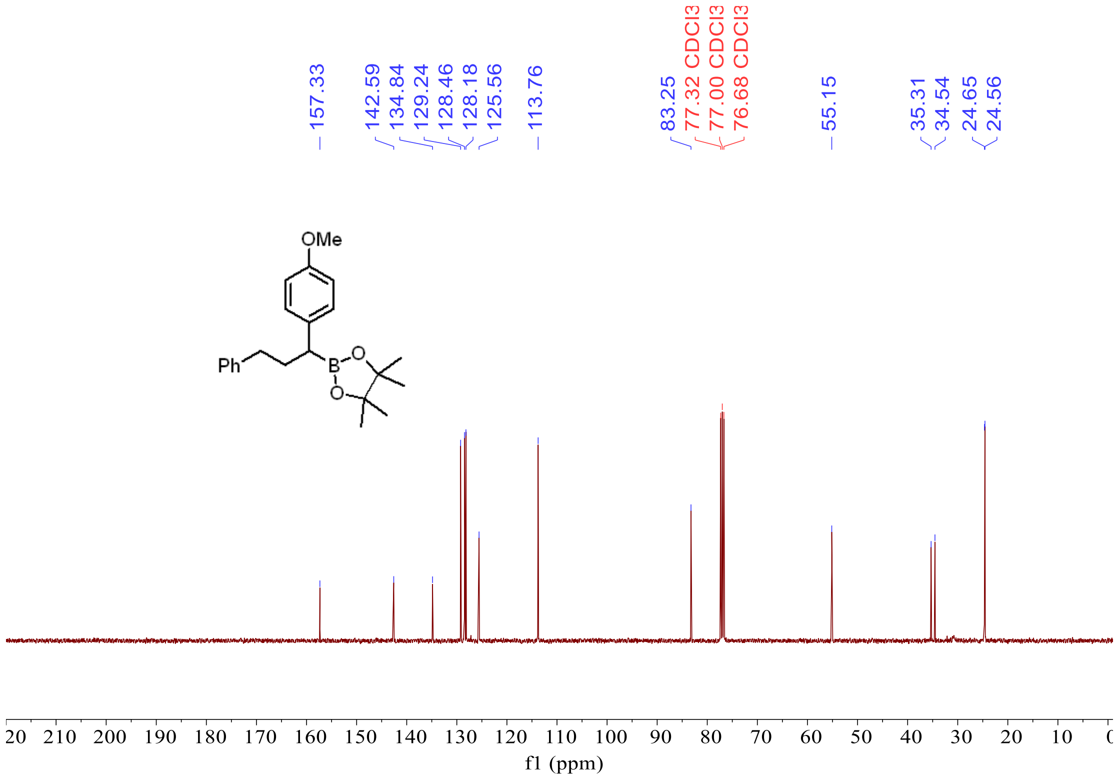

Supplementary Figure 266. ^{13}C NMR (101 MHz, $CDCl_3$) spectra for compound **68**

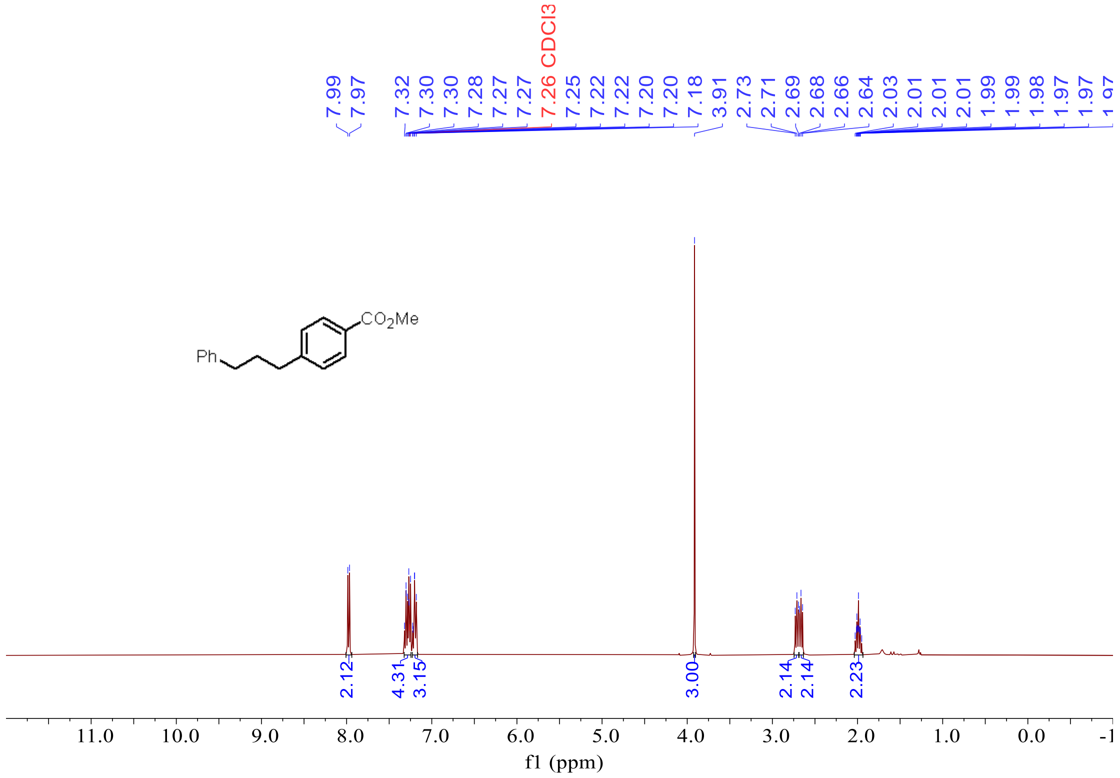

Supplementary Figure 267. ^{1}H NMR (400 MHz, $CDCl_3$) spectra for compound **69**

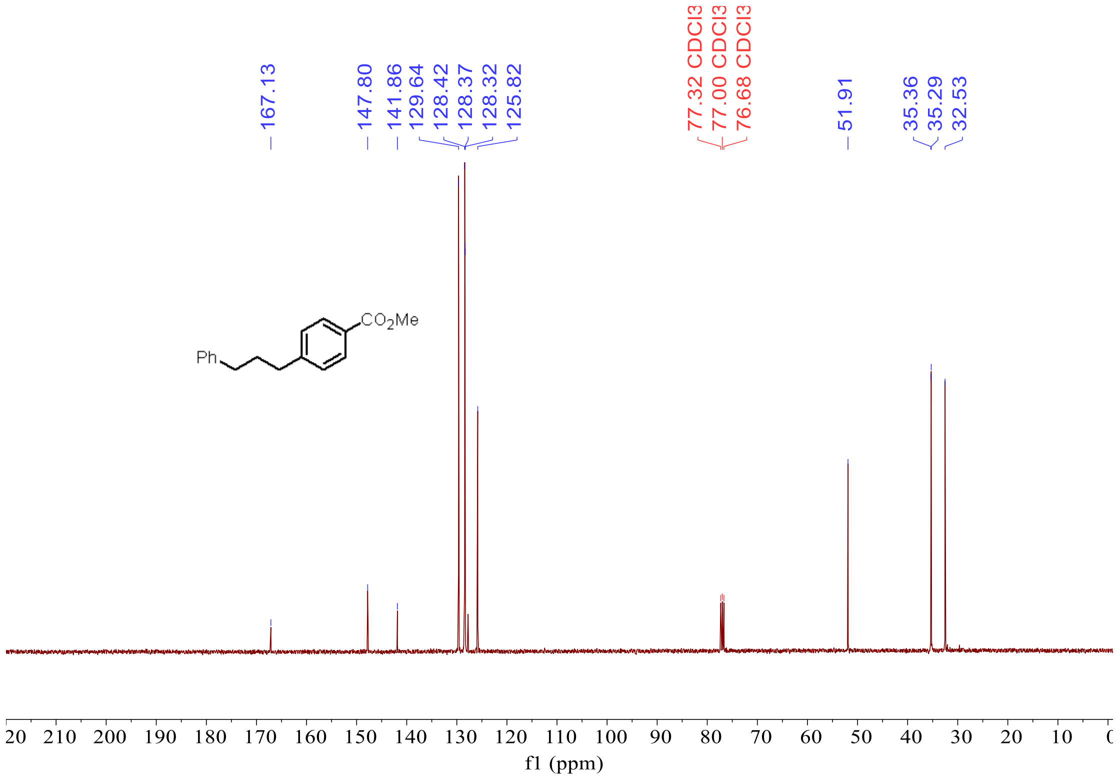

Supplementary Figure 268. ^{13}C NMR (101 MHz, $CDCl_3$) spectra for compound **69**

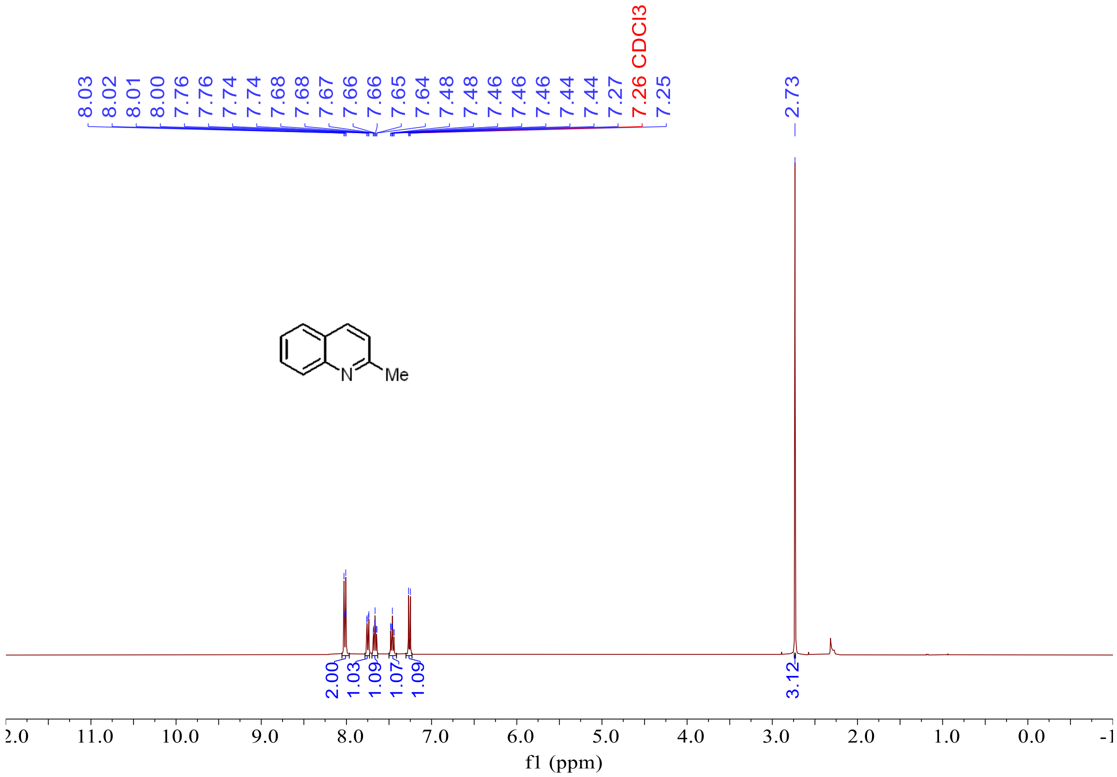

Supplementary Figure 269. ^{1}H NMR (400 MHz, $CDCl_3$) spectra for compound **70**

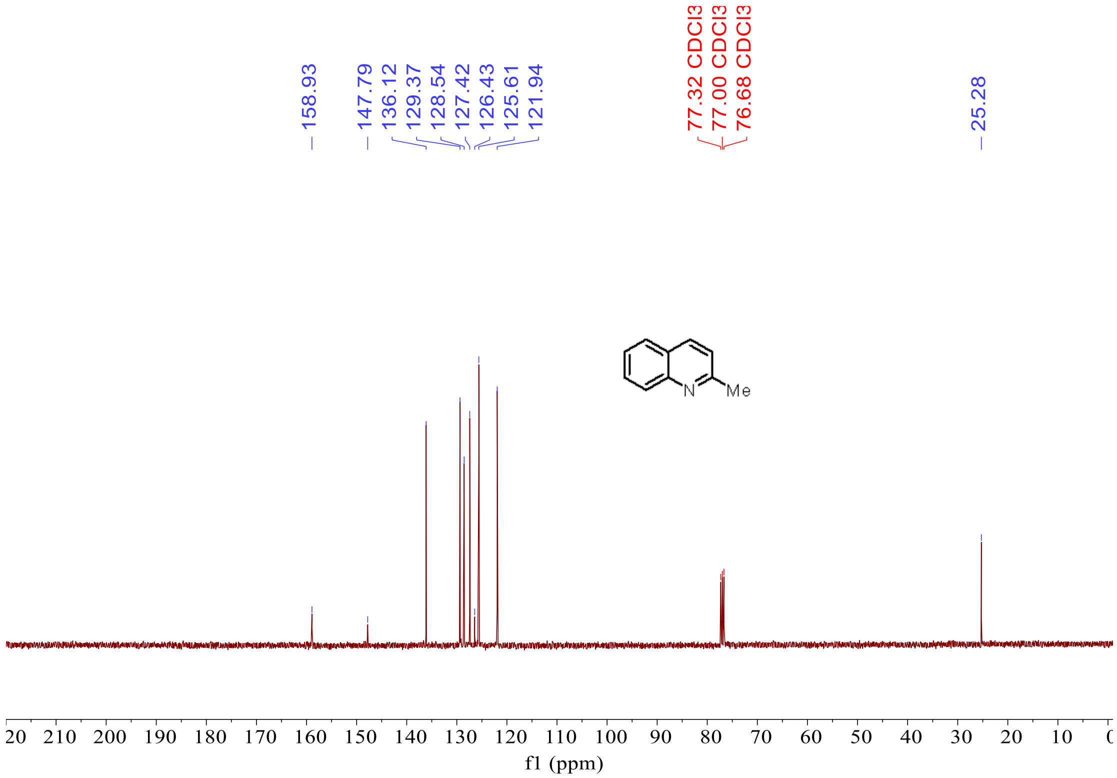

Supplementary Figure 270. ^{13}C NMR (101 MHz, $CDCl_3$) spectra for compound **70**

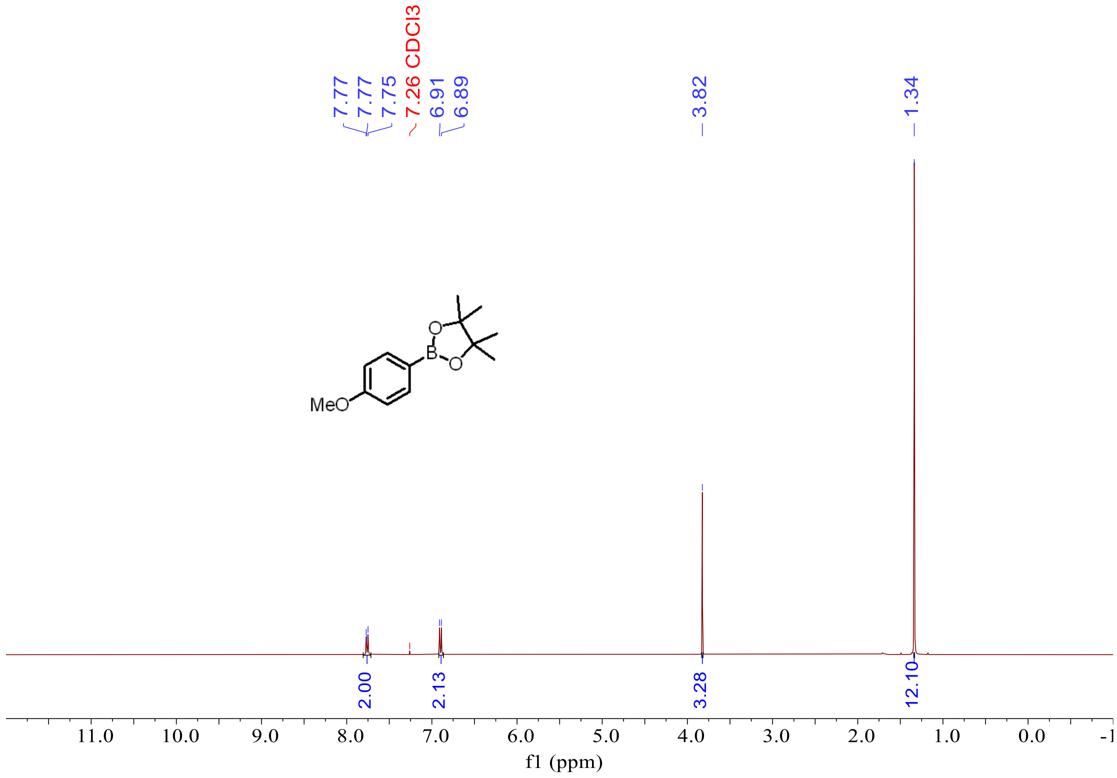

Supplementary Figure 271. ^{1}H NMR (400 MHz, $CDCl_3$) spectra for compound **71**

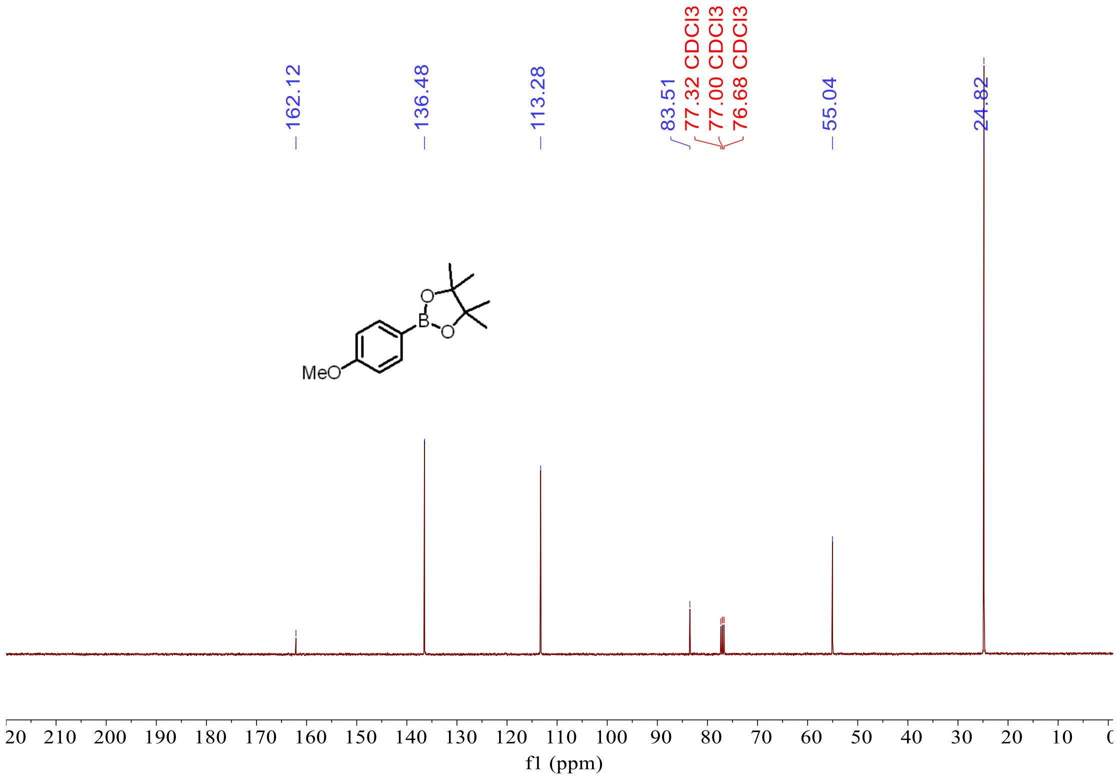

Supplementary Figure 272. ^{13}C NMR (101 MHz, $CDCl_3$) spectra for compound **71**

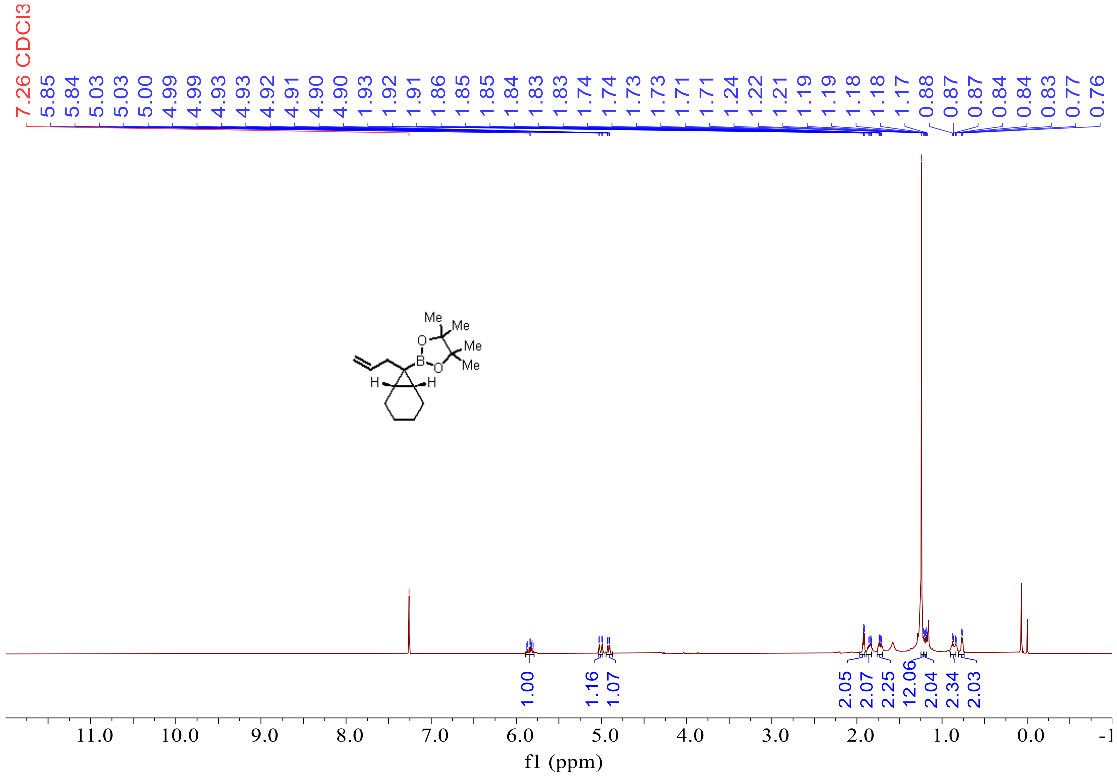

Supplementary Figure 273. ^{1}H NMR (500 MHz, $CDCl_3$) spectra for compound **72**

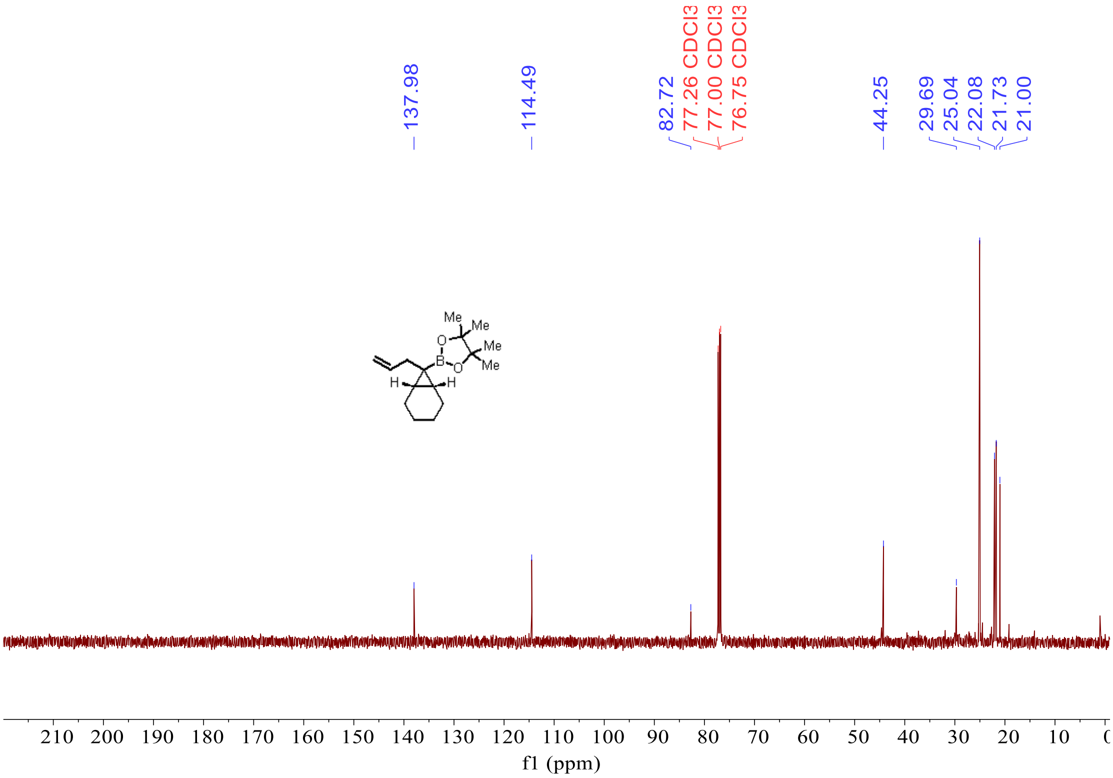

Supplementary Figure 274. ^{13}C NMR (126 MHz, $CDCl_3$) spectra for compound **72**

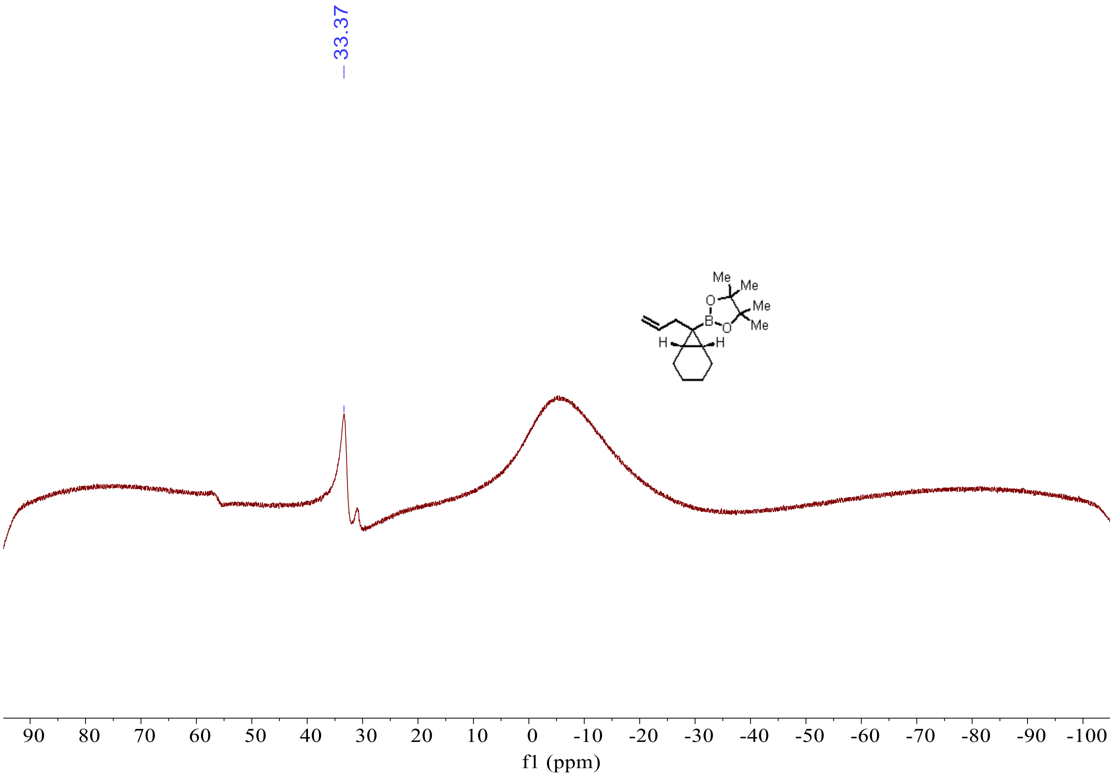

Supplementary Figure 275. ^{11}B NMR (160 MHz, $CDCl_3$) spectra for compound **72**

Supplementary References

1. Hong, K., Liu, X. & Morken, J. P. Simple access to elusive *α*-Boryl carbanions and their alkylation: an umpolung construction for organic synthesis. *J. Am. Chem. Soc.* **136**, 10581-10584 (2014).
2. Hu, M. & Ge, S. Versatile cobalt-catalyzed regioselective chain-walking double hydroborylation of 1, n-dienes to access *gem*-bis(boryl)alkanes. *Nat. Commun.* **11**, 765 (2020).
3. Fu, L., Chen, Q., Wang, Z. & Nishihara, Y. Palladium-catalyzed decarbonylative alkylation of acyl fluorides. *Org. Lett.* **22**, 2350−2353 (2020).
4. Jo, W. et al. $ZnMe_2$-mediated, direct alkylation of electron deficient *N*-heteroarenes with 1,1-diborylalkanes: scope and mechanism. *J. Am. Chem. Soc.* **142**, 13235−13245 (2020).
5. Lee, Y., Baek, S.-Y., Park, J., Kim, S.-T., Tussupbayev, S., Kim, J., Baik, M.-H. & Cho, S. H. Chemoselective coupling of 1,1-bis[(pinacolato)boryl]alkanes for the transition-metal-free borylation of aryl and vinyl halides: A combined experimental and theoretical investigation. *J. Am. Chem. Soc.* **139**, 976–984 (2017).
6. Wang, L. et al. C–O functionalization of *α*-oxyboronates: A deoxygenative *gem*-diborylation and *gem*-silylborylation of aldehydes and ketones. *J. Am. Chem. Soc.* **139**, 5257-5264 (2017).
7. He, Z. et al. Cooperation between an alcoholic proton and boryl species in the catalytic *gem*-hydrodiborylation of carboxylic esters to access 1,1-diborylalkanes. *Org. Chem. Front.* **6**, 900-907 (2019).
8. Wang, B. et al. Electrooxidative activation of B−B bond in B_2cat_2: access to *gem*-diborylalkanes via paired electrolysis. *Angew. Chem.Int. Ed.* **62**, e2022181 (2023)
9. Wang, B. et al. Electrochemical borylation of alkyl halides: fast, scalable access to alkyl boronic esters. *J. Am. Chem. Soc.* **143**, 12985-12991 (2021).
10. Li, S. et al. Site-fixed hydroborylation of terminal and internal alkenes using $BX_3/^iPr_2NEt$. *Angew. Chem. Int. Ed.* **60**, 26238-26245 (2021).
11. An, L., Tong, F.-F., Zhang, S. & Zhang, X. Stereoselective functionalization of racemic cyclopropylzinc reagents via enantiodivergent relay coupling. *J. Am. Chem. Soc.* **142**, 11884-11892 (2020).
12. Huang, H., Zhang, G., Gong, L., Zhang, S. & Chen, Y. Visible-light-induced chemoselective deboronative alkynylation under biomolecule-compatible conditions. *J. Am. Chem. Soc.* **136**, 2280-2283 (2014)
13. Fawcett, A. et al. Photoinduced decarboxylative borylation of carboxylic acids. *Science* **357**, 283 (2017).
14. Wu, J., Bär, R. M., Guo, L., Noble, A. & Aggarwal, V. K. Photoinduced deoxygenative borylations of aliphatic alcohols. *Angew. Chem. Int. Ed.* **58**, 18830-18834 (2019).
15. Perdew, J. P., Burke, K. & Ernzerhof, M. Generalized Gradient Approximation Made Simple. *Phys. Rev. Lett.* **77**, 3865-3868 (1996).
16. Grimme, S., Ehrlich, S. & Goerigk, L. Effect of the damping function in dispersion corrected density functional theory. *J. Comput. Chem.* **32**, 1456-1465 (2011).
17. Weigend, F. & Ahlrichs, R. Balanced basis sets of split valence, triple zeta valence and quadruple zeta valence quality for H to Rn: Design and assessment of accuracy. *Phys. Chem. Chem. Phys.* **7**, 3297-3305 (2005).

18. Miertuš, S., Scrocco, E. & Tomasi, J. Electrostatic interaction of a solute with a continuum. A direct utilizaion of AB initio molecular potentials for the prevision of solvent effects. *Chem. Phys.* **55**, 117-129 (1981).
19. Miertuš, S. & Tomasi, J. Approximate evaluations of the electrostatic free energy and internal energy changes in solution processes. *Chem. Phys.* **65**, 239-245 (1982).
20. Pascual-ahuir, J. L., Silla, E. & Tuñon, I. GEPOL: An improved description of molecular surfaces. III. A new algorithm for the computation of a solvent-excluding surface. *J. Comput. Chem.* **15**, 1127-1138 (1994).
21. Lu, T. & Chen, Q. Shermo: A general code for calculating molecular thermochemistry properties. *Comput. Theor. Chem.* **1200**, 113249 (2021).
22. Fukui, K. Formulation of the reaction coordinate. *J. Phys. Chem.*, **74**, 4161-4163, (1970).
23. Fukui, K. The path of chemical reactions - the IRC approach. *Accounts. Chem. Res.* **14**, 363-368 (1981).
24. Dreuw, A. & Head-Gordon, M. Single-Reference ab Initio Methods for the Calculation of Excited States of Large Molecules. *Chem. Rev.* **105**, 4009-4037 (2005).
25. Gaussian 09, Revision B.01, M. J. Frisch, G. W. Trucks, H. B. Schlegel, G. E. Scuseria, M. A. Robb, J. R. Cheeseman, G. Scalmani, V. Barone, B. Mennucci, G. A. Petersson, H. Nakatsuji, M. Caricato, X. Li, H. P. Hratchian, A. F. Izmaylov, J. Bloino, G. Zheng, J. L. Sonnenberg, M. Hada, M. Ehara, K. Toyota, R. Fukuda, J. Hasegawa, M. Ishida, T. Nakajima, Y. Honda, O. Kitao, H. Nakai, T. Vreven, J. A. Montgomery, Jr., J. E. Peralta, F. Ogliaro, M. Bearpark, J. J. Heyd, E. Brothers, K. N. Kudin, V. N. Staroverov, T. Keith, R. Kobayashi, J. Normand, K. Raghavachari, A. Rendell, J. C. Burant, S. S. Iyengar, J. Tomasi, M. Cossi, N. Rega, J. M. Millam, M. Klene, J. E. Knox, J. B. Cross, V. Bakken, C. Adamo, J. Jaramillo, R. Gomperts, R. E. Stratmann, O. Yazyev, A. J. Austin, R. Cammi, C. Pomelli, J. W. Ochterski, R. L. Martin, K. Morokuma, V. G. Zakrzewski, G. A. Voth, P. Salvador, J. J. Dannenberg, S. Dapprich, A. D. Daniels, O. Farkas, J. B. Foresman, J. V. Ortiz, J. Cioslowski, and D. J. Fox, Gaussian, Inc., Wallingford CT, 2013.
26. Neese, F., Wennmohs, F., Becker, U. & Riplinger, C. The ORCA quantum chemistry program package. *J. Chem. Phys.* **152**, 224108 (2020).
27. Neese, F. Wiley Interdiscip. *Rev. Comput. Mol. Sci.* **12**, 1606-1620 (2022).
28. Mardirossian, N. & Head-Gordon, M. ωB97M-V: A combinatorially optimized, range-separated hybrid, meta-GGA density functional with VV10 nonlocal correlation. *J. Chem. Phys.* **144**, 214110 (2016).
29. Barone, V. & Cossi, M. Quantum Calculation of Molecular Energies and Energy Gradients in Solution by a Conductor Solvent Model. *J. Phys. Chem. A.* **102**, 1995-2001 (1998).
30. Eichkorn, K., Treutler, O., Öhm, H., Häser, M. & Ahlrichs, R. Auxiliary basis sets to approximate Coulomb potentials. *Chem. Phys. Lett.* **242**, 652-660 (1995).
31. Whitten, J. L. Coulombic potential energy integrals and approximations. *J. Chem. Phys.* **58**, 4496-4501 (1973).
32. Lu, T. & Chen, F. Multiwfn: A multifunctional wavefunction analyzer. *J. Comput. Chem.* **33**, 580-592 (2012).
33. Humphrey, W., Dalke, A. & Schulten, K. VMD: Visual molecular dynamics. *J. Mol. Graph.* **14**, 33-38 (1996).
34. CYLview 1.0b; Legault, C. Y. Université de Sherbrooke, (http://www.cylview.org). (2009).

35. Isse, A. A. & Gennaro, A. J. *J. Phys. Chem. B*, **114**, 7894-7899 (2010).
36. Sun, J., Perfetti, M. T. & Santos, W. L. A Method for the deprotection of alkylpinacolyl boronate esters. *J. Org. Chem.*, **76**, 3571- 3575 (2011).
